# Exploring the active ingredients and pharmacological mechanisms of the oral intake formula Huoxiang Suling Shuanghua Decoction on influenza virus type A based on network pharmacology and experimental exploration

**DOI:** 10.3389/fmicb.2022.1040056

**Published:** 2022-11-01

**Authors:** Ruying Tang, Linyuan Wang, Jianjun Zhang, Xinyu Li, Lingyun Tan, Wei He, Hui Han, Yuan Liu, Keyu Wang, Mengyao Wang

**Affiliations:** ^1^School of Chinese Materia Medica, Beijing University of Chinese Medicine, Beijing, China; ^2^School of Traditional Chinese Medicine, Beijing University of Chinese Medicine, Beijing, China; ^3^Department of Immunology, School of Basic Medical Sciences, Anhui Medical University, Hefei, China; ^4^Clinical Basic Teaching and Research Office of Medical College, Qingdao Binhai University, Qingdao, China; ^5^College of Biological Sciences, China Agricultural University, Beijing, China; ^6^Solarbio Life Sciences, Beijing, China; ^7^Department of Clinical Laboratory, The Second Medical Center of Chinese PLA General Hospital, National Clinical Research Center for Geriatric Diseases, Beijing, China

**Keywords:** Huoxiang Suling Shuanghua Decoction, influenza virus type A, UPLC/Q-TOF MS, systems pharmacology, TLR4/NF-κB p65 signaling pathway, HIF-1α/IL17 signaling pathway

## Abstract

**Objective:**

To investigate the active ingredients, underlying anti-influenza virus effects, and mechanisms of Huoxiang Suling Shuanghua Decoction (HSSD).

**Materials and methods:**

The therapeutic effect of HSSD were confirmed through the survival rate experiment of H1N1-infected mice. Then, the HSSD solution and the ingredients absorbed into the blood after treatment with HSSD in rats were identified by UPLC/Q-TOF MS, while the main contents of ingredients were detected by high performance liquid chromatography (HPLC). Next, a systems pharmacology approach incorporating target prediction, gene ontology (GO) enrichment, kyoto encyclopedia of genes and genomes (KEGG) pathway analysis, and molecular docking were performed to screen out the active compounds and critical pathways of HSSD in treating influenza. According to prediction results, real-time quantitative polymerase chain reaction (RT-qPCR) and immunohistochemistry assay were used to detect the mRNA and protein expression levels of critical targets in H1N1-infected mice lungs.

**Results:**

Huoxiang Suling Shuanghua Decoction improved the survival rate of H1N1-infected mice and prolonged the mice’s lifespan. Besides, HSSD exerts an antivirus effect by decreasing the levels of hemagglutinin (HA) and nucleoprotein (NP) to inhibit the replication and proliferation of H1N1, reducing the lung pathological state, inhibiting the cell apoptosis in the lung, and regulating the abnormal responses of peripheral blood, including GRA, LYM, white blood cell (WBC), PLT, and hemoglobin (HGB). Then, 87 compounds in the HSSD solution and 20 ingredients absorbed into the blood after treatment with HSSD were identified. Based on this, combined with the network analysis and previous research on antivirus, 16 compounds were screened out as the active components. Moreover, 16 potential targets were predicted by network pharmacology analysis. Next, molecular docking results showed stable binding modes between compounds and targets. Furthermore, experimental validation results indicated that HSSD regulates the contents of Immunoglobulin A (IgA), Immunoglobulin M (IgM), and Immunoglobulin G (IgG) in serum, modulating the levels of IFN-γ, IL-6, IL-10, MCP-1, MIP-1α, and IP-10 in the lung tissue, and significantly decreasing the mRNA and protein expressions of TLR4, CD14, MyD88, NF-κB p65, HIF1 α, VEGF, IL17A, and IL6 in the lung tissue.

**Conclusion:**

Huoxiang Suling Shuanghua Decoction exerts an anti-influenza effect by affecting the expressions of mRNA and protein including TLR4, CD14, MyD88, NF-kB p65, HIF-1α, VEGF, IL17A, IL6, and inhibiting the accumulation of inflammation. Our study provided experimental pieces of evidence about the practical application of HSSD in treating influenza.

## Introduction

Influenza is an acute respiratory infectious disease caused by an influenza virus infection, which has the characteristics of high pathogenicity and explosiveness, resulting in seriously endangering human health (Esper et al., 2011). In a relatively confined environment, there easily occurred an outbreak of influenza, and the old, weak, and pregnant are all high-risk groups of infected people. Worldwide, as many as 290,000–650,000 influenza patients die each year, and 3–5 million influenza patients develop severe diseases (Zimmerman et al., 2014). Influenza virus is a kind of orthomyxovirus, which is an RNA virus that causes influenza in humans and animals. Based on the differences between nuclear proteins and matrix proteins in the composition structure of the influenza virus, the influenza virus can be divided into three types (Caini et al., 2018). However, influenza is mainly caused by influenza A and B viruses, among which influenza A virus is the most important pathogen causing influenza epidemics (Hurt et al., 2004). In a previous clinical research report, the lowest survival rates were observed in patients with H1N1 infection after comparing with different strains of the influenza virus (Murillo-Zamora et al., 2021). Therefore, the influenza virus type A infection remain an urgent issue around the world.

Most of the influenza patients are accompanied by severe pneumonia and heart, kidney, and another organ failure that lead to death in severe cases, thus causing a high mortality rate during the severe period of illness (Lee and Lee, 2010). Commonly like many viruses, influenza virus type A relies on host factors and signaling pathways for replication, which may provide alternative options for treating infections. Nowadays, influenza vaccination is the intervention strategy of choice to prevent influenza, but its effectiveness is reduced in older adults and infants who are most vulnerable to severe or fatal influenza infections (Elbahesh et al., 2019). In addition, the antigenic variation of influenza virus type A complicates the production of an effective vaccine (Vemula et al., 2017). Similarly, the effectiveness of currently used antiviral drugs, including oseltamivir, ribavirin, amantadine, etc., is threatened by the development of resistance (Pizzorno et al., 2011). Compared with chemical drugs, traditional Chinese medicine (TCM) with thousands of years of history application in preventing and treating influenza has some advantages in the aspect of few adverse reactions and less drug resistance (Wei et al., 2019). In addition, TCM plays an important role in antiviral by killing the virus directly, inhibiting the replication of the influenza virus, alleviating the inflammatory reaction, and enhancing the body’s immunity (Li et al., 2022).

Huoxiang Suling Shuanghua Decoction (HSSD) is a TCM formula that has been formulated and used clinically to prevent and treat influenza by experienced clinicians at the Beijing University of Chinese Medicine after the outbreak of COVID-19. HSSD was formulated by the classical combination formula of Huoxiang Zhengqi Powder (HZP) and Er Xiang Powder (EXP) for treating influenza. HZP was recorded in the “Prescriptions People’s Welfare Pharmacy” in ancient China, while EXP was used during China’s Ming dynasty, and both HZP and EXP have clinical applications in influenza infection until now. Oral Chinese patent medicine Huoxiang Zhengqi dropping pills, whose prescription originated from HZP, were used to prevent and treat influenza, and results showed good curative effects according to previous clinical research reportings (Xiao et al., 2020). Similarly, HSSD has some clinical evidence not only in the TCM theory record but also in its therapeutic effects on influenza in population prevention applications. For instance, during the time of the outbreak of COVID-19 and influenza season, HSSD was widely used for the prevention and treatment of influenza in universities and communities in Beijing and volunteers reported no adverse reactions after the application of HSSD. Therefore, we conducted basic research on HSSD for the exploration of the efficacy and mechanism of HSSD in treating influenza in this study to provide an experimental piece of evidence for the clinical application of HSSD.

**FIGURE 1:**
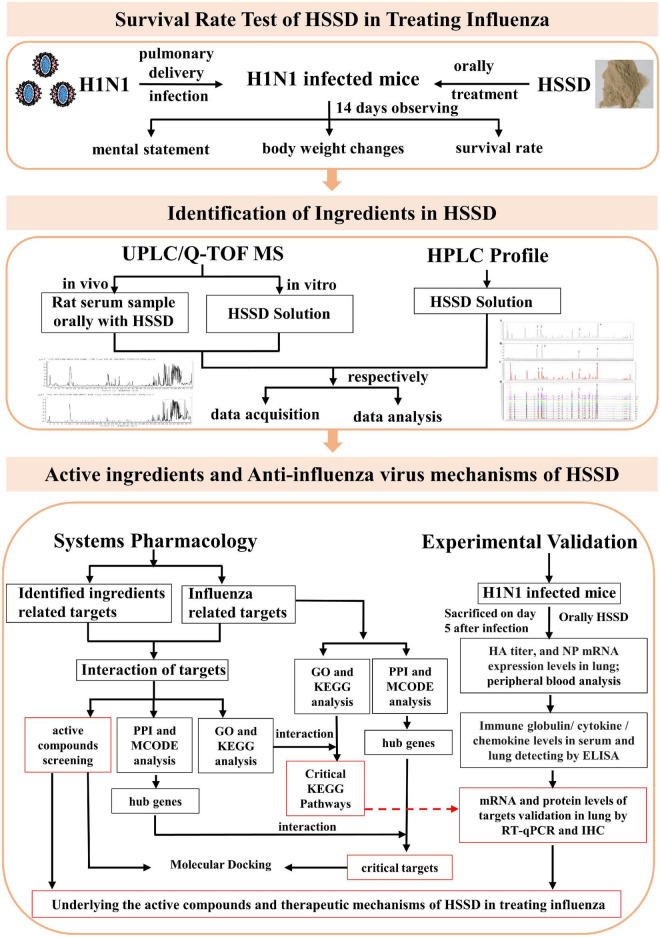
The scheme of exploring the active ingredients and anti-influenza virus effect mechanisms of HSSD.

Network pharmacology refers to the construction of a “drug–target–gene–disease” network through the interaction and integration of systems biology, pharmacology, information network science, and computer science, by which the interactive relationship between drugs and diseases is studied through network analysis, processing, exploring, and predicting the mechanisms of the drug in treating diseases (Ye et al., 2021; Khan and Lee, 2022). Gradually, network pharmacology has become an effectively used tool in investigating the therapeutic mechanisms of the TCM formula. In our study, we investigated the therapeutic effect and mechanisms of HSSD in treating influenza by employing a systems pharmacology approach combined with animal experimental validation. The detailed study steps are shown in Figure 1. First, the A/PR8/34 (H1N1) virus-infected influenza virus type A mice model was used to evaluate the survival rate and antiviral effect of HSSD. Next, methods of high performance liquid chromatography (HPLC) profile and UPLC/Q-TOF MS were performed to identify the compounds in the HSSD solution directly and the ingredients absorbed into the blood after HSSD administration orally. Based on this compounds’ information, target prediction and enrichment analysis were carried out to systematically predict the underlying active compounds and potential pathways of HSSD in treating influenza. Then molecular docking was used for preliminary verification of the network pharmacology results between active compounds and critical protein targets. Finally, the critical kyoto encyclopedia of genes and genomes (KEGG) signaling pathway of HSSD in the treatment of influenza was experimentally validated. Our study explored the understanding of the effective substances and mechanisms of HSSD in treating influenza and contributing to the clinical development and application of HSSD.

## Materials and methods

### Chemicals and materials

Huoxiang Suling Shuanghua Decoction (batch No. 210101) was provided by Tianjin Zhongwei Hezhi Pharmaceutical Co., Ltd. (Beijing, China). The herbal prescription of HSSD consists of 15 TCM, which were obtained from Beijing Chenyi Pharmaceutical Co., Ltd., (Beijing, China) and identified by Professor Chun Wang (School of Traditional Chinese Medicine, Beijing University of Chinese Medicine). The detailed information is as follows: Pogostemonis Herba [Lot No. 190820002, the dry aboveground portion of *Pogostemon cablin* (Blanco) Benth., Lamiaceae family], Moslae Herba (Lot No. 191014002, the dry aboveground portion of *Mosla chinensis* Maxim. or *Mosla chinensis* “Jiangxiangru,” Lamiaceae family.), Polygonati Rhizoma (Lot No. 190315005, the dry rhizoma of *Polygonatum kingianum* Coll. et Hemsl., *Polygonatum sibiricum* Red., or *Polygonatum cyrtonema* Hua, Lily family.), Perillae Folium [Lot No. 191226002, dried leaves (or with shoots) of *Perilla frutescens* (L.) Britt., Lamiaceae family.], Tsaoko Fructus (Lot No. 181203003, the desiccative ripe fruit of *Amomum tsao-ko* Crevost et Lemaire, Zingiberaceae.), Poria [Lot No. 200122004, the dry sclerotia of Poria cocos (Schw.) Wolf, polyporaceae.], Citri Reticulatae Pericarpium (Lot No. 200105001, the dry mature peel of *Citrus reticulata* Blanco, rue family.), Radix Fici Hirtae (Lot No. 200730003, the dry root of *Ficus hirta* Vahl, Moraceae.), Platycodonis Radix [Lot No. 190923004, the dry root of *Platycodon grandiflorum* (Jacq.) A. DC., Campanulaceae.], Zingiberis Rhizoma Recens (Lot No. 190312007, the fresh rhizomes of *Zingiber officinale* Rosc., Zingiberaceae.), Jujubae Fructus (Lot No. 200601001, the desiccative ripe fruit of *Ziziphus jujuba* Mill, rhamnaceae.), Lonicerae Japonicae Flos (Lot No. 200726001, the dried buds or with opening flowers of *Lonicera japonica* Thunb, Caprifoliaceae.), Eriobotryae Folium [Lot No. 181009007, the dry leaves of *Eriobotrya japonica* (Thunb.) Lindl., Rosaceae.], Mori Folium (Lot No. 191203002, the dry leaves of *Morus alba* L., Moraceae.), and Raphani Semen (Lot No. 190917002, the dry mature seed of *Raphanus sativus* L., Brassicaceae.). Besides, oseltamivir (Lot No. 0222001036, Yichang HEC Changjiang Pharmaceutical Co., Ltd., China) was taken as a positive control. Reference standards (purity ≥ 98%) chlorogenic acid (110753–202018) was purchased from National Institutes for Food and Drug Control (Beijing, China), while caffeic acid (M27GB143417), scutellarin (Z01M10 × 81701), and rosmarinic acid (Y06A9K67402) were purchased from Shanghai Yuanye Biotechnology Co., Ltd. (Shanghai, China). HPLC-grade acetonitrile (I1098029022), methanol (I1144135113), and formic acid (Y6170039) were supplied by Merck (Darmstadt, Germany).

### Animals and virus

Female BALB/c mice [6 weeks old, 15–17 g, certificate no. SCXK (Jing) 2011–0004] and adult male Sprague-Dawley rats [12-week-old, 180–200 g, certificate no. SCXK (Jing) 2016–0002] were obtained from SPF (Beijing) Biotechnology Co., Ltd., and then fed in a standard animal room (room temperature: 20–4°C; relative humidity: 30–40%; and light condition: 12-h dark/light cycle). All animal experiments were performed according to protocols approved by the Welfare and Ethical Inspection in the Beijing University of Chinese Medicine Animal Care Committee (No. BUCM-4-2021071601-3022).

Influenza A/PR/8/34 (H1N1) virus was propagated in the allantois of embryonated chicken eggs for 48 h at 35°C and then for 12 h at 4°C. The produced virus was stored at -80°C until infection experiments. Before this study, a 50% tissue culture infectious dose (TCID_50_) according to the Reed–Muench formula of influenza A virus strain for MDCK cells was calculated to be 10^–4^/100 μL. Besides, LD_50_, according to the Reed–Muench formula of influenza A virus strain for BALB/c mice infected the serial dilutions of the stock virus liquid by lung fluid quantification nebulizer (HRH-MAG4, Beijing Huironghe Technology Co., Ltd., Beijing, China), was calculated to be 10^–3^.^81^/50 μL. In this present study, mice were inoculated with the H1N1 virus with a lung fluid quantification nebulizer in the lung at a lethal dose of 4 LD_50_ to test the anti-influenza effect of HSSD.

### Huoxiang Suling Shuanghua Decoction preparation and high performance liquid chromatography analysis

Huoxiang Suling Shuanghua Decoction was manufactured in Tianjin Zhongwei Hezhi Pharmaceutical Co., Ltd., (Beijing, China) as follows: a mixture of Pogostemonis Herba (30 kg), Moslae Herba (20 kg), Polygonati Rhizoma (40 kg), Perillae Folium (30 kg), Tsaoko Fructus (20 kg), Poria (40 kg), Citri Reticulatae Pericarpium (30 kg), Radix Fici Hirtae (40 kg), Platycodonis Radix (20 kg), Zingiberis Rhizoma Recens (30 kg), Jujubae Fructus (30 kg), Lonicerae Japonicae Flos (12 kg), Eriobotryae Folium (20 kg), Mori Folium (20 kg), and Raphani Semen (10 kg) at a weight ratio of 1.5:1:2:1.5:1:2:1.5:2:1:1.5:1.5:0.6:1:1:0.5 were soaked for 1 h with 10-fold of water (volume/weight). Then, the soak was extracted by refluxing for 2 h as the first time extraction, the volatile oil, and condensed aromatic water were collected for standby application, while the liquid extract was filtered to obtain the filtrate A. Next, eightfold of water (volume/weight) was added into the filter residue and boiled again for 1 h, then the liquid extract was filtered to obtain the filtrate B. Subsequently, filtrate A and filtrate B were combined and concentrated under reduced pressure (temperature 65–70^°^C, vacuum degree-0.06 ∼ -0.08 MPa) to the concentrated solution with a relative density of 1.06 (50^°^C), then using spray dried method (moisture ≤ 5%, inlet temperature 195^°^C, and outlet temperature 90^°^C) to yield powder A. Besides, the above volatile oil and condensed aromatic water were wrapped in β-cyclodextrin (6.3 kg), and dried in a hot air circulation oven (45∼50^°^C drying for about 7–8 h, moisture ≤ 5%) to obtain powder B. Finally, HSSD was obtained by mixing powder A and powder B in a 3D mixing jar for 30 min (moisture ≤ 5%). According to the daily prescription amount of HSSD, which was calculated to be 19.6 g/day of the mixture TCM crude drug for one person, combined with the obtained powder A and powder B, the daily oral fine powder of HSSD for one person was calculated to be 5.58 g/day of the mixture powder (5.36 g/day powder A and 0.22 g/day powder B). Then, the HPLC analysis of HSSD was performed on the Waters 2,695 HPLC system (Waters Technologies) with Agilent Zorbax SB-C18 column (4.6 mm × 250 mm, 5 μm) at a flow rate of 0.8 ml/min and 30^°^C column temperature. The mobile phase was composed of solvent A (acetonitrile) and solvent B (water—0.1% phosphoric acid): 0–80 min, 5–21% A; 80–95 min, 21–30% A; 95–103 min, 30–42% A; 103–110 min, 42–60% A; 110–115 min, maintained at 98% A. The injection volume was 10 μL and the wavelength was set at 320 nm.

### Components identification of Huoxiang Suling Shuanghua Decoction by using UPLC/Q-TOF MS analysis

Components identification of HSSD was performed by UPLC/Q-TOF MS analysis including two parts of detection. We first identified the ingredients of HSSD using an HSSD solution, then the analysis of serum samples (given orally in rats) after HSSD was performed subsequently to identify the ingredients of HSSD absorbing into the blood. Detected samples were prepared as follows: First, 2 ml HSSD solution (25.11 mg/ml) was added with 2 ml 20% methanol to dilute, shake, and centrifuge (12,000 rpm, 5 min), and the supernatant was obtained as the HSSD solution sample for liquid chromatography coupled with mass spectrometry (LC-MS) analysis. Next, after 7 days of adaptive feeding, eight SD rats were randomly divided into the blank group (*n* = 4) and the experiment group (*n* = 4). The HSSD was administrated orally at a single dose of 10,044 mg/kg body weight (20-fold clinical doses; the daily dose of HSSD powder for human adults is 93 mg/kg/day and the equivalent dose ratio of human and rat was 0.018 according to the body surface area) to the experiment group. At the same time, an equivalent of distilled water was given to the blank group. For the collection of serum samples, the rats were anesthetized by the method of isoflurane inhalation after oral administration of the HSSD. The blood samples were collected from the orbital venous blood at 0.5, 1, 2, and 4 h, respectively. The collected samples were centrifuged at 4,000 rpm for 15 min at 4°C and then mixed to obtain a pooled serum. The blank serum was collected in the same way. A volume of 4 ml serum samples was immediately added with 12 ml methanol to precipitate serum proteins. After centrifuging at 12,000 rpm for 15 min at 4°C, the supernatant was concentrated and dried. Before analysis, the residue was redissolved with 100 μL 80% methanol, mixing by 5 min vortexing. Finally, the supernatant was obtained as the serum sample for LC-MS analysis after centrifuging the residue redissolved solution for 15 min (12,000 rpm, 4°C).

UPLC/Q-TOF MS method was performed to identify the compounds of HSSD. The analysis of the prepared HSSD solution sample was performed by Waters H-Class UPLC (Waters Technologies Co., Ltd.) and AB Sciex Triple TOF^®^ 4600 LC-MS (SCIEX Co., Ltd.) with both ESI-Negative/Positive ion mode. Samples were separated by the Waters CORTECS^®^UPLC^®^ T3 (2.1 mm × 100 mm, 1.6 μm), the temperature was maintained at 30°C, and the injection volume was 2 μL. The mobile phase consisted of acetonitrile (A), and water–0.1% formic acid (B) (100% B at 0–5 min, 100–95% B at 5–10 min, 95–85% B at 10–30 min, 85–80% B at 30–47 min, 80–55% B at 47–62 min, 55–10% B at 62–68 min, maintained 10% B at 68–73 min, 10–100% B at 73–73.1 min, maintained 100% B at 73.1–75 min) and the flow rate was 0.3 ml/min. The detection wavelength was 254 nm, 190–400 nm. The Mass parameters (Sciex Triple TOF 4600 LC-MS) conditions were as follows: the MS parameters including TOF mass range, 50-−1,700; ion source gas 1, 50 psi; ion source gas 2, 50 psi; curtain gas, 35 psi; ion spray voltage floating, −4,500/5,000; ion source temperature, 500^°^C; declustering potential, ±100 V; collision energy, ±10 eV. And the MS/MS parameters include MS/MS mass range, 50–1,250; declustering potential, ±100 V; collision energy spread, 20 eV; ion release delay, 30 ms; ion release width, 15 ms. The data collection software was Analyst TF 1.7.1. While the data processing software was Peak View 1.2.

Subsequently, the UPLC/Q-TOF MS method was also used to identify the serum compounds. The analysis of the prepared serum sample after HSSD was taken orally in rats was performed by Agilent 1290 UPLC and Agilent Q-TOF 6545 LC-MS (Agilent Technologies Co., Ltd.) with both ESI-negative/positive ion modes. Samples were separated by the Waters CORTECS^®^UPLC^®^ T3 (2.1 mm × 100 mm, 1.6 μm), the temperature was maintained at 30°C, and the injection volume was 5 μL. The mobile phase consisted of acetonitrile (A), and water–0.1% formic acid (B) (100% B at 0–5 min, 100–95% B at 5–10 min, 95–85% B at 10–30 min, 85–80% B at 30–47 min, 80–55% B at 47–62 min, 55–10% B at 62–68 min, maintained 10% B at 68–73 min, 10–100% B at 73–73.1 min, maintained 100% B at 73.1–75 min) and the flow rate was 0.3 ml/min. The detection wavelength was 190–400 nm. The mass parameters (Agilent 6545 Q-TOF LC-MS) conditions were as follows: the MS parameters including mass range, 50–1,700; gas temperature, 320°C; drying gas, 8 L/min; nebulizer, 35 psi; sheath gas temperature, 350°C; sheath gas flow, 11 L/min; vcap, 4,000 V; nozzle voltage, 1,000 V; fragmentor, 175 V; skimmer, 65 V; ref mass, pos. 121.0508&922.0097, and neg. 197.8073&1033.9881. And the MS/MS parameters include MS/MS mass range, 50–1,700; collision energy, ±40 eV. The data collection software was Mass Hunter Workstation Software LC/MS Data Acquisition for 6200 series TOF/6500 series Q-TOF (version B.06.01). While the data processing software was Mass Hunter Workstation Software Qualitative Analysis (version B.07.00).

### Collection of Huoxiang Suling Shuanghua Decoction’s candidate ingredients and its potential targets

Based on the above ingredients analysis results of HSSD by the methods of HPLC and UPLC/Q-TOF MS, combined with their oral bioavailability [OB ≥ 30%, drug likeness (DL) ≥ 0.14] in TCMSP database (Ru et al., 2014; Lai et al., 2020), as well as the pharmacodynamic components information with definite anti-influenza virus effects in the published researches, the candidate components of HSSD were supplemented and collected. Subsequently, the potential targets of the identified ingredients of HSSD were obtained mainly from PubChem (Wang et al., 2012), Swiss Target Prediction (Daina et al., 2019), and BATMAN-TCM (Liu et al., 2016). The HSSD candidate ingredients’ potential targets obtained from the above different databases were put together, removing duplicate targets and retaining the unique targets to get the “complete set of HSSD targets.” Then, UniProt was used to convert the protein name of bioactive ingredients to the gene symbols (UniProt Consortium, 2019). Finally, Cytoscape v3.7.2 software was used to construct the “herb-compounds-targets” network, and the a cytoscape plugin for centrality analysis and evaluation of protein interaction networks (CytoNCA) plug-in function was performed to analyze the network topology attributes and screen the critical compounds and targets of the core network according to the value of degree and betweenness.

### Acquisition of influenza related targets

The known influenza-related targets were obtained mainly from four databases using “influenza” as the keyword. Searching disease databases include DisGeNET (obtained all targets) (Piñero et al., 2021), CTD (obtained targets which score ≥ 10) (Davis et al., 2021), GeneCards (obtained targets which score ≥ 1) (Safran et al., 2010), and GenBank (obtained all targets) (Benson et al., 2018). The influenza-related targets obtained from the above different databases were put together, removing duplicate targets and retaining the unique targets to get the “complete set of influenza targets.” Then, UniProt was used to convert the protein name of bioactive ingredients to the gene symbols (UniProt Consortium, 2019).

### Identification of intersecting targets

The above “complete set of HSSD targets” and “complete set of influenza targets” were intersected to obtain a Venn diagram (Jia et al., 2021) of the “intersected common targets of HSSD & influenza.” Besides, influenza-related targets from different databases were also intersected to obtain a Venn diagram of the “intersected common targets of influenza.”

### Protein-protein interaction and molecular complex detection analysis

Protein-protein interaction (PPI) networks were created respectively by putting the “intersected common targets of HSSD & influenza” and the “intersected common targets of influenza” into the STRING database (setting the organism into Homo sapiens, and confidence score ≥ 0.4) (Szklarczyk et al., 2019). Then, the result of the PPI networks was saved and inputted into Cytoscape v3.7.2 for further analysis. The Cytohubba plug-in was performed for the identification of the top 10 hub genes by using the maximum neighborhood component (MNC) method (Li et al., 2021). Besides, The molecular complex detection (MCODE) plug-in in Cytoscape (Deng et al., 2019) and Metascape (Zhou et al., 2019) was used for the cluster analysis of PPI networks.

### Enrichment analysis and molecular docking analysis

The “intersected common targets of HSSD & influenza” and the “intersected common targets of influenza” were put into the database for annotation, visualization, and integrated discovery (DAVID) (Dennis et al., 2003) to carry out gene ontology (GO) functional enrichment and KEGG pathway enrichment analysis. Then, critical GO and KEGG results of HSSD in treating influenza were identified by intersecting the above two enrichment results of the “intersected common targets of HSSD & influenza” and the “intersected common targets of influenza.” Finally, molecular docking between key active ingredients and critical pathway-related target proteins was performed with the help of the software AutoDock 4.2.6 and the Discovery Studio according to the method reported in a previous study (Li et al., 2021).

### Influenza infection mice experiments and intervention by Huoxiang Suling Shuanghua Decoction

The crude drug daily dose of HSSD for human adults is 0.33 g/kg (0.093 g/kg powder), and the equivalent dose in mice is 10-fold that of adults commonly (Guo et al., 2021), which was calculated to be 3.3 g/kg/day (0.93 g/kg powder). After 7 days of adaption, 60 BALB/c mice were randomly divided into six groups (*n* = 10 per group) as follows: Control normal group (Normal), H1N1-infected group (Infected), Oseltamivir-treated group (Oseltamivir, 25 mg/kg/day, 10-fold of the clinical dose), Huoxiang Suling Shuanghua Decoction low-dose group (HSSD-L, 1.65 g/kg/day, fivefold of the clinical dose), Huoxiang Suling Shuanghua Decoction middle-dose group (HSSD-M, 3.3 g/kg/day, 10-fold of the clinical dose), and Huoxiang Suling Shuanghua Decoction high-dose group (HSSD-H, 6.6 g/kg/day, 20-fold of the clinical dose). Another 60 mice were also randomly divided into six groups for the survival test. Before virus inoculation, mice in the HSSD group were orally prophylaxis with HSSD for 14 days continuously. At the same time, mice in the other groups were treated under similar conditions with a phosphate buffer saline (PBS) medium. On the 15th day of the experiment, except for the control normal mice, mice in other groups were anesthetized with 1% pentobarbital solution (50 mg/kg body weight) by intraperitoneal injection, and then mice were inoculated with H1N1 at a lethal dose of 4 LD_50_ suspended in PBS medium by lung fluid quantification nebulizer in the lung. The day of virus inoculation was defined as day 0. Treatment was initiated 24 h after the virus infection. Mice were gavage once daily with the above medicine from day 0 to day 5 separately. After 5 days of treatment, the lung tissues and blood were collected, while the survival experiment of mice was monitored after virus infection for 14 days (Zhu et al., 2018).

### Body weight, lung wet/dry ratio, and hemagglutinin titer assay

The body weight of mice was recorded during the experiment. Besides, the lung wet/dry weight ratio was tested to quantify the degree of pulmonary edema. Briefly, fresh lung tissues were removed, rinsed in saline, and then weighed to obtain the wet weight. Later, the lung tissue was dried at 80°C for 48 h in an oven to obtain the dry weight. Finally, in accordance with a previous stud calculating as wet/dry ratio = wet weight/dry weight (Peng et al., 2020). Otherwise, the viral load in lung tissues was presented as an hemagglutinin (HA) titer. Briefly, each lung was homogenized to moderate volume suspension with PBS, and 50 μL of homogenates were serially diluted (twofold) in V-bottom 96-well plates and mixed with 50 μL of 1% chicken red blood cell suspension. Plates were incubated at room temperature for 30 min. The HA titer was expressed as the maximum dilution times in which agglutination was observed according to a previous study (Zhu et al., 2018).

### Hematoxylin and eosin assay

After mice were sacrificed, lung tissues were collected and fixed in 4% paraformaldehyde, then embedded in paraffin, sectioned, and finally stained with hematoxylin and eosin (H&E). Under light microscopy, the lung tissue pathological changes were visualized (magnification 100×). Besides, according to reported previous research, two methods of semiquantitative visual scoring (lung injury score) and ImageJ analysis (counts of inflammatory cells and % area occupied) of the lung tissue pathological images were performed to assess the degree of lung injury according to the reported researches (Prakash et al., 2015; Tian et al., 2019).

### Hematologic indices assay

Blood was taken from the mouse orbit. Immediately, using a Mindray automatic animal blood cell analyzer (BC-2800 VET, Shenzhen, China), part of the whole blood was collected in an ethylenediaminetetraacetic acid (EDTA)-treated blood collection tube for the determination of blood parameters, including granulocyte percentage (GRA%), granulocyte absolute value (GRA#), lymphocyte percentage (LYM%), lymphocyte absolute value (LYM#), white blood cell count (WBC), blood platelet count (PLT), hemoglobin (HGB), and red blood cell (RBC).

### Enzyme-linked immunosorbent assay

The serum samples were separated by centrifugation at 3,500 *g* for 10 min at 4°C. Then, Immunoglobulin A (IgA), Immunoglobulin G (IgG), and Immunoglobulin M (IgM) in the serum were measured using commercially available enzyme-linked immunosorbent (ELISA) kits according to the manufacturer’s instructions. These ELISA kits were purchased from Kote Biotechnology Co., Ltd., Jiangsu, China. Besides, Interferon-γ (IFN-γ), Interleukin-6 (IL-6), Interleukin-10 (IL-10), monocyte chemotactic protein 1 (MCP-1), macrophage inflammatory protein 1α (MIP-1α), and interferon-inducible protein-10 (IP-10) in lung homogenates were assayed using ELISA kits according to the manufacturer’s instructions. These ELISA kits were purchased from Boaosen Biotechnology Co., Ltd., Beijing, China.

### Real-time quantitative polymerase chain reaction assay

Levels of toll-like receptor 4 (TLR4), cluster of differentiation 14 (CD14), myeloid differentiation factor 88 (MyD88), nuclear factor-kappa B (NF-κB p65), hypoxia-inducible factor-1α (HIF1α), vascular endothelial growth factor (VEGF), interleukin-17A (IL-17A), interleukin-6 (IL-6), and influenza A virus nucleoprotein (IAV-NP) mRNAs were identified by real-time quantitative polymerase chain reaction (RT-qPCR). Lung tissues from different groups were weighed and dissociated with the Trizol reagent. Next, total RNA was determined by a spectrophotometer (Thermo Scientific, Waltham, MA, USA). Using a Revert Aid First Strand cDNA Synthesis kit (Thermo Fisher Scientific, Waltham, MA, USA), the RNA of each sample was utilized to synthesize the first-strand cDNA. Table 1 shows the sequences for primers which were designed based on published mRNA sequences in the NCBI (National Center for Biotechnology Information), and synthesized by a specialized biotechnology company (Wuhan Servicebio Technology Co., Ltd., Wuhan, China). Next, a Multicolor Real-time PCR Detection System (Bio-Rad Laboratories Inc., CA, Hercules, USA) was used to amplify the cDNA with an SYBR^®^ Green PCR Master Mix (Thermo Fisher Scientific). GAPDH was the internal reference for TLR4, CD14, MyD88, and NF-κB p65. While β-actin was the internal reference for HIF1α, VEGF, IL-17A, IL-6, and IAV-NP. The 2^–Δ^ ^Δ^ ^Ct^ method was used to assess the mRNA expressions.

**TABLE 1:** Primers for RT-qPCR.

Primer	Sequence (5′ to 3′)
TLR4-S	TGAGGACTGGGTGAGAAATGAGC
TLR4-A	CTGCCATGTTTGAGCAATCTCAT
CD14-S	TCAAGTTCCCGACCCTCCAA
CD14-A	GCCCAGTGAAAGACAGATTGAG
MyD88-S	TGACCCCACTCGCAGTTTGT
MyD88-A	TTTGTTTGTGGGACACTGCTTTC
NF-κB p65-S	TCCTTTTCTCAAGCTGATGTGC
NF-κB p65-A	TTTCGGGTAGGCACAGCAAT
GAPDH-S	CCTCGTCCCGTAGACAAAATG
GAPDH-A	TGAGGTCAATGAAGGGGTCGT
HIF1α-S	TTGCTTTGATGTGGATAGCGAT
HIF1α-A	CATACTTGGAGGGCTTGGAGAAT
VEGF-S	AGGAGTACCCCGACGAGATAGA
VEGF-A	CACATCTGCTGTGCTGTAGGAA
IL17A-S	TCCACCGCAATGAAGACCCT
IL17A-A	CATGTGGTGGTCCAGCTTTCC
IL6-S	CCCCAATTTCCAATGCTCTCC
IL6-A	CGCACTAGGTTTGCCGAGTA
IAV-NP-F	GTCAGAATGATCAAACGTGGGA
IAV-NP-R	TACGGCAGGTCCATACACACAG
β-actin-S	GTGACGTTGACATCCGTAAAGA
β-actin-A	GTAACAGTCCGCCTAGAAGCAC

### Immunohistochemistry assay and immunofluorescence staining

Immunohistochemistry (IHC) staining steps were performed as previously described as follows (Zhu et al., 2018): Paraffin sections were dewaxed in water. Then immersing deparaffinized slides in trisodium citrate buffer (pH 6.0) in a water bath at 99°C to perform antigen retrieval. Endogenous peroxidase was quenched with 3% hydrogen peroxide in methanol, and then non-specific binding sites were blocked with 5% BSA. After the removal of BSA, the sections were incubated with primary antibody TLR4, CD14, MyD88, NF-κB p65, HIF1α, VEGF, IL-17A, and IL-6, respectively, at 4°C overnight in a humidified chamber, and then incubated with HRP-conjugated goat anti-rabbit or anti-mouse IgG antibody at 37°C. After quenching of endogenous peroxidase, the sections were blocked with 5% BSA for 30 min and then incubated with HRP-conjugated goat anti-rabbit IgA antibody at 4°C overnight in a humidified chamber. Slides were visualized using chromogenic substrate solution DAB and counterstained with hematoxylin, and then observed under a microscope. Histochemistry score (H-Score) was used for semi-quantification of target protein staining in lung tissue by converting a number of positives in each section and the intensity of staining into corresponding values. H-Score = Σ (pi × i) = (percentage of weak intensity × 1) + (percentage of moderate intensity × 2) + (percentage of strong intensity × 3), “pi” representing the proportion of positive signal pixel area/cell number, while “i” stands for coloring intensity. H-score value ranged from 0 to 300, and the larger the value, the stronger the comprehensive positive intensity.

For immunofluorescence staining was performed according to previous research (Liu et al., 2020), lung tissue paraffin sections were deparaffinized in xylene, and subsequently hydrated in different concentrations of ethanol, repairing, and blocking endogenous peroxidase. After balancing at room temperature, the reaction liquid was added (Recombinant TDT enzyme:Biotin-dUTP labeling Mix:Equilibration Buffer = 1 μL:5 μL:50 μL), and finally Streptavidin-HRP reaction liquid was added (Streptavidin-HRP: TBST = 1:200). Slides were visualized using chromogenic substrate solution 4’,6-diamidino-2-phenylindole (DAPI), and then observed under a Nikon microscope (NIKON ECLIPSE C1, Japan). The nucleus is blue by labeling with DAPI. Terminal-deoxynucleoitidyl transferase mediated nick end labeling (TUNEL) assay kit is labeled with fluorescein isothiocyanate (FITC), and positive apoptosis cells are green. Finally, positive cell density, number/mm^2^ of images, was calculated.

### Statistical analysis

All experimental data were expressed as the means ± SD. Statistical analysis was carried out using the SPSS 20.0 software in a one-way analysis of variance (ANOVA) followed by the Least-Significant Difference (LSD) *post hoc* test or the Dunnett T3 test for comparison of multiple groups. *P* < 0.05 was considered statistically significant.

## Results

### HPLC profile of Huoxiang Suling Shuanghua Decoction

**FIGURE 2:**
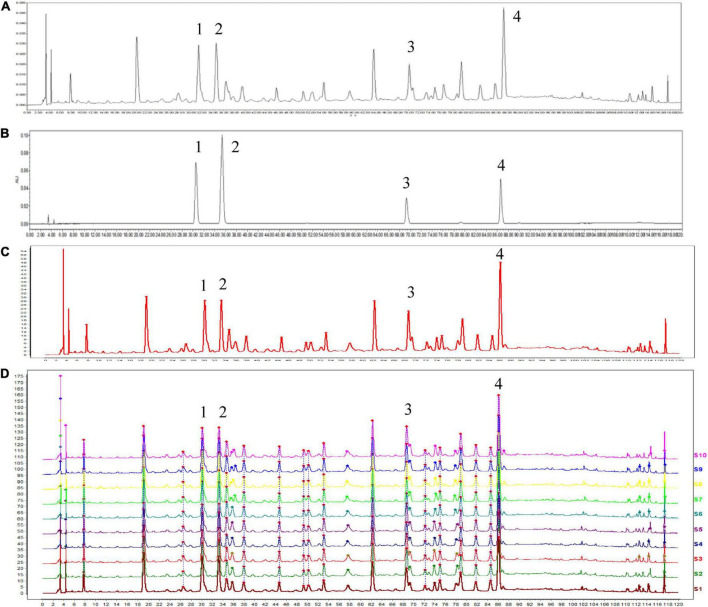
High performance liquid chromatography (HPLC) analysis of HSSD. **(A)** HPLC chromatographic of HSSD. **(B)** HPLC chromatographic of four standard compounds. **(C)** Control chromatographic of HPLC fingerprint. **(D)** Comparability results of reproducibility of HPLC fingerprint analysis in 10 batches of HSSD samples. The main components of HSSD in the chromatograms include (1) chlorogenic acid, (2) caffeic acid, (3) scutellarin, and (4) rosmarinic acid.

Reproducibility analysis of HSSD (10 batches of samples) was conducted in Figure 2 by using the HPLC method, and the results (similarities > 0.97) indicated that the preparation process of HSSD was reasonable and feasible. Four compounds from the HPLC chromatographic peaks, including chlorogenic acid, caffeic acid, scutellarin, and rosmarinic acid, were identified by comparing them with standard references. Besides, the contents of two signature ingredients in the HSSD sample (batch No. 210101) were determined by the HPLC method as chlorogenic acid (0.63 mg/g), and hesperidin (3.13 mg/g), respectively. Quality control results of HSSD by HPLC are shown in Supplementary Datasheet 1.

### Compounds identification results of Huoxiang Suling Shuanghua Decoction solution by UPLC/Q-TOF MS

During composition identification, the mass spectral data were preferentially matched with the Natural Products HR-MS/MS Spectral Library 1.0 database. Then, the compounds were initially screened according to the score information of each chromatographic peak, and then the compounds were further confirmed according to the primary and secondary information of each chromatographic peak. A total of 87 compounds from HSSD were identified finally. The detailed information on the compounds is summarized in Table 2. The extracted ion chromatograms of the 87 compounds in dosed groups and blank groups in positive and negative ion modes and the total ion chromatograms of the HSSD sample are shown in Supplementary Datasheet 2.

**TABLE 2:** The compounds identified in the HSSD solution.

No.	Rt (min)	m/z	m/z actual value	m/z theoretical value	Error (ppm)	Formula	Formula weight	Name
1	3.17	[M-H]^–^	169.0141	169.0142	–0.9	C_7_H_6_O_5_	170.02	Gallic acid
2	3.92	[M + H]^+^	268.1041	268.1040	0.3	C_10_H_13_N_5_O_4_	267.10	Adenosine
3	4.31	[M + H]^+^	144.0477	144.0478	–0.4	C_6_H_9_NOS	143.04	5-(Methylsulfinyl)-4-pentenenitrile
4	4.79	[M-H]^–^	282.0849	282.0844	1.8	C_10_H_13_N_5_O_5_	283.09	Guanosine
5	5.46	[M + H]^+^	330.0599	330.0598	0.3	C_10_H_12_N_5_O_6_P	329.05	Cyclic adenosine monophosphate
6	7.19	[M-H]^–^	153.0194	153.0193	0.4	C_7_H_6_O_4_	154.03	Protocatechuic acid
7	7.88	[M-H]^–^	197.0455	197.0455	–0.2	C_9_H_10_O_5_	198.05	Danshensu
8	10.90	[M + FA-H]^–^	1179.3676	1179.3680	–0.3	C_42_H_70_O_35_	1134.37	Cyclomaltoheptaose
9	11.48	[M + H]^+^	188.0706	188.0706	0.0	C_11_H_9_NO_2_	187.06	Indoleacrylic acid
10	12.24	[M-H]^–^	385.0777	385.0776	0.2	C_16_H_18_O_11_	386.08	D-Glucaric acid 2-[(2E)-3-(4-hydroxy-3-methoxyphenyl)-2-propenoate] (ACI)
11	12.55	[M-H]^–^	353.0880	353.0878	0.5	C_16_H_18_O_9_	354.10	Neochlorogenic acid
12	13.17	[M-H]^–^	385.0775	385.0776	–0.4	C_16_H_18_O_11_	386.08	D-Galactaric acid 2-[(2E)-3-(4-hydroxy-3-methoxyphenyl)-2-propenoate] (ACI)
13	15.15	[M-H]^–^	385.0779	385.0776	0.7	C_16_H_18_O_11_	386.08	Hexaric acid, 3-[3-(4-hydroxy-3-methoxyphenyl)-2-propenoate] (ACI)
14	15.70	[M + H]^+^	405.1392	405.1391	0.2	C_17_H_24_O_11_	404.13	Kingiside
15	16.02	[M-H]^–^	385.0779	385.0776	0.7	C_16_H_18_O_11_	386.08	Hexaric acid, 2-[3-(4-hydroxy-3-methoxyphenyl)-2-propenoate] (ACI) Isomer
16	16.47	[M-H]^–^	179.0350	179.0350	0.1	C_9_H_8_O_4_	180.04	Caffeic acid
17	16.85	[M-H]^–^	353.0875	353.0878	–0.9	C_16_H_18_O_9_	354.10	Chlorogenic acid
18	17.37	[M-H]^–^	375.1299	375.1297	0.6	C_16_H_24_O_10_	376.14	8-Epiloganic acid
19	18.29	[M-H]^–^	353.0878	353.0878	0.0	C_16_H_18_O_9_	354.10	Cryptochlorogenic acid
20	18.49	[M-H]^–^	373.1138	373.1140	–0.6	C_16_H_22_O_10_	374.12	Secologanic acid
21	18.57	[M-H]^–^	385.0777	385.0776	0.2	C_16_H_18_O_11_	386.08	Hexaric acid, 2-[3-(4-hydroxy- 3-methoxyphenyl)-2-propenoate] (ACI) Isomer
22	19.09	[M + FA-H]^–^	595.2612	595.2607	0.8	C_25_H_42_O_13_	550.26	Tricalysionoside A
23	20.51	[M + FA-H]^–^	431.1922	431.1923	–0.2	C_19_H_30_O_8_	386.19	(6S,9S)-Roseoside
24	20.73	M^+^	310.1652	310.1649	1.0	C_16_H_24_NO_5_^+^	310.17	Sinapine
25	20.86	[M + FA-H]^–^	403.1248	403.1246	0.5	C_16_H_22_O_9_	358.13	Sweroside
26	21.57	[M + H]^+^	773.2138	773.2135	0.4	C_33_H_40_O_21_	772.21	Quercetin-7-O-glucoside-3-O-rutinoside
27	22.45	[M + H]^+^	611.1610	611.1607	0.6	C_27_H_30_O_16_	610.15	Luteolin 6,8-di-C-glucoside
28	22.67	[M-H]^–^	365.0874	365.0878	–1.1	C_17_H_18_O_9_	366.10	Psoralenoside
29	22.96	M^+^	342.1709	342.1700	0.3	C_20_H_24_NO_4_^+^	342.17	Magnoflorine
30	23.28	[M + FA-H]^–^	525.1619	525.1614	1.0	C_23_H_28_O_11_	480.16	Paeoniflorin
31	24.53	[M-H]^–^	637.1051	637.1046	0.7	C_27_H_26_O_18_	638.11	Scutellarein-7-O-diglucuronide
32	24.58	[M + H]^+^	595.1667	595.1657	1.6	C_27_H_30_O_15_	594.16	Vicenin II
33	24.77	[M-H]^–^	403.1244	403.1246	–0.5	C_17_H_24_O_11_	404.13	Secoxyloganin
34	25.98	[M + H]^+^	743.2383	743.2393	–1.4	C_33_H_42_O_19_	742.23	Narirutin-4′-glucoside
35	26.26	[M-H]^–^	637.1042	637.1046	–0.7	C_27_H_26_O_18_	638.11	Luteolin-7-O-diglucuronide
36	26.43	[M + H]^+^	627.1551	627.1556	–0.8	C_27_H_30_O_17_	626.15	Quercetin-3,7-diglucoside
37	29.45	[M + H]^+^	623.1247	623.1243	0.7	C_27_H_26_O_17_	622.12	Apigenin-7-O-diglucuronide
38	31.11	[M-H]^–^	609.1465	609.1461	0.6	C_27_H_30_O_16_	610.15	Rutin
39	31.46	[M + H]^+^	463.0873	463.0871	0.4	C_21_H_18_O_12_	462.08	Luteolin-7-O-glucuronide
40	31.51	[M-H]^–^	463.0884	463.0882	0.4	C_21_H_20_O_12_	464.10	Hyperoside
41	31.89	[M-H]^–^	447.0937	447.0933	0.9	C_21_H_20_O_11_	448.10	Luteolin 7-glucoside
42	32.39	[M-H]^–^	187.0975	187.0976	–0.4	C_9_H_16_O_4_	188.10	Azelaic acid
43	33.93	[M + H]^+^	581.1857	581.1865	–1.3	C_27_H_32_O_14_	580.18	Naringin
44	34.67	[M-H]^–^	515.1196	515.1195	0.2	C_25_H_24_O_12_	516.13	Isochlorogenic acid B
45	35.49	[M-H]^–^	515.1196	515.1195	0.2	C_25_H_24_O_12_	516.13	Isochlorogenic acid A
46	36.28	[M-H]^–^	623.1977	623.1981	–0.7	C_29_H_36_O_15_	624.21	Acteoside
47	36.28	[M-H]^–^	445.0776	445.0776	–0.1	C_21_H_18_O_11_	446.08	Apigenin-7-glucuronide
48	37.61	[M + H]^+^	611.1976	611.1970	0.9	C_28_H_34_O_15_	610.19	Hesperidin
49	38.35	[M-H]^–^	359.0767	359.0772	–1.5	C_18_H_16_O_8_	360.08	Rosmarinic acid
50	39.16	[M-H]^–^	753.2242	753.2248	–0.7	C_34_H_42_O_19_	754.23	3,6′-Disinapoyl sucrose
51	39.24	[M-H]^–^	515.1199	515.1195	0.8	C_25_H_24_O_12_	516.13	Isochlorogenic acid C
52	41.28	[M-H]^–^	651.1577	651.1567	1.6	C_29_H_32_O_17_	652.16	Limocitrunshin
53	45.19	[M + H]^+^	301.1072	301.1071	0.5	C_17_H_16_O_5_	300.10	5-Hydroxy-6,7-dimethoxyflavanone
54	47.10	[M-H]^–^	633.2555	633.2553	0.4	C_32_H_42_O_13_	634.26	Obacunone-17-O-β-D-glucoside
55	48.37	[M + FA-2H]^2–^	796.3426	796.3443	–2.2	C_69_H_112_O_38_	1548.68	Platycodin E
56	49.79	[M + H]^+^	595.2017	595.2021	–0.7	C_28_H_34_O_14_	594.19	Poncirin
57	52.92	[M + H]^+^	711.2125	711.2131	–0.8	C_32_H_38_O_18_	710.21	3,5-Dihydroxy-6,7,8,3′,4′-pentamethoxyflavonol-3-O-[6″-(3-hydroxy-3-methylglutaryl)]-glucoside
58	53.23	[M + H]^+^	728.3991	728.4004	–1.8	C_36_H_53_N_7_O_9_	727.39	Citrusin III
59	53.49	[M + H]^+^	901.4797	901.4791	0.6	C_45_H_72_O_18_	900.47	Neosibiricoside D
60	53.50	[M + H]^+^	697.1980	697.1974	0.8	C_31_H_36_O_18_	696.19	acid, 3-hydroxy-3- methyl-,6′-ester with 3-(β-D-glucopyranosyloxy)-7-hydroxy-2-(4-hydroxy-3methoxyphenyl)-5,6,8-trimethoxy-4H-1-benzopyran-4-one (ZCI)
61	53.70	[M-H]^–^	1237.5581	1237.5495	6.9	C_57_H_90_O_29_	1238.56	Platyconic acid A
62	54.10	[M + H]^+^	1225.5868	1225.5848	1.6	C_57_H_92_O_28_	1224.58	Platycodin D
63	54.33	[M-H]^–^	1265.5840	1265.5808	10.4	C_59_H_94_O_29_	1266.59	Platycodin A
64	54.63	[M-H]^–^	1237.5570	1237.5495	6.1	C_57_H_90_O_29_	1238.56	Platycodin J
65	54.83	[M-H]^–^	327.2179	327.2177	0.6	C_18_H_32_O_5_	328.23	Trihydroxy Octadecadienoic acid
66	55.06	[M-H]^–^	327.2177	327.2177	0.0	C_18_H_32_O_5_	328.23	Trihydroxy Octadecadienoic acid
67	55.24	[M + H]^+^	725.2296	725.2287	1.2	C_33_H_40_O_18_	724.22	Natsudaidain-3-O-(3-hydroxy- 3-methylglutarate)-glucoside
68	56.17	[M + H]^+^	683.3996	683.4001	–0.7	C_36_H_58_O_12_	682.39	3-O-β-D-glucopyranosyl platycodigenin
69	56.47	[M + H]^+^	373.1280	373.1282	–0.5	C_20_H_20_O_7_	372.12	Isosinensetin
70	56.59	[M + NH4]^+^	840.4585	840.4587	–0.3	C_39_H_66_O_18_	822.42	Loquatifolin A
71	56.74	[M-H]^–^	329.2330	329.2333	–1.1	C_18_H_34_O_5_	330.24	9,10,11-Trihydroxy-12-octadecanoic acid
72	57.21	[M-H]^–^	329.2332	329.2333	–0.4	C_18_H_34_O_5_	330.24	9,12,13-Trihydroxy-10-octadecanoic acid
73	57.65	[M + H]^+^	403.1389	403.1387	0.4	C_21_H_22_O_8_	402.13	5,6,7,3′,4′,5′-Hexamethoxyflavone
74	57.83	[M + H]^+^	285.0758	285.0758	0.2	C_16_H_12_O_5_	284.07	Wogonin
75	58.32	[M + H]^+^	373.1278	373.1282	–1.0	C_20_H_20_O_7_	372.12	Sinensetin
76	58.38	[M + H]^+^	343.1176	343.1176	0.0	C_19_H_18_O_6_	342.11	4′,5,7,8-Tetramethoxyflavone
77	58.66	[M + H]^+^	471.2013	471.2013	–0.1	C_26_H_30_O_8_	470.19	Limonin
78	59.99	[M-H]^–^	307.1915	307.1915	0.1	C_18_H_28_O_4_	308.20	Methyl-6-gingerol
79	60.31	[M + H]^+^	403.1387	403.1387	–0.1	C_21_H_22_O_8_	402.13	Nobiletin
80	61.55	[M + H]^+^	433.1497	433.1493	0.9	C_22_H_24_O_9_	432.14	3,5,6,7,8,3′,4′-Heptamethoxyflavone
81	62.12	[M-H]^–^	517.3176	517.3171	1.0	C_30_H_46_O_7_	518.32	Corosin
82	62.30	[M + H]^+^	373.1281	373.1282	–0.2	C_20_H_20_O_7_	372.12	Tangeretin
83	62.73	[M + H]^+^	345.0972	345.0969	0.9	C_18_H_16_O_7_	344.09	5,2′-dihydroxy-6,7,8-Trimethoxyflavone
84	62.73	[M-H]^–^	485.3271	485.3272	–0.3	C_30_H_46_O_5_	486.33	Poricoic acid G
85	67.16	[M + H]^+^	455.3520	455.3520	0.1	C_30_H_46_O_3_	454.34	Betulonic acid
86	67.26	[M-H]^–^	497.3269	497.3272	–0.7	C_31_H_46_O_5_	498.33	Poricoic acid A
87	69.50	[M-H]^–^	455.3526	455.3531	–1.0	C_30_H_48_O_3_	456.36	Ursolic acid

### Absorbing compounds of Huoxiang Suling Shuanghua Decoction in blood identification results by UPLC/Q-TOF MS

According to the accurate mass measurements, reference standards, and fragmentation behavior of the UPLC/Q-TOF MS method, as well as the related literature, a total of 20 prototype compounds in rat serum were identified from HSSD. The detailed information on the compounds is summarized in Table 3. The extracted ion chromatograms of the 20 compounds in dosed groups and blank groups in positive and negative ion modes and the total ion chromatograms of the serum sample are shown in Supplementary Datasheet 3.

**TABLE 3:** The compounds identified in rat serum after oral administration of HSSD.

No.	Rt (min)	m/z	m/z actual value	m/z theoretical value	Error (ppm)	Formula	Formula weight	Name
1	5.25	[M + H]^+^	144.0481	144.0483	–1.4	C_6_H_9_NOS	143.04	5-(Methylsulfinyl)-4-pentenenitrile
2	17.69	[M-H]^–^	353.0874	353.0873	0.3	C_16_H_18_O_9_	354.10	Chlorogenic acid
3	18.09	[M-H]^–^	375.1299	375.1291	2.1	C_16_H_24_O_10_	376.14	8-epiloganic acid
4	19.23	[M-H]^–^	353.0872	353.0873	–0.3	C_16_H_18_O_9_	354.10	Cryptochlorogenic acid
5	19.47	[M-H]^–^	385.0776	385.0771	1.3	C_16_H_18_O_11_	386.08	Hexaric acid, 2-[3-(4-hydroxy-3-methoxyphenyl)-2-propenoate] (ACI) Isomer
6	21.9	[M + FA-H]^–^	403.1246	403.1240	1.5	C_16_H_22_O_9_	358.13	Sweroside
7	24.44	[M + FA-H]^–^	525.1622	525.1608	2.7	C_23_H_28_O_11_	480.16	Paeoniflorin
8	25.71	[M-H]^–^	593.1495	593.1507	–2.0	C_27_H_30_O_15_	594.16	Vicenin II
9	25.92	[M-H]^–^	403.1244	403.1241	0.7	C_17_H_24_O_11_	404.13	Secoxyloganin
10	27.95	[M-H]^–^	637.1023	637.1041	–2.8	C_27_H_26_O_18_	638.11	Luteolin-7-O-diglucuronide
11	30.68	[M-H]^–^	621.1103	621.1092	1.8	C_27_H_26_O_17_	622.12	Apigenin-7-O-diglucuronide
12	33.85	[M-H]^–^	187.0969	187.0971	–1.1	C_9_H_16_O_4_	188.10	Azelaic acid
13	35.46	[M-H]^–^	579.1717	579.1714	0.5	C_27_H_32_O_14_	580.18	Naringin
14	38.19	[M-H]^–^	445.0779	445.0771	1.8	C_21_H_18_O_11_	446.08	Apigenin-7-glucuronide
15	39.35	[M-H]^–^	609.1817	609.1820	–0.5	C_28_H_34_O_15_	610.19	Hesperidin
16	54.89	[M + H]^+^	725.2294	725.2293	0.1	C_33_H_40_O_18_	724.22	Natsudaidain-3-O-(3-hydroxy-3-methylglutarate)-glucoside
17	56.21	[M + H]^+^	373.1287	373.1287	0.0	C_20_H_20_O_7_	372.12	Isosinensetin
18	57.32	[M + H]^+^	403.1384	403.1393	–2.2	C_21_H_22_O_8_	402.13	5,6,7,3′,4′,5′-Hexamethoxyflavone
19	60.01	[M + H]^+^	403.1390	403.1393	–0.7	C_21_H_22_O_8_	402.13	Nobiletin
20	61.25	[M + H]^+^	433.1498	433.1498	0.0	C_22_H_24_O_9_	432.14	3,5,6,7,8,3′,4′-Heptamethoxyflavone

### Protective effects of Huoxiang Suling Shuanghua Decoction in improving the survival of H1N1-infected mice

**FIGURE 3:**
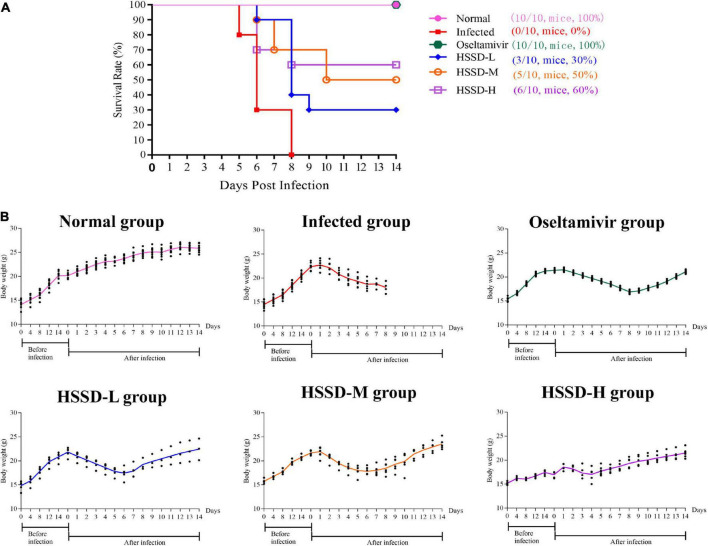
Survival test results. **(A)** Effect of HSSD on the survival rate of H1N1-infected mice. **(B)** Body weight changes of mice in different groups during a survival test experiment.

The therapeutic potential of HSSD against H1N1 infection was evaluated in a mouse model of 4 LD_50_ H1N1 infections. After 3 days of infection with a lethal dose of H1N1, mice showed signs of piloerection, lethargy, weight loss, and reduced food intake. Results in Figure 3A show that the infected group mice were all dead after 8 days of infection with a lethal dose of H1N1 and the survival rate was 0%. While survival rate increased to 100, 30, 50, and 60% after 14 days of infection with a lethal dose of H1N1 in the Oseltamivir group, HSSD-L group, HSSD-M group, HSSD-H group, respectively. Results in Figure 3B show that compared with the normal group, the body weight of the mice in the infected group decreased until death, and oseltamivir or HSSD administration intervention in different dose groups could regain the body weight of mice.

### Compounds and targets screening and the intersecting targets analysis

**FIGURE 4:**
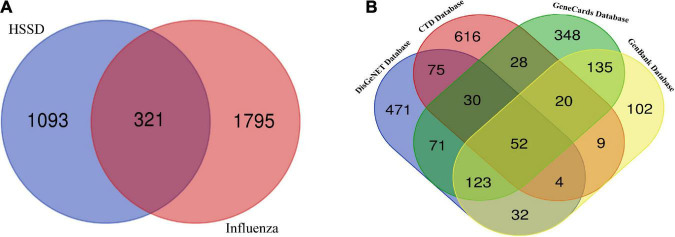
Intersecting targets analysis. **(A)** Common targets of HSSD and influenza. A total of 321 common targets as the “intersected common targets of HSSD and influenza.” **(B)** Common targets of influenza from different databases. A total of 52 targets as the “intersected common targets of influenza.”

According to the results of the survival test, HSSD showed protection in the H1N1-infected mice. Next, we performed network pharmacology and molecular docking to predict the mechanisms of HSSD in treating H1N1. As a result, 357 compounds of HSSD were obtained from the TCMSP database together with the identification from UPLC/Q-TOF MS and HPLC. Then, 241 components of HSSD were obtained by removing the duplicate compounds. Subsequently, 1,414 potential targets of HSSD were collected after removing the duplicate targets. The “herb-compounds-targets” of HSSD were constructed to select the critical compounds and targets according to the value of the network degree. As a result, the top eight critical compounds, including quercetin, beta-sitosterol, ursolic acid, kaempferol, hyperoside, wogonin, luteolin, and acacetin were used for subsequent molecular docking. The top five targets were mainly ESR1, AR, PGR, GABRA3, and NR1I2. Detailed information on the network of “herb-compounds-targets” of HSSD is shown in Supplementary Datasheet 4. Besides, in Figure 4A, we obtained core 321 targets after intersecting 1414 targets of HSSD and 2116 targets of influenza as the “intersected common targets of HSSD & influenza.” In Figure 4B, we obtained core 52 targets after intersecting influenza targets getting from different disease databases as the “intersected common targets of influenza.” Detailed target information is shown in Supplementary Datasheet 5.

### PPI network construction and analysis

The PPI networks and its clusters analysis networks were established. The “intersected common targets of HSSD and influenza” PPI network was constructed. Besides, red color targets were cluster 1 with a score of 14.286, blue color targets were cluster 2 with a score of 12.936, green color targets were cluster 3 with a score of 11.778, purple color targets were cluster 4 with a score of 4.000, orange color targets were cluster 5 with a score of 3.636, and yellow color targets were cluster 6 with a score of 3.000. Besides, the “intersected common targets of influenza” PPI network was constructed, and its MCODE analysis resulted in two clusters. In these networks, the darker the color, the larger the circle, indicating that the targets occupied an important position in the network. Subsequently, hub genes were screened out from these two PPI networks. Finally, the top 10 critical hub genes of HSSD in treating H1N1 were obtained including TNF, IL6, IL1B, TLR4, CCL2, CXCL8, IL10, STAT1, IFNG, and CXCL10. Detailed information on PPI and MCODE analysis results is shown in Supplementary Datasheet 6.

### Gene ontology and kyoto encyclopedia of genes and genomes enrichment analysis

**FIGURE 5:**
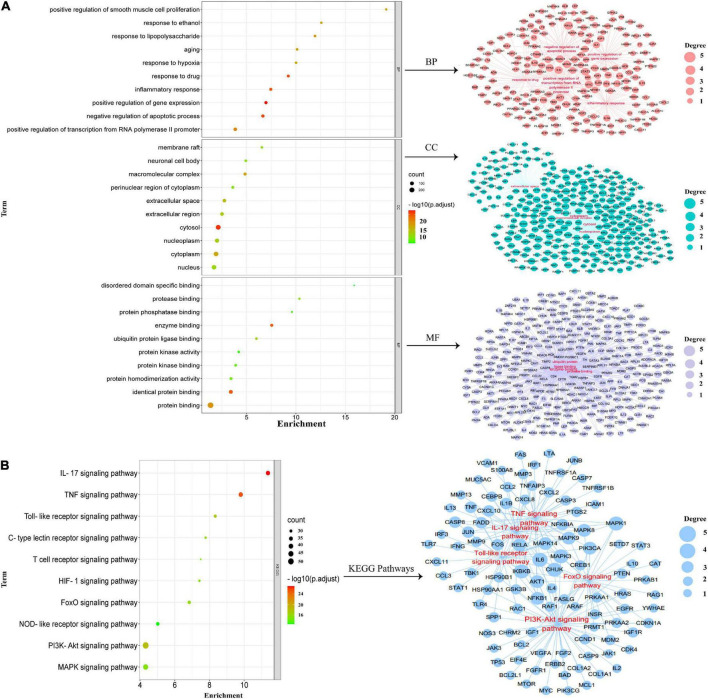
Gene ontology (GO) enrichment analysis. **(A)** Critical GO enrichment results were obtained by intersecting “intersected common targets of HSSD and influenza” GO results and “intersected common targets of influenza” GO results. **(B)** Critical kyoto encyclopedia of genes and genomes (KEGG) enrichment signaling pathways results were obtained by intersecting “intersected common targets of HSSD and influenza” KEGG results and “intersected common targets of influenza” KEGG results.

The “intersected common targets of HSSD and influenza” enrichment analysis of GO terms was performed with 1,049 BPs, 146 CCs, and 204 MFs. While the “intersected common targets of influenza” enrichment analysis of GO terms was performed with 305 BPs, 23 CCs, and 42 MFs. After intersecting, “intersected common targets of HSSD & influenza” GO results and “intersected common targets of influenza” GO results, critical top 10 GO enrichment results and their networks are obtained in Figure 5A. According to the critical GO enrichment analysis, the gene targets associated with biological process (BP) are embroiled in positive or negative regulation of gene expression, response to drugs, inflammatory response, positive regulation of transcription from RNA polymerase II promoter, etc. Gene targets in cellular component (CC) are primarily found in the cytosol, macromolecular complex, cytoplasm, extracellular space, nucleoplasm, etc. While molecular function (MF) is mainly revealed in enzyme binding, identical protein binding, protein binding, ubiquitin protein ligase binding, protease binding, etc. In conclusion, after intersecting “intersected common targets of HSSD and influenza” GO results and “intersected common targets of influenza” GO results, the critical top 10 GO enrichment demonstrated that HSSD treating influenza mainly revealed in the regulation of the drug, inflammatory response, and the regulation of apoptosis, cytokines, protein binding, etc.

Besides, the “intersected common targets of HSSD and influenza” enrichment analysis of 186 KEGG results was performed. While the “intersected common targets of influenza” enrichment analysis of 96 KEGG results was performed. After intersecting “intersected common targets of HSSD & influenza” KEGG results and “intersected common targets of influenza” KEGG results, critical top 10 KEGG signaling pathways and their networks are obtained in Figure 5B. The critical top 10 KEGG signaling pathways mainly revealed in the IL-17 signaling pathway, TNF signaling pathway, PI3K-Akt signaling pathway, Toll-like receptor signaling pathway, FoxO signaling pathway, C-type lectin receptor signaling pathway, HIF-1 signaling pathway, MAPK signaling pathway, T-cell receptor signaling pathway, and NOD-like receptor signaling pathway. In brief, GO and KEGG enrichment analysis results suggested that HSSD treats influenza mainly through combining multiple components with multiple influenza virus proteins, acting on multiple targets, and regulating multiple pathways, which include inflammatory response, immune response, cell apoptosis and influenza virus replication, as well as the IL-17, TNF, PI3K-Ak, and Toll-like receptor signaling pathways to achieve the effect of treatment of influenza. Detailed GO and KEGG enrichment information is shown in Supplementary Datasheets 7, 8.

### Molecular docking analysis

**FIGURE 6:**
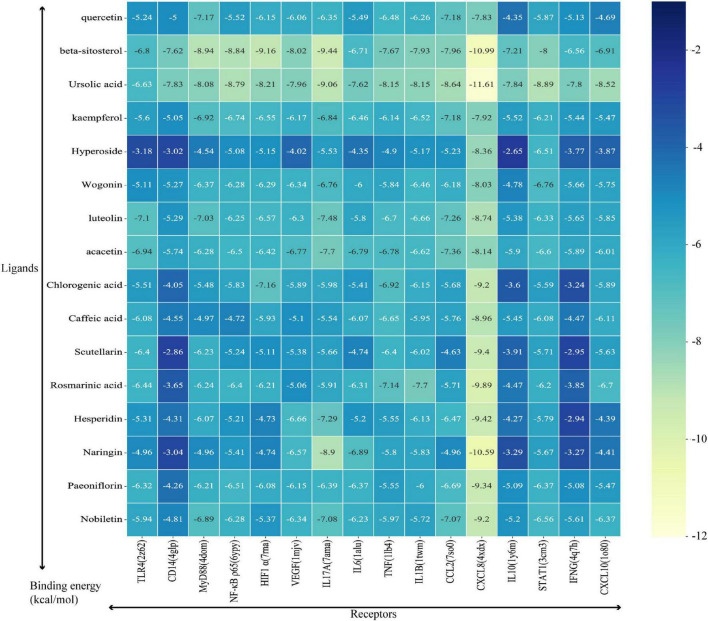
Binding energy between ligands (critical compounds) and receptors (critical targets).

The critical KEGG signaling pathway IL-17 signaling pathway and Toll-like receptor signaling pathway were selected for our study to explore the mechanisms of HSSD in treating H1N1 according to the results of network pharmacology. Besides, combined with the published research, TLR4/NF-κB p65, and HIF-1α/IL17 pathways were tested. Then, the TLR4, CD14, MyD88, NF-κB p65, HIF1 α, VEGF, IL17A, and IL6 in the critical pathways, as well as the TNF, IL1B, CCL2, CXCL8, IL10, STAT1, IFNG, and CXCL10 in the critical hub genes of HSSD in treating H1N1, were molecularly docked with the 16 key active compounds of HSSD, including quercetin, beta-sitosterol, ursolic acid, kaempferol, hyperoside, wogonin, luteolin, acacetin, chlorogenic acid, caffeic acid, scutellarin, rosmarinic acid, hesperidin, naringin, paeoniflorin, and nobiletin. The docking score represents an active compounds (ligands) affinity for core targets (receptors). The lower the docking score, the better the ligand’s interaction with the receptor. Figure 6 displays the outcomes of molecular docking in terms of docking scores between 16 core targets and 16 active compounds. The stable combining mode between 16 core targets and active compounds is shown in Supplementary Datasheet 9.

### Effects of Huoxiang Suling Shuanghua Decoction on body weight changes and lung damage in H1N1-infected mice

**FIGURE 7:**
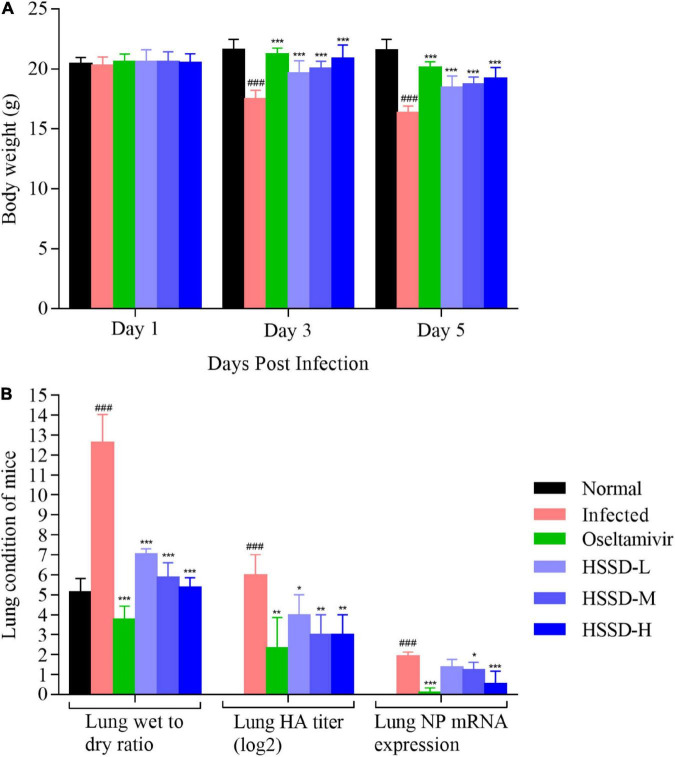
Body weight changes and lung condition of mice. **(A)** Body weight changes of mice on days 1, 3, and 5 after the H1N1 virus infection (*n* = 10). **(B)** Lung wet-to-dry ratio, lung HA titer, and lung NP mRNA expression levels of mice on day 5 after H1N1 influenza virus infection (*n* = 3). Mean ± SD. ^###^*P* < 0.001: significantly differently from the Normal group; *P < 0.05, **P < 0.01, ***P < 0.001: significantly differently from the Infected group.

We recorded the body weight changes and pulmonary damage changes in lung tissue *in vivo* to confirm the therapeutic effect of HSSD in treating H1N1-infected mice. As shown in Figure 7A, after being infected with H1N1 on days 3 and 5, mice in the Infected group lost weight when compared with the Normal group (*P* < 0.001). While mice in other groups significantly increased their body weight after treating with HSSD in different doses compared with the Infected group (*P* < 0.001). In Figure 7B, the wet/dry ratio of the lung was remarkably elevated after being infected with the H1N1 virus in the Infected group (*P* < 0.001). Conversely, the increased lung wet/dry ratio was distinctly attenuated by HSSD (*P* < 0.001). Besides, hemagglutinin (HA) titer and nucleoprotein (NP) mRNA expressions in the lung were determined to verify whether HSSD attenuated viral replication in the respiratory tract *in vivo*. HA and NP were positively correlated with copies of the influenza virus. In Figure 7B, results show that HA titers and NP mRNA expression were significantly increased in H1N1-infected mice (*P* < 0.001). After treatment with HSSD, HA and NP were decreased significantly than the Infected group (*P* < 0.01, *P* < 0.05, or *P* < 0.001).

### Effects of Huoxiang Suling Shuanghua Decoction on lung lesions in H1N1-infected mice

**FIGURE 8:**
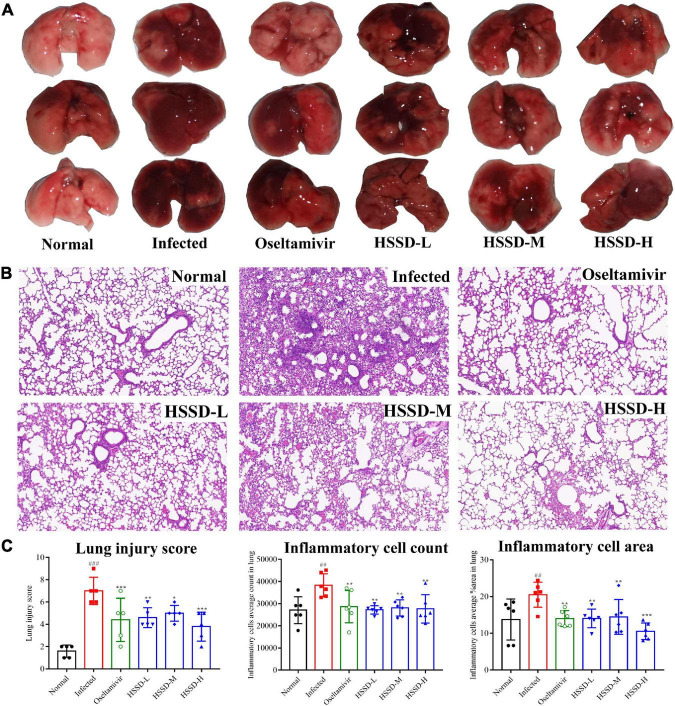
Effects of HSSD on lung lesions in H1N1-infected mice. **(A)** Gross macroscopic examination of lungs in different groups. **(B)** Pathological changes of lung tissues in mice following H&E staining, viewed under light microscopy (magnification 100×). **(C)** Lung injury score, inflammation cells average count, and inflammation cells average area in lung tissues. Mean ± SD, *n* = 3. ^##^*P* < 0.01, ^###^*P* < 0.001: significantly differently from the Normal group; *P < 0.05, **P < 0.01, ***P < 0.001: significantly differently from the Infected group.

We explored the pulmonary gross lesions and pathological changes in lung tissue of H1N1-infected mice to confirm the treatment of HSSD in treating influenza. Figure 8A shows the color of lung tissue in the Normal group was bright pink, while in the Infected group, the lung tissue lesions were dark red and obvious observing the congestion and edematous. After the administration of HSSD or Oseltamivir, the areas of pulmonary injury were decreased at different doses, indicating the remission of lung lesions. Furthermore, pathology changes in lung tissues are shown in Figure 8B, the Normal mice showed thin alveolar walls and intact bronchial epithelium, without secretions in the lumen, or inflammatory cell infiltration in the periphery. While in the Infected mice, the lung tissues observed the alveolar cavity, which was filled with inflammatory and bloody exudates, the alveolar wall structure was unclear, and a large number of lung consolidation areas existed. By comparison with the Infected group, after treatment with HSSD or Oseltamivir, the density of inflammatory cells and the exudate in the alveolar cavity were decreased, and more complete alveolar structures. In Figure 8C, the results of lung injury score, the inflammatory cell count, and the inflammatory cell area in lung tissues of mice treated with HSSD or Oseltamivir were significantly declined compared with the Infected group (*P* < 0.001, *P* < 0.01, or *P* < 0.05). The above results demonstrated that HSSD has a therapeutic effect on influenza viral pneumonia. Original images of lung H&E of three repeats are shown in Supplementary Datasheet 10.

### Effects of Huoxiang Suling Shuanghua Decoction on the apoptosis of infected cells in the lung tissues

**FIGURE 9:**
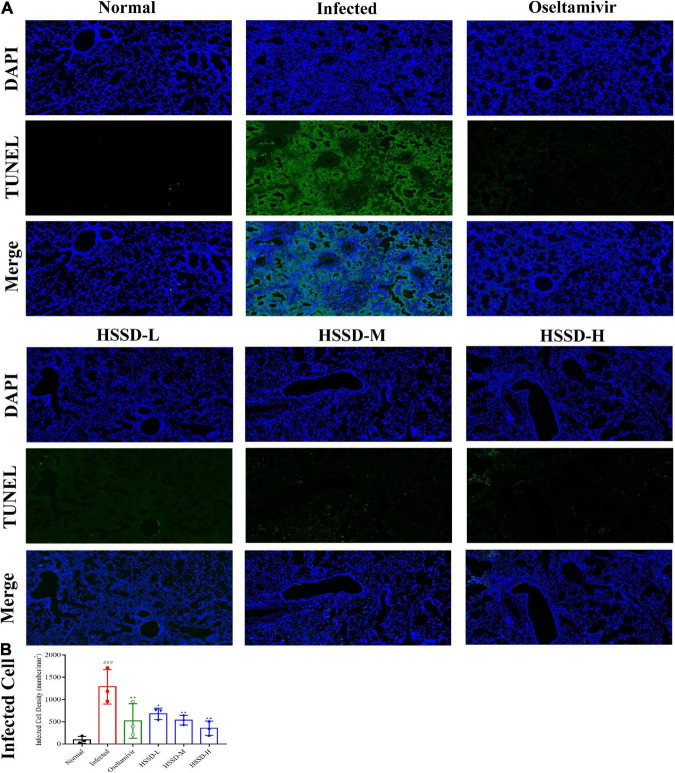
Effects of HSSD on the apoptosis of infected cells in the lung tissues. **(A)** Images of the alteration of the apoptosis of infected cells in the lung tissues of H1N1-infected mice. TUNEL-infected dead cells were stained in green, cell nuclei were labeled with DAPI in blue (magnification 100×). **(B)** Infected dead cells density in lung tissues. Mean ± SD, *n* = 3. ^###^*P* < 0.001: significantly different from the Normal group; *P < 0.05, **P < 0.01: significantly different from the Infected group.

TUNEL of lung tissues in each group was detected by fluorescent staining to observe the apoptosis of H1N1-infected cells as the TUNEL is often used as a marker of apoptosis. As shown in Figure 9A, the nucleus was blue by labeling with DAPI, while positive apoptosis cells were green which was labeled with the FITC of the TUNEL assay kit. There were a large number of TUNEL + cells in the lung tissues of the Infected group of mice. However, the HSSD or Oseltamivir-treated groups showed significantly lower numbers of TUNEL-positive cells than the Infected group. In Figure 9B, the infected dead cells density in lung tissues was measured in images, the number of infected dead cells density was increased in the Infected group (*P* < 0.001), while decreased after treatment with HSSD or Oseltamivir (*P* < 0.01, or *P* < 0.05). Original images of lung TUNEL of three repeats are shown in Supplementary Datasheet 11.

### Effects of Huoxiang Suling Shuanghua Decoction on levels of immune globulin/cytokine/chemokine in H1N1-infected mice

**FIGURE 10:**
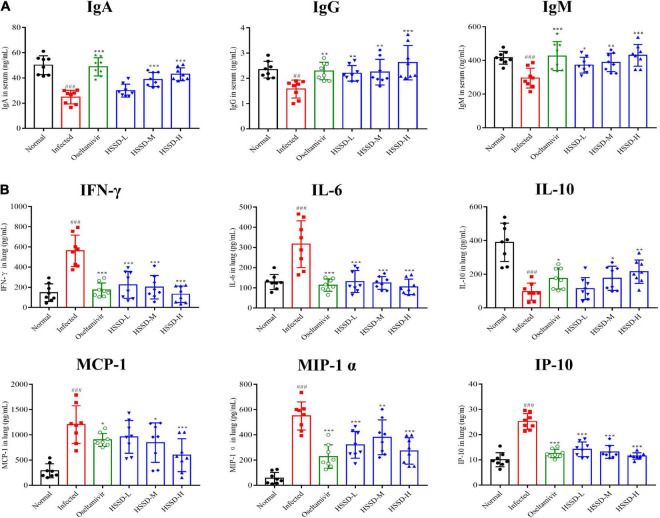
Effects of HSSD on levels of immune globulin/cytokine/chemokine in H1N1-infected mice. **(A)** Levels of IgA, IgG, and IgM in serum were detected by ELISA kits. **(B)** Levels of IFN-γ, IL-6, IL-10, MCP-1, MIP-1α, and IP-10 in lung homogenates were determined using ELISA kits. Mean ± SD, *n* = 8. ^##^*P* < 0.01, ^###^*P* < 0.001: significantly differently from the Normal group; *P < 0.05, **P < 0.01, ***P < 0.001: significantly differently from the Infected group.

Influenza virus infection occurred usually leading to inflammatory reactions to the imbalances of immune globulin, cytokines, and chemokines. As shown in Figure 10A, immune globulin including IgA, IgG, and IgM in the serum of the Infected group mice was significantly decreased (*P* < 0.001, or *P* < 0.01), while HSSD could increase this abnormal decline (*P* < 0.001, *P* < 0.01, or *P* < 0.05). Besides, Figure 10B shows that the treatment with HSSD could significantly inhibit the production of IFN-γ, IL-6, MCP-1, MIP-1α, and IP-10 (*P* < 0.001, *P* < 0.05, or *P* < 0.01), while increasing the production of IL-10 (*P* < 0.05 or *P* < 0.01). In addition, results revealed that GRA#, GRA%, and PLT were increased, while the LYM#, LYM%, WBC, and HGB were decreased in plasma—changes induced by H1N1 infection (*P* < 0.001). Notably, HSSD in different doses reverted the routine blood parameters markedly by reducing the GRA#, GRA%, and PLT (*P* < 0.001), and increasing the LYM#, LYM%, WBC, and HGB (*P* < 0.001, or *P* < 0.05). Effects of HSSD on routine blood parameters in H1N1-infected mice are shown in Supplementary Datasheet 12.

### Huoxiang Suling Shuanghua Decoction inhibited the expressions of TLR4/NF-κB p65 and HIF-1α/IL17 pathways in the lung tissues of H1N1-infected mice

**FIGURE 11:**
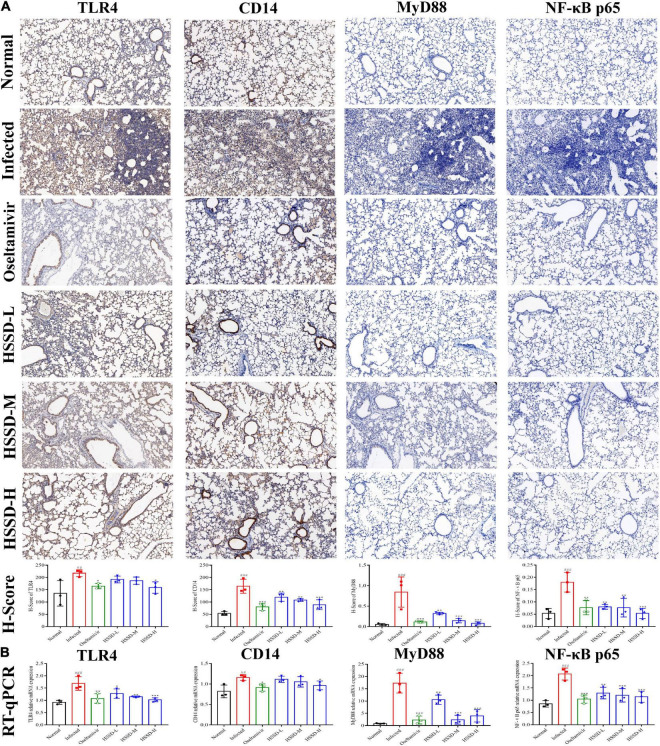
Expressions of TLR4/NF-κB p65 pathway in the lung tissues. **(A)** Protein expressions of TLR4, CD14, MyD88, and NF-κB p65 in the lung tissues of H1N1-infected mice and its H-score calculation (immunohistochemistry, ×100). **(B)** mRNA expression levels of TLR4, CD14, MyD88, and NF-κB p65 in the lung tissues of H1N1-infected mice. Mean ± SD, *n* = 3. ^##^*P* < 0.01, ^###^*P* < 0.001: significantly differently from the Normal group; *P < 0.05, **P < 0.01, ***P < 0.001: significantly differently from the Infected group.

**FIGURE 12:**
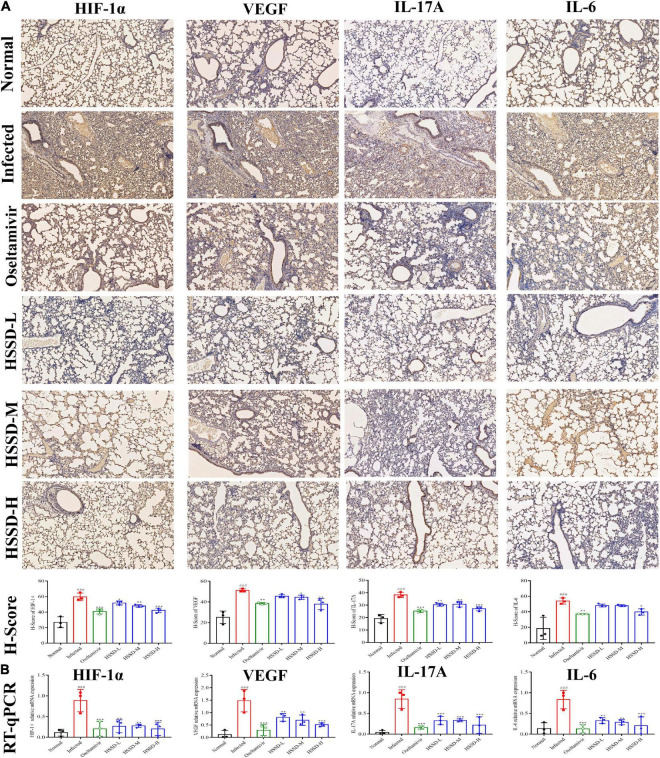
Expressions of HIF-1α/ IL17 pathway in the lung tissues. **(A)** Protein expressions of IL-17A, IL-6, HIF-1α, and VEGF in the lung tissues of H1N1 infected mice and its H-score calculation (immunohistochemistry, ×100). **(B)** mRNA expressions levels of IL-17A, IL-6, HIF-1α, and VEGF in the lung tissues of H1N1 infected mice. Mean ± SD, *n* = 3. ^###^*P* < 0.001: significantly differently from the Normal group; *P < 0.05, **P < 0.01, and ***P < 0.001: significantly differently from the Infected group.

To better understand the therapeutic mechanism of HSSD in H1N1-infected mice, we measured the expressions of TLR4/NF-κB p65 and HIF-1α/IL17 pathways according to the prediction of network pharmacology results in lung tissues on the day 5 post-infection by using RT-qPCR and IHC staining. In Figures 11, 12, both mRNA expressions and IHC protein expressions showed that H1N1 infection resulted in a significant increase in the expressions of TLR4, CD14, MyD88, NF-κB p65, HIF-1α, VEGF, IL-17A, and IL-6, (*P* < 0.01, or *P* < 0.001). After treatment with HSSD or Oseltamivir could downregulate their expressions (*P* < 0.05, *P* < 0.001, or *P* < 0.01). Original IHC images of three repeats are shown in Supplementary Datasheets 13, 14.

## Discussion

Traditional Chinese medicine formula is widely used in treating influenza in China. In our study, oral intake TCM formula HSSD, deriving from the clinical prescriptions HZP and Er Xiangshan Powder, showed considerable efficacy in the treatment of influenza. According to previous research, after being lethally infected with the influenza virus, the survival rate of mice, the change in body weight, and the state of the mice are the most intuitive indicators to assess antiviral efficacy (Song et al., 2016). The survival rate results showed that HSSD significantly improved the survival rate of mice infected with a lethal dose of H1N1 and prolonged the mice’s lifespan. Furthermore, HSSD inhibits the decreased body weight of infected mice, improving the statement of shivering, squinting, slow movement, etc. The survival rate test in an H1N1-infected mice model suggests that HSSD may be used as an alternative therapy for influenza. After viral transcription and replication, HA and NP playing the important role in the production, replication, proliferation, and release of the virus from host cells, which directly suggests the influenza virus loading in the host (Ma et al., 2018). After the virus infects the host, the corresponding immune status changes. Immunoglobulin is an effector molecule of humoral immunity, among which serotype of IgA can enhance the cytotoxic effect mediated by antibody-dependent cells, thereby inhibiting the adhesion of pathogenic bodies on the mucosa, phagocytosis, and lysis of bacteria and viruses, and then prevent them from entering the body (Gould et al., 2017). While IgM is the earliest antibody synthesized in body development and can be used for early diagnosis of infectious diseases (Justel et al., 2013). Besides, IgG is a humoral immune response antibody that can enhance macrophage phagocytosis of bacteria or kill target cells (Fujimoto et al., 2012). Therefore, we detected the levels of immunoglobulin (IgA, IgM, and IgG) contents to preliminarily explore the changes in immune status in influenza virus-infected mice and HSSD-treated mice. Moreover, routine blood parameters of GRA, LYM, WBC, PLT, and HGB represent the inflammatory response of the body after the infection of the influenza virus (Karimi et al., 2022). Our results demonstrated that HSSD can decrease the levels of HA and NP to inhibit the replication and proliferation of H1N1. In addition, HSSD reduces the lung pathological state of the H1N1-infected mice, inhibits the cell apoptosis in the lungs of the H1N1-infected mice, and regulates the abnormal responses of immunoglobulins (IgA, IgM, and IgG) and routine blood parameters, indicating the antiviral effects of HSSD.

Next, to explore the active ingredients of HSSD in treating influenza, the major chemical components of HSSD were identified by the UPLC/Q-TOF MS method. As a result, 87 kinds of compounds were identified by using the HSSD solution directly, and 20 kinds of compounds were identified in the absorbed blood. There were 54 kinds of compounds that had higher than 1,000,000 intensity of HSSD. While there were 12 kinds of compounds that had higher than 100,000 in the intensity of HSSD absorbing into the blood, and the flavonoids including luteolin-7-O-diglucuronide, apigenin-7-glucuronide, hesperidin, nobiletin, 5,6,7,3′,4′,5′-hexamethoxyflavone, and 3,5,6,7,8,3′,4′-heptamethoxyflavone were the most abundant components that absorbed into the serum after treating HSSD. Furthermore, the main components of HSSD in the HPLC profile were identified including chlorogenic acid, caffeic acid, scutellarin, and rosmarinic acid. Based on these identified compounds of HSSD and related databases, the network of “herb-compounds-targets” of HSSD was constructed and the critical compounds were ranked according to the degree value of the network. Finally, the 16 active ingredients of HSSD, including quercetin, beta-sitosterol, ursolic acid, kaempferol, hyperoside, wogonin, luteolin, acacetin, chlorogenic acid, caffeic acid, scutellarin, rosmarinic acid, hesperidin, naringin, paeoniflorin, and nobiletin, were screened out by combining with the experimental identified compounds results and the network analysis critical compounds results. Focusing on these critical compounds above, these 16 active compounds may be the potential material basis for the anti-influenza virus effect of HSSD. Furthermore, previous research has demonstrated the anti-influenza virus effect on most of these active compounds as followings. Quercetin shows inhibitory activity in the early stage of influenza infection (Wu et al., 2016). Beta-sitosterol is reported for its protection against influenza through NF-κB and p38 mitogen-activated protein kinase (MAPK) signaling in influenza A virus (IAV) infected cells to suppress inflammatory response (Zhou et al., 2020). Ursolic acid attenuates IAV-triggered inflammatory responses by regulating the miR-34c-5p/TLR5 axis (Wei et al., 2022). Kaempferol exhibits a protective effect on virus-induced inflammation *via* suppression of TLR4/MyD88-mediated NF-κB and MAPK pathways (Zhang et al., 2017). Hyperoside is reported as a universal drug against various influenza strains by perfect binding with conserved residues of influenza virus NP (Ahmad et al., 2016). Wogonin possesses a potent anti-influenza activity mediated by the regulation of AMPK activation (Seong et al., 2018). Luteolin suppresses coat protein I complex expression, which is related to influenza virus entry and endocytic pathway (Yan et al., 2019). Acacetin exhibits active effect on neuraminidase (NA) (Xu et al., 2020). Chlorogenic acid acts as a neuraminidase blocker to inhibit the influenza A virus both in cellular and animal models (Ding et al., 2017). Caffeic acid exhibits anti-virus in the early stages of infection (Utsunomiya et al., 2014). Scutellarin can be used in the treatment of influenza through anti-inflammatory (Fang et al., 2019). Rosmarinic acid has been reported to exert inhibitory activity against NA (Mahalapbutr et al., 2020). Hesperidin alleviates H1N1-induced impairment of pulmonary function by inhibiting cytokine production through MAPK signaling pathways (Ding et al., 2018). Paeoniflorin inhibits the expression of αvβ3, TGF-β1, Smad2, NF-κB, and p38MAPK in the lung tissues for anti-influenza virus (Yu et al., 2021). These above-reported pieces of evidence of pharmacodynamic action of HSSD in treating influenza are mainly related to the inhibition of protein target NA required for early viral infection or the inhibition of inflammatory response caused by viral infection including TLR4/MyD88, NF-κB, and MAPKs pathways. Briefly, these pieces of evidence between small molecule compounds and signaling pathways in previous research suggest reference signaling pathways of HSSD in treating influenza in our following mice experiments.

Network pharmacology was performed to investigate the potential key targets and critical KEGG signaling pathways of HSSD in treating influenza. As a result, the critical top 10 KEGG signaling pathways are mainly revealed in the IL-17 signaling pathway, TNF signaling pathway, PI3K-Akt signaling pathway, Toll-like receptor signaling pathway, FoxO signaling pathway, etc. While the TLR4, CD14, MyD88, NF-κB p65 in TLR4/NF-κB p65 signaling pathway, HIF1 α, VEGF, IL17A, and IL6 in HIF-1α/IL17 signaling pathway, as well as the TNF, IL1B, CCL2, CXCL8, IL10, STAT1, IFNG, and CXCL10 in the critical hub genes, were obtained as the key targets of HSSD in treating H1N1. Molecular docking results between ingredient ligands (quercetin, beta-sitosterol, ursolic acid, kaempferol, hyperoside, wogonin, luteolin, acacetin, chlorogenic acid, caffeic acid, scutellarin, rosmarinic acid, hesperidin, naringin, paeoniflorin, and nobiletin) and protein targets (TLR4, CD14, MyD88, NF-κB p65, HIF1 α, VEGF, IL17A, IL6, TNF, IL1B, CCL2, CXCL8, IL10, STAT1, IFNG, and CXCL10) in our study shown stable binding modes. Influenza A is a single-stranded (ss) RNA, which can infect monocytes and macrophages causing the production of mediators of inflammatory and chemotactic cytokine and leading to acute lung injury (Cline et al., 2017). CD14 can be found either on the surface of macrophages or in soluble form in the serum, and CD14 plays a pro-inflammatory role leading to increased inflammation during influenza infection (Palipane et al., 2019). Research also reported that blocking TLR4 or CD14 in mice can protect against influenza-induced acute lung injury (Shirey et al., 2013). Besides, previous studies have reported that CD14, together with Toll-like receptors (TLRs), are the essential coreceptors recognizing viral proteins and nucleic acid of the influenza A virus in the sensing recognition system (Pauligk et al., 2004, Shi et al., 2020). In addition, blocking of CD14 on human monocytes by specific antibody or use of CD14-deficient murine macrophages abolished influenza A virus-induced cytokine production suggesting CD14 is required for influenza-induced cytokine production during infection of mouse macrophages (Dessing et al., 2007). Furthermore, if the virus infection persists, the NF-κB p65 pathway is activated and produces excessive proinflammatory cytokines, leading to acute inflammatory lung injury (Ma et al., 2021). Therefore, TLR4 or CD14 may bind to ssRNA, activating MyD88, and then the activation of MyD88 transmitting signals and activating NF-κB p65, which increases the production of proinflammatory cytokines of IFN-γ, IL-6, and chemokines of MCP-1, MIP-1α, and IP-10, while inhibiting the production of anti-inflammatory cytokine of IL-10 (Zhu et al., 2018; Ma et al., 2020). Besides, clinical research has shown that the levels of cytokines IL-6 and IL-17 are related to the disease severity of H1N1 infection patients, especially the IL-6 may be a biomarker for predicting fatal outcomes of H1N1 infection (Alagarasu et al., 2021). Hypoxia-inducible factor 1α (HIF-1α) is an important transcription factor that regulates many downstream target genes (Huang et al., 2022). Once HIF-1α is over expression, its downstream target of VEGF is activated and overexpressed, and then promoting vascular endothelial cell division, inducing angiogenesis, and increasing vascular permeability, finally resulting in hyperosmolar pulmonary edema and inflammation pulmonary capillary endothelial cell damage (Li et al., 2020). Besides, the increased HIF-1α expression commonly enhances the production of IL-6 and IL-17 in a disease state (Talreja et al., 2019). Furthermore, researchers demonstrated that HIF-1α may function in conjunction with TLR4-stimulated innate immune responses to drive inflammation, suggesting the important role of TLR4/NF-κB p65 pathway and HIF-1α/IL-17 pathway in the occurrence of inflammation after the H1N1 infection (Hu et al., 2014). Thus, the TLR4, CD14, MyD88, and NF-κB p65 in TLR4/NF-κB p65 signaling pathway, HIF1 α, VEGF, IL17A, and IL6 in HIF-1α/IL17 signaling pathway may be the targets responsible for the anti-inflammatory function of HSSD in treating influenza.

Overall, our results suggest that HSSD exerts pharmacological activity against influenza through multiple targets involved in TLR4/NF-κB p65 signaling pathway and the HIF-1α/IL17 signaling pathway. Regardless, this study still has some limitations. There are multiple targets and multiple signaling pathways of HSSD in treating influenza that was screened out by network pharmacology; we tested the critical two signaling pathways of TLR4/NF-κB p65 signaling pathway and HIF-1α/IL17 signaling pathway, which may fail in confirming the other actual effects of candidate targets and other signaling pathways. Therefore, further experimental validation is required in future studies from other respects.

## Conclusion

In our study, an integrative pharmacology strategy combined with experimental validation was used to explore the effects and mechanisms of HSSD in treating influenza. First, the therapeutic effects of HSSD were confirmed through the animal survival rate experiment by the lethal dose of H1N1 infection. Then, 87 kinds of compounds were identified using HSSD solution directly by UPLC/Q-TOF MS method, and 20 kinds of absorbing blood compounds were identified by using the serum sample treating the HSSD orally in rats. Second, 16 kinds of active compounds including quercetin, beta-sitosterol, ursolic acid, kaempferol, hyperoside, wogonin, luteolin, acacetin, chlorogenic acid, caffeic acid, scutellarin, rosmarinic acid, hesperidin, naringin, paeoniflorin, and nobiletin were screened out as the critical compounds of HSSD in treating influenza according to the identification results and the network analysis results. Third, through network pharmacology and molecular docking analysis, the TLR4/NF-κB p65 signaling pathway and HIF-1α/IL17 signaling pathway were found to be highly related to the anti-inflammatory action of HSSD in treating influenza. Next, eight key targets were identified, including TLR4, CD14, MyD88, NF-κB p65, HIF1 α, VEGF, IL17A, and IL6, which were verified to be key targets for HSSD in the treatment of influenza. Finally, the H1N1 infection mice model demonstrated that HSSD can inhibit the accumulation of inflammation in influenza mice by decreasing the levels of HA and NP, reducing the cell apoptosis and pathological state in the lung, regulating the levels of routine blood parameters, IFN-γ, IL-6, IL-10, MCP-1, MIP-1α, and IP-10, as well as attenuating the mRNA and protein expressions of key targets including TLR4, CD14, MyD88, NF-κB p65, HIF1 α, VEGF, IL17A, and IL6. In conclusion, our study suggested that TLR4, CD14, MyD88, NF-κB p65, HIF1 α, VEGF, IL17A, and IL6 in the TLR4/NF-κB p65 signaling pathway and HIF-1α/IL17 signaling pathway may be the key targets for the identified active compounds of HSSD to treat influenza by inhibiting inflammatory responses. Our study provides an efficient and powerful approach to a preliminary understanding of the mechanisms of the TCM formula. Further experimental validation of these predictions will be conducted to encourage a broader application of HSSD in clinical settings.

## Data availability statement

The raw UPLCQ-TOF MS DATA presented in the study are deposited in the FigShare repository, accession number https://doi.org/10.6084/m9.figshare.21300021.

## Ethics statement

All animal experiments were performed according to protocols approved by the Welfare and Ethical Inspection in the Beijing University of Chinese Medicine Animal Care Committee (No. BUCM-4-2021071601-3022).

## Author contributions

RT, JZ, LW, and WH conceived and designed the experiments. RT, XL, LT, and MW carried out the animal experiment. HH, XL, and YL conducted molecular experiments. RT and MW analyzed the data. RT wrote the manuscript. LW, WH, and KW contribution to the funding acquisition and administration. All authors contributed to the article and approved the submitted version.
