# Supplementary material for: Exploring the active ingredients and pharmacological mechanisms of the oral intake formula Huoxiang Suling Shuanghua Decoction on influenza virus type A based on network pharmacology and experimental exploration
Source: Front Microbiol. 2022 Nov 1;13:1040056. doi: 10.3389/fmicb.2022.1040056 (PMC9663660; doi:10.3389/fmicb.2022.1040056)
Supplement: Supplementary file 1 [file Data_Sheet_2.PDF]

Supplementary Data Sheet 2: Raw data of compounds identification of HSSD solution by UPLC/Q-TOF MS.

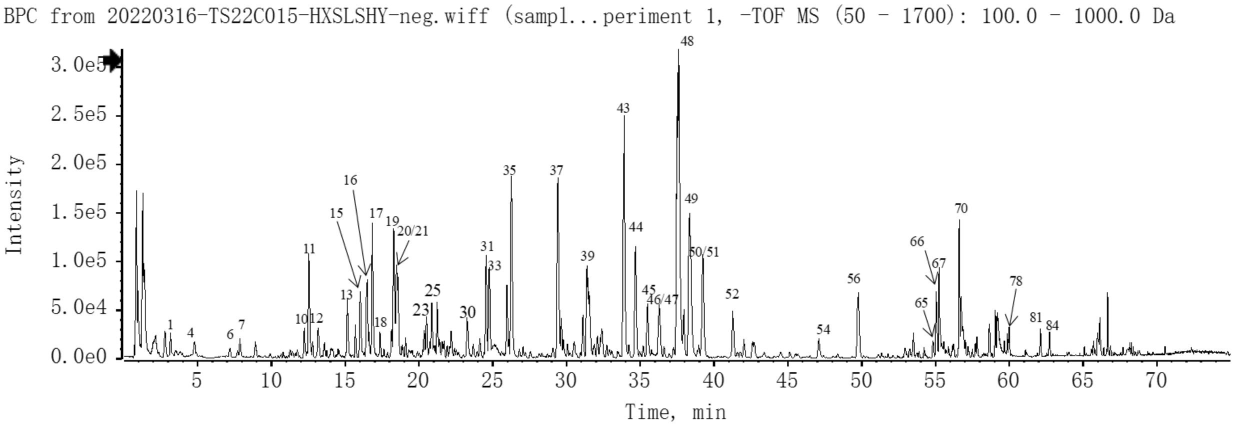

Figure S1 Negative ion mode of HSSD solution samples UPLC-HRMS base peak ion current diagram (BPC).

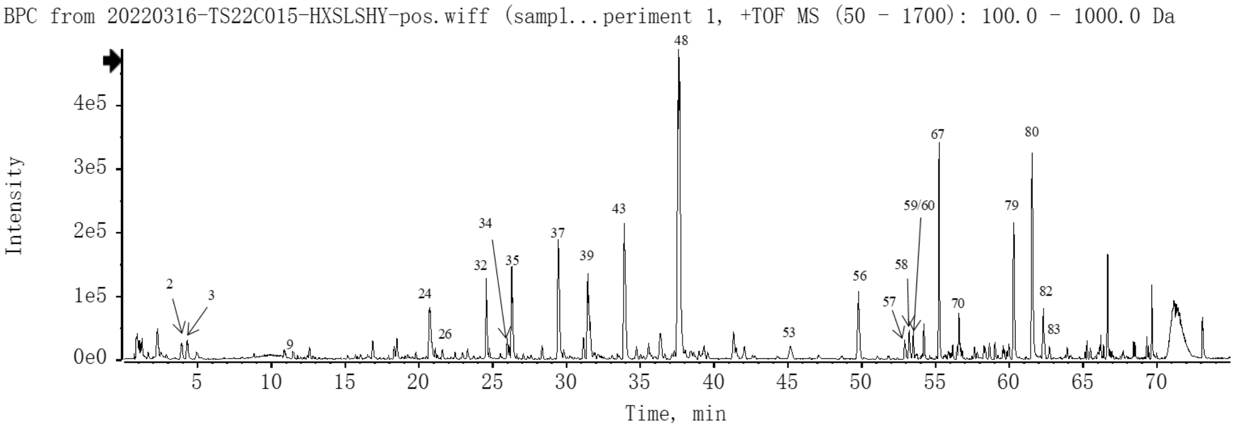

Figure S2 Positive ion mode of HSSD solution samples UPLC-HRMS base peak ion current

diagram (BPC).

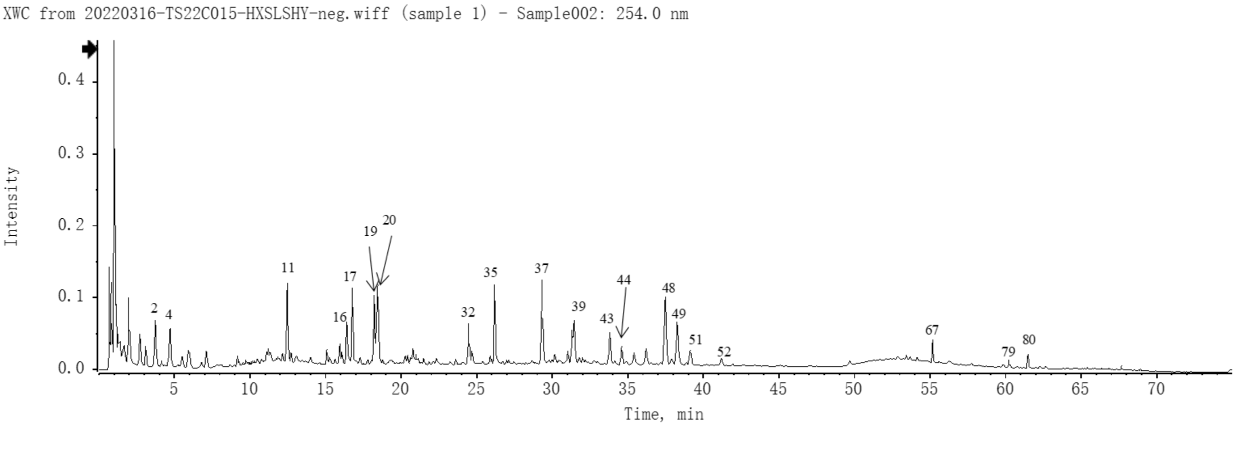

Figure S3 UV chromatogram (UV 254 nm) of UPLC of HSSD solution.

Figure S4 MS[1]and MS[2] spectrograms of 87 components of HSSD as followings:

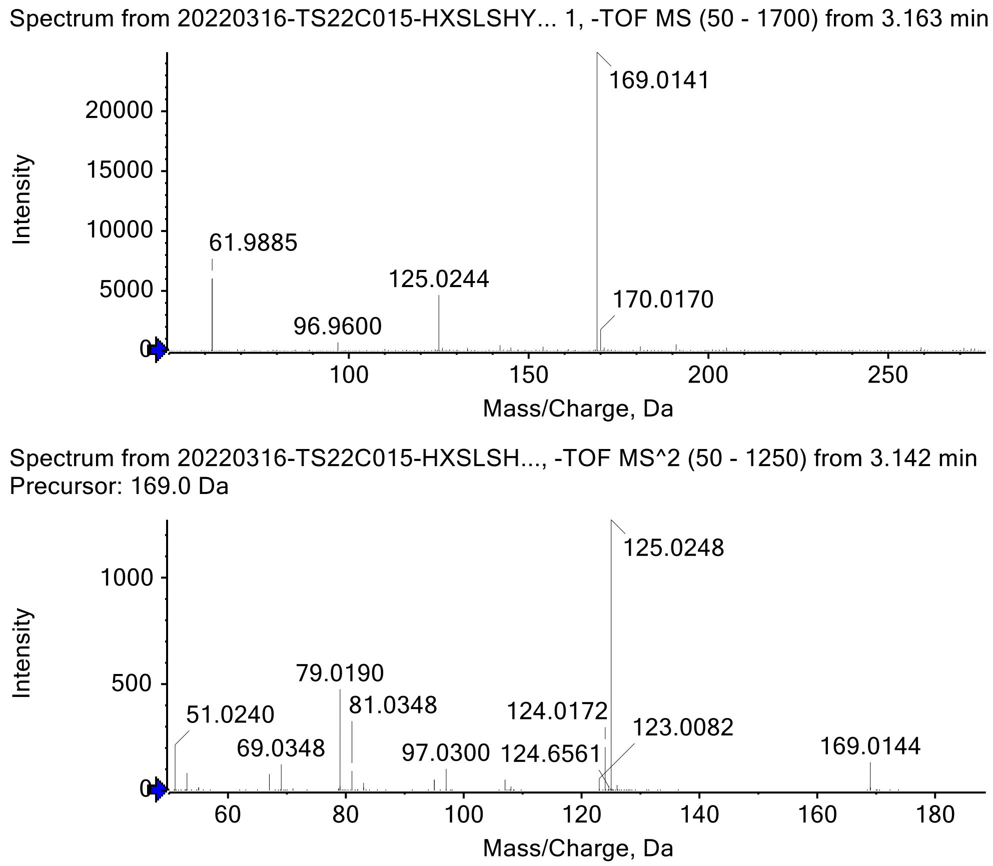

Figure S4-1: MS[1]and MS[2] spectrograms of component 1 in HSSD

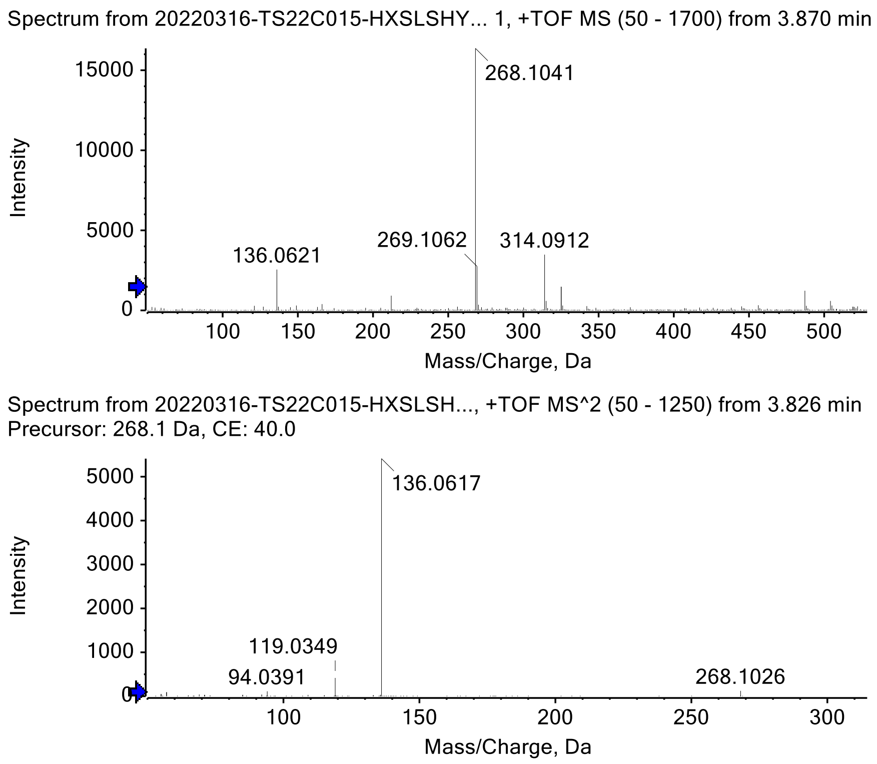

Figure S4-2: MS^{1}and MS^2 spectrograms of component 2 in HSSD

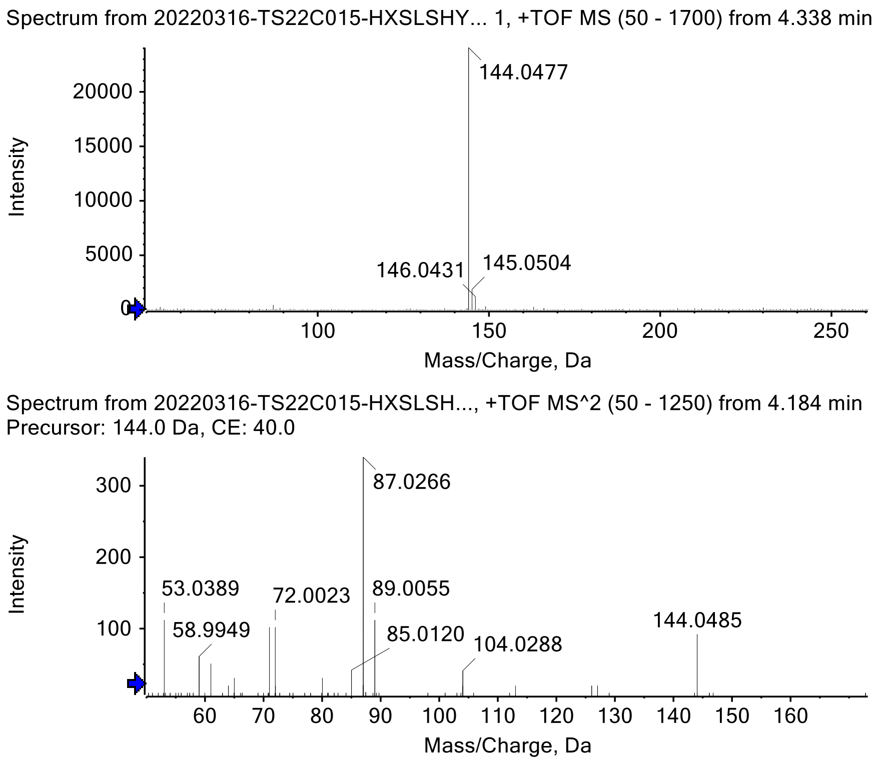

Figure S4-3: MS^{1}and MS^2 spectrograms of component 3 in HSSD

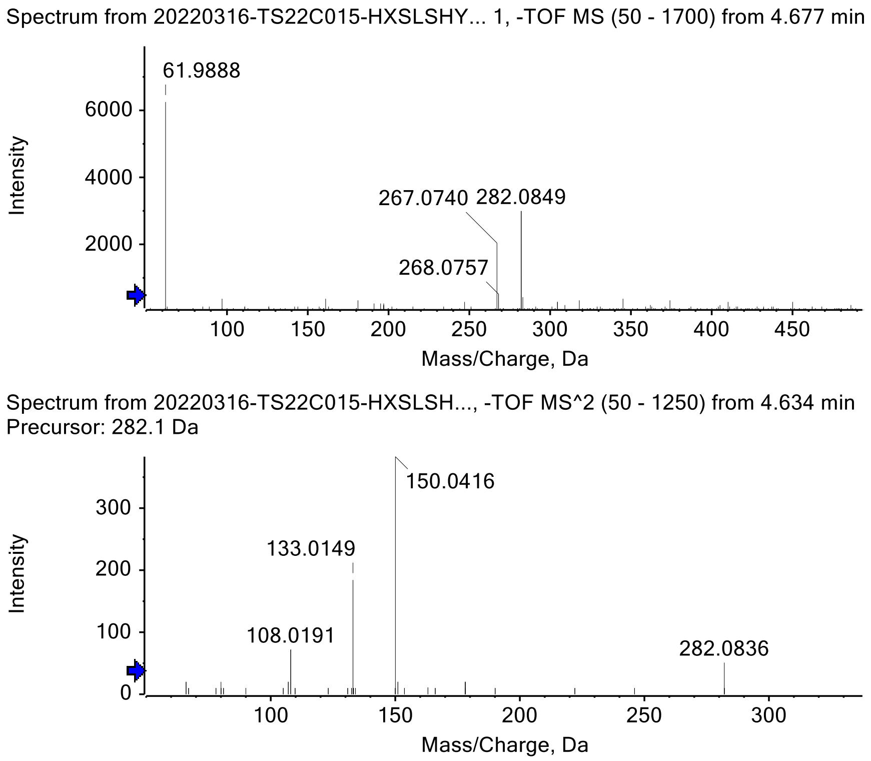

Figure S4-4: $MS^1$and MS^2 spectrograms of component 4 in HSSD

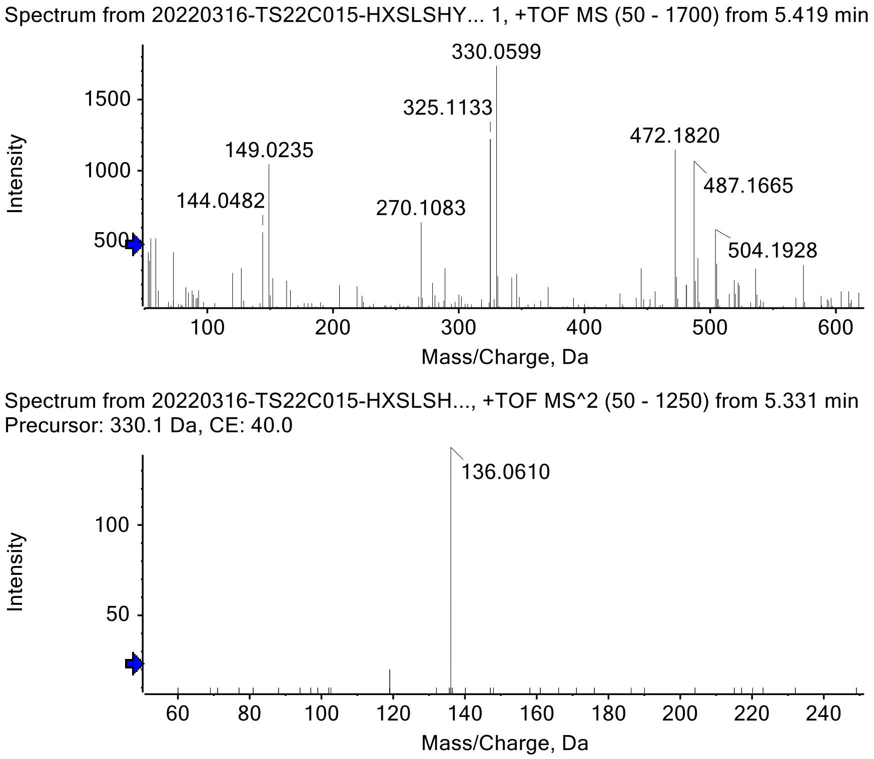

Figure S4-5: $MS^1$and MS^2 spectrograms of component 5 in HSSD

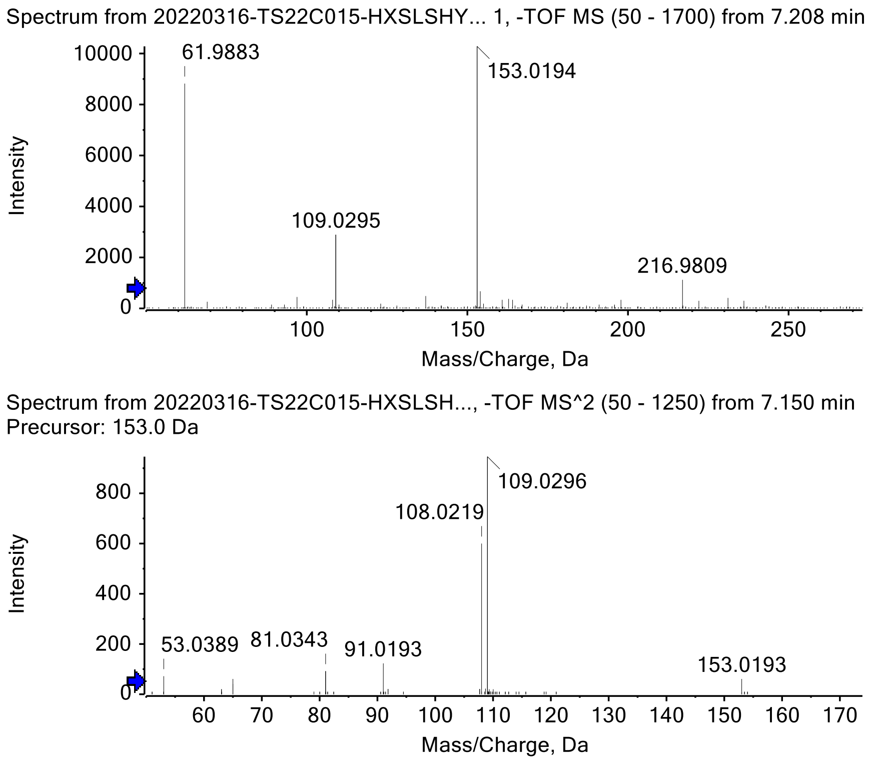

Figure S4-6: MS^{1}and MS^2 spectrograms of component 6 in HSSD

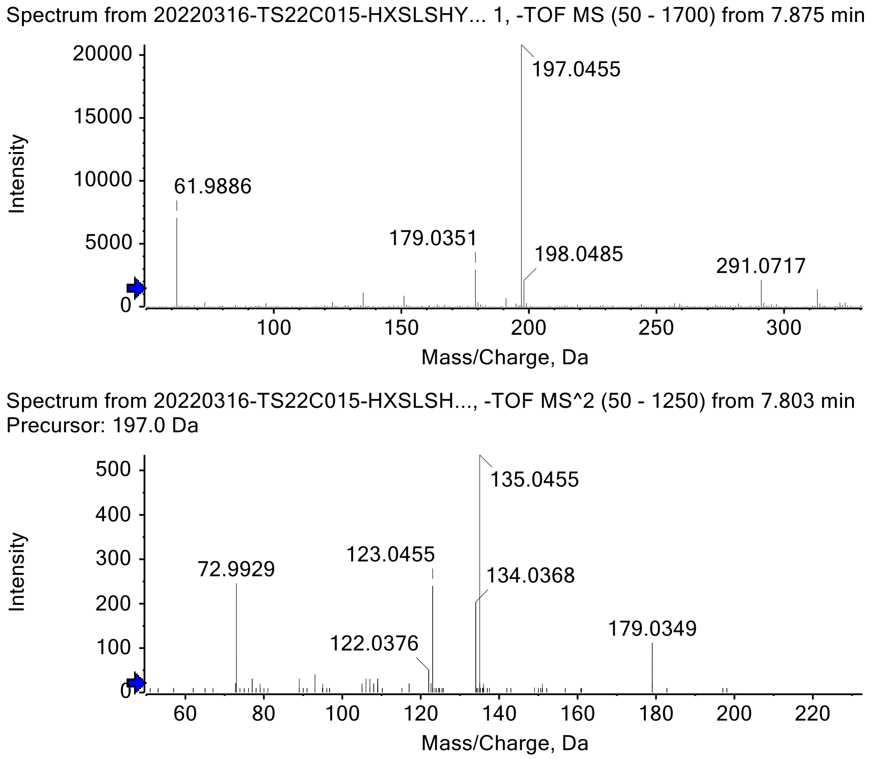

Figure S4-7: MS^{1}and MS^2 spectrograms of component 7 in HSSD

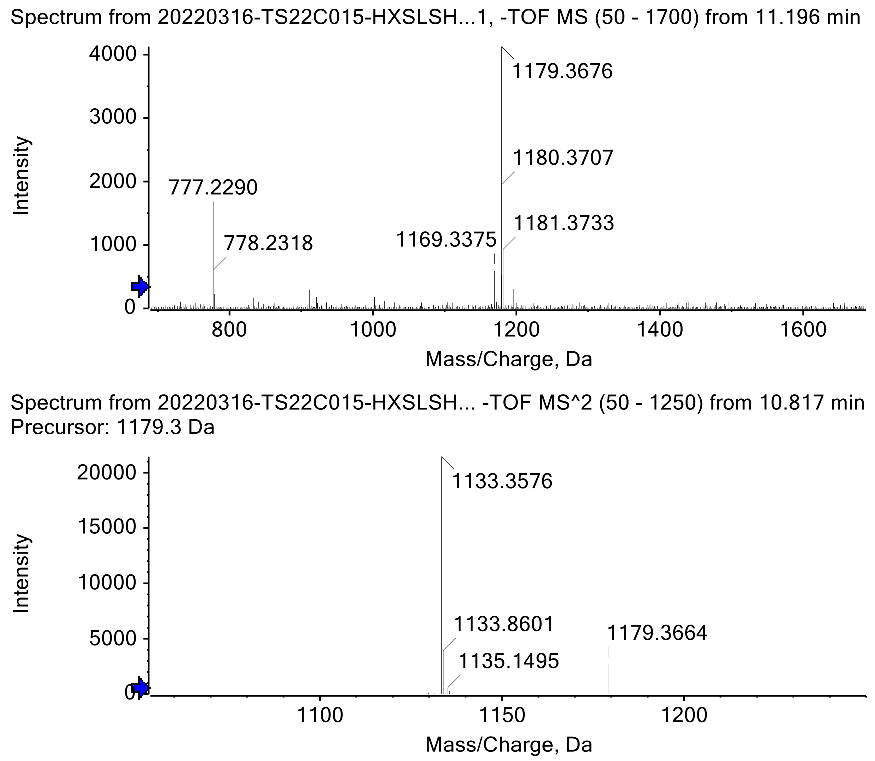

Figure S4-8: MS[1]and MS[2] spectrograms of component 8 in HSSD

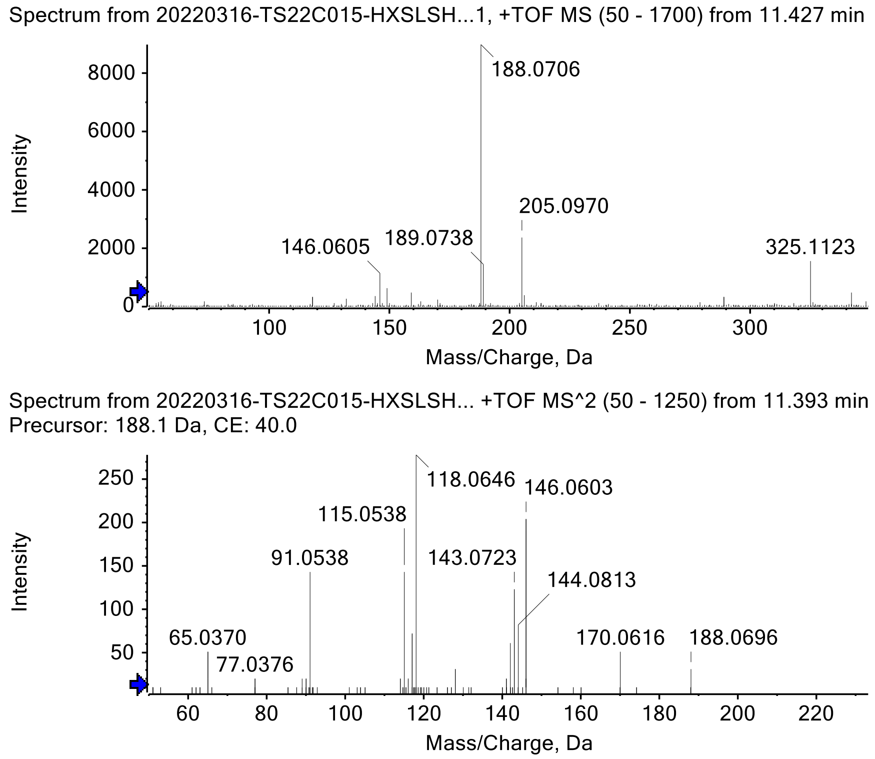

Figure S4-9: MS[1]and MS[2] spectrograms of component 9 in HSSD

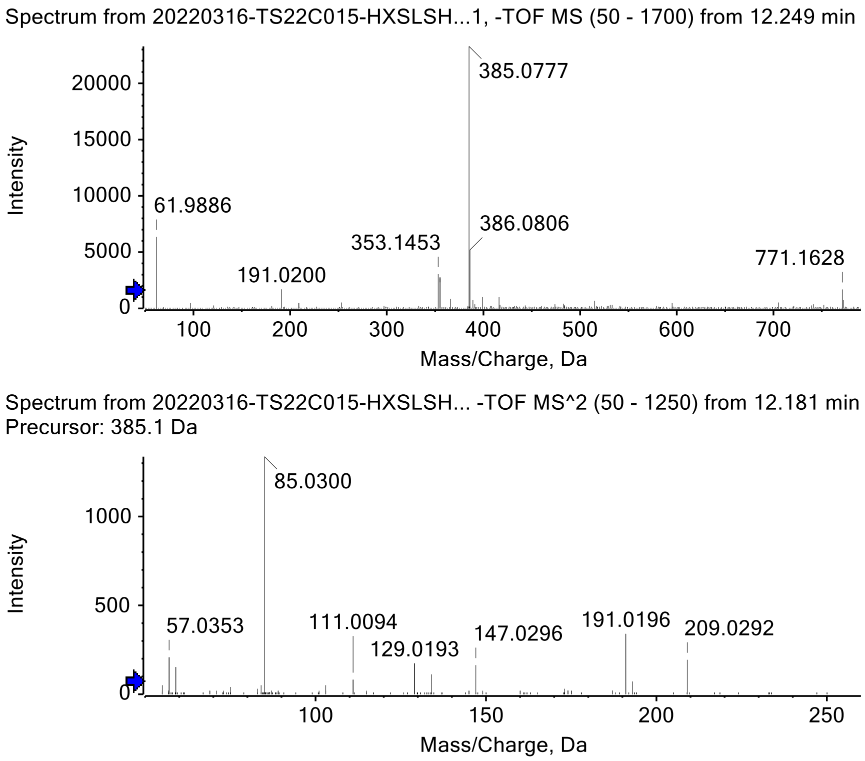

Figure S4-10: MS^{1}and MS^2 spectrograms of component 10 in HSSD

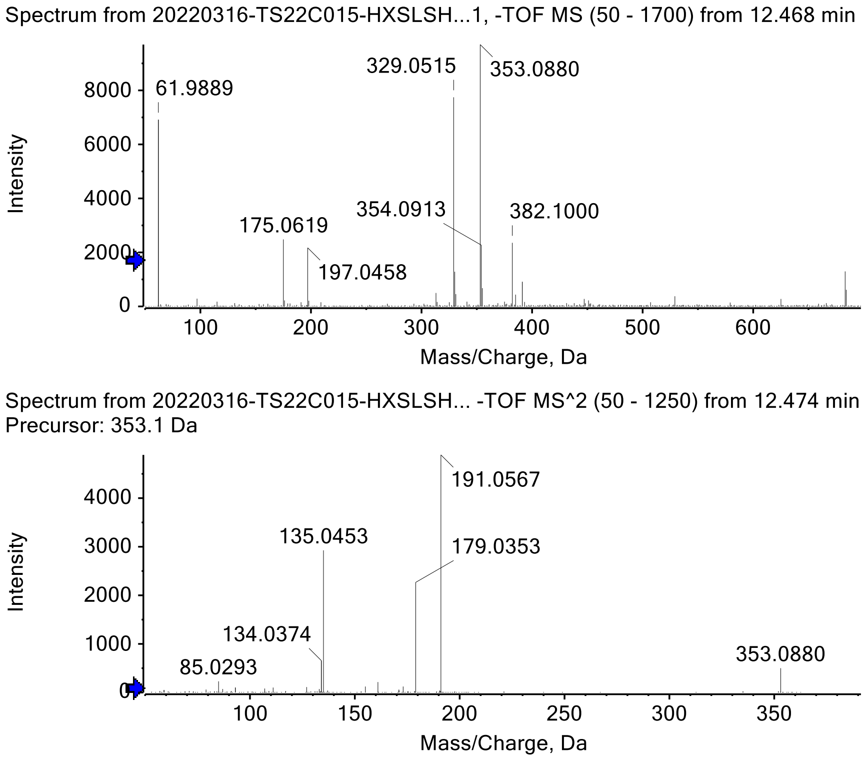

Figure S4-11: MS^{1}and MS^2 spectrograms of component 11 in HSSD

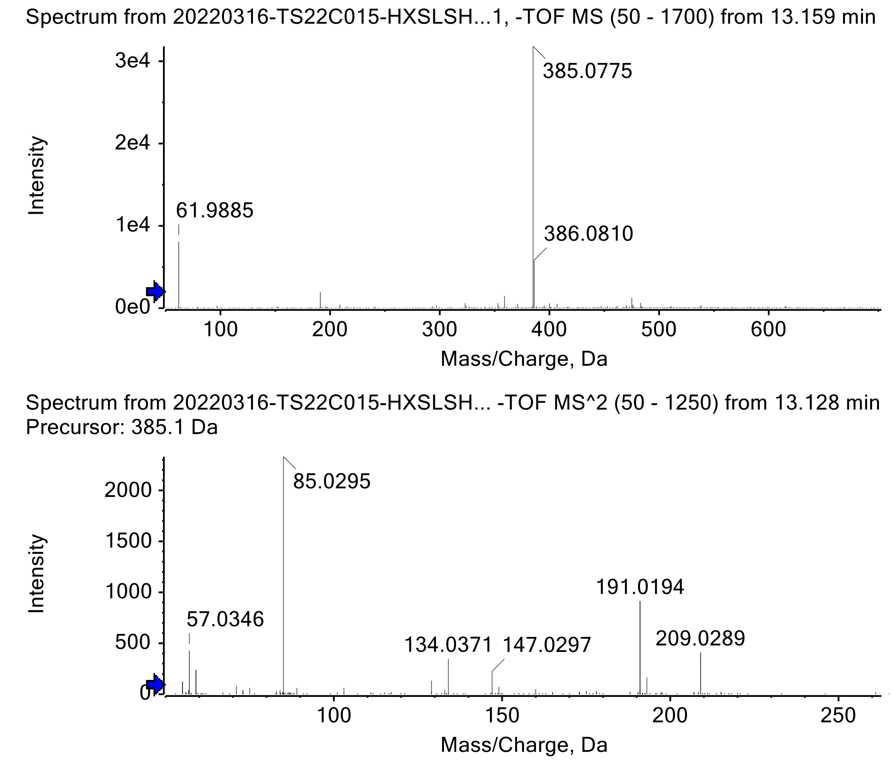

Figure S4-12: MS[1]and MS[2] spectrograms of component 12 in HSSD

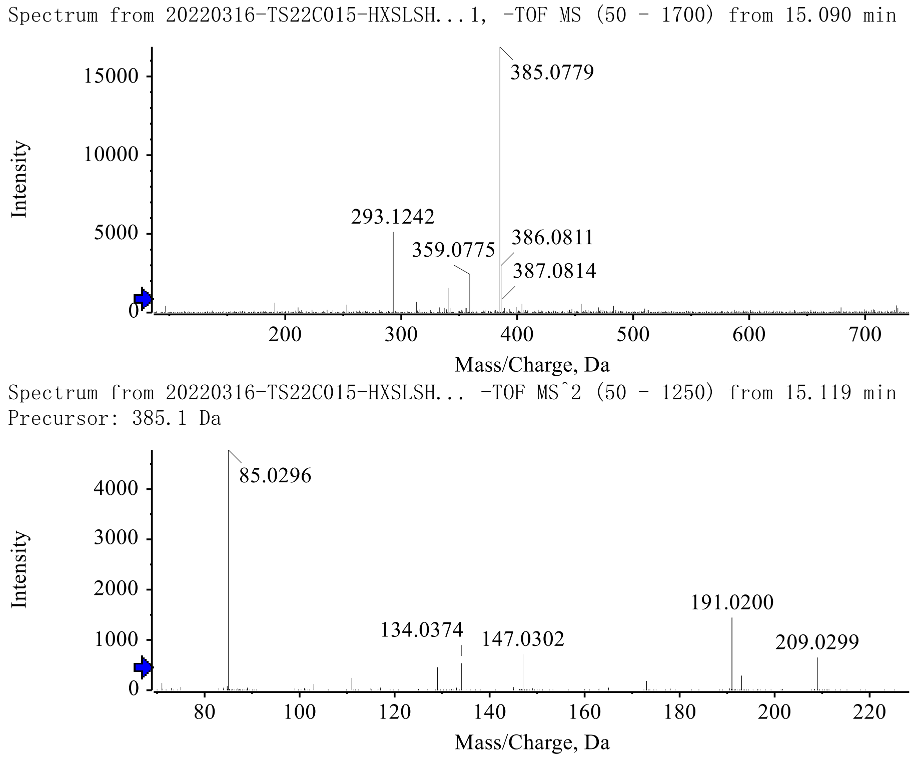

Figure S4-13: MS[1]and MS[2] spectrograms of component 13 in HSSD

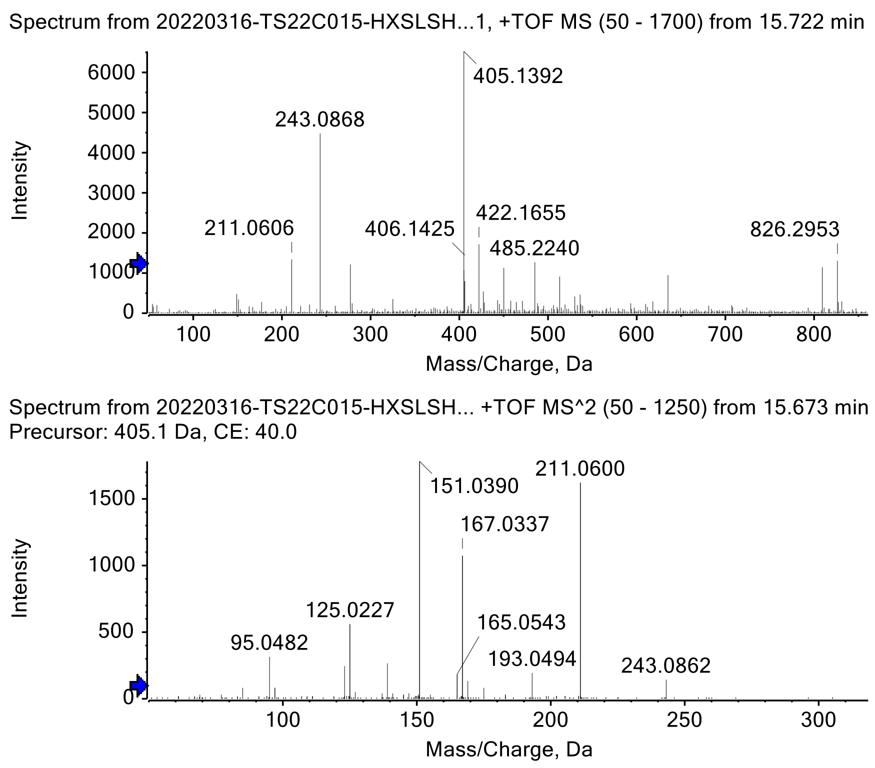

Figure S4-14: MS^{1}and MS^2 spectrograms of component 14 in HSSD

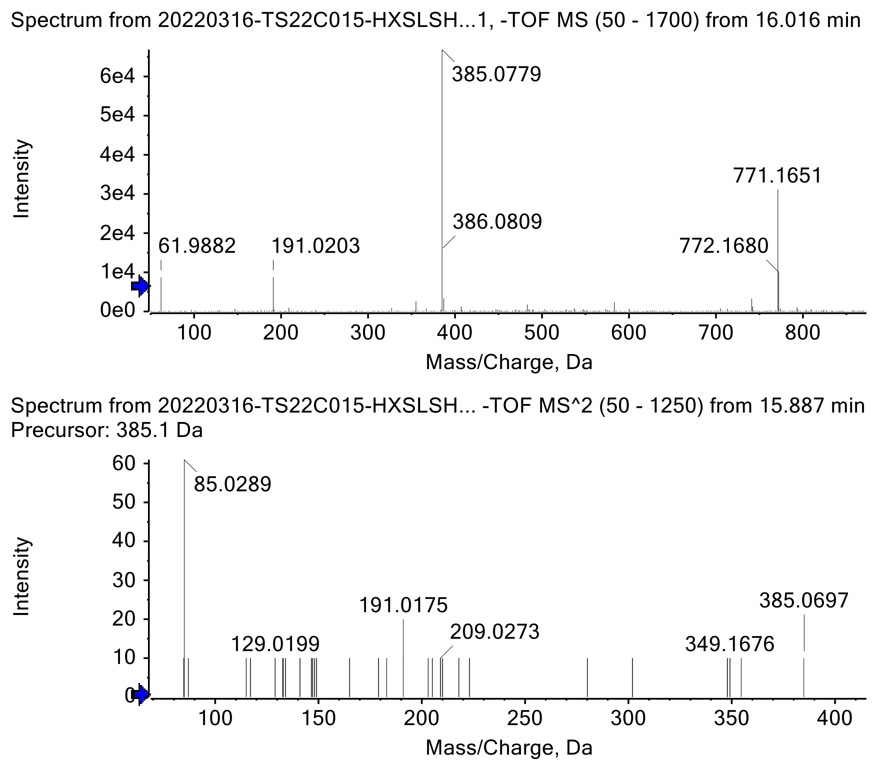

Figure S4-15: MS^{1}and MS^2 spectrograms of component 15 in HSSD

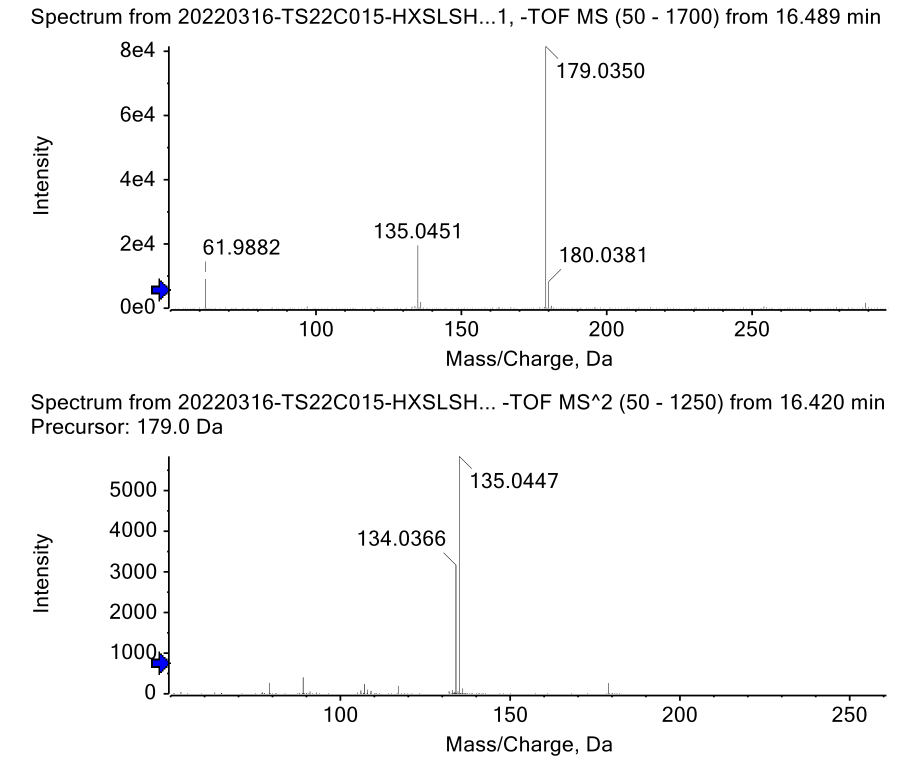

Figure S4-16: MS[1]and MS[2] spectrograms of component 16 in HSSD

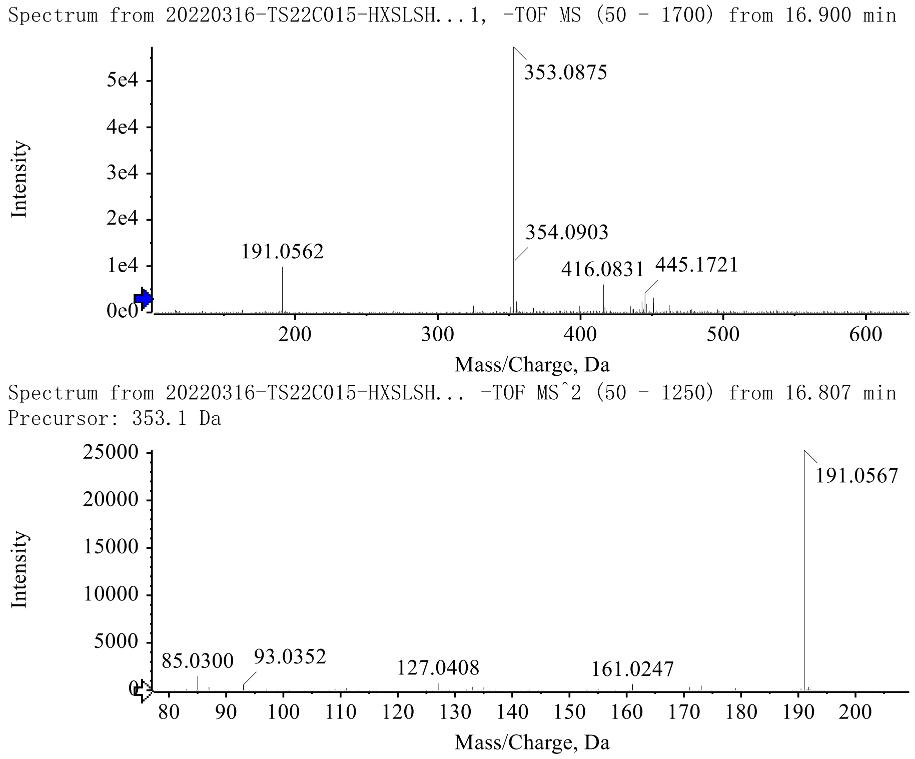

Figure S4-17: MS[1]and MS[2] spectrograms of component 17 in HSSD

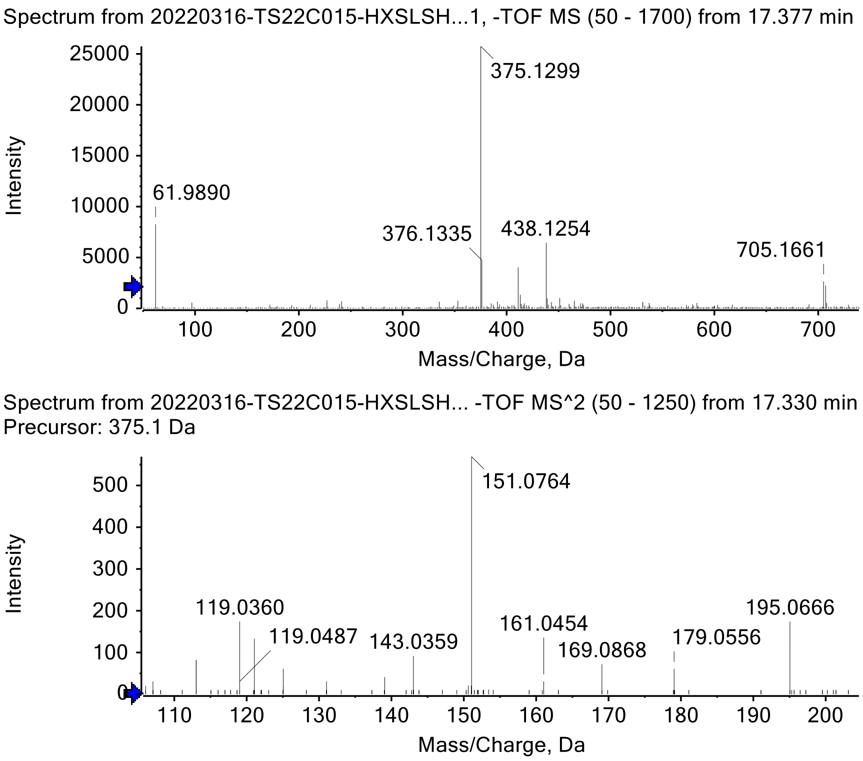

Figure S4-18: MS[1]and MS[2] spectrograms of component 18 in HSSD

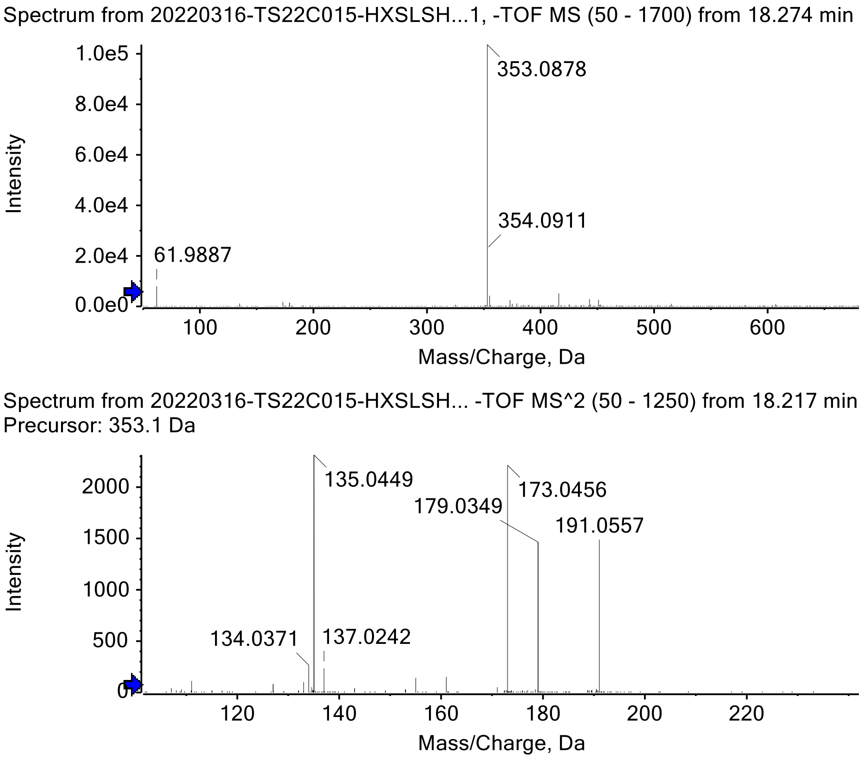

Figure S4-19: MS[1]and MS[2] spectrograms of component 19 in HSSD

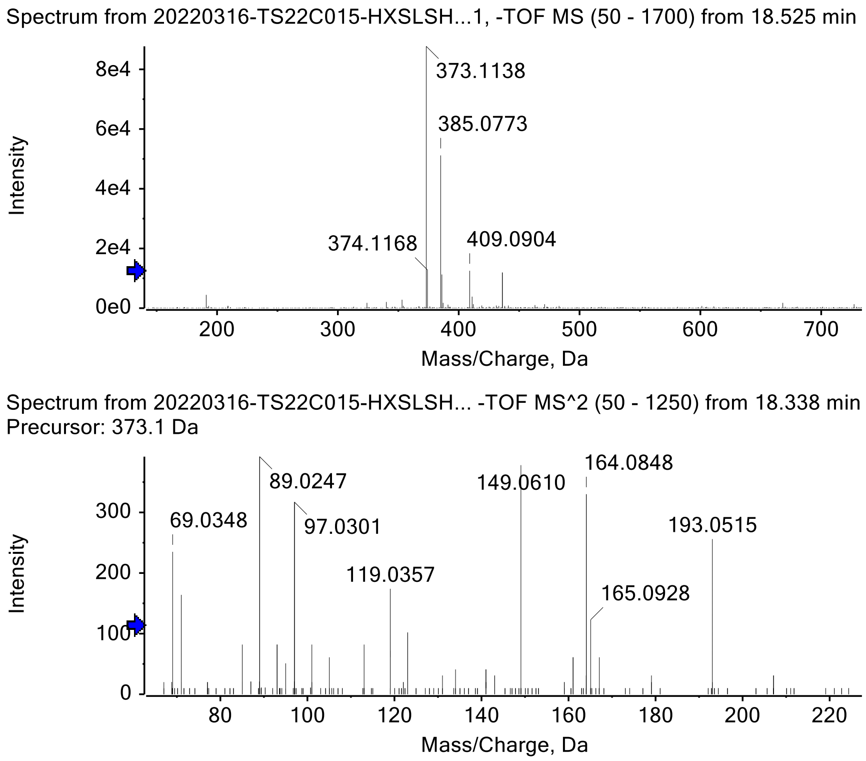

Figure S4-20: MS[1]and MS[2] spectrograms of component 20 in HSSD

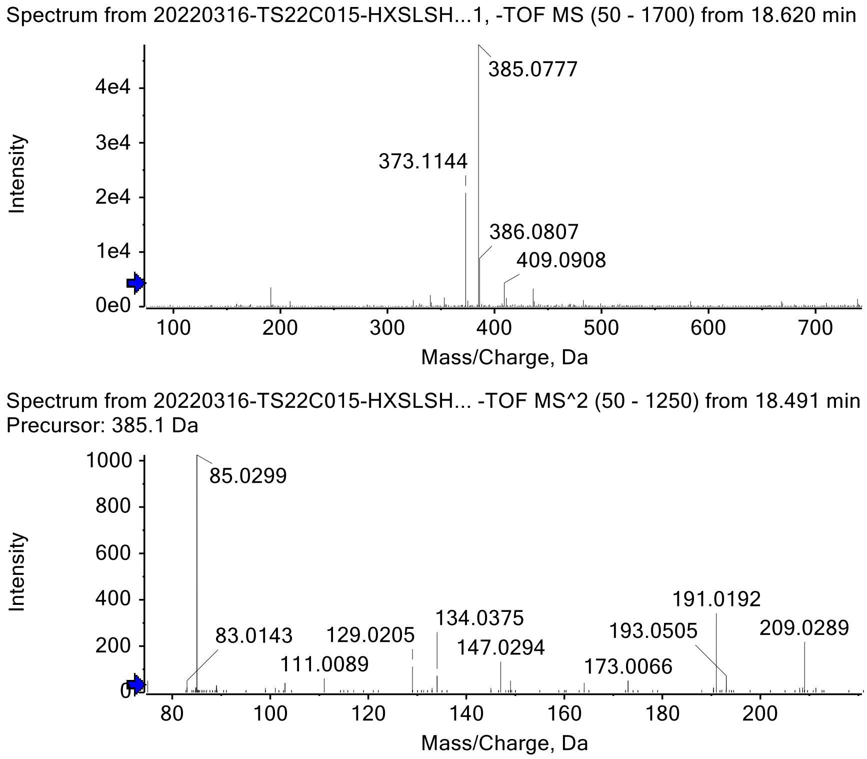

Figure S4-21: MS[1]and MS[2] spectrograms of component 21 in HSSD

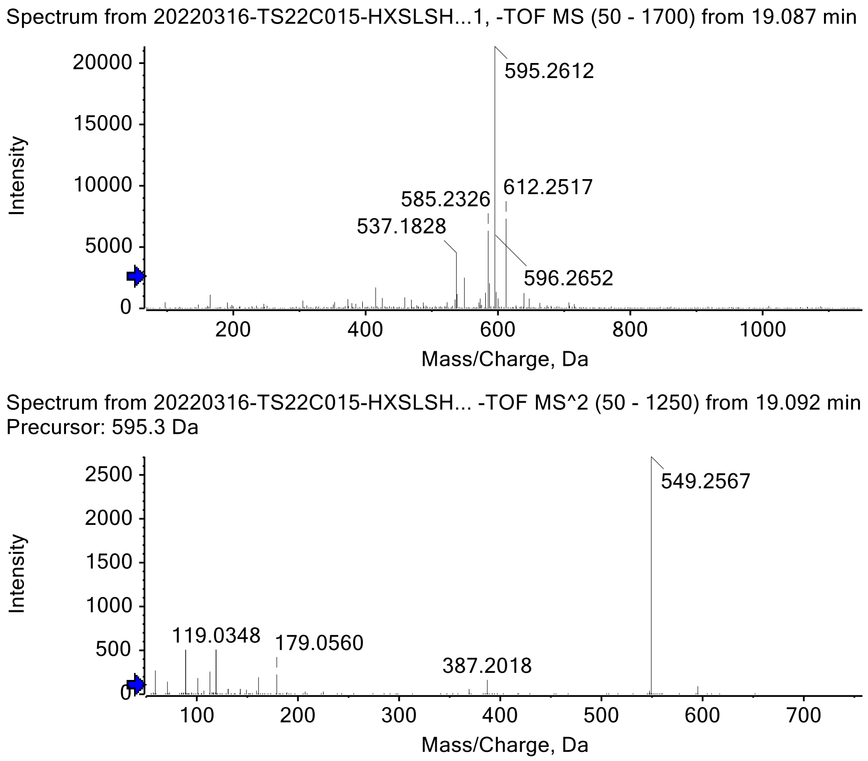

Figure S4-22: $MS^1$and MS^2 spectrograms of component 22 in HSSD

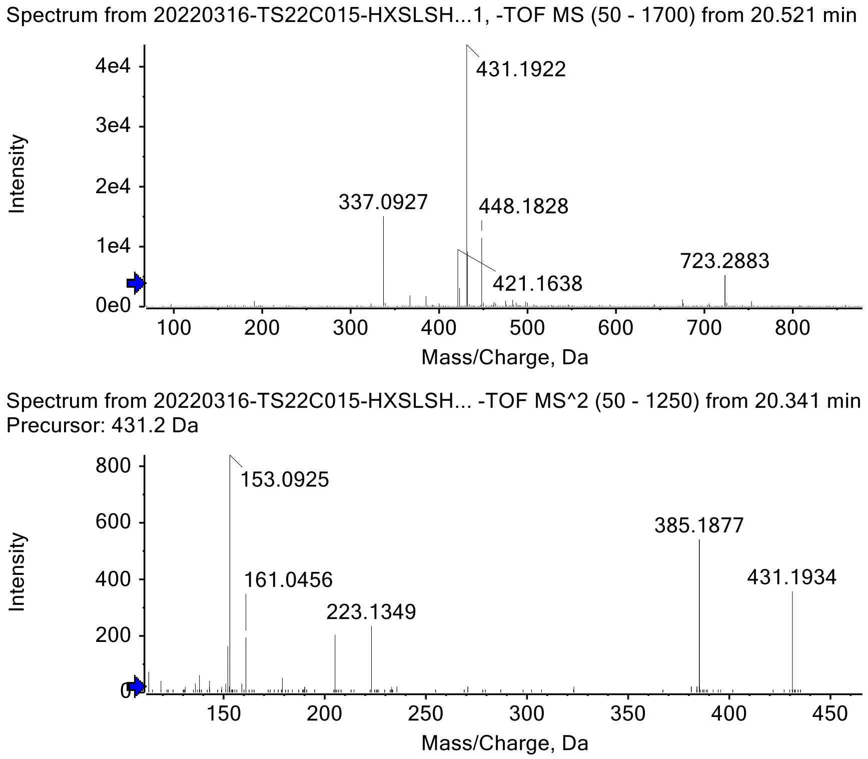

Figure S4-23: $MS^1$and MS^2 spectrograms of component 23 in HSSD

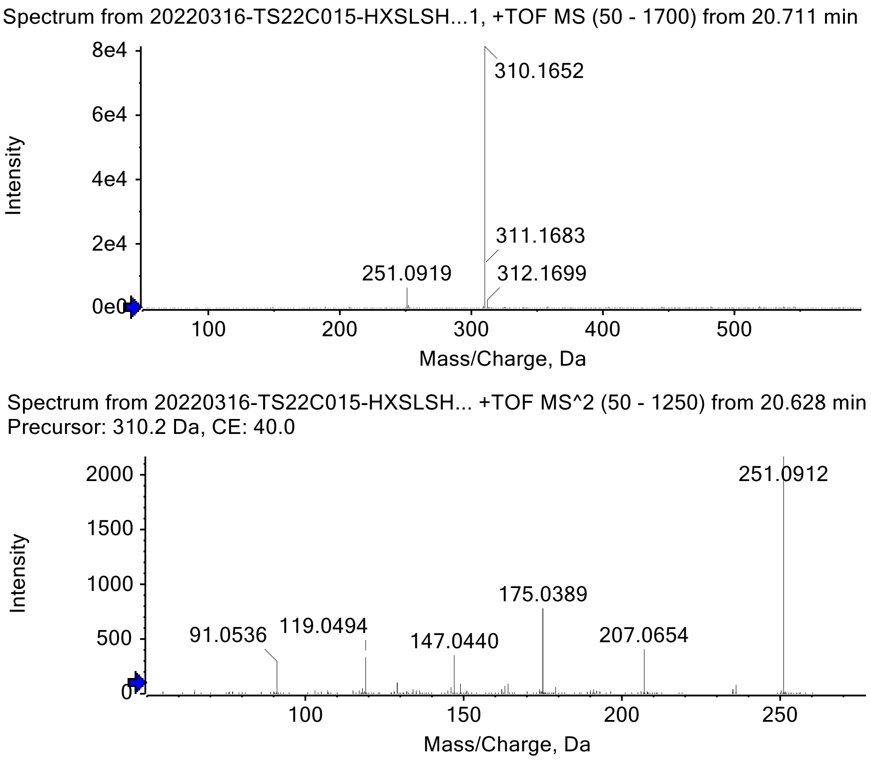

Figure S4-24: MS^{1}and MS^2 spectrograms of component 24 in HSSD

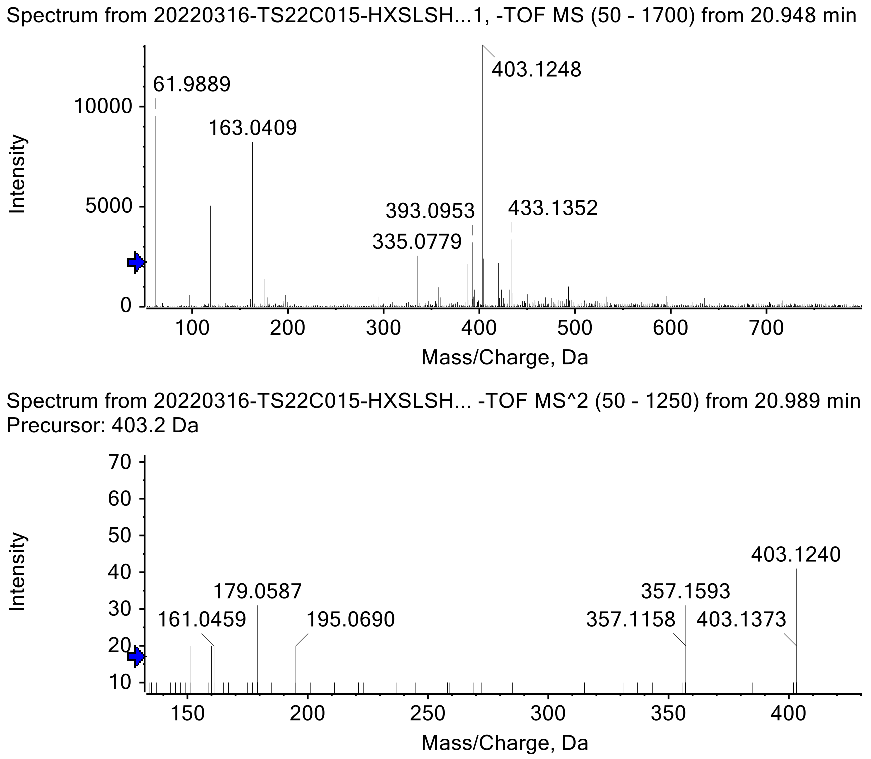

Figure S4-25: MS^{1}and MS^2 spectrograms of component 25 in HSSD

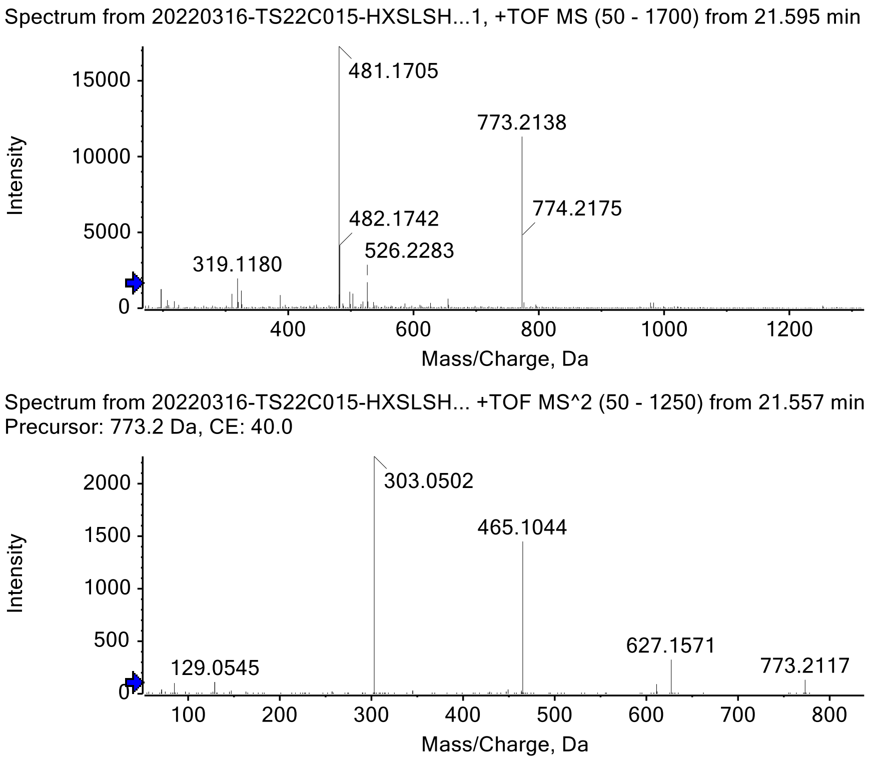

Figure S4-26: MS[1]and MS[2] spectrograms of component 26 in HSSD

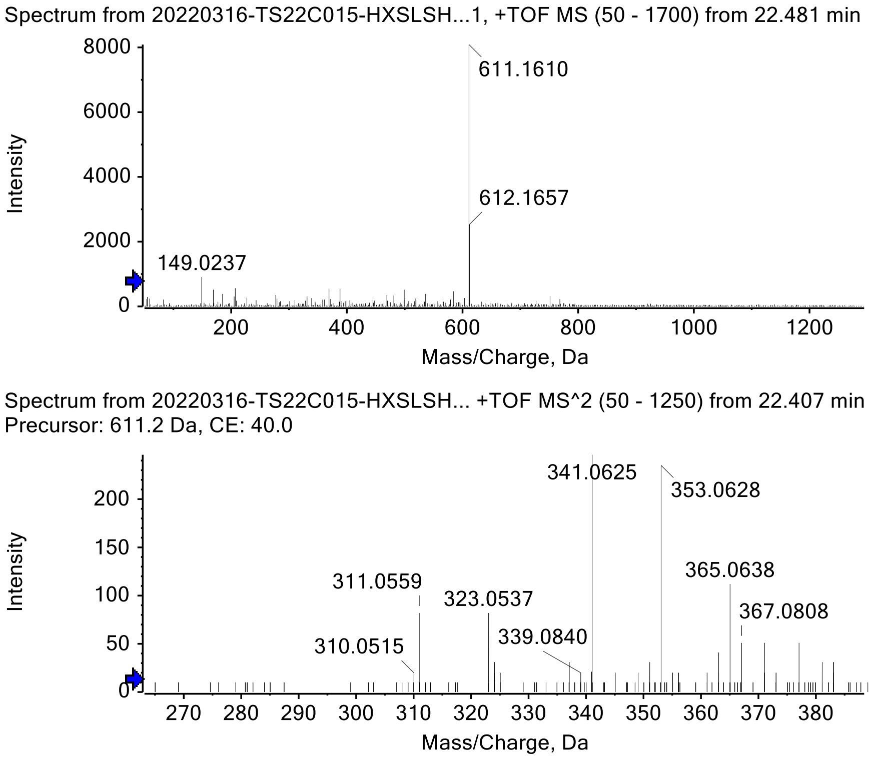

Figure S4-27: MS[1]and MS[2] spectrograms of component 27 in HSSD

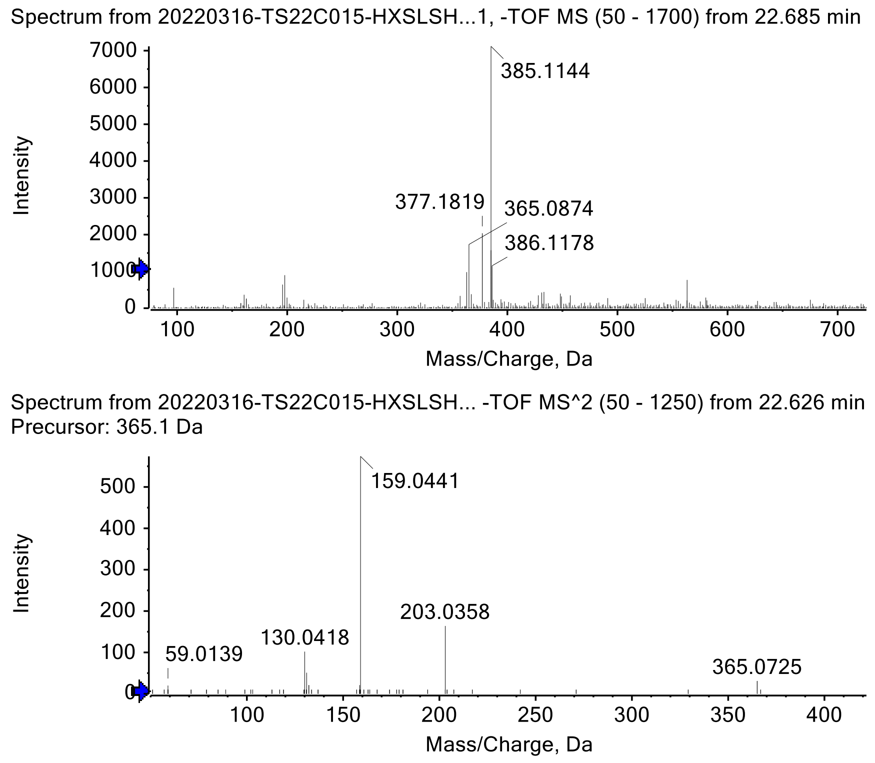

Figure S4-28: MS^{1}and MS^2 spectrograms of component 28 in HSSD

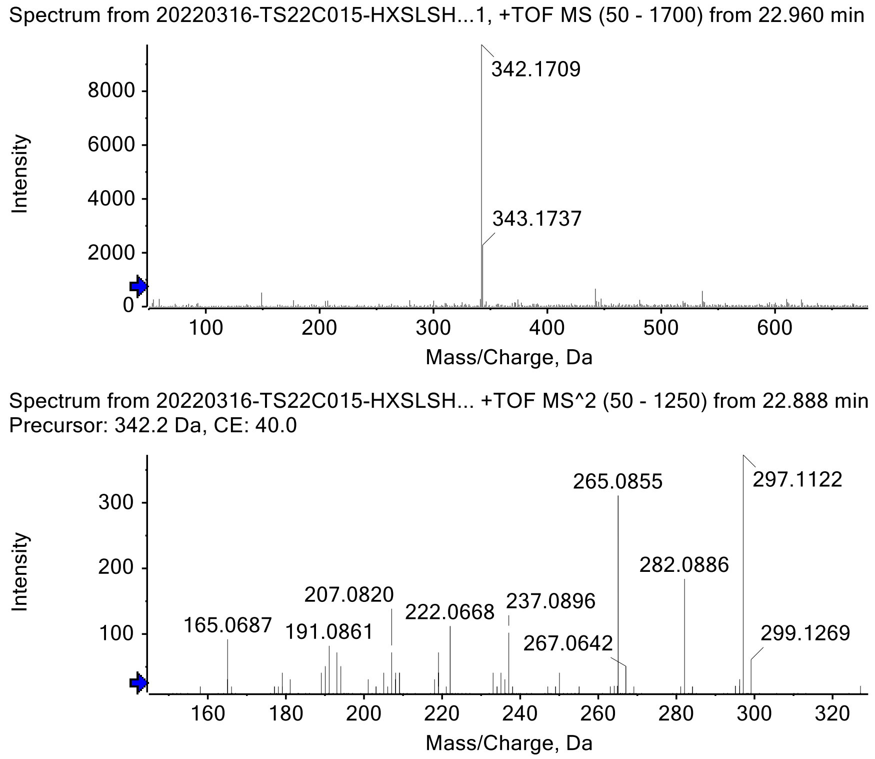

Figure S4-29: MS^{1}and MS^2 spectrograms of component 29 in HSSD

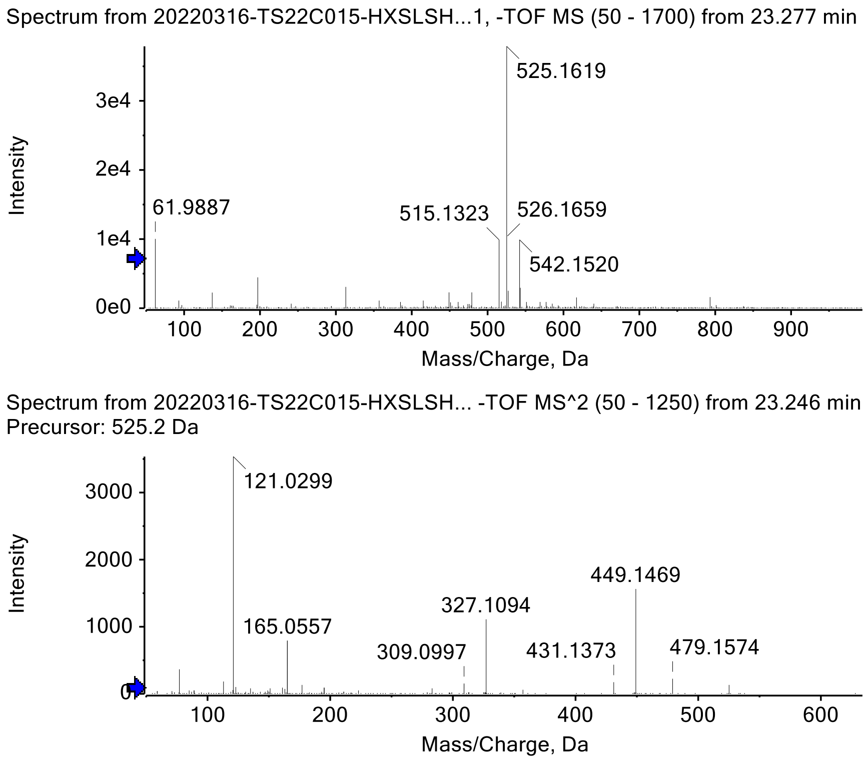

Figure S4-30: $MS^1$and MS^2 spectrograms of component 30 in HSSD

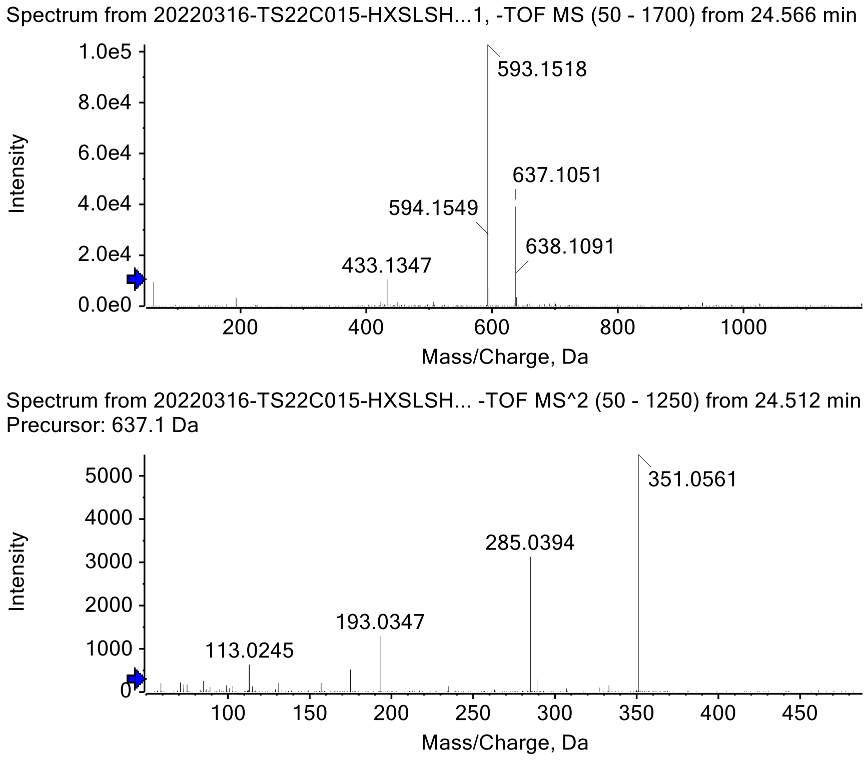

Figure S4-31: $MS^1$and MS^2 spectrograms of component 31 in HSSD

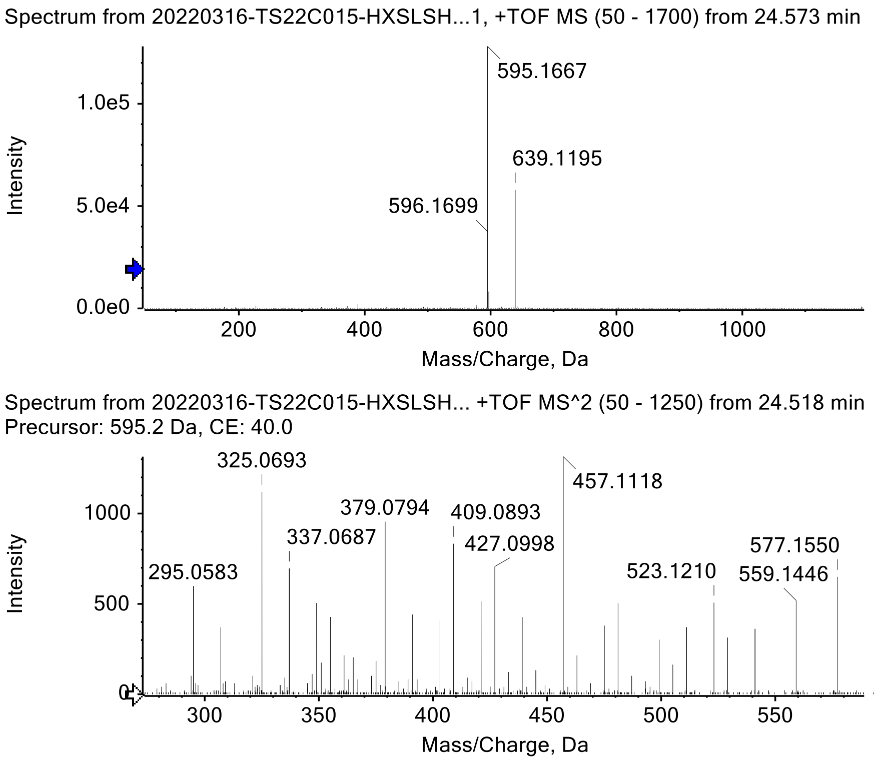

Figure S4-32: MS[1]and MS[2] spectrograms of component 32 in HSSD

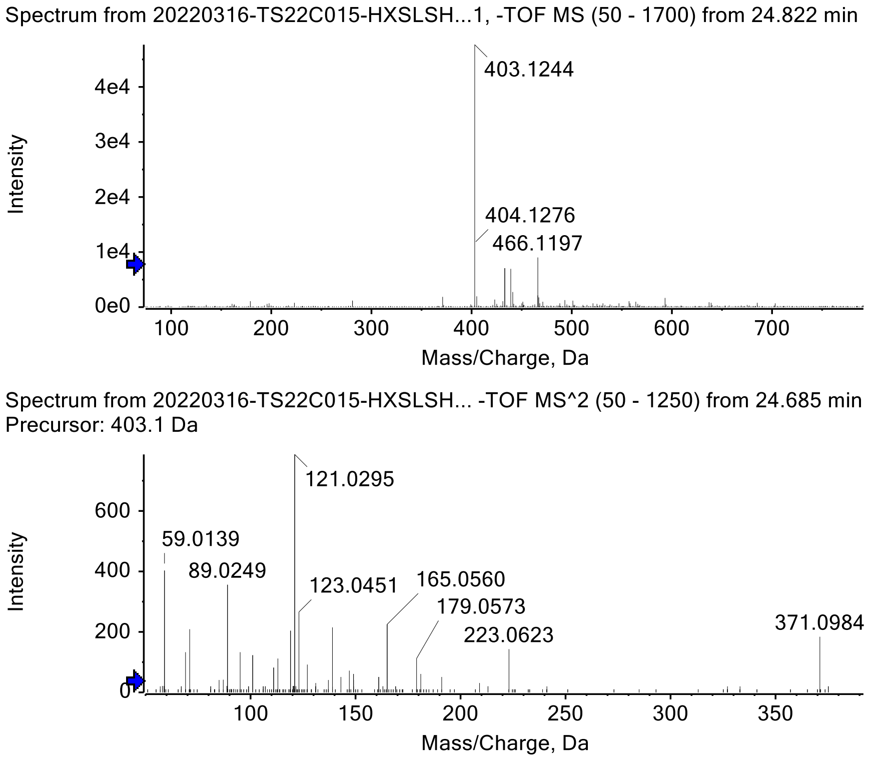

Figure S4-33: MS[1]and MS[2] spectrograms of component 33 in HSSD

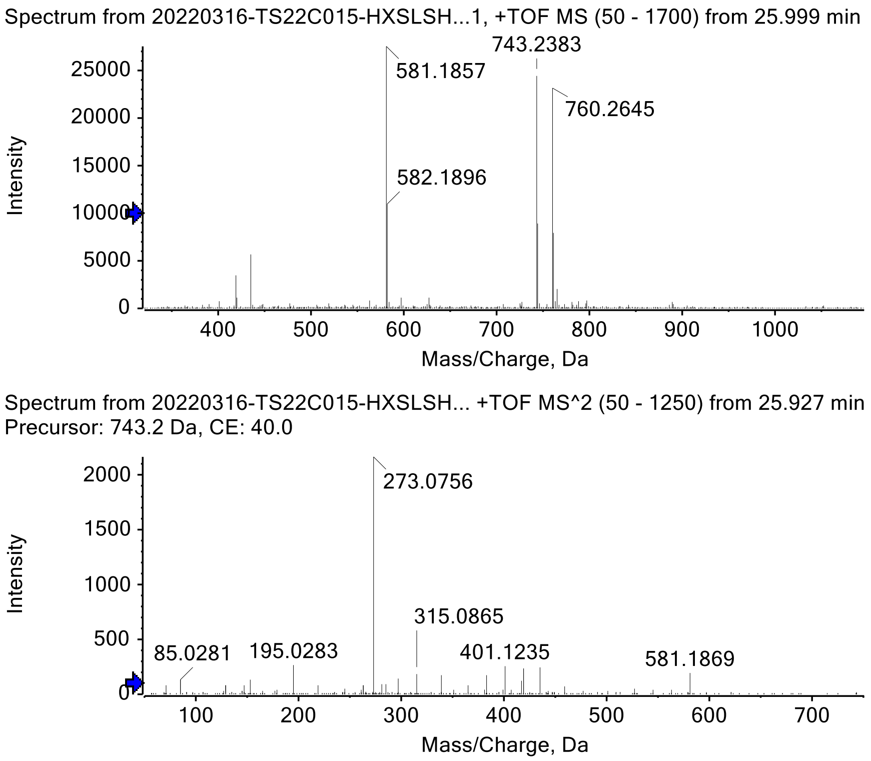

Figure S4-34: $MS^1$and MS^2 spectrograms of component 34 in HSSD

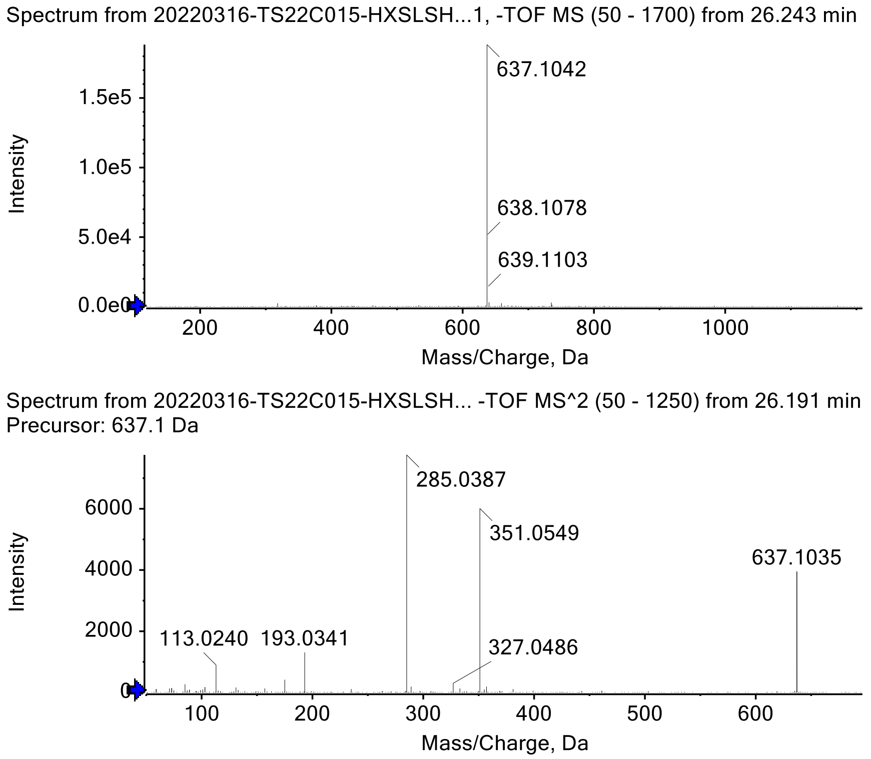

Figure S4-35: $MS^1$and MS^2 spectrograms of component 35 in HSSD

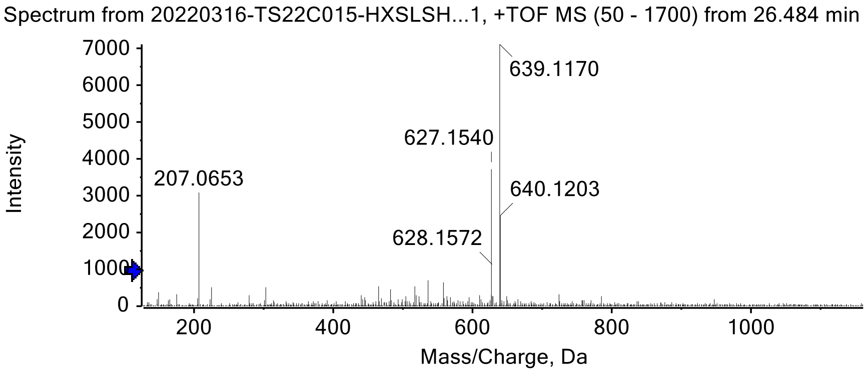

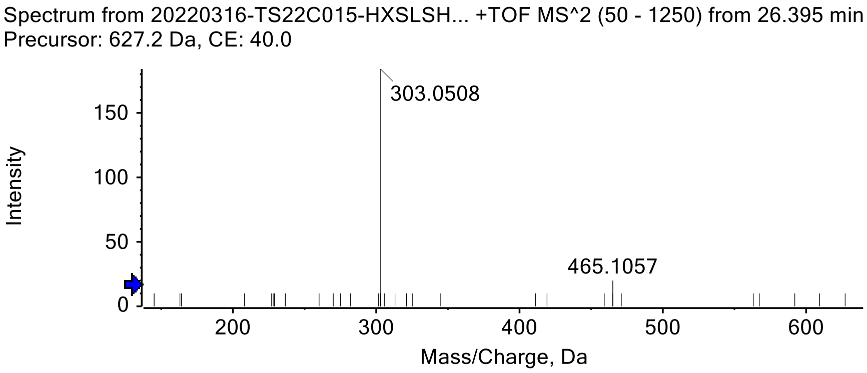

Figure S4-36: MS[1]and MS[2] spectrograms of component 36 in HSSD

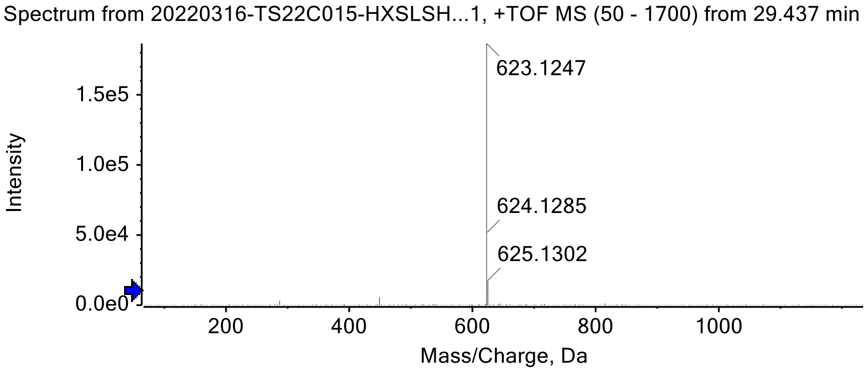

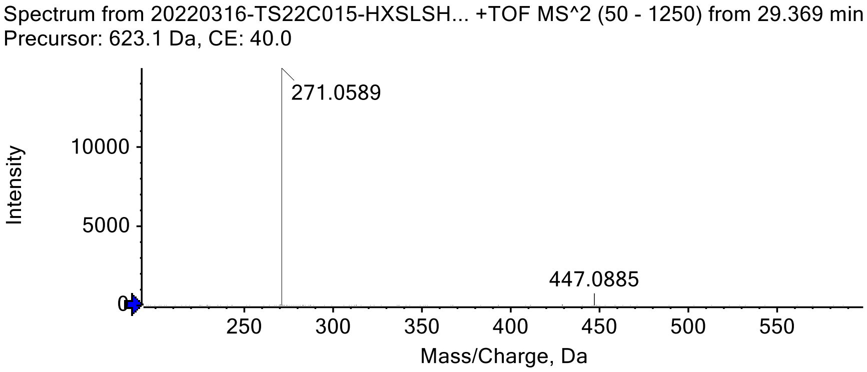

Figure S4-37: MS[1]and MS[2] spectrograms of component 37 in HSSD

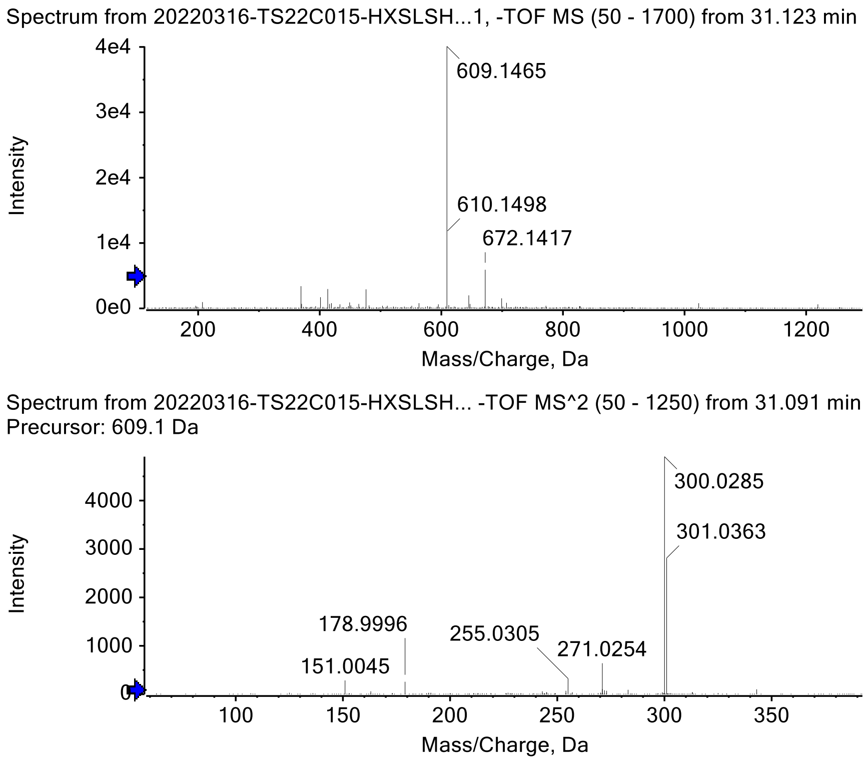

Figure S4-38: MS[1]and MS[2] spectrograms of component 38 in HSSD

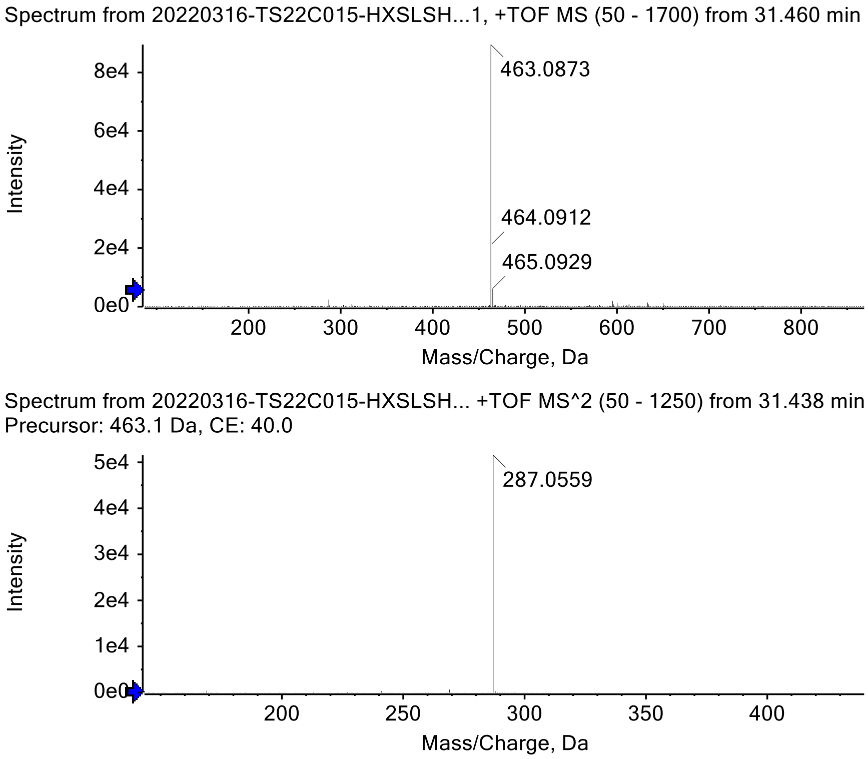

Figure S4-39: MS[1]and MS[2] spectrograms of component 39 in HSSD

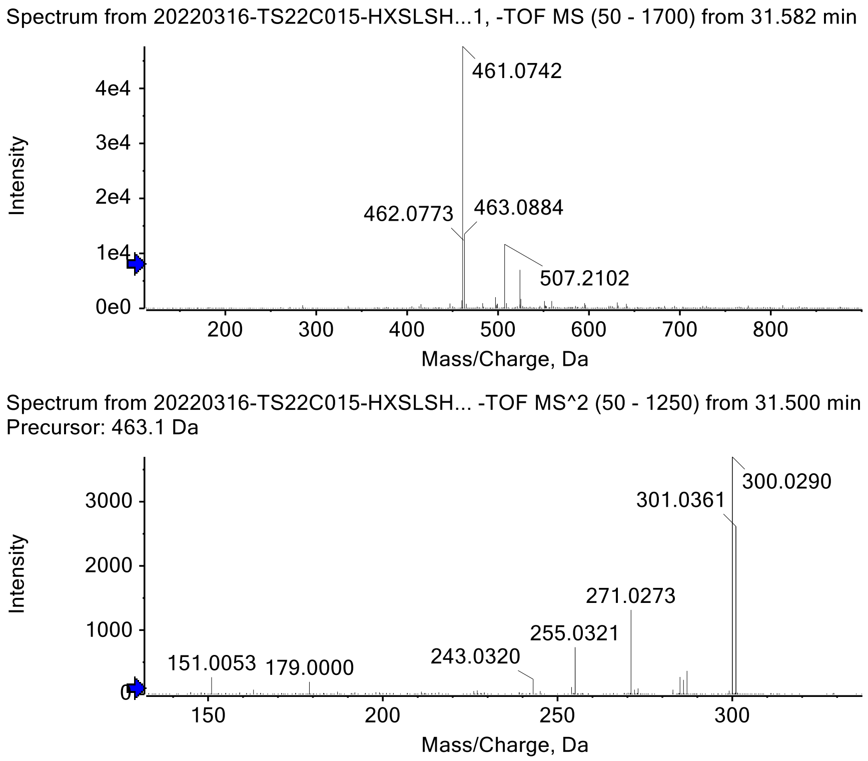

Figure S4-40: MS^{1}and MS^2 spectrograms of component 40 in HSSD

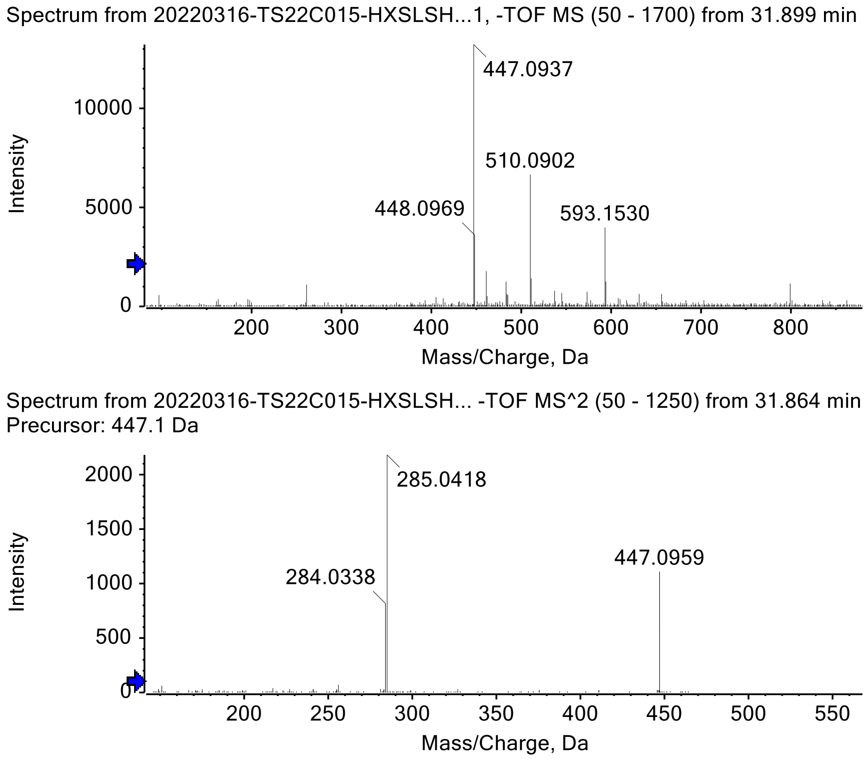

Figure S4-41: MS^{1}and MS^2 spectrograms of component 41 in HSSD

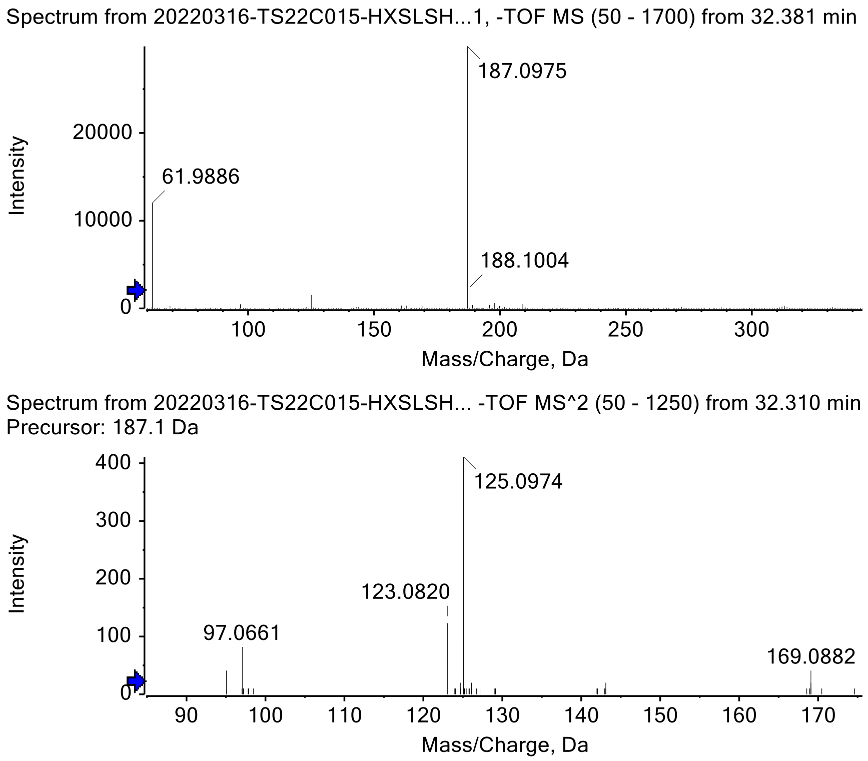

Figure S4-42: $MS^1$and MS^2 spectrograms of component 42 in HSSD

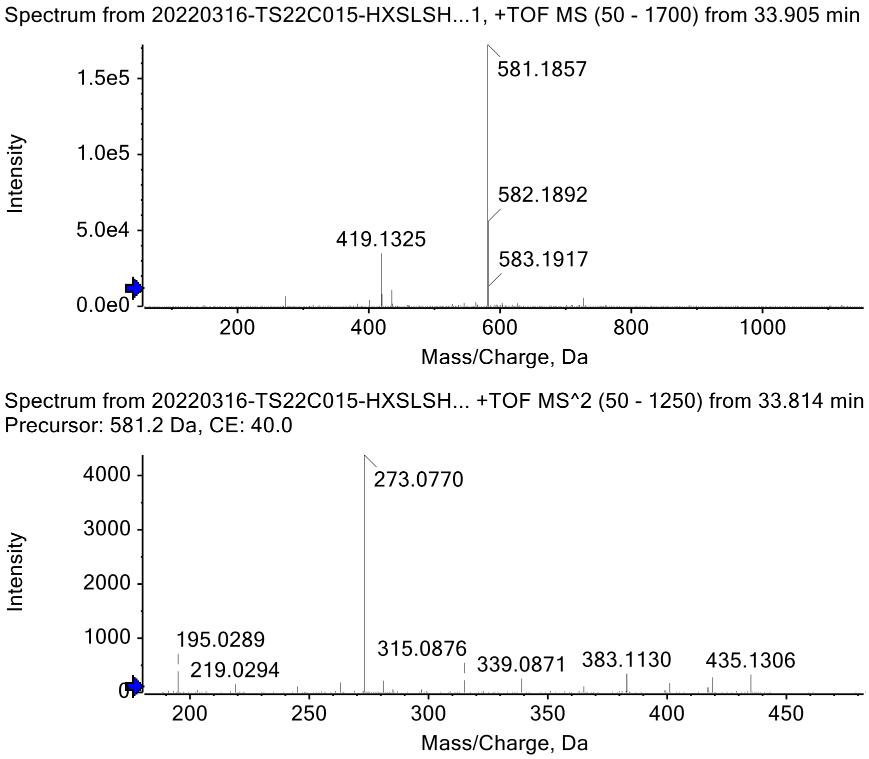

Figure S4-43: $MS^1$and MS^2 spectrograms of component 43 in HSSD

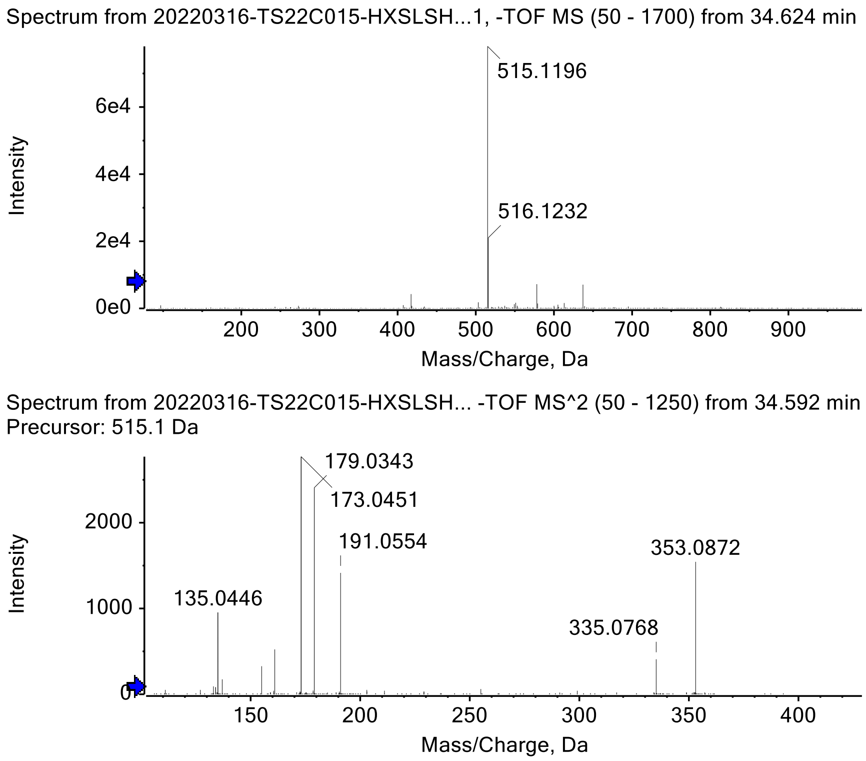

Figure S4-44: MS^{1}and MS^2 spectrograms of component 44 in HSSD

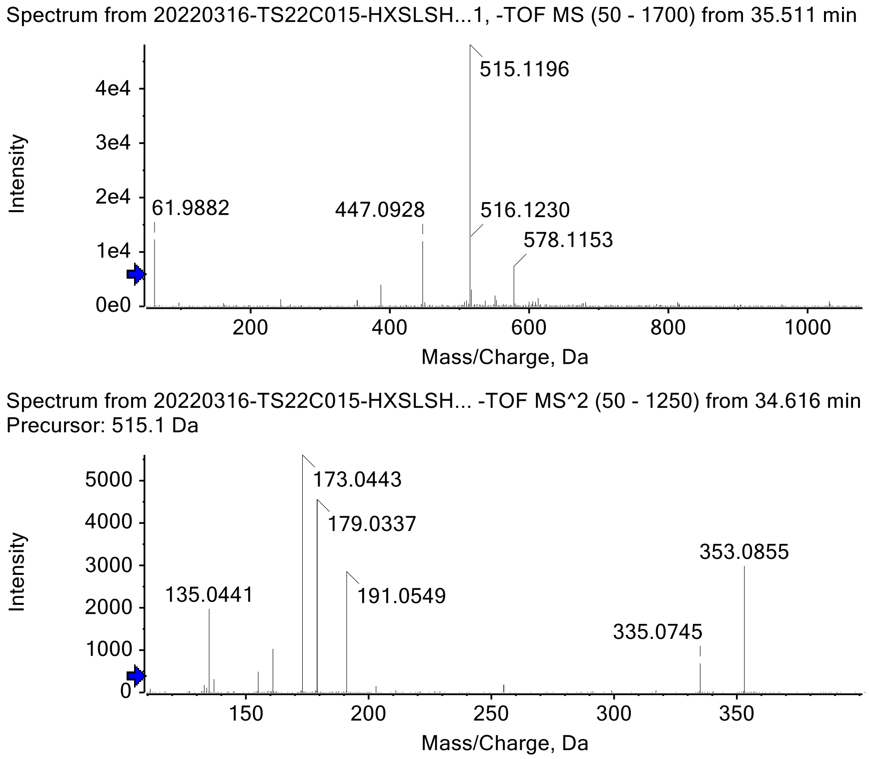

Figure S4-45: MS^{1}and MS^2 spectrograms of component 45 in HSSD

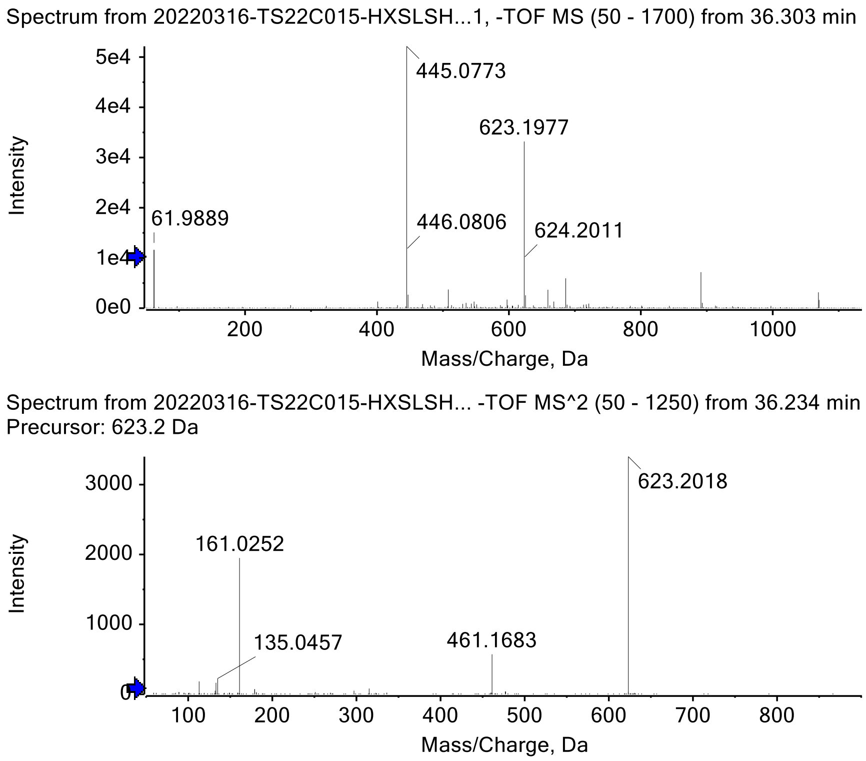

Figure S4-46: $MS^1$and MS^2 spectrograms of component 46 in HSSD

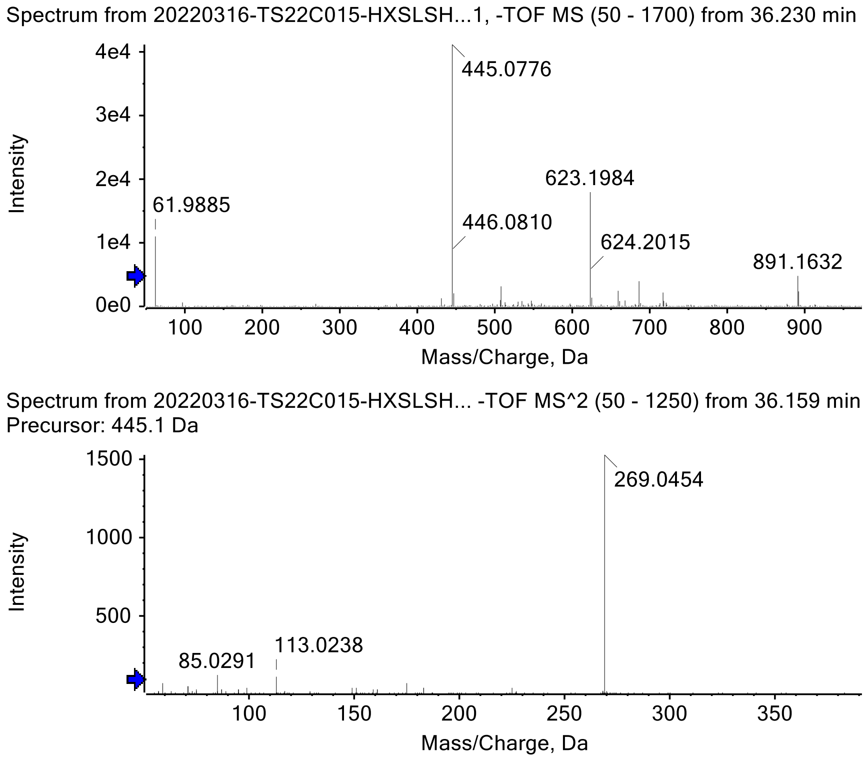

Figure S4-47: $MS^1$and MS^2 spectrograms of component 47 in HSSD

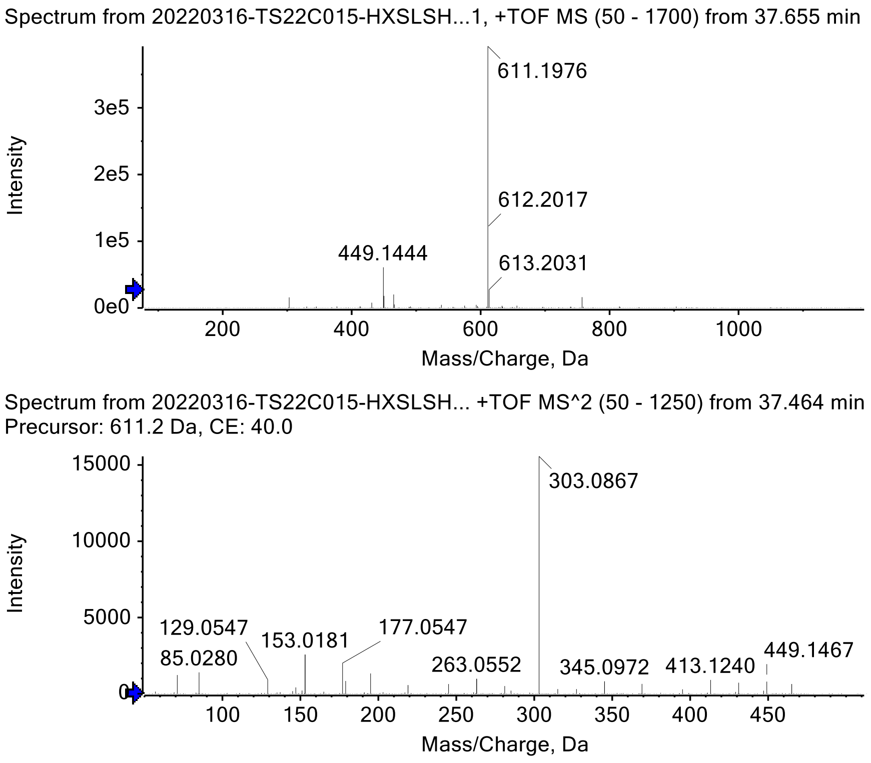

Figure S4-48: MS[1]and MS[2] spectrograms of component 48 in HSSD

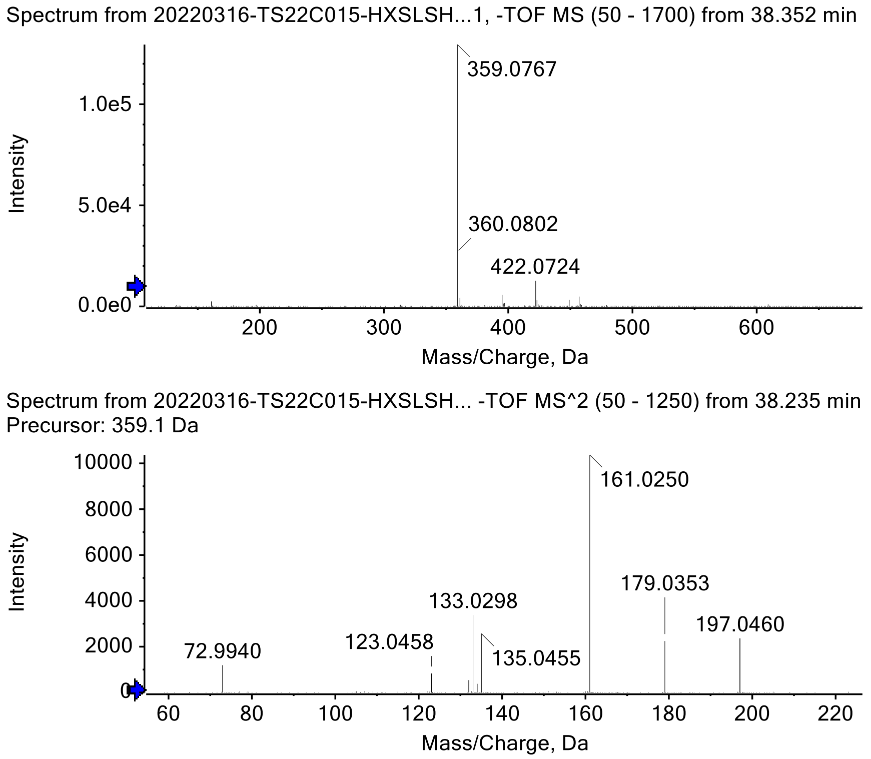

Figure S4-49: MS[1]and MS[2] spectrograms of component 49 in HSSD

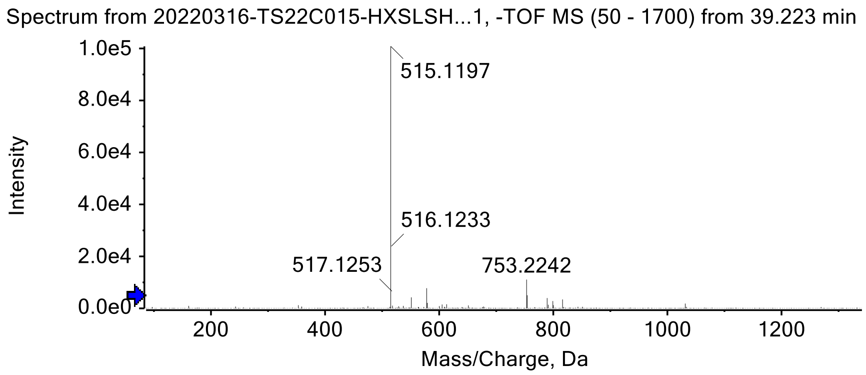

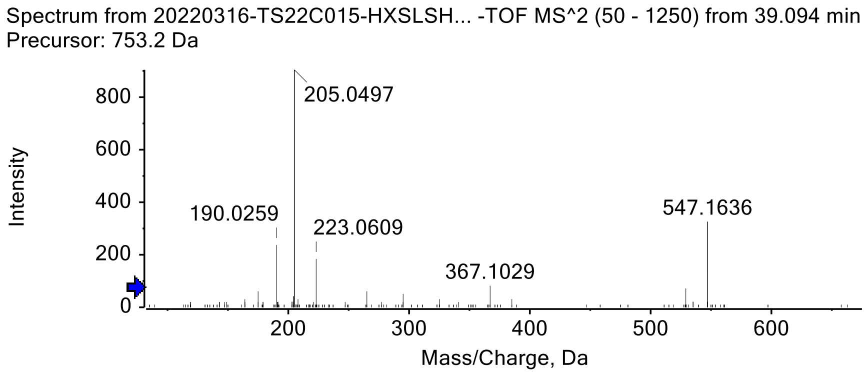

Figure S4-50: $MS^1$and MS^2 spectrograms of component 50 in HSSD

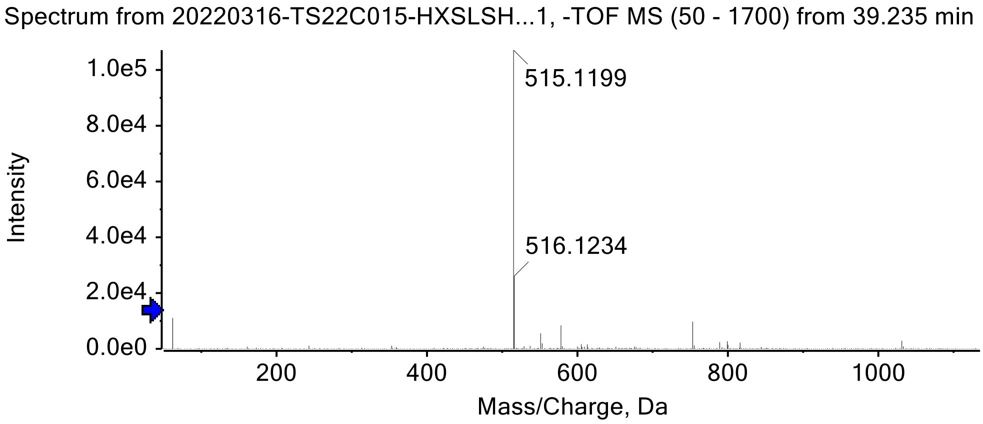

Figure S4-51: MS^1 spectrograms of component 51 in HSSD

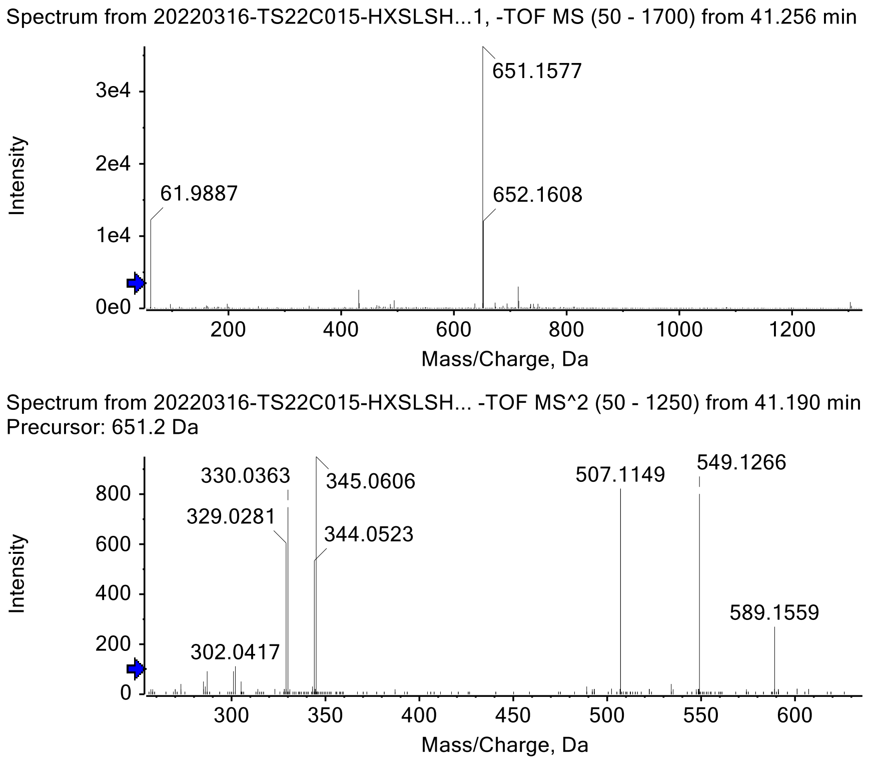

Figure S4-52: MS[1]and MS[2] spectrograms of component 52 in HSSD

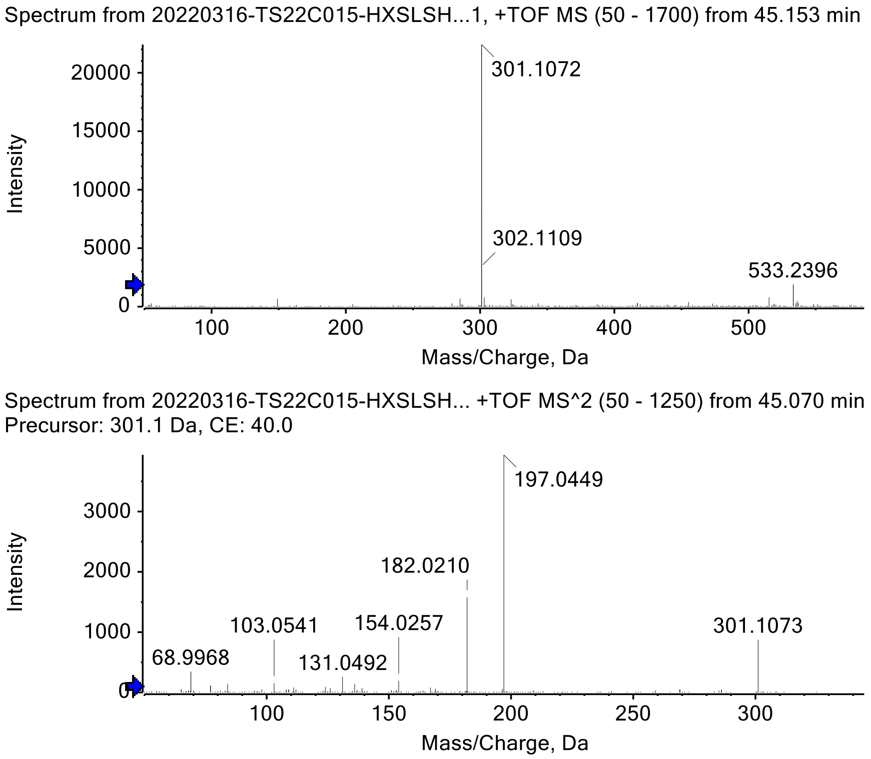

Figure S4-53: MS[1]and MS[2] spectrograms of component 53 in HSSD

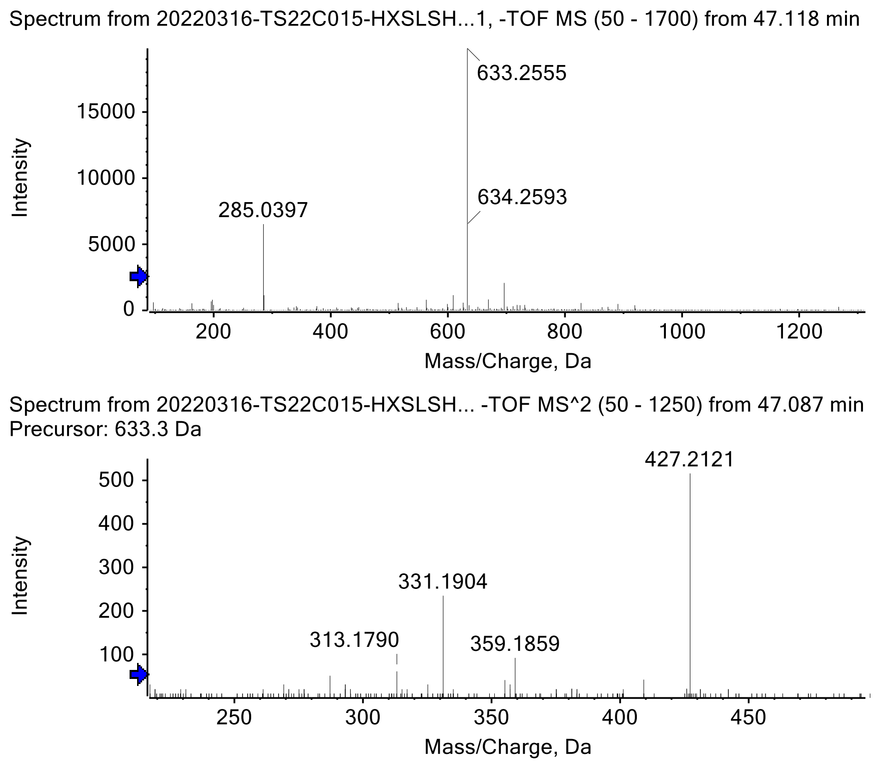

Figure S4-54: $MS^1$and MS^2 spectrograms of component 54 in HSSD

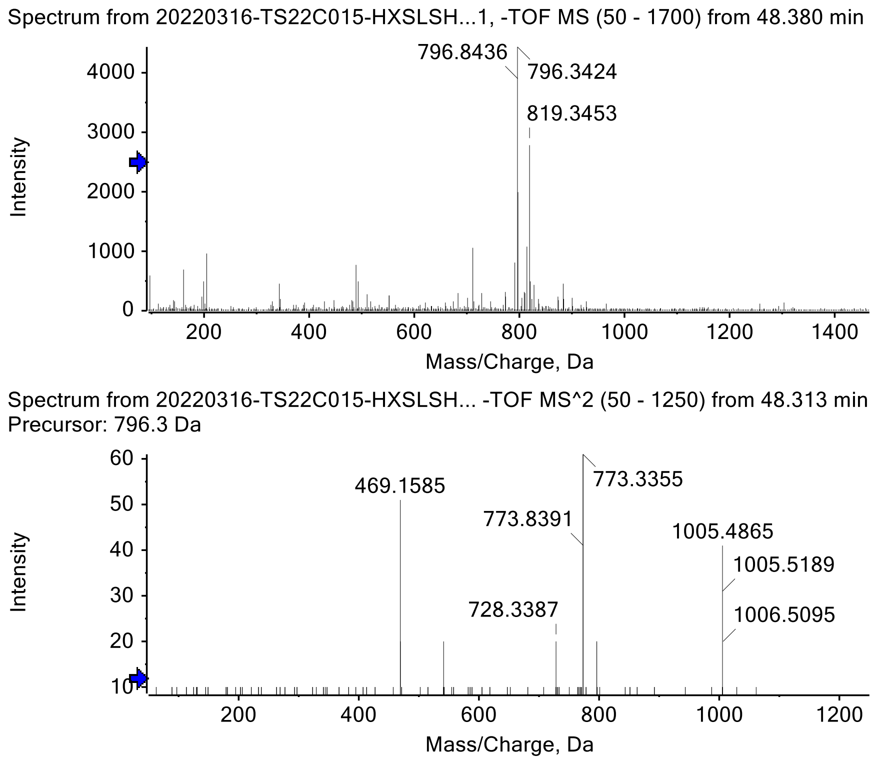

Figure S4-55: $MS^1$and MS^2 spectrograms of component 55 in HSSD

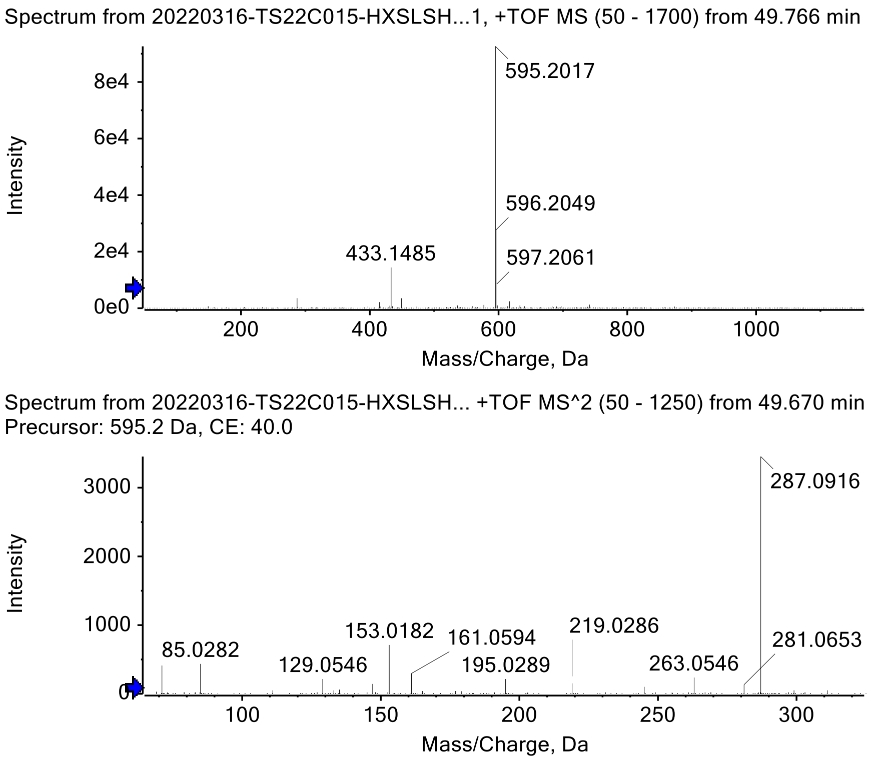

Figure S4-56: MS^{1}and MS^2 spectrograms of component 56 in HSSD

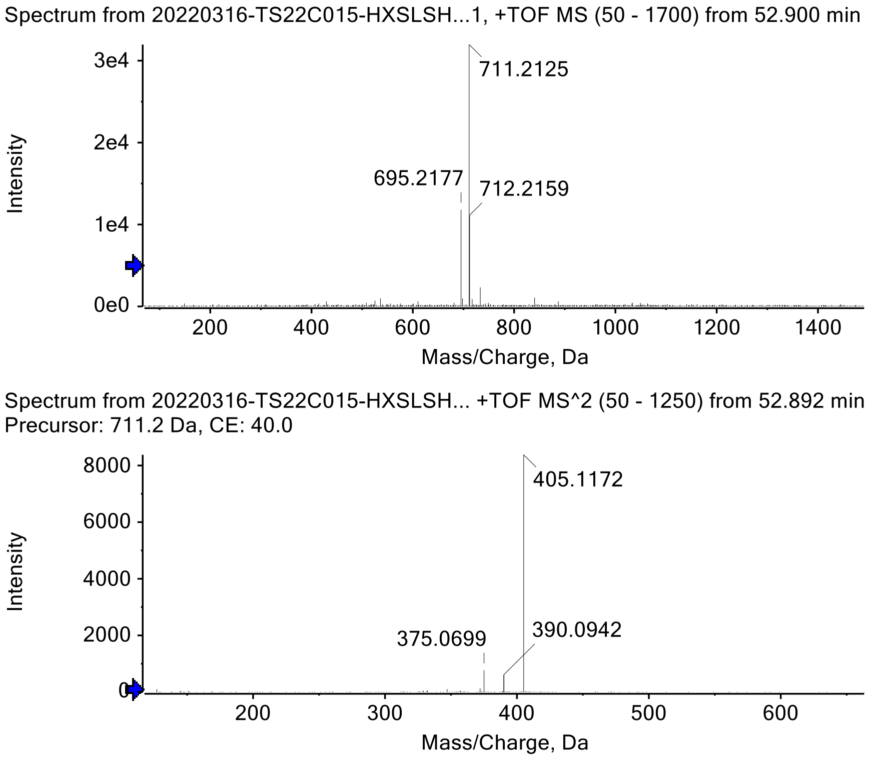

Figure S4-57: MS^{1}and MS^2 spectrograms of component 57 in HSSD

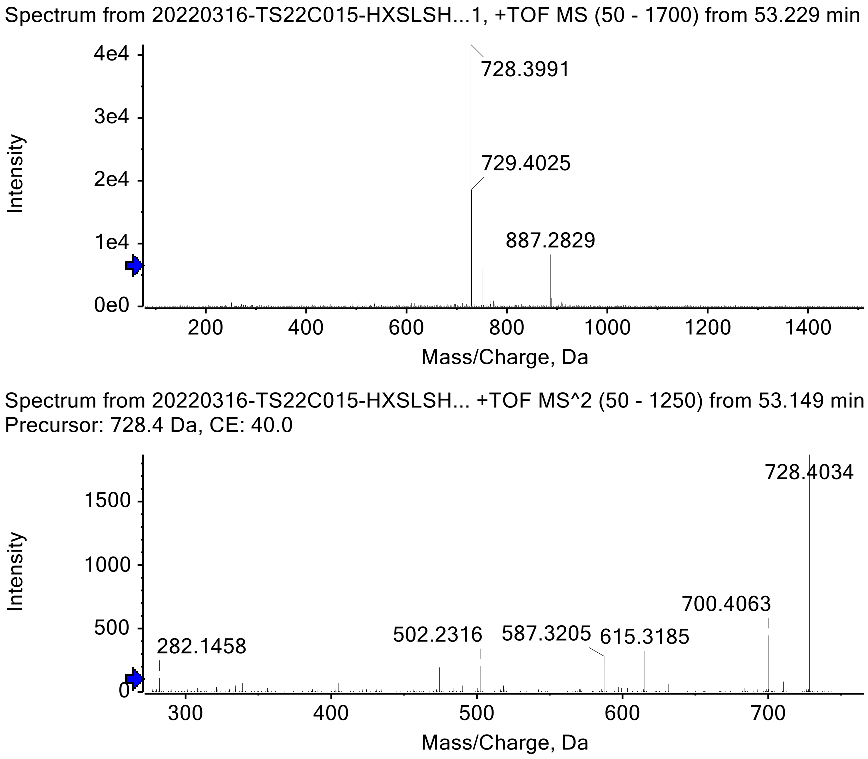

Figure S4-58: MS[1]and MS[2] spectrograms of component 58 in HSSD

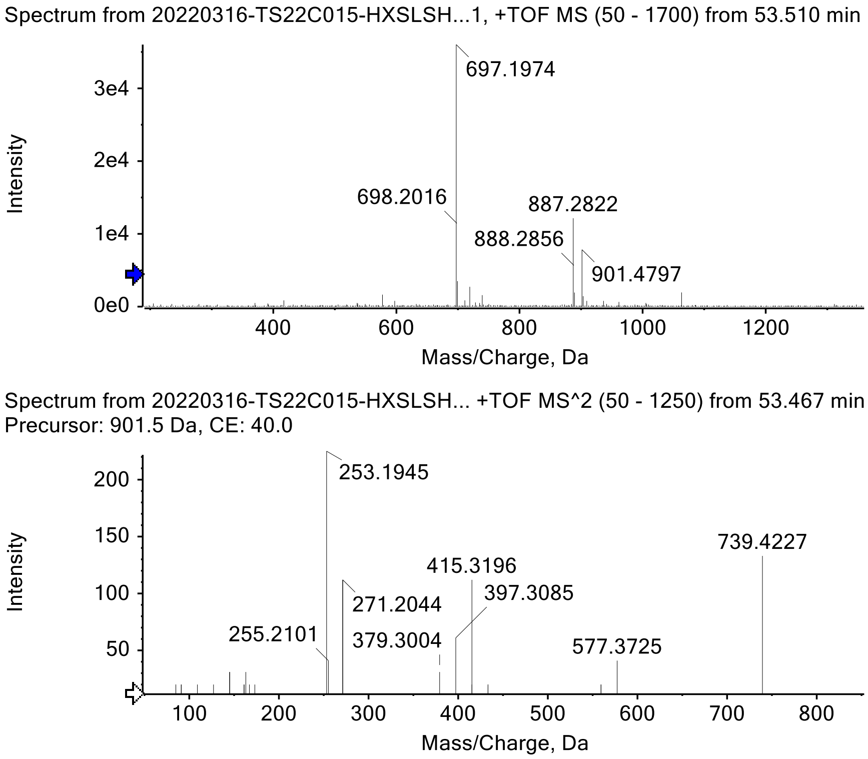

Figure S4-59: MS[1]and MS[2] spectrograms of component 59 in HSSD

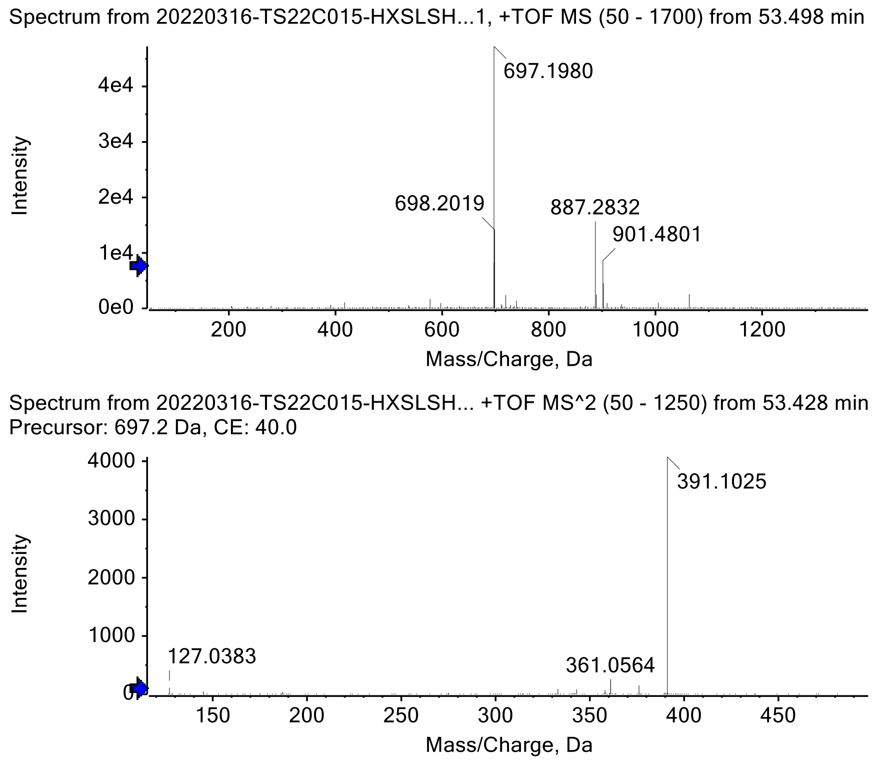

Figure S4-60: MS[1]and MS[2] spectrograms of component 60 in HSSD

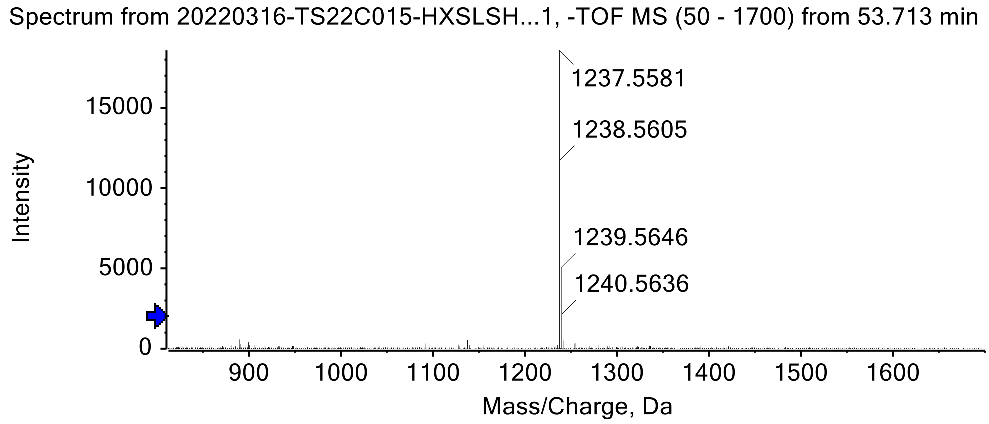

Figure S4-61: MS[1] spectrograms of component 61 in HSSD

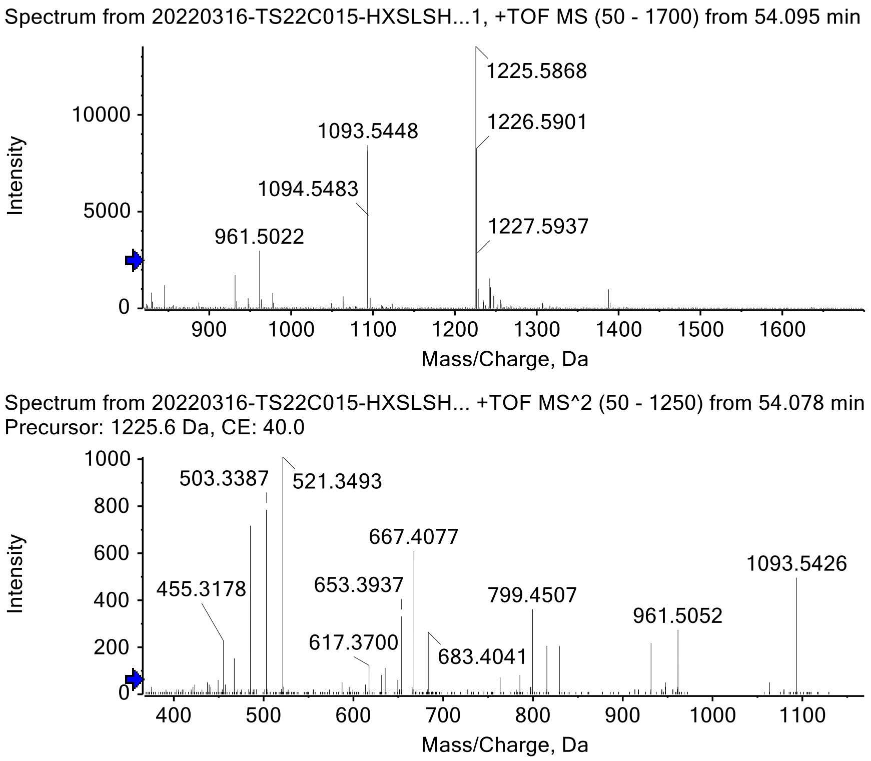

Figure S4-62: MS[1]and MS[2] spectrograms of component 62 in HSSD

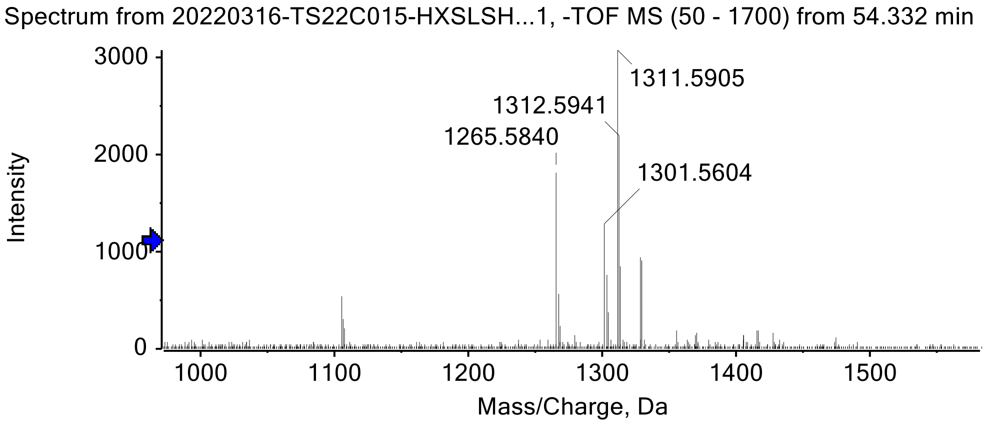

Figure S4-63: MS[1] spectrograms of component 63 in HSSD

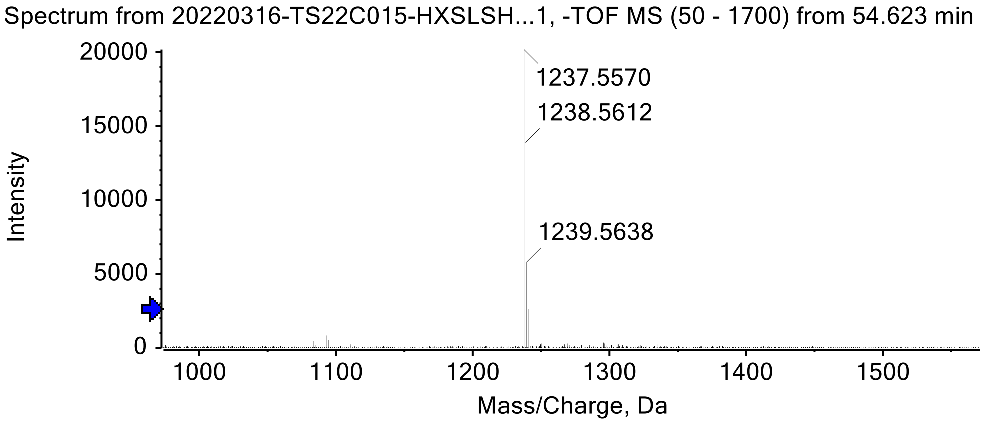

Figure S4-64: MS^1 spectrograms of component 64 in HSSD

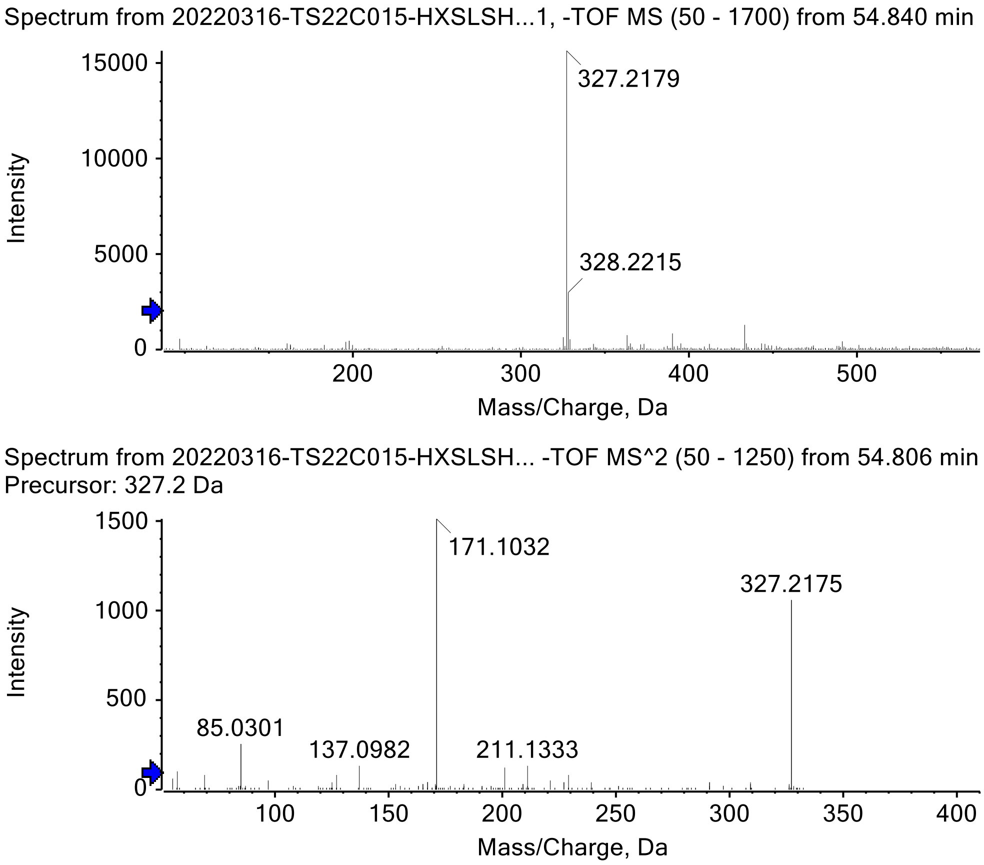

Figure S4-65: $MS^1$and MS^2 spectrograms of component 65 in HSSD

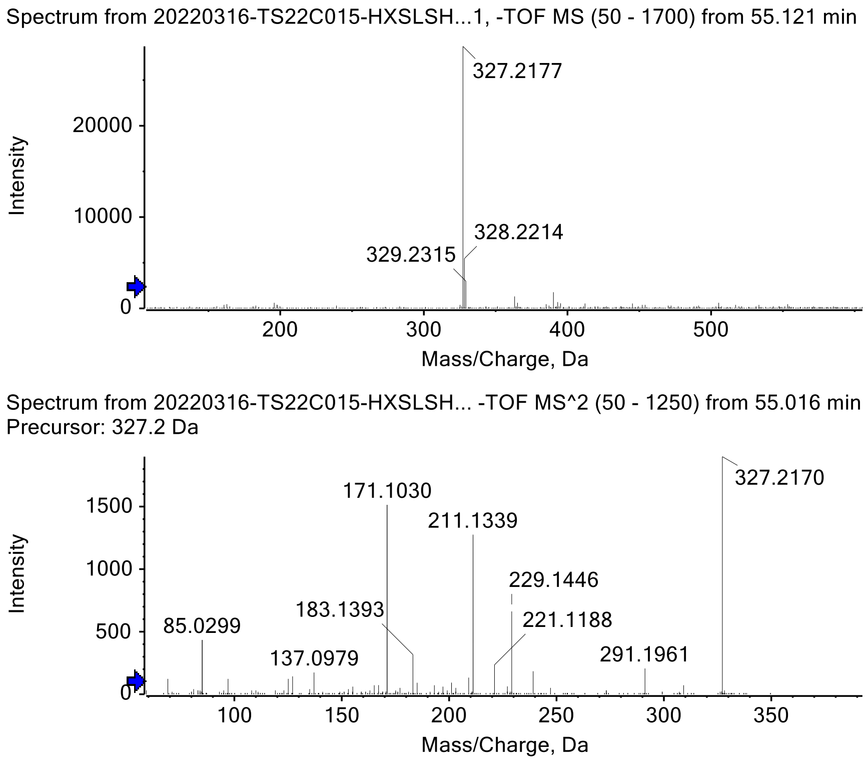

Figure S4-66: MS[1]and MS[2] spectrograms of component 66 in HSSD

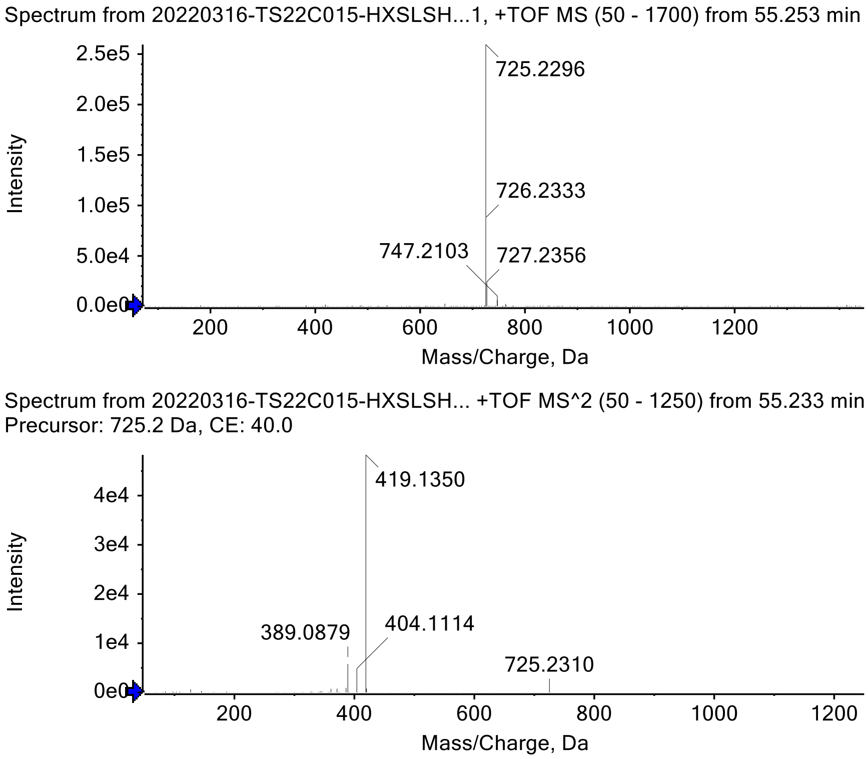

Figure S4-67: MS[1]and MS[2] spectrograms of component 67 in HSSD

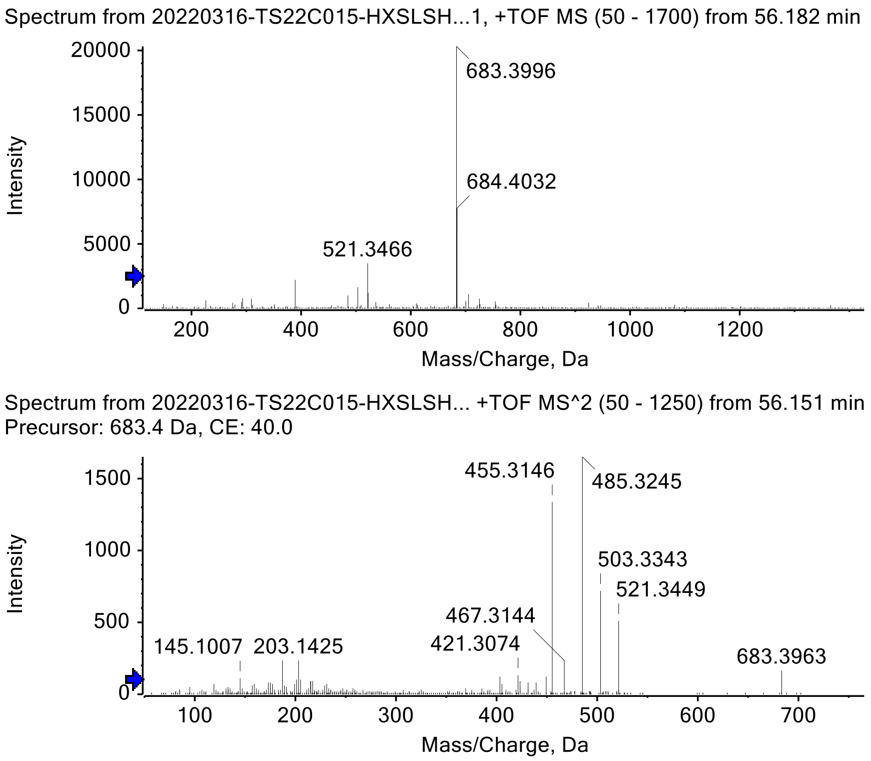

Figure S4-68: MS[1]and MS[2] spectrograms of component 68 in HSSD

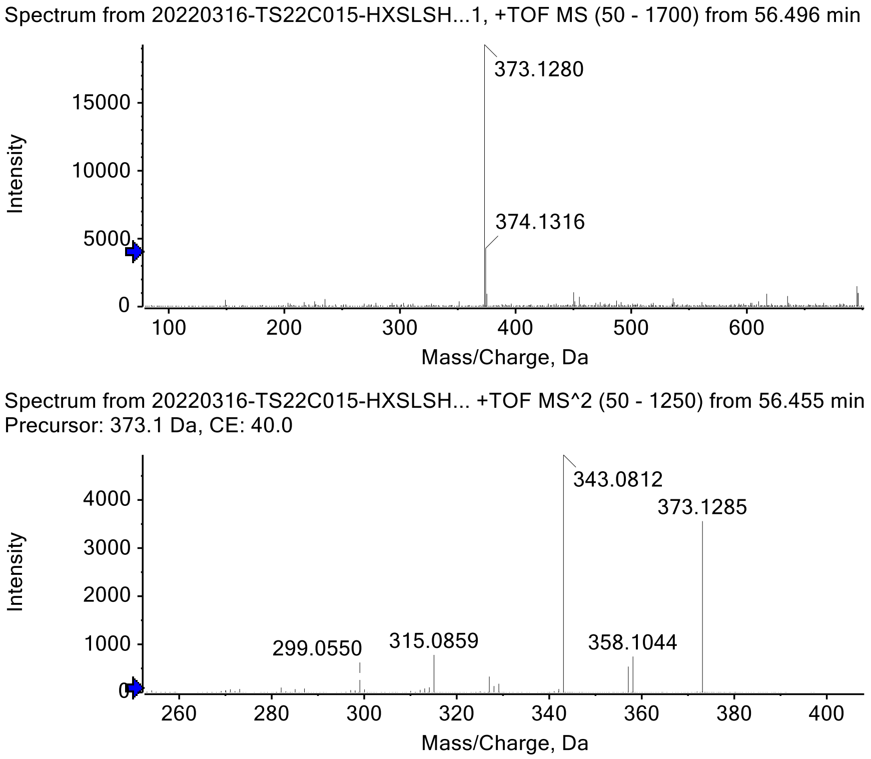

Figure S4-69: MS[1]and MS[2] spectrograms of component 69 in HSSD

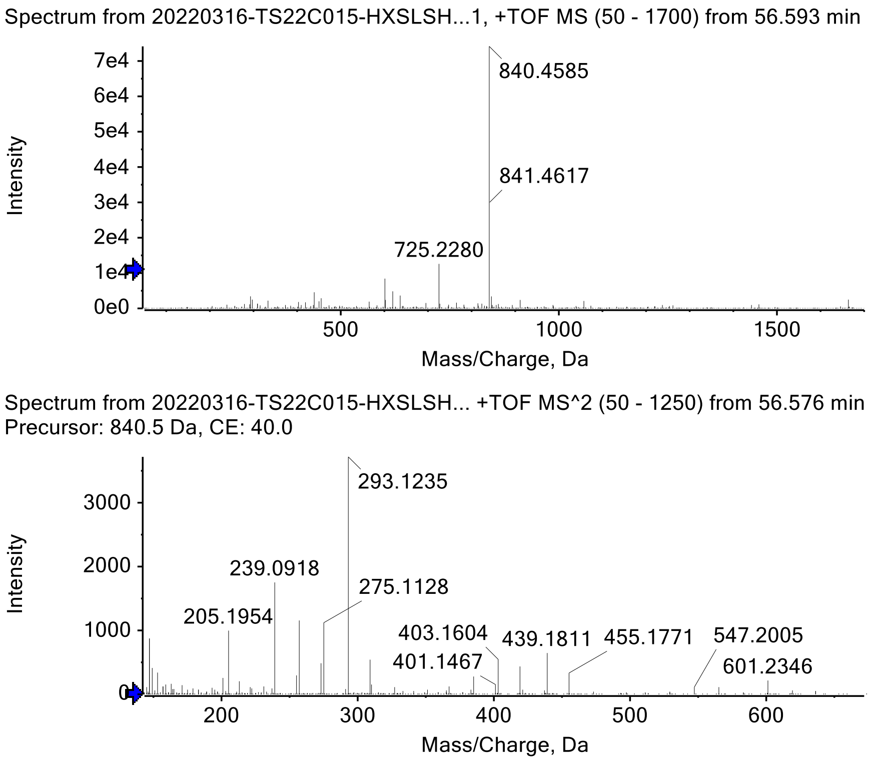

Figure S4-70: MS^{1}and MS^2 spectrograms of component 70 in HSSD

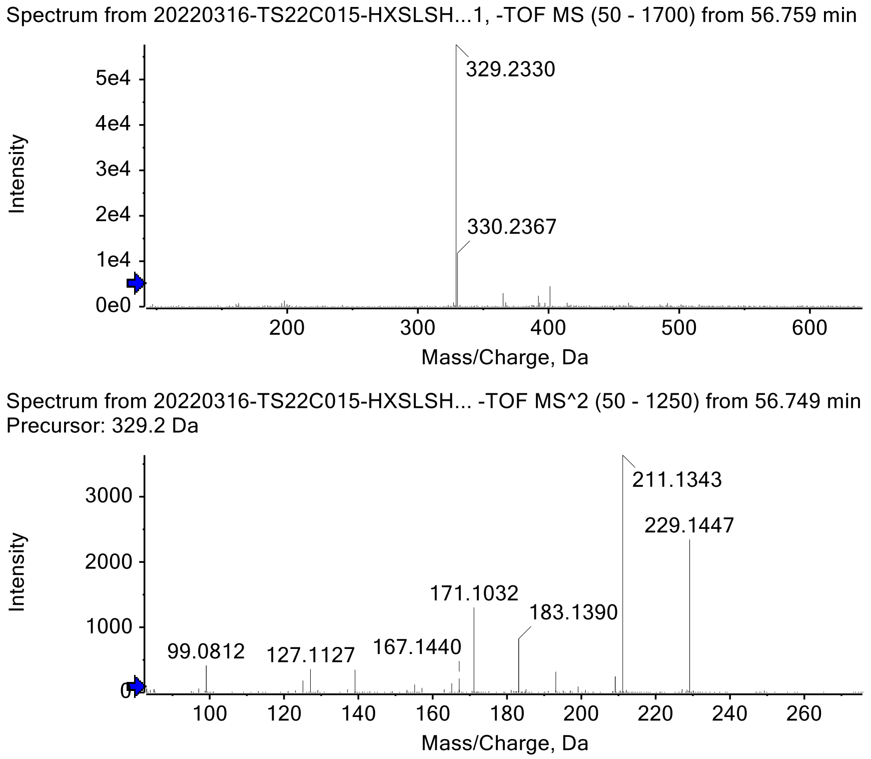

Figure S4-71: MS^{1}and MS^2 spectrograms of component 71 in HSSD

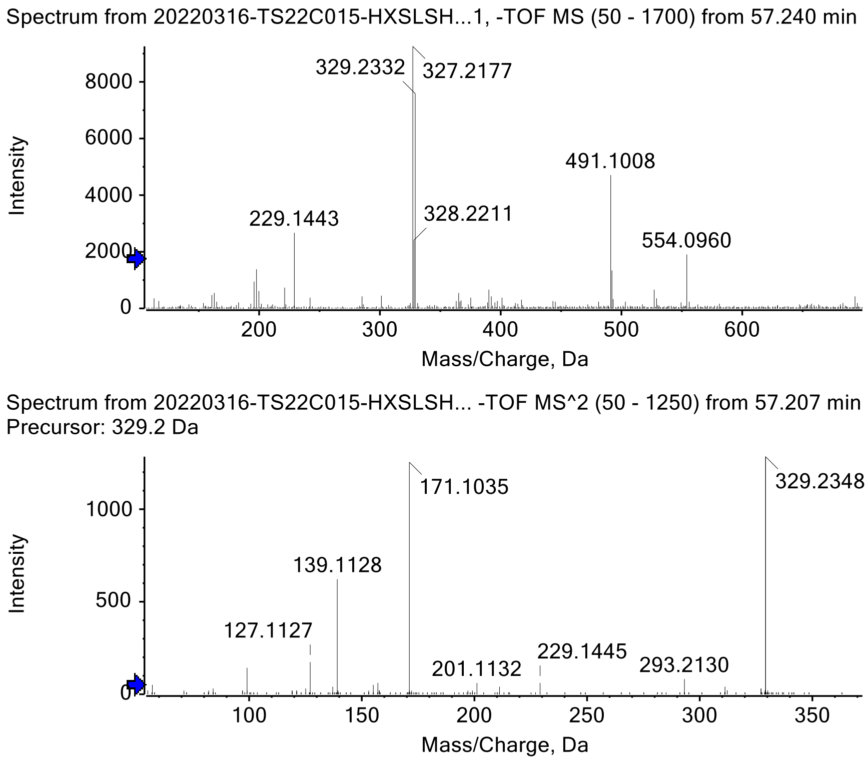

Figure S4-72: $MS^1$and MS^2 spectrograms of component 72 in HSSD

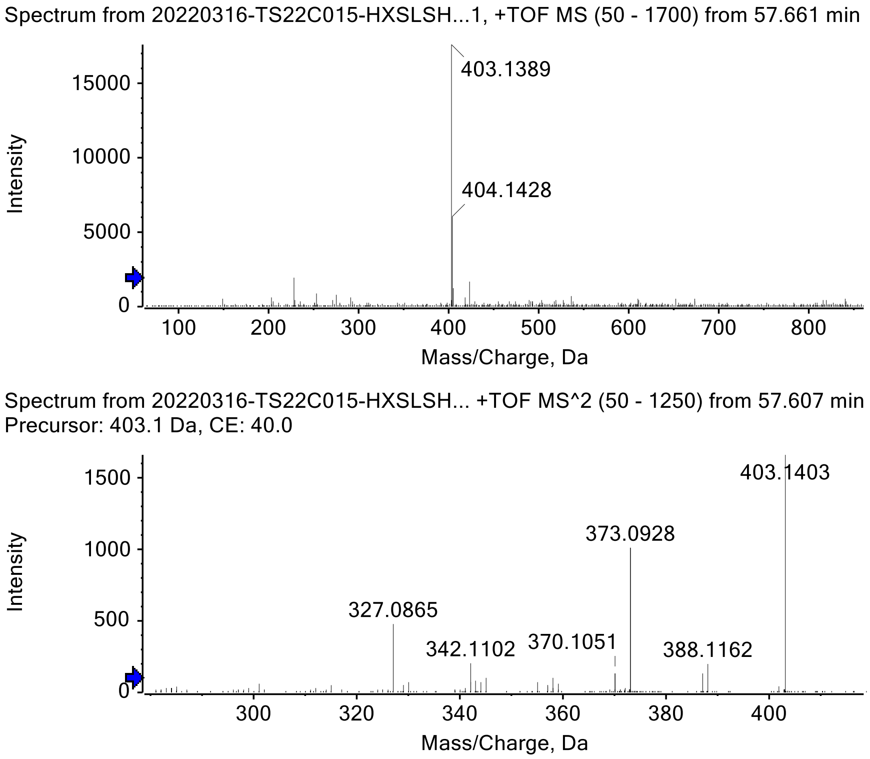

Figure S4-73: $MS^1$and MS^2 spectrograms of component 73 in HSSD

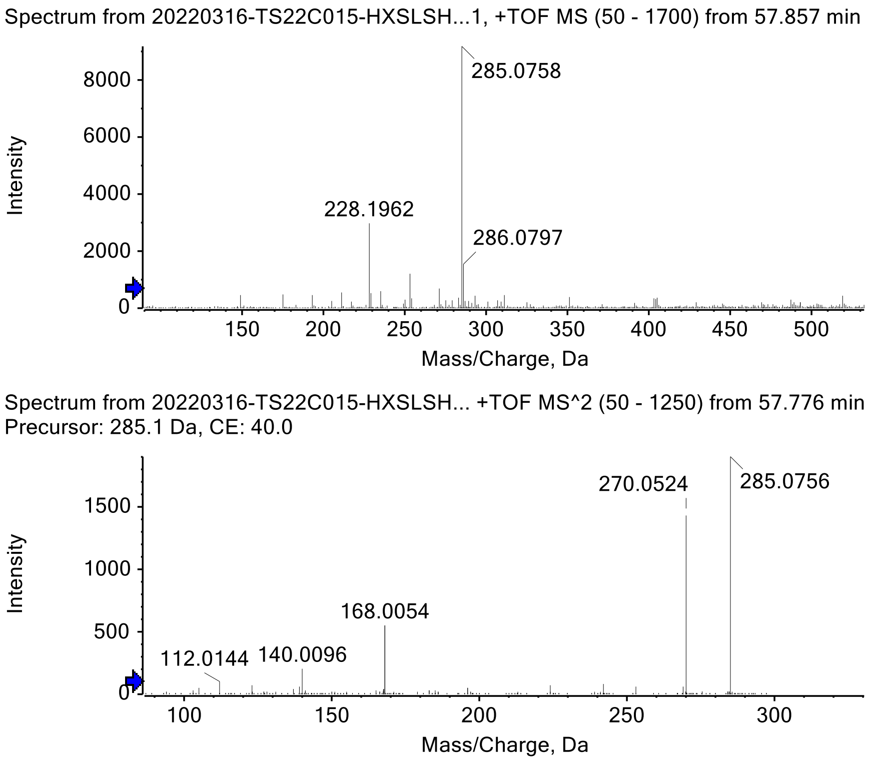

Figure S4-74: MS[1]and MS[2] spectrograms of component 74 in HSSD

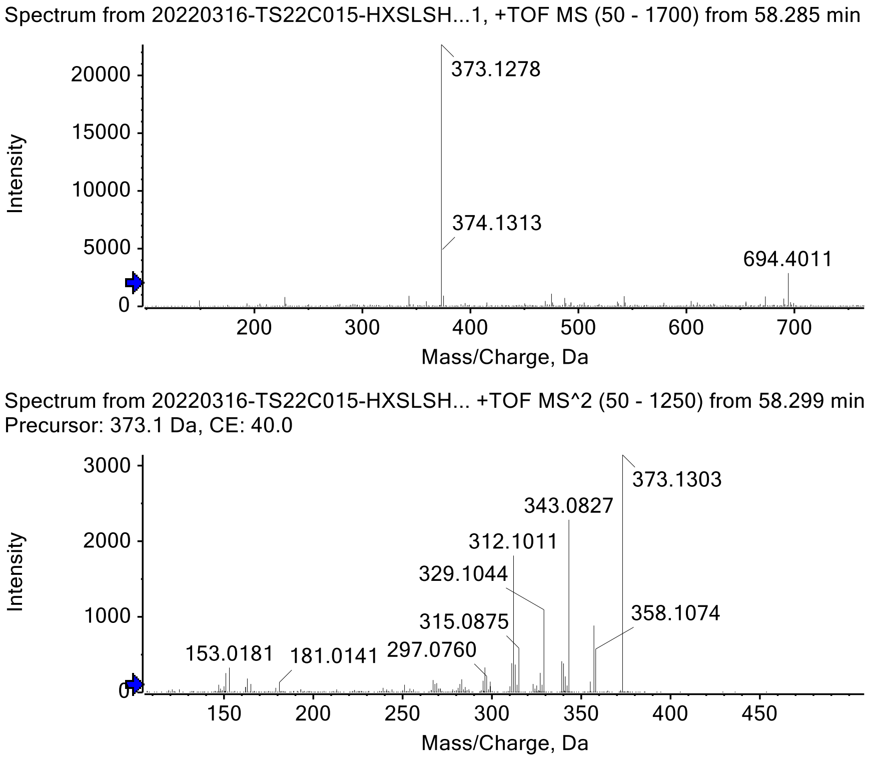

Figure S4-75: MS[1]and MS[2] spectrograms of component 75 in HSSD

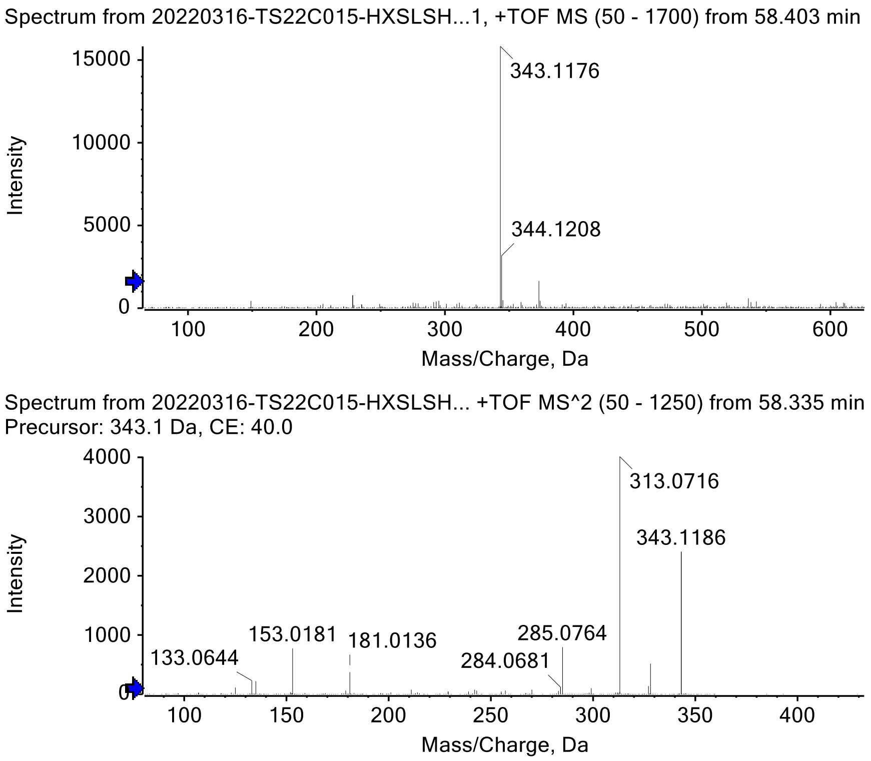

Figure S4-76: MS^{1}and MS^2 spectrograms of component 76 in HSSD

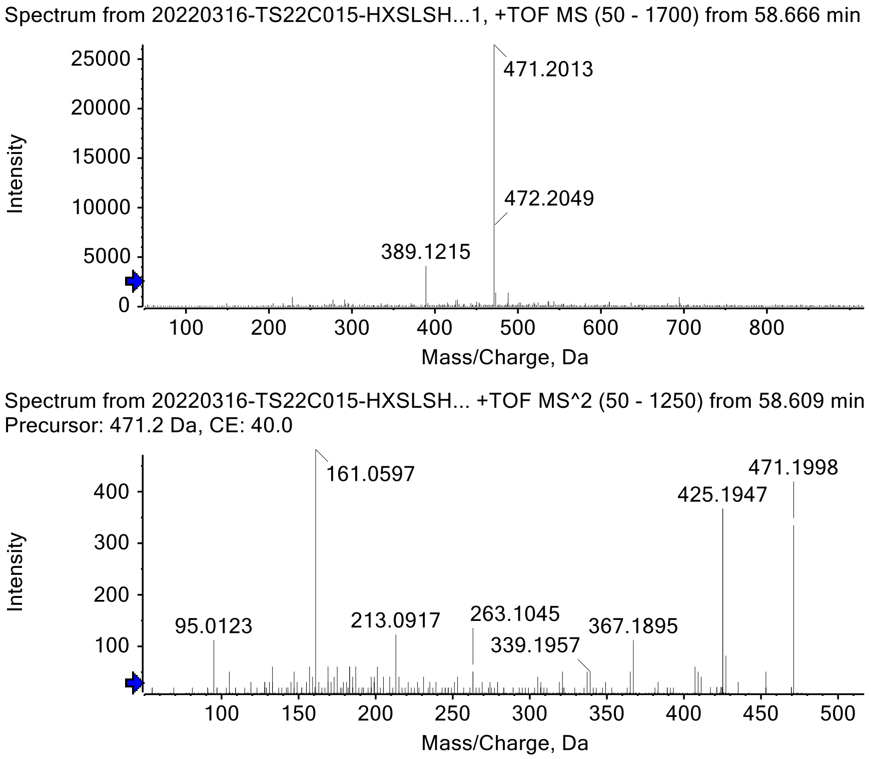

Figure S4-77: MS^{1}and MS^2 spectrograms of component 77 in HSSD

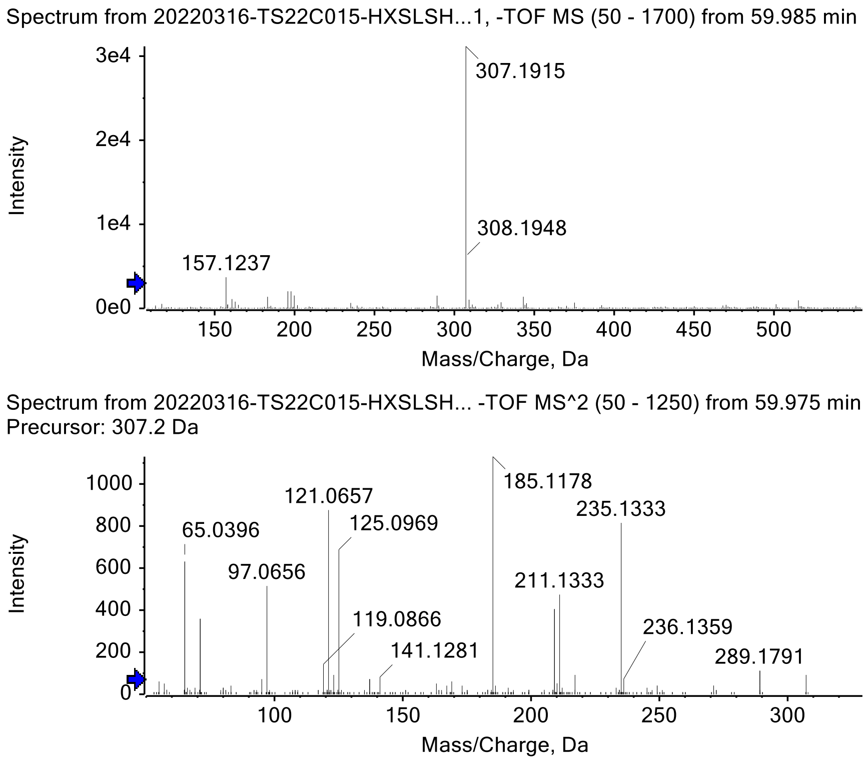

Figure S4-78: MS[1]and MS[2] spectrograms of component 78 in HSSD

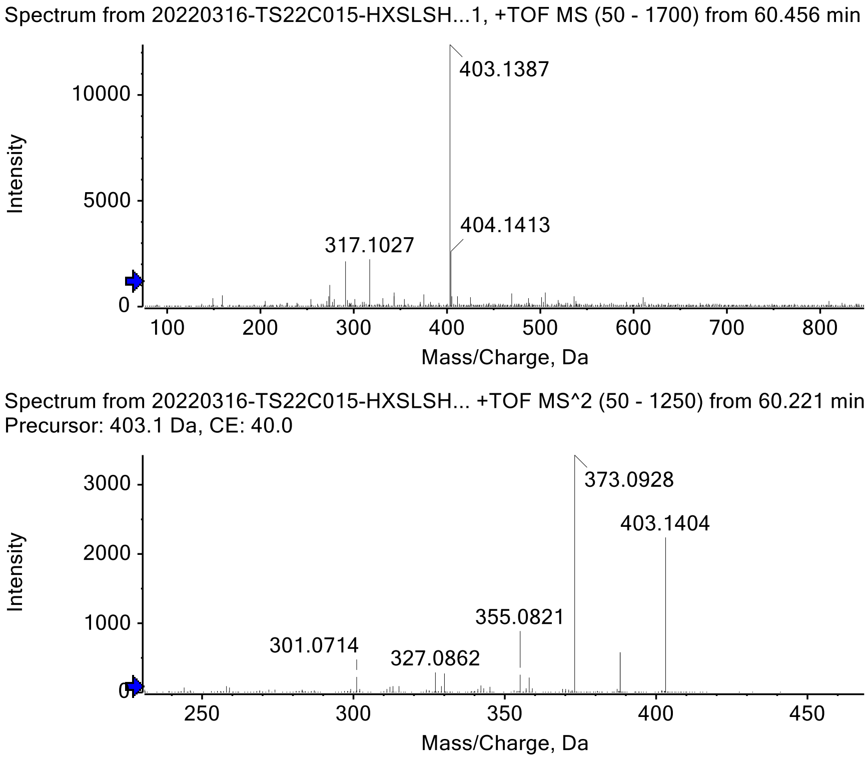

Figure S4-79: MS[1]and MS[2] spectrograms of component 79 in HSSD

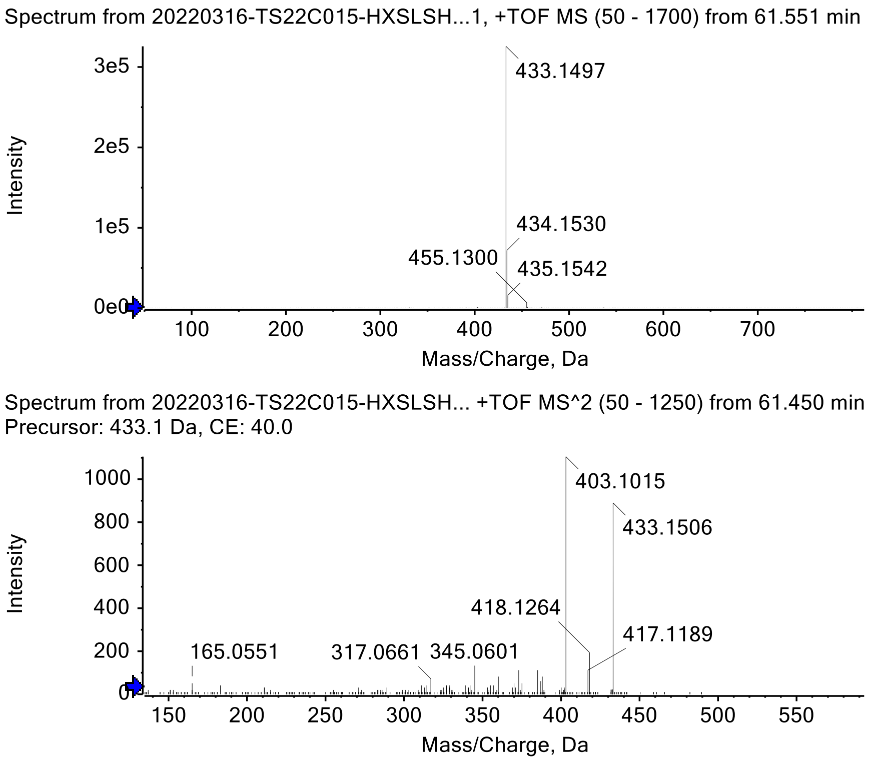

Figure S4-80: MS[1]and MS[2] spectrograms of component 80 in HSSD

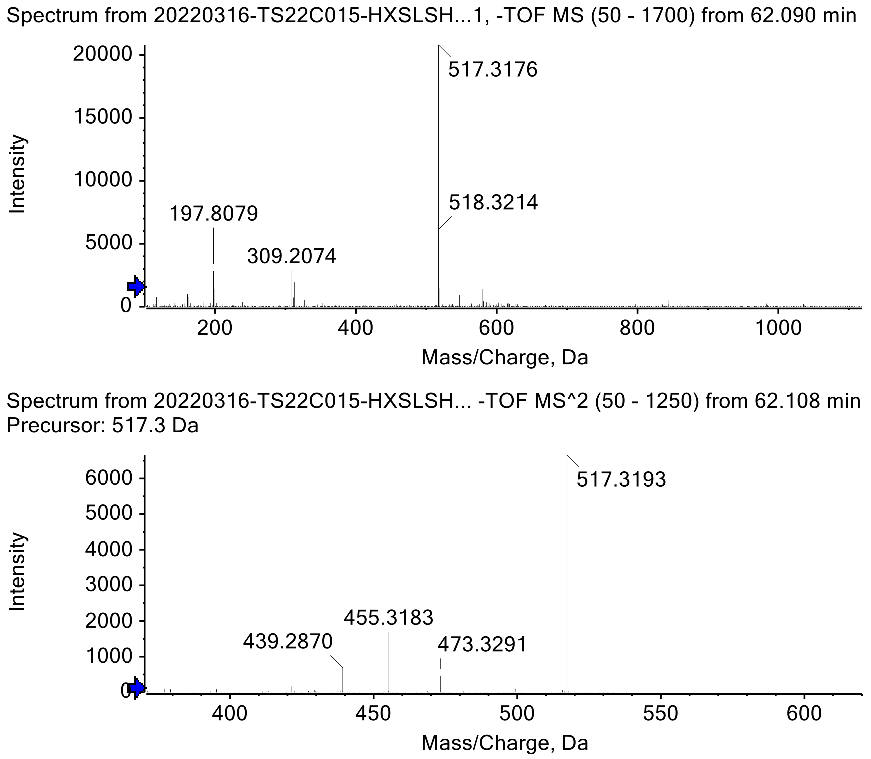

Figure S4-81: MS[1]and MS[2] spectrograms of component 81 in HSSD

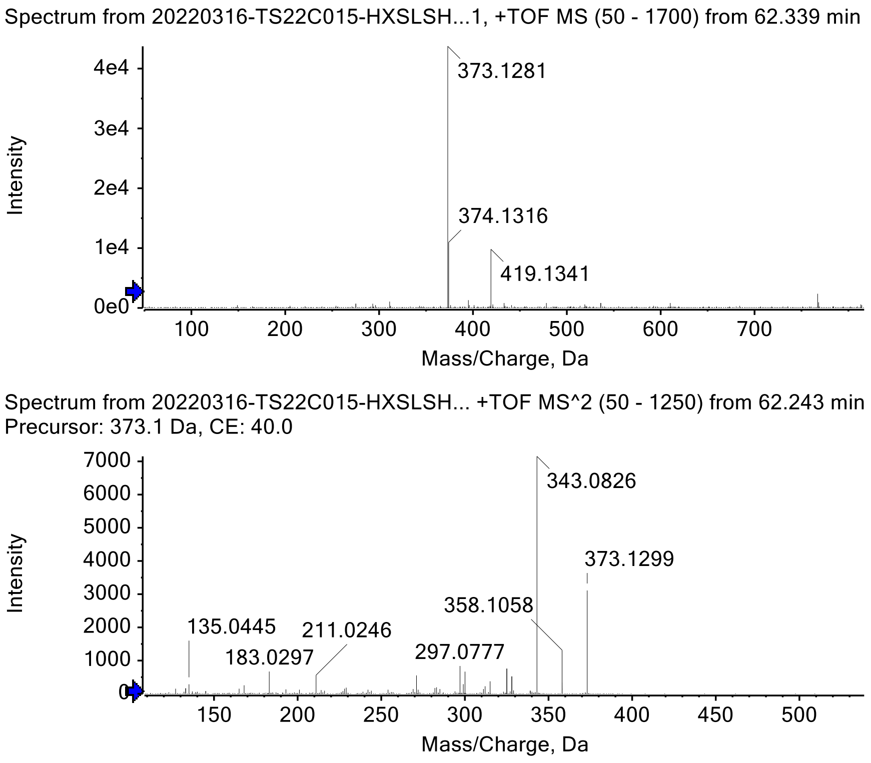

Figure S4-82: MS^{1}and MS^2 spectrograms of component 82 in HSSD

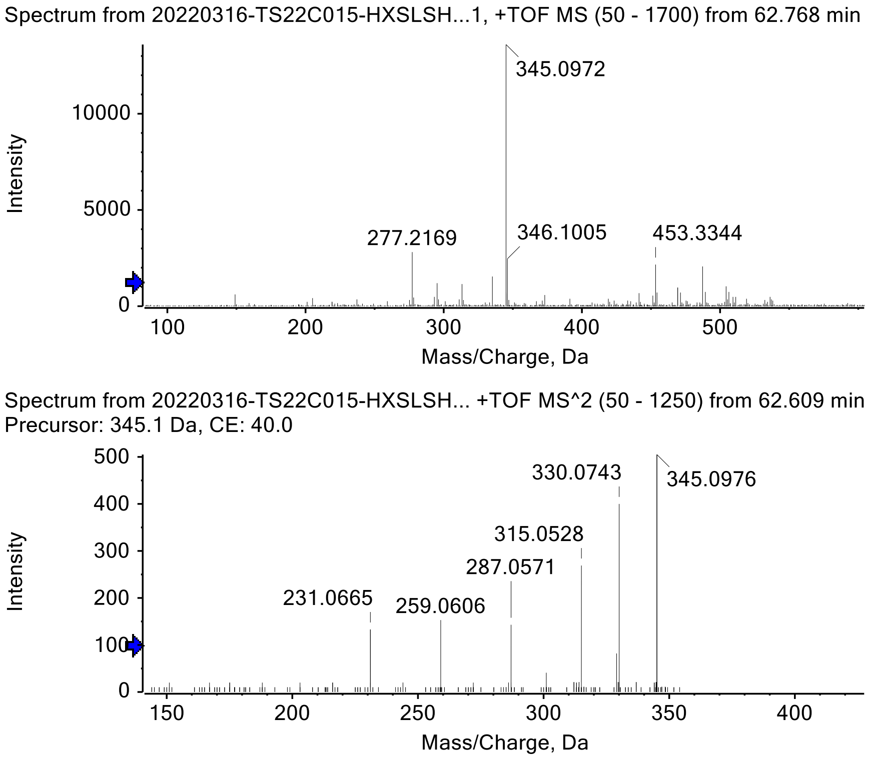

Figure S4-83: MS^{1}and MS^2 spectrograms of component 83 in HSSD

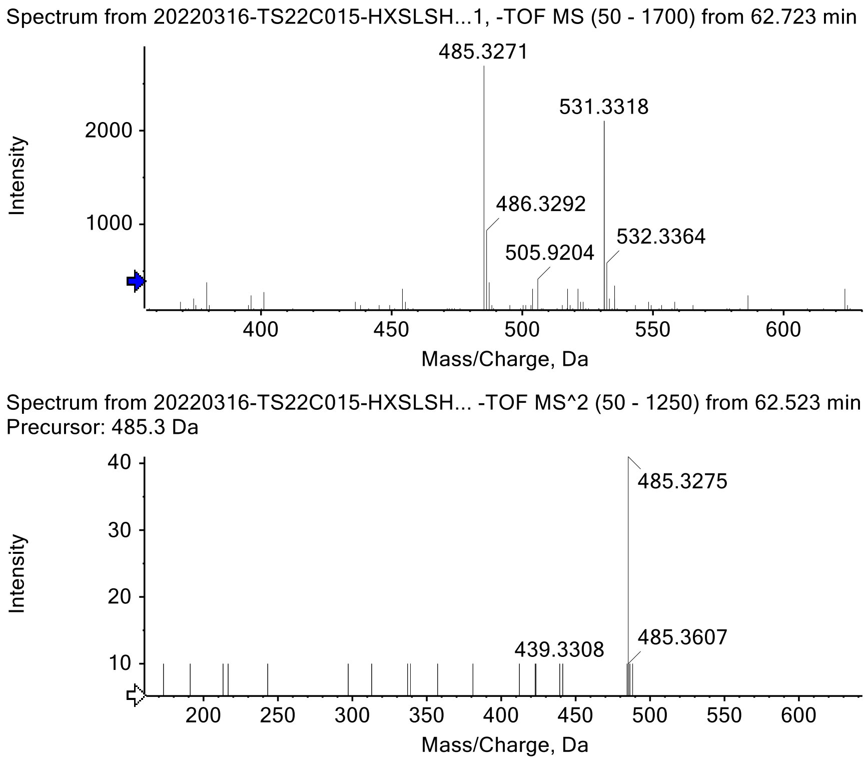

Figure S4-84: MS[1]and MS[2] spectrograms of component 84 in HSSD

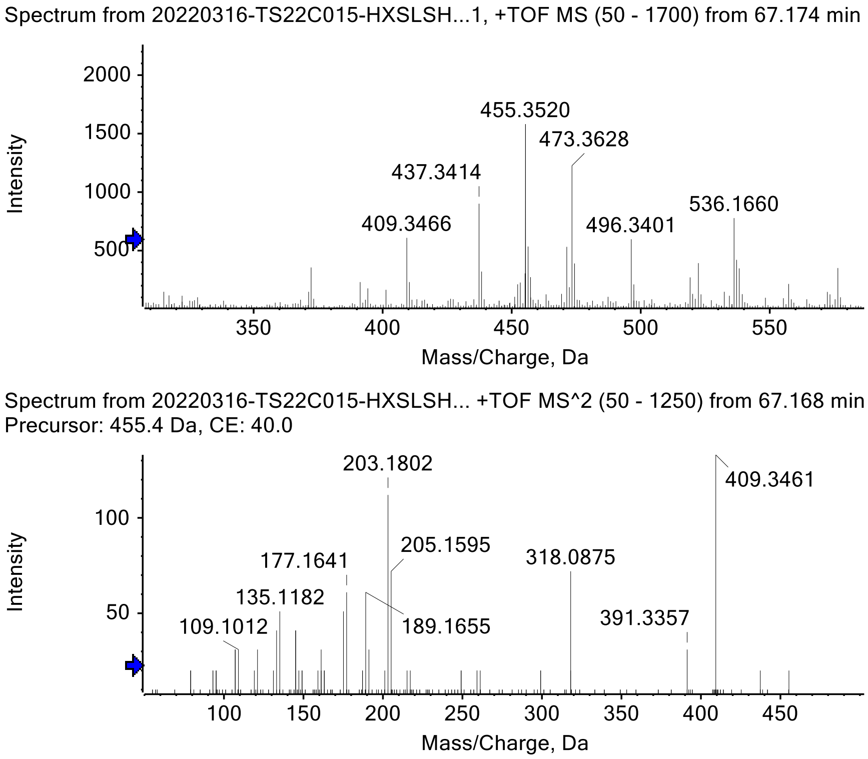

Figure S4-85: MS[1]and MS[2] spectrograms of component 85 in HSSD

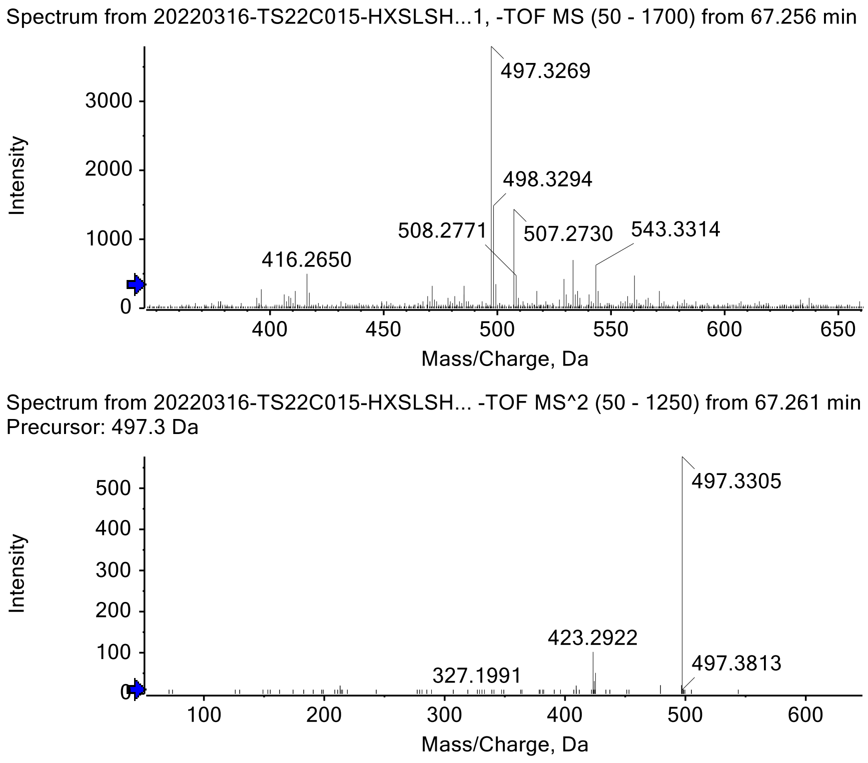

Figure S4-86: MS[1]and MS[2] spectrograms of component 86 in HSSD

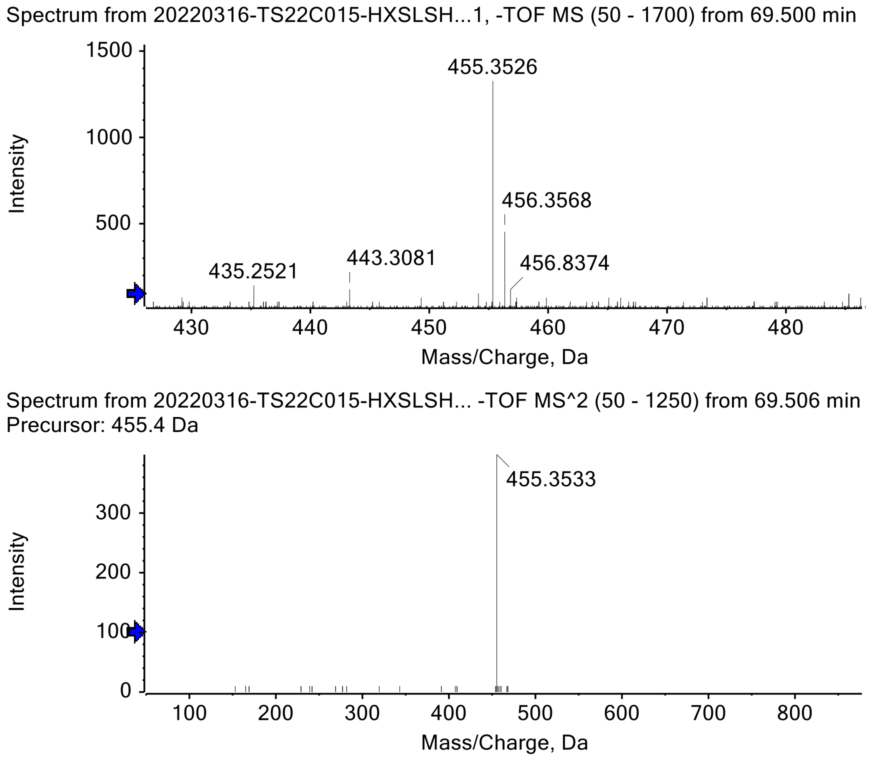

Figure S4-87: MS[1]and MS[2] spectrograms of component 87 in HSSD
