# Supplementary material for: Exploring the active ingredients and pharmacological mechanisms of the oral intake formula Huoxiang Suling Shuanghua Decoction on influenza virus type A based on network pharmacology and experimental exploration
Source: Front Microbiol. 2022 Nov 1;13:1040056. doi: 10.3389/fmicb.2022.1040056 (PMC9663660; doi:10.3389/fmicb.2022.1040056)
Supplement: Supplementary file 2 [file Data_Sheet_3.PDF]

Supplementary Data Sheet 3: Raw data of serum ingredients identification after HSSD orally in rat by UPLC/Q-TOF MS.

A

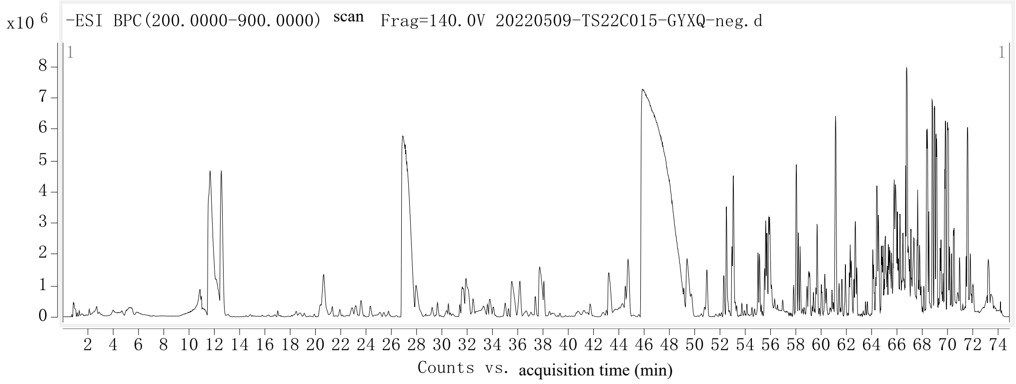

B

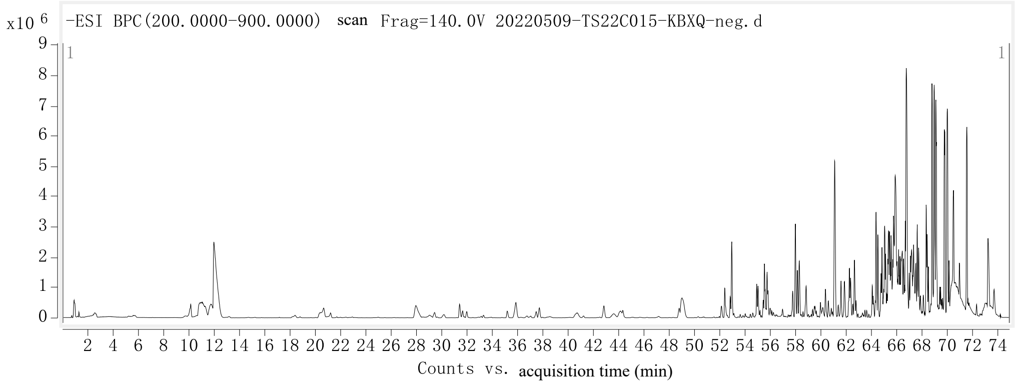

Figure S1 Negative ion mode of serum samples UPLC-HRMS base peak ion current diagram (BPC). A: diagram of serum sample intervention of HSSD. B: diagram of blank serum sample.

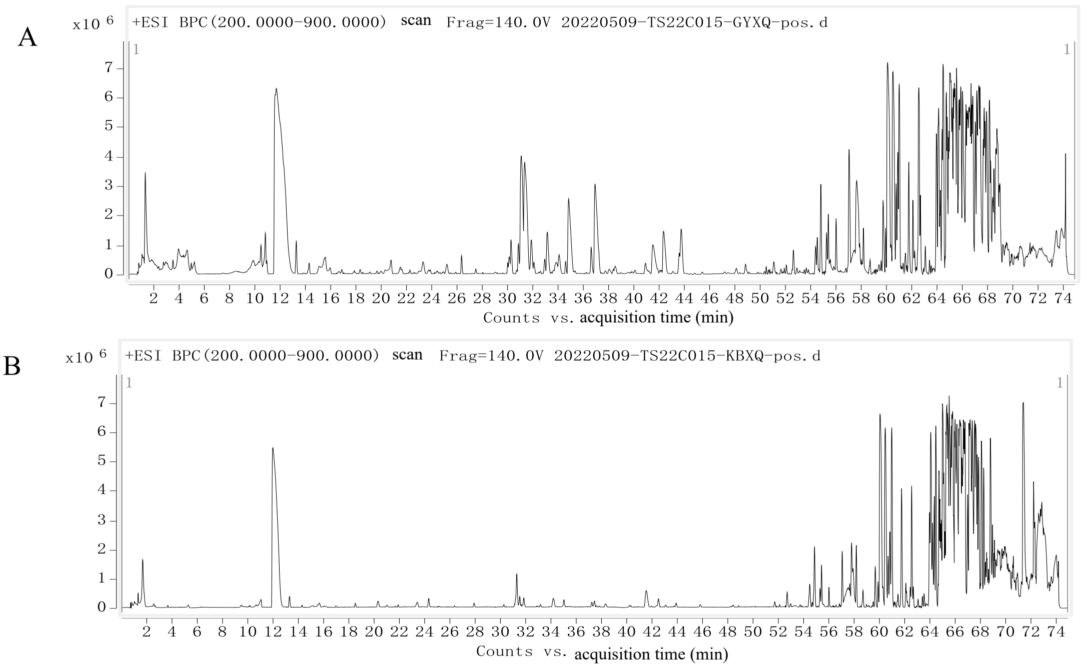

Figure S2 Positive ion mode of serum samples UPLC-HRMS base peak ion current diagram (BPC). A: diagram of serum sample intervention of HSSD. B: diagram of blank serum sample.

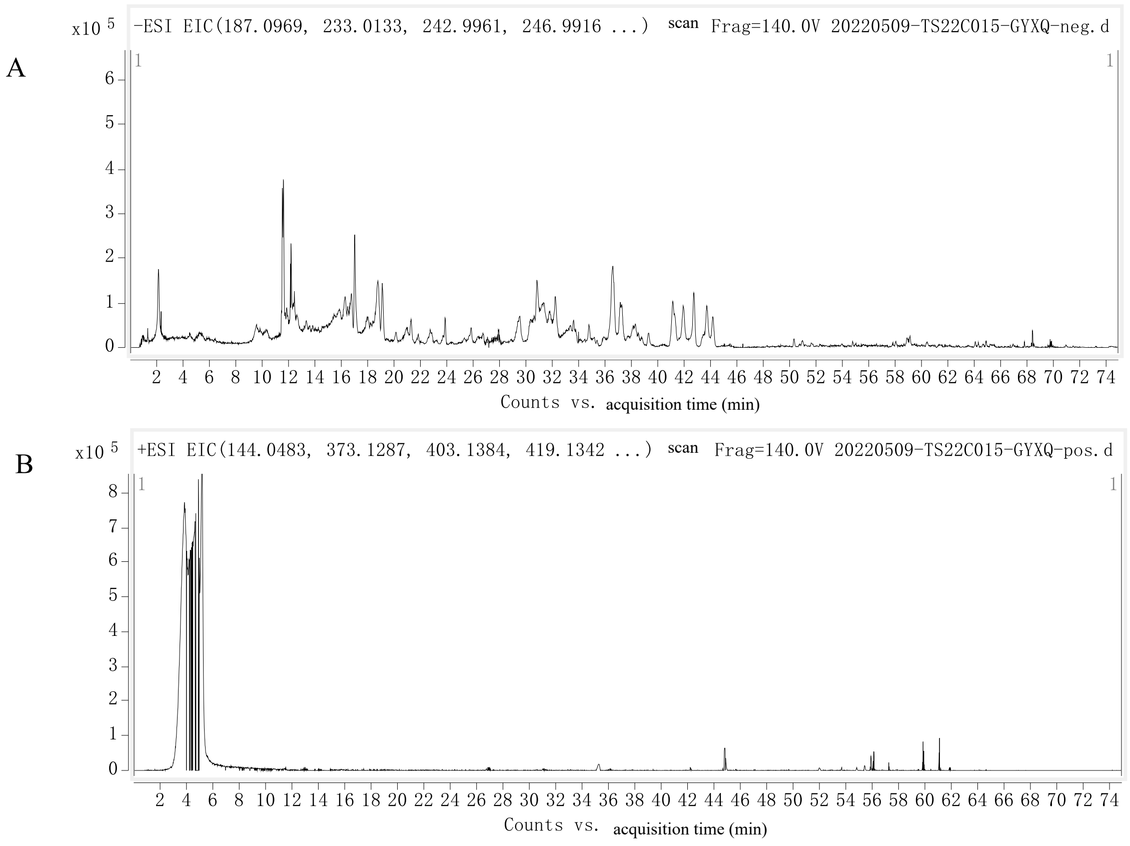

Figure S3 UPLC-HRMS Extraction of Plasma Flow Map (EIC)

A: Negative ion mode. B: Positive ion mode.

Figure S4 Grade one mass spectrograms of 20 incoming blood components of HSSD as followings:

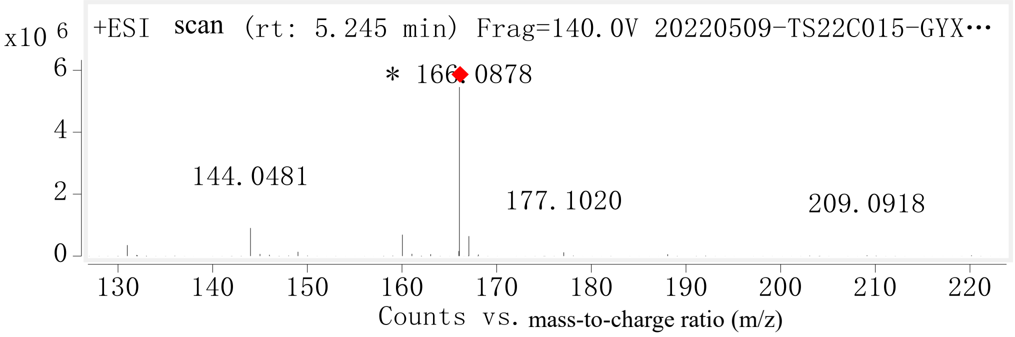

Figure S4-1: Grade one mass spectrograms of incoming blood component 1 of HSSD

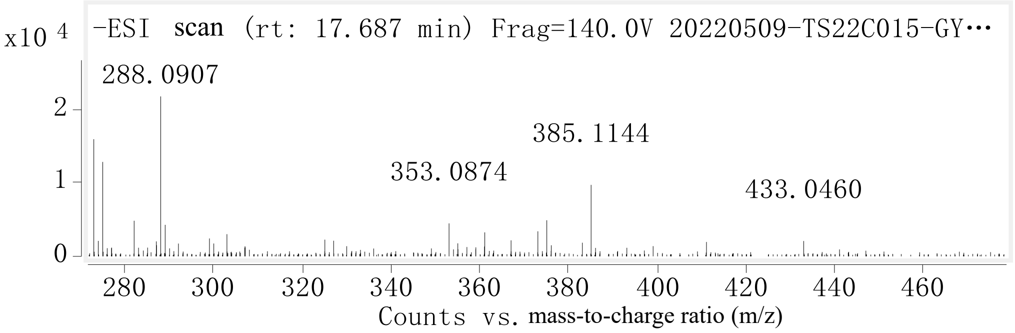

Figure S4-2: Grade one mass spectrograms of incoming blood component 2 of HSSD

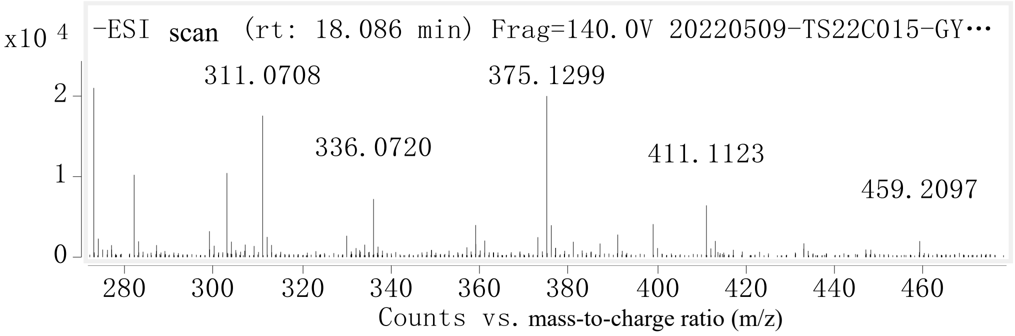

Figure S4-3: Grade one mass spectrograms of incoming blood component 3 of HSSD

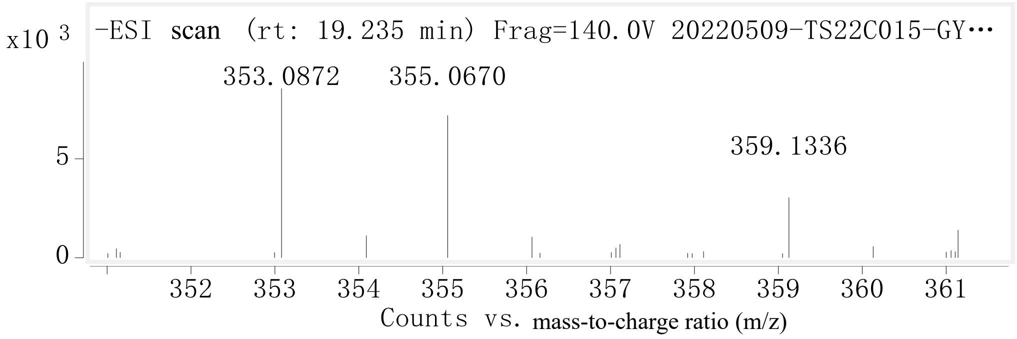

Figure S4-4: Grade one mass spectrograms of incoming blood component 4 of HSSD

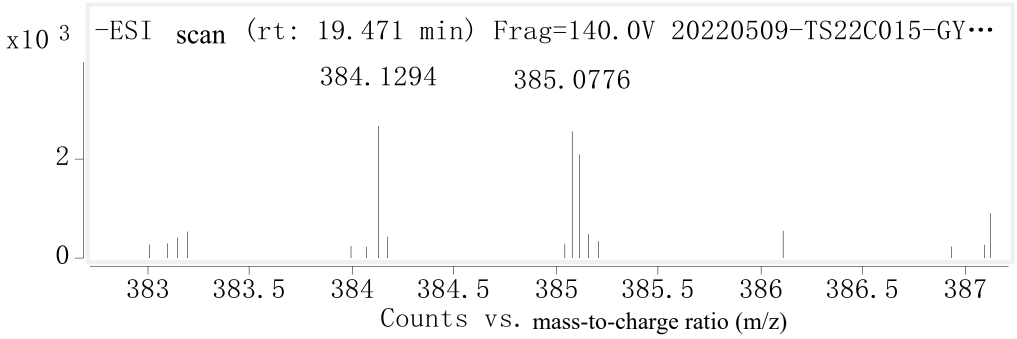

Figure S4-5: Grade one mass spectrograms of incoming blood component 5 of HSSD

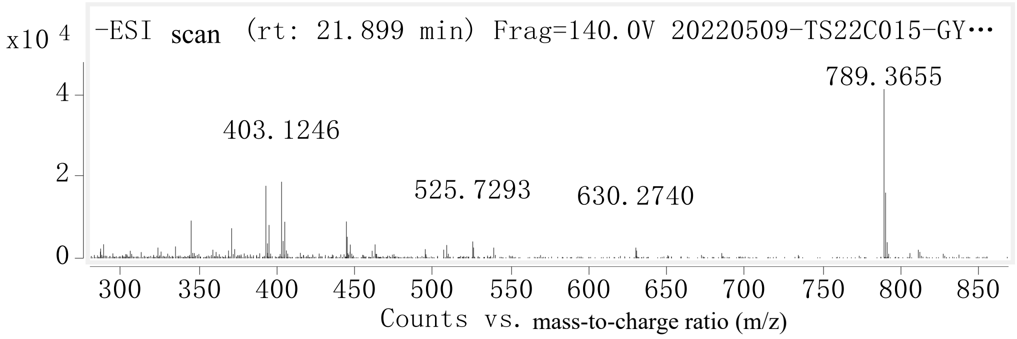

Figure S4-6: Grade one mass spectrograms of incoming blood component 6 of HSSD

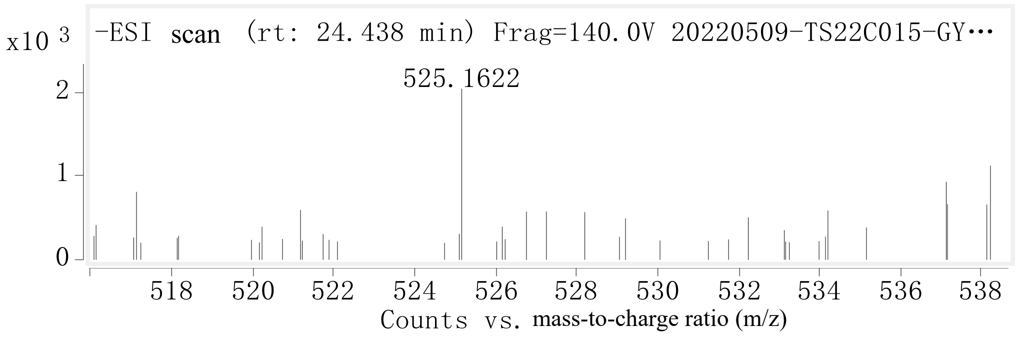

Figure S4-7: Grade one mass spectrograms of incoming blood component 7 of HSSD

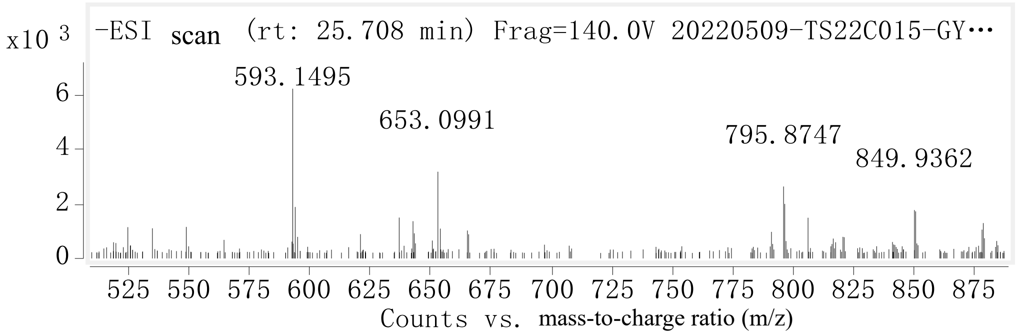

Figure S4-8: Grade one mass spectrograms of incoming blood component 8 of HSSD

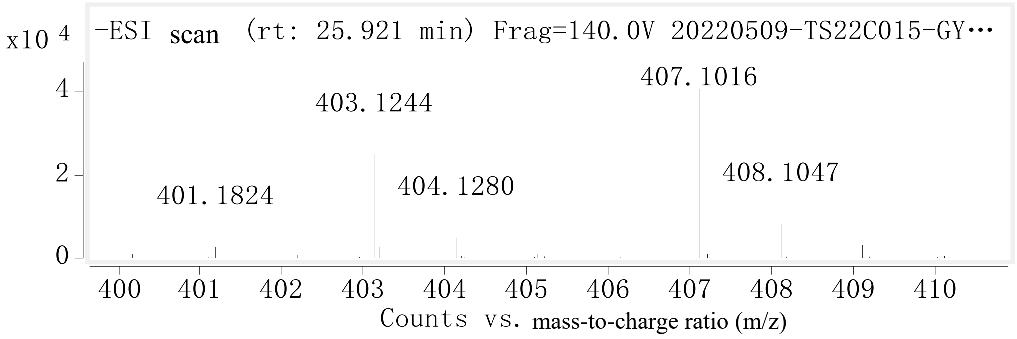

Figure S4-9: Grade one mass spectrograms of incoming blood component 9 of HSSD

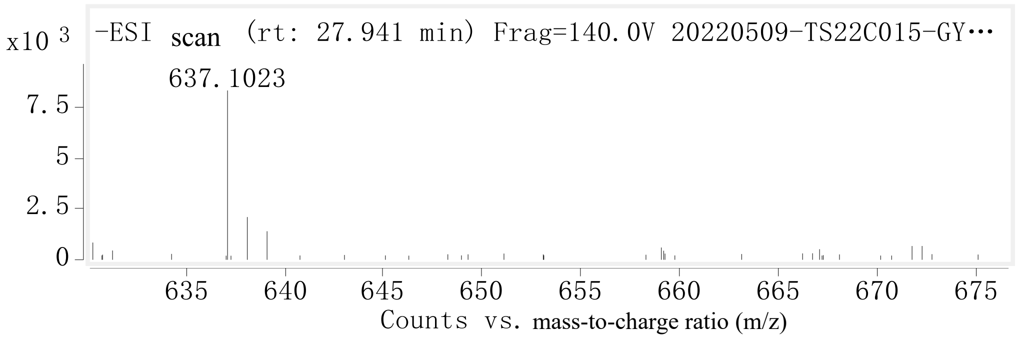

Figure S4-10: Grade one mass spectrograms of incoming blood component 10 of HSSD

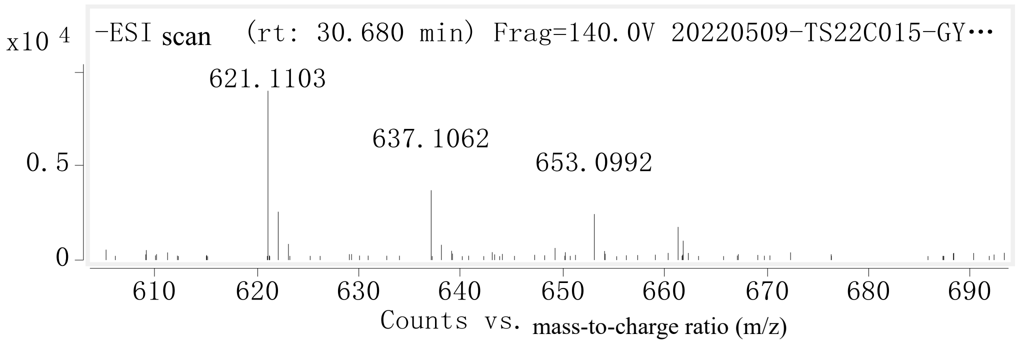

Figure S4-11: Grade one mass spectrograms of incoming blood component 11 of HSSD

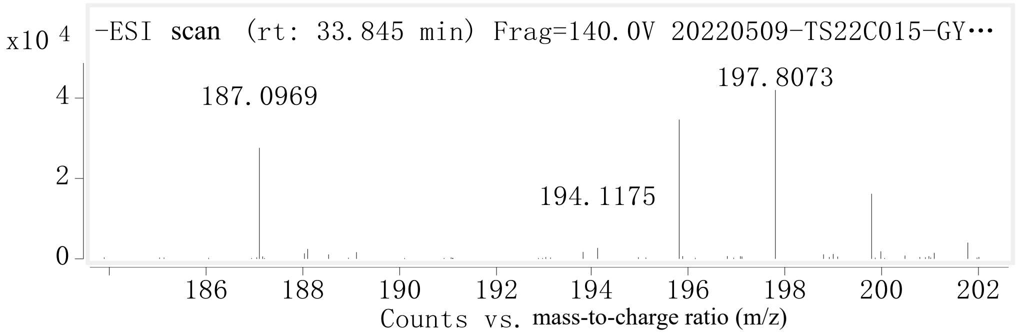

Figure S4-12: Grade one mass spectrograms of incoming blood component 12 of HSSD

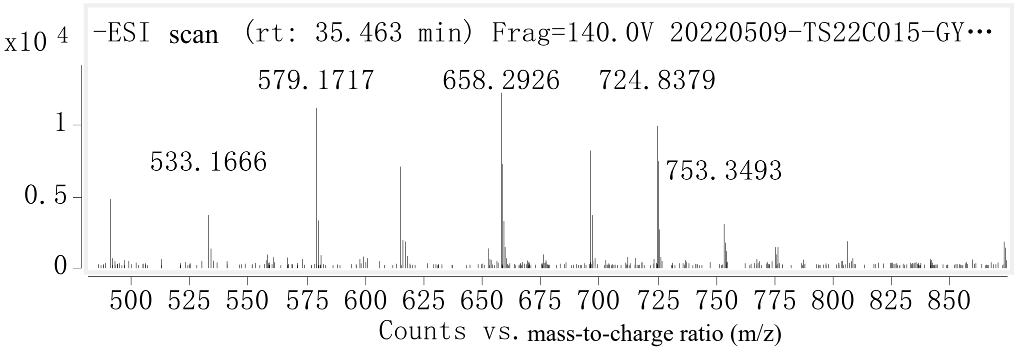

Figure S4-13: Grade one mass spectrograms of incoming blood component 13 of HSSD

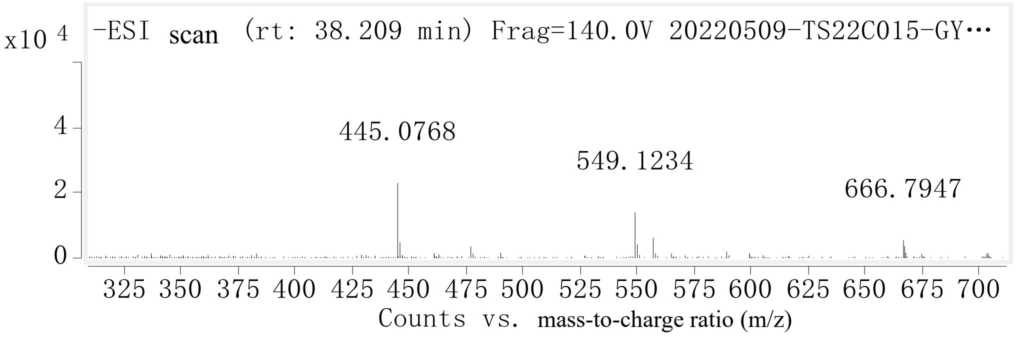

Figure S4-14: Grade one mass spectrograms of incoming blood component 14 of HSSD

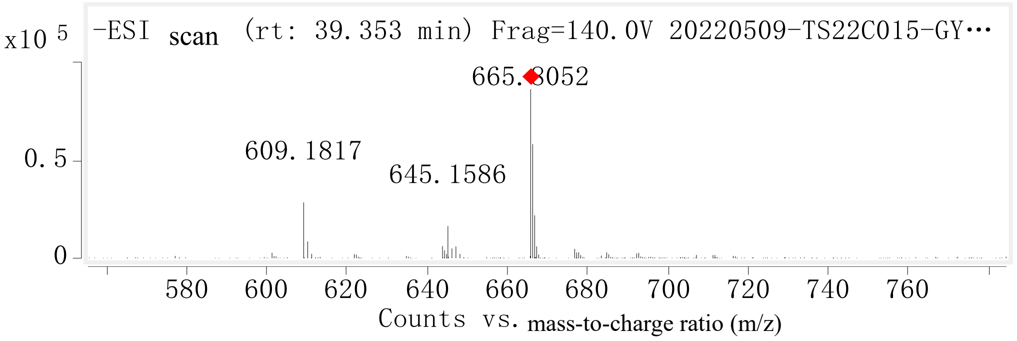

Figure S4-15: Grade one mass spectrograms of incoming blood component 15 of HSSD

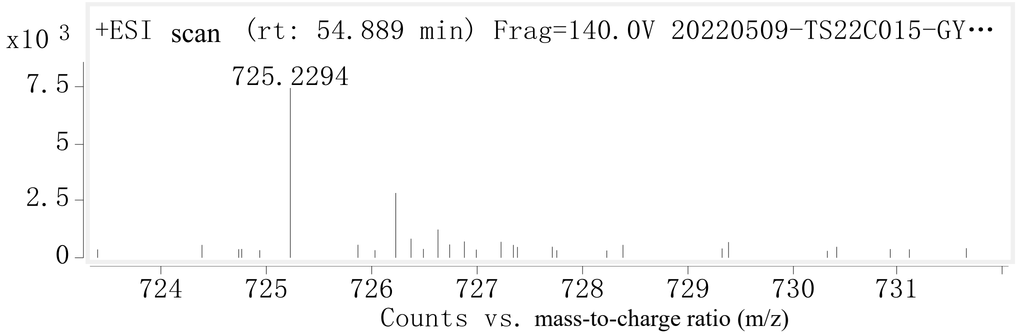

Figure S4-16: Grade one mass spectrograms of incoming blood component 16 of HSSD

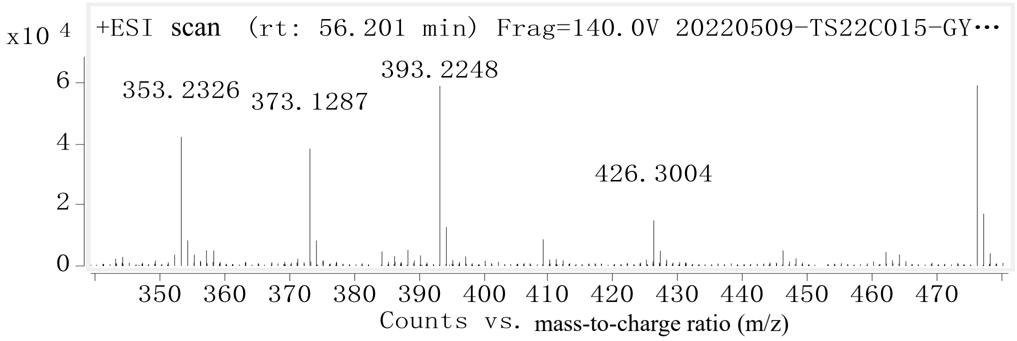

Figure S4-17: Grade one mass spectrograms of incoming blood component 17 of HSSD

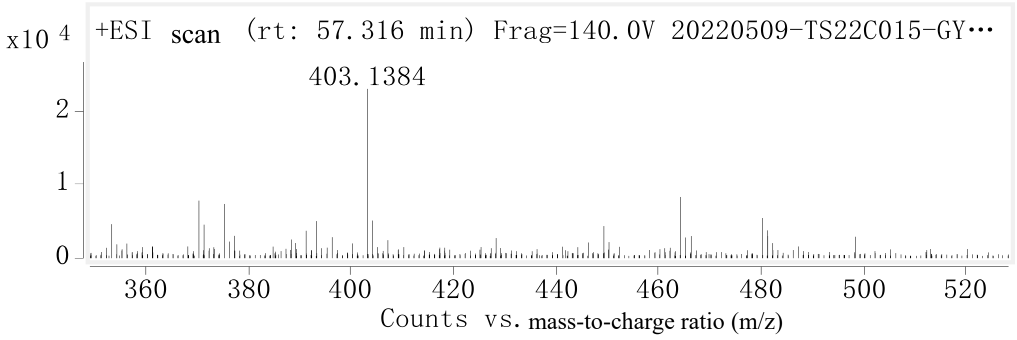

Figure S4-18: Grade one mass spectrograms of incoming blood component 18 of HSSD

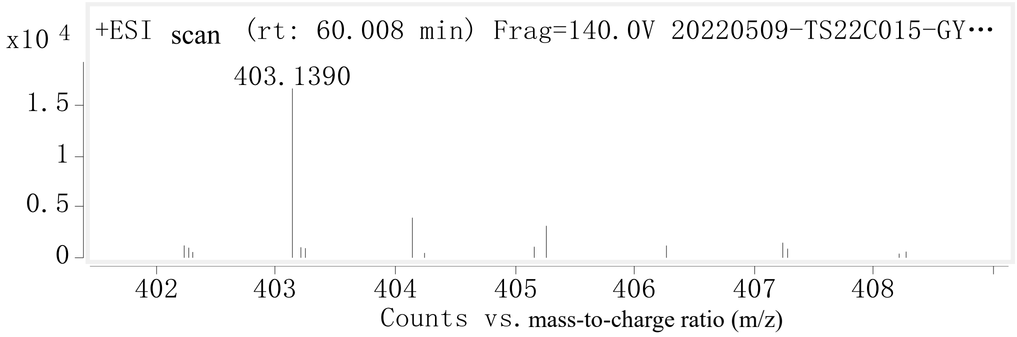

Figure S4-19: Grade one mass spectrograms of incoming blood component 19 of HSSD

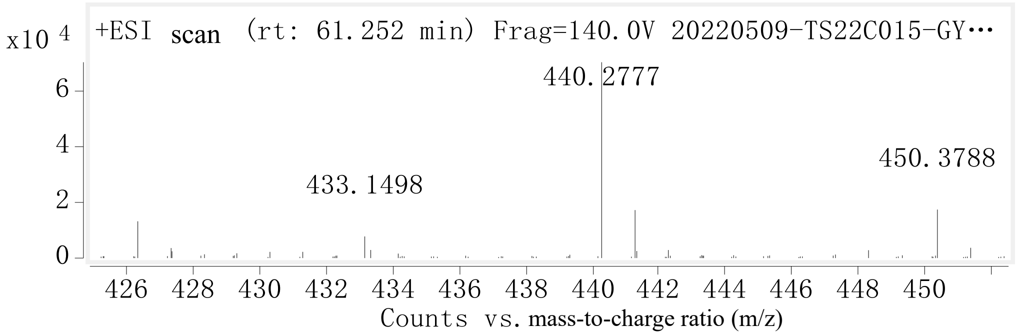

Figure S4-20: Grade one mass spectrograms of incoming blood component 20 of HSSD
