# Supplementary material for: Exploring the active ingredients and pharmacological mechanisms of the oral intake formula Huoxiang Suling Shuanghua Decoction on influenza virus type A based on network pharmacology and experimental exploration
Source: Front Microbiol. 2022 Nov 1;13:1040056. doi: 10.3389/fmicb.2022.1040056 (PMC9663660; doi:10.3389/fmicb.2022.1040056)
Supplement: Supplementary file 3 [file Data_Sheet_4.PDF]

Supplementary Data Sheet 4: Detail information of the network of “herb-compounds-targets” of HSSD.

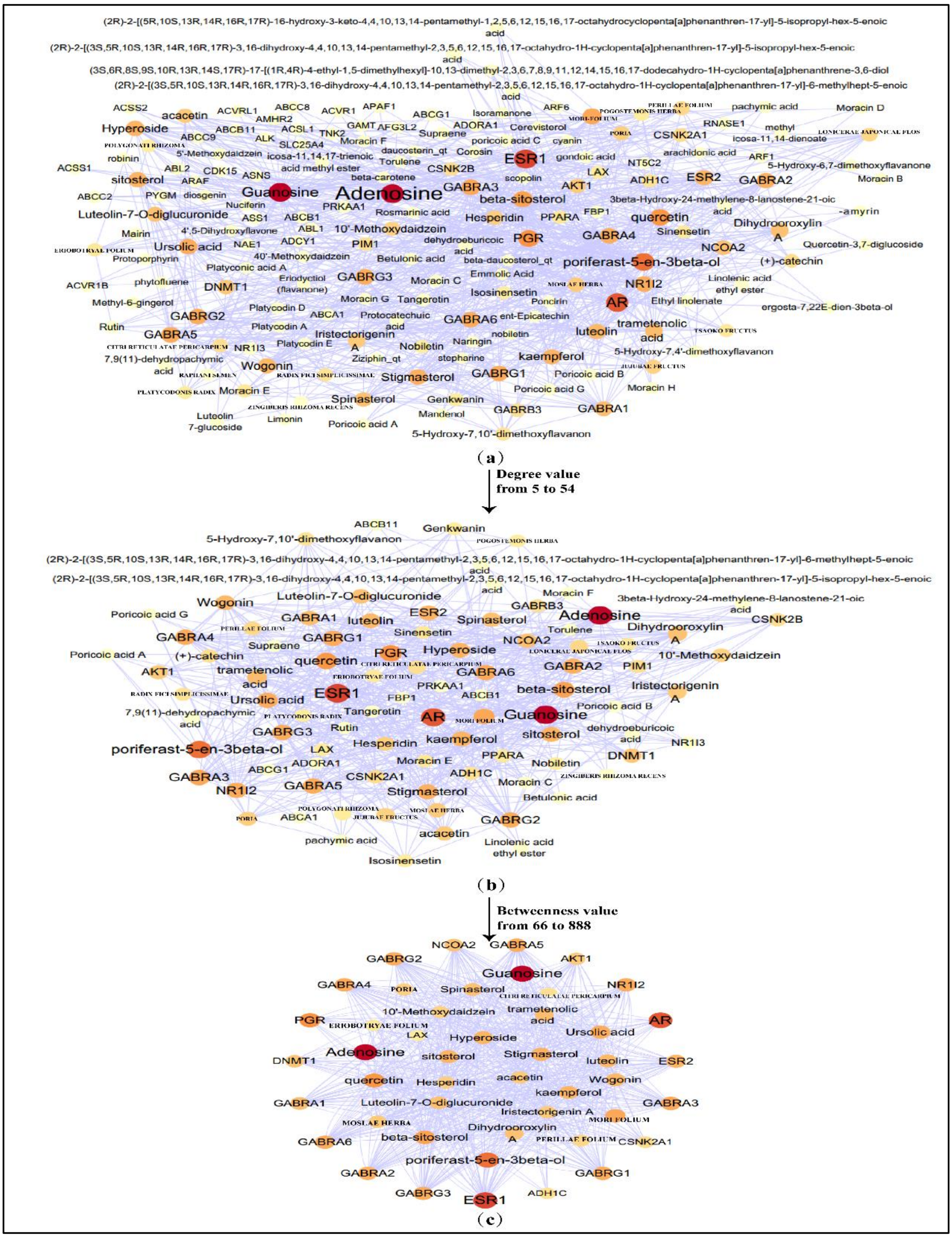

Figure S1: “herb-compounds-targets” network topology analysis. (a) “herb-compounds-targets” network topology analysis results figure with 171 nodes and 814 edges; (b) “herb-compounds-targets” network topology analysis results figure setting degree range from 5 to 54 based on (a) with 90 nodes and 583 edges; (c) “herb-compounds-targets” network topology analysis results figure setting betweenness range from 66 to 888 based on (b) with 46 nodes and 626 edges.

Note: The larger the circle, the darker the color and the larger the font size, representing the more important the node is in this network graph.

Table S1 Critical compounds and targets information in the network of "herb-compounds-targets" of HSSD.

**Critical compounds**	**Degree**	**Betweenness**	**Critical targets**	**Degree**	**Betweenness**
Guanosine	54	607.02	ESR1	43	887.55
Adenosine	53	697.87	AR	40	429.54
Poriferast-5-en-3beta-ol	36	209.20	PGR	30	409.89
Quercetin	29	432.23	GABRA3	25	162.67
Beta-sitosterol	26	408.26	NR1I2	24	273.99
Ursolic acid	24	308.09	GABRG3	24	162.67
Stigmasterol	23	318.89	GABRG2	24	162.67
Sitosterol	22	233.04	GABRG1	24	162.67
Kaempferol	22	168.64	GABRA6	24	162.67
Hyperoside	22	202.26	GABRA5	24	162.67
Wogonin	21	155.25	GABRA4	24	162.67
Luteolin	21	134.29	GABRA2	23	142.71
Trametenolic acid	20	249.38	ESR2	22	105.02
Spinasterol	19	186.54	GABRA1	20	92.07
Luteolin-7-O-diglucuronide	19	117.50	NCOA2	19	96.10
Dihydrooroxylin A	19	99.01	DNMT1	18	80.15
Iristectorigenin A	17	86.70	ADH1C	11	116.12
Acacetin	17	103.48	LAX	9	76.44
Hesperidin	16	77.57	CSNK2A1	13	63.96
10'-Methoxydaidzein	15	66.70	AKT1	17	96.21
