# Supplementary material for: Exploring the active ingredients and pharmacological mechanisms of the oral intake formula Huoxiang Suling Shuanghua Decoction on influenza virus type A based on network pharmacology and experimental exploration
Source: Front Microbiol. 2022 Nov 1;13:1040056. doi: 10.3389/fmicb.2022.1040056 (PMC9663660; doi:10.3389/fmicb.2022.1040056)
Supplement: Supplementary file 7 [file Data_Sheet_8.PDF]

Supplementary Data Sheet 8: Results explanation of BP analysis, CC analysis, MF analysis and KEGG pathways.

GO enrichment analysis results were shown in **Figure S1**. In **Figure S1 A**, the "intersected common targets of HSSD & influenza" enrichment analysis of GO terms were performed with 1049 BPs, 146 CCs, and 204 MFs. According to the top 5 GO enrichment analysis, the gene targets associated with BP are embroiled in positive regulation of gene expression, negative regulation of apoptotic process, response to drug, inflammatory response, and positive regulation of transcription from RNA polymerase II promoter, etc. Gene targets in CC are primarily found in cytosol, macromolecular complex, extracellular exosome, cytoplasm, and extracellular space, etc. While MF are mainly revealed in identical protein binding, enzyme binding, protein binding, macromolecular complex binding, and ubiquitin protein ligase binding, etc. Conclusion, the "intersected common targets of HSSD & influenza" enrichment analysis of GO terms suggested that HSSD treating influenza mainly revealed in the regulation of enzyme activity, protein binding, apoptosis, cytokines and inflammatory response processes, etc.

While in **Figure S1 B**, the "intersected common targets of influenza" enrichment analysis of GO terms were performed with 305 BPs, 23 CCs, and 42 MFs. According to the top 5 GO enrichment analysis, the gene targets associated with BP are embroiled in cellular response to lipopolysaccharide, response to virus, inflammatory response, defense response to virus, and positive regulation of smooth muscle cell proliferation, etc. Gene targets in CC are primarily found in extracellular region, extracellular space, cytoplasm, cell surface, and macromolecular complex, etc. While MF are mainly revealed in cytokine activity, protein binding, identical protein binding, transcription regulatory region sequence-specific DNA binding, and enzyme binding, etc. Conclusion, the "intersected common targets of influenza" enrichment analysis of GO terms suggested that influenza mainly revealed in virus, inflammatory response, and regulation of cytokine activity, protein binding, etc.

After intersecting "intersected common targets of HSSD & influenza" GO results and "intersected common targets of influenza" GO results, critical top 10 GO enrichment results and its networks were obtained in **Figure S1 C**. According to the critical GO enrichment analysis, the gene targets associated with BP are embroiled in positive or negative regulation of gene expression, response to drug, inflammatory response, and positive regulation of transcription from RNA polymerase II promoter, etc. Gene targets in CC are primarily found in cytosol, macromolecular complex, cytoplasm, extracellular space, and nucleoplasm, etc. While MF are mainly revealed in enzyme binding, identical protein binding, protein binding, ubiquitin protein ligase binding, and protease binding, etc. In conclusion, after intersecting "intersected common targets of HSSD & influenza" GO results and "intersected common targets of influenza" GO results, the critical top 10 GO enrichment demonstrated that HSSD treating influenza mainly revealed in the regulation of drug, inflammatory response, and the regulation of

apoptosis, cytokines, protein binding, etc.

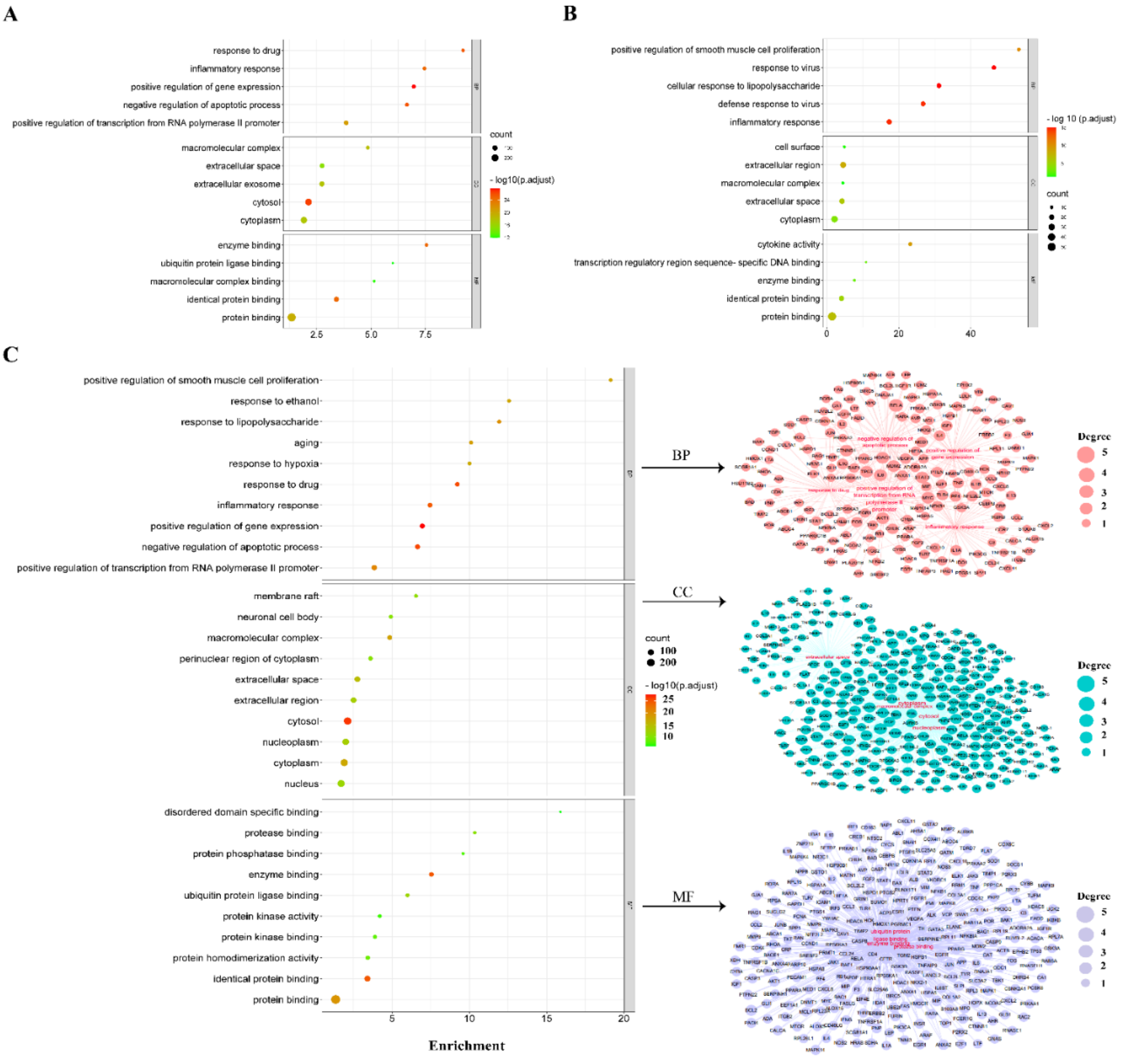

FIGURE S1

GO enrichment analysis. **(A)** GO enrichment results of "intersected common targets of HSSD & influenza". **(B)** GO enrichment results of "intersected common targets of influenza". **(C)** Critical GO enrichment results getting by intersecting "intersected common targets of HSSD & influenza" GO results and "intersected common targets of influenza" GO results.

KEGG enrichment analysis results were shown in **Figure S2**. In **Figure S2 A**, the "intersected common targets of HSSD & influenza" enrichment analysis of 186 KEGG results were performed. The top 10 KEGG pathways mainly revealed in Lipid and atherosclerosis, Pathways in cancer, Kaposi sarcoma-associated herpesvirus infection, AGE-RAGE signaling pathway in diabetic complications, Hepatitis B, Fluid shear stress and atherosclerosis, IL-17 signaling pathway, Measles, Human cytomegalovirus infection, and Hepatitis C, etc. Conclusion, the "intersected common targets of HSSD & influenza" enrichment analysis of KEGG suggested that HSSD treating influenza mainly revealed in the regulation of virus related signaling pathways, and IL-17 signaling pathway, etc.

While in **Figure S2 B**, the “intersected common targets of influenza” enrichment analysis of 96 KEGG results were performed. The top 10 KEGG pathways mainly revealed in Influenza A, Coronavirus disease-COVID-19, Lipid and atherosclerosis, Measles, Chagas disease, Inflammatory bowel disease, Pertussis, Yersinia infection, IL-17 signaling pathway, and Hepatitis C, etc. Conclusion, the “intersected common targets of influenza” enrichment analysis of KEGG suggested that influenza mainly revealed in different virus (including influenza A and Coronavirus disease-COVID-19) related signaling pathways, and IL-17 signaling pathway, etc.

After intersecting “intersected common targets of HSSD & influenza” KEGG results and “intersected common targets of influenza” KEGG results, critical top 10 KEGG signaling pathways and its networks were obtained in **Figure S2 C**. The critical top 10 KEGG signaling pathways mainly revealed in IL-17 signaling pathway, TNF signaling pathway, PI3K-Akt signaling pathway, Toll-like receptor signaling pathway, FoxO signaling pathway, C-type lectin receptor signaling pathway, HIF-1 signaling pathway, MAPK signaling pathway, T cell receptor signaling pathway, and NOD-like receptor signaling pathway, etc. In conclusion, after intersecting “intersected common targets of HSSD & influenza” GO results and “intersected common targets of influenza” KEGG results, critical top 10 KEGG signaling pathways mainly revealed in the regulation of IL-17, TNF, PI3K-Ak, and Toll-like receptor signaling pathway, and apoptosis, chemokines, influenza A and other signaling pathways, etc.

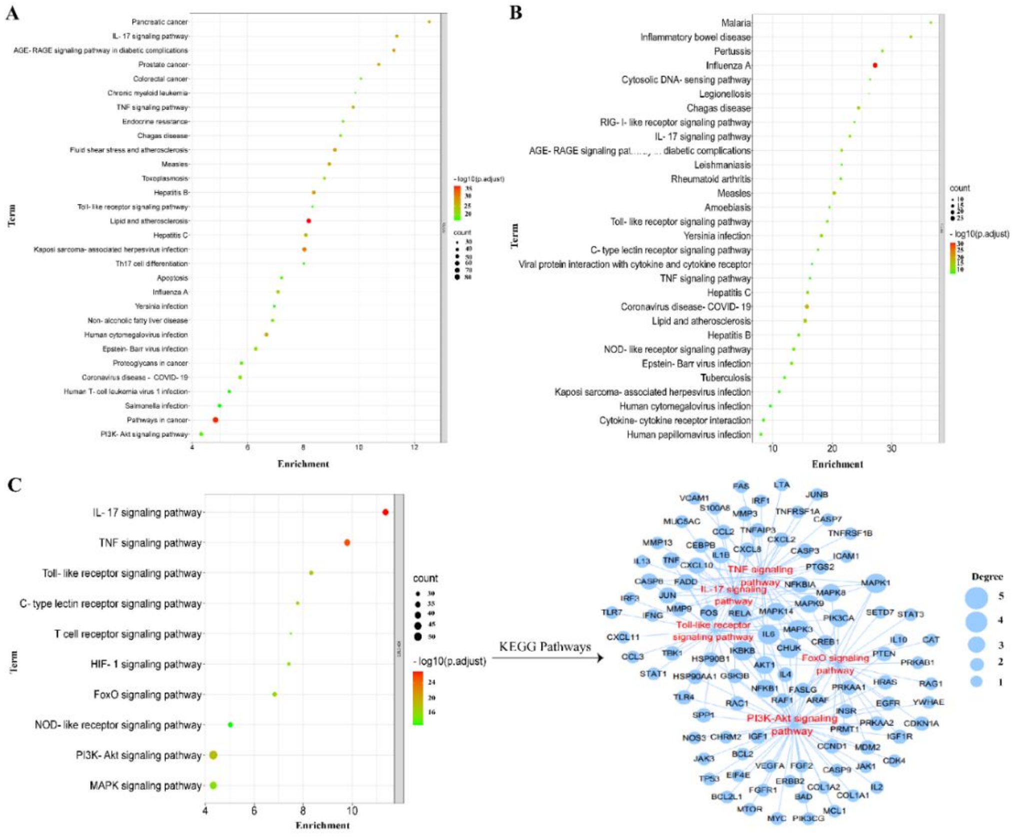

FIGURE S2

KEGG enrichment analysis. **(A)** KEGG enrichment results of “intersected common targets of HSSD & influenza”. **(B)** KEGG enrichment results of “intersected common targets of influenza”. **(C)** Critical KEGG enrichment signaling pathways results getting by intersecting “intersected common targets of HSSD & influenza” KEGG results and “intersected common targets of influenza” KEGG results.

In brief, GO and KEGG enrichment analysis results suggested us that HSSD treating influenza mainly through combining multiple components with multiple influenza virus proteins, acting on multiple targets and regulating multiple pathways, which including inflammatory response, immune response, cell apoptosis and influenza virus replication, as well as the IL-17, TNF, PI3K-Ak, and Toll-like receptor signaling pathways to achieve the effects of treatment of influenza. Critical signaling pathways results such as IL-17 or Toll-like receptor signaling pathway of network pharmacology results provided us the detection direction of next mice experimental validation of HSSD in treating influenza.
