# Supplementary material for: Exploring the active ingredients and pharmacological mechanisms of the oral intake formula Huoxiang Suling Shuanghua Decoction on influenza virus type A based on network pharmacology and experimental exploration
Source: Front Microbiol. 2022 Nov 1;13:1040056. doi: 10.3389/fmicb.2022.1040056 (PMC9663660; doi:10.3389/fmicb.2022.1040056)
Supplement: Supplementary file 8 [file Data_Sheet_9.PDF]

Supplementary Data Sheet 9: The stable combining mode between 16 core targets and active compounds.

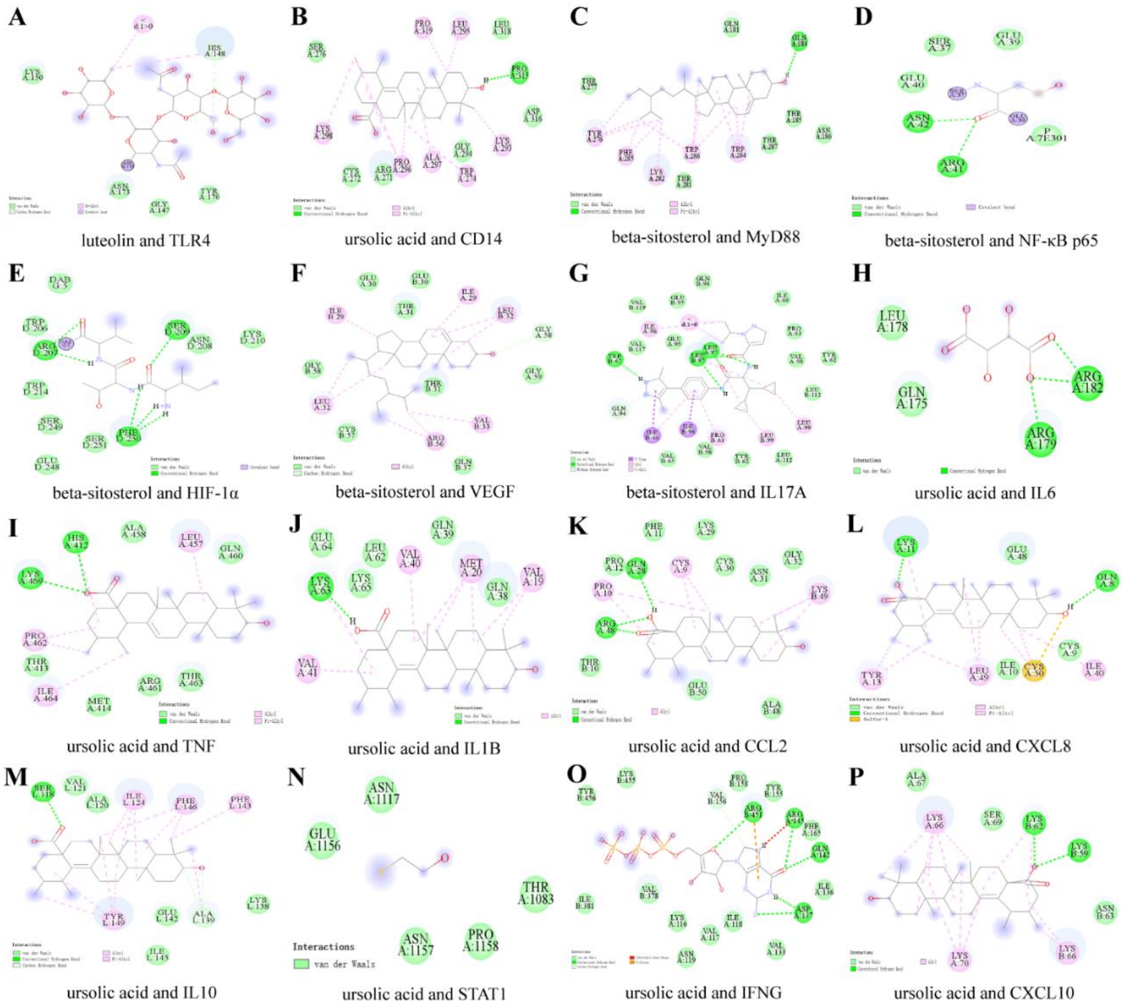

Figure S1

Molecular docking of critical compounds and critical targets. Binding of **(A)** luteolin and TLR4 (PDB ID: 2z62), **(B)** ursolic acid and CD14 (PDB ID: 4glp), **(C)** beta-sitosterol and MyD88 (PDB ID: 4dom), **(D)** beta-sitosterol and NF-κB p65 (PDB ID: 6ypy), **(E)** beta-sitosterol and HIF-1α(PDB ID: 7rna), **(F)** beta-sitosterol and VEGF (PDB ID: 1mjv), **(G)** beta-sitosterol and IL17A (PDB ID: 7ama), **(H)** ursolic acid and IL6 (PDB ID: 1alu), **(I)** ursolic acid and TNF (PDB ID: 1lb4), **(J)** ursolic acid and IL1B (PDB ID: 1twm), **(K)** ursolic acid and CCL2 (PDB ID: 7so0), **(L)** ursolic acid and CXCL8 (PDB ID: 4xdx), **(M)** ursolic acid and IL10 (PDB ID: 1y6m), **(N)** ursolic acid and STAT1 (PDB ID: 3cm3), **(O)** ursolic acid and IFNG (PDB ID: 4q7h), **(P)** ursolic acid and CXCL10 (PDB ID: 1o80). Different colors represent the different interactions between the critical compounds and the critical targets.
