# Supplementary material for: Exploring the active ingredients and pharmacological mechanisms of the oral intake formula Huoxiang Suling Shuanghua Decoction on influenza virus type A based on network pharmacology and experimental exploration
Source: Front Microbiol. 2022 Nov 1;13:1040056. doi: 10.3389/fmicb.2022.1040056 (PMC9663660; doi:10.3389/fmicb.2022.1040056)

Supplementary Data Sheet 10: Original images of lung HE of three repeats.

Normal group repeat 1

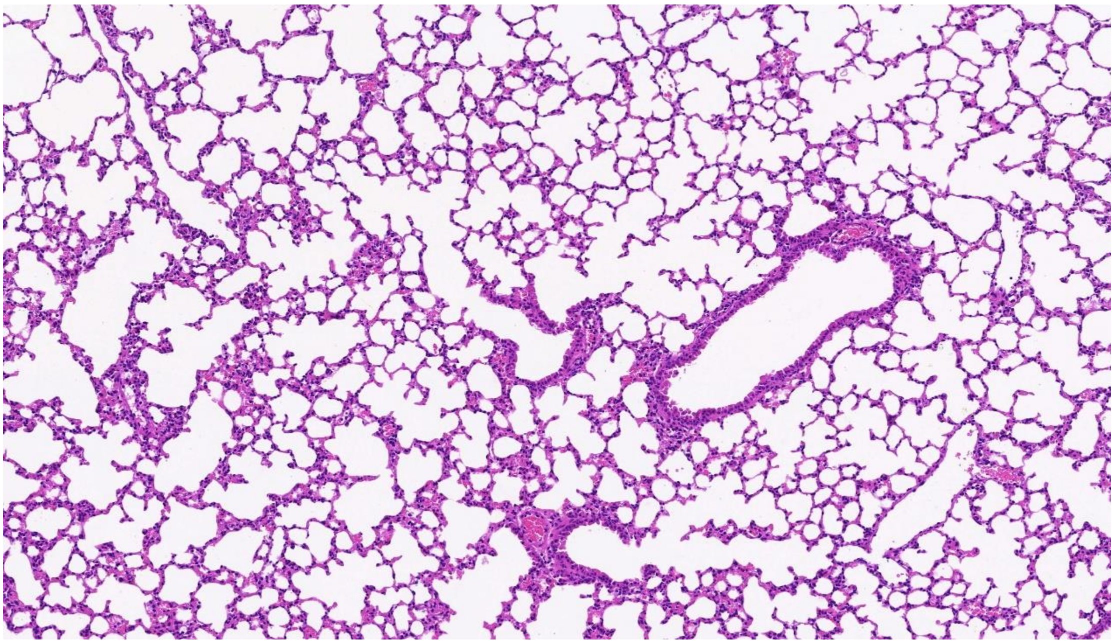

Normal group repeat 2

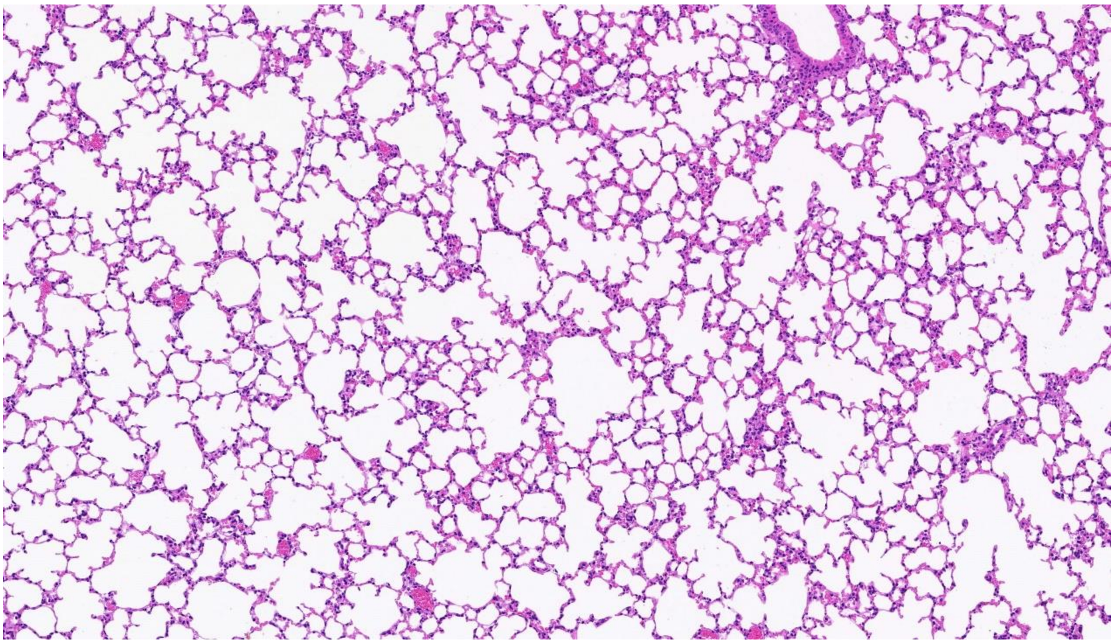

Normal group repeat 3

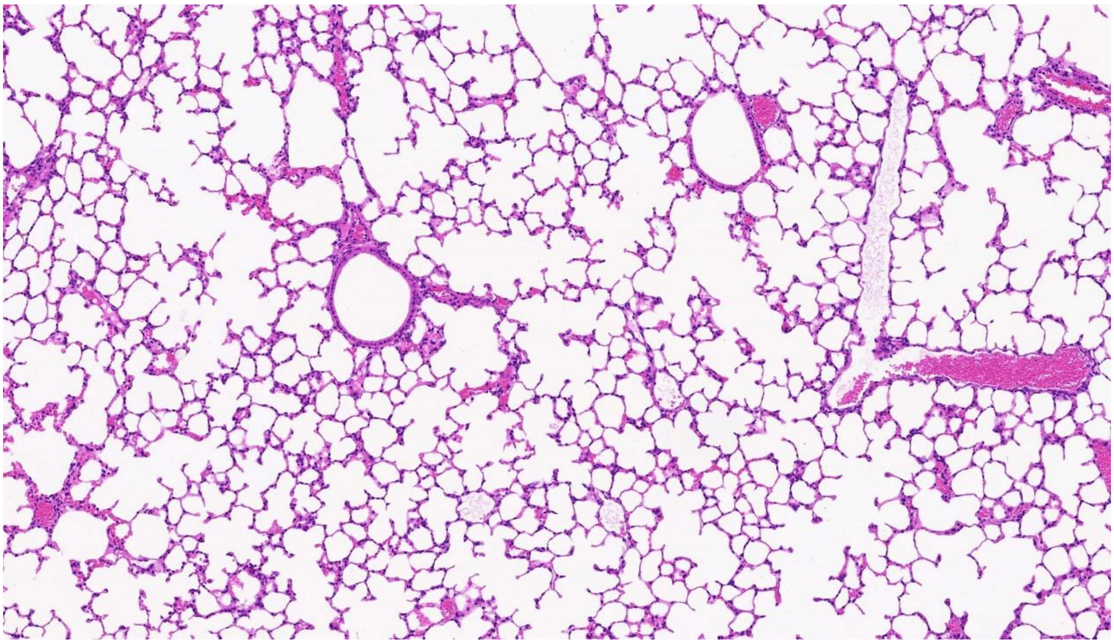

Infected group repeat 1

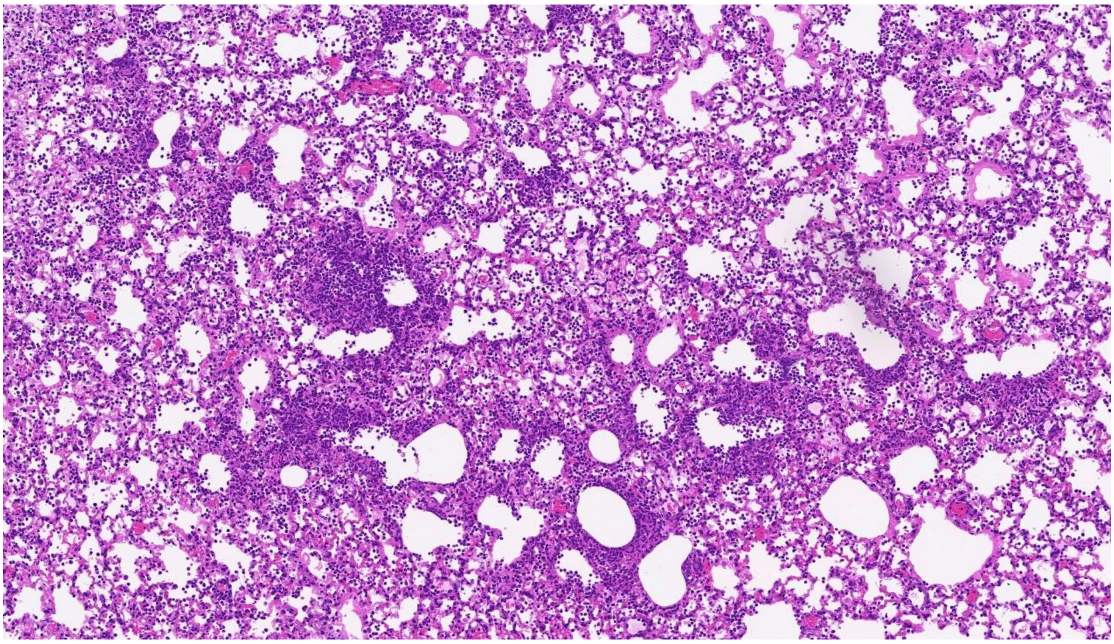

Infected group repeat 2

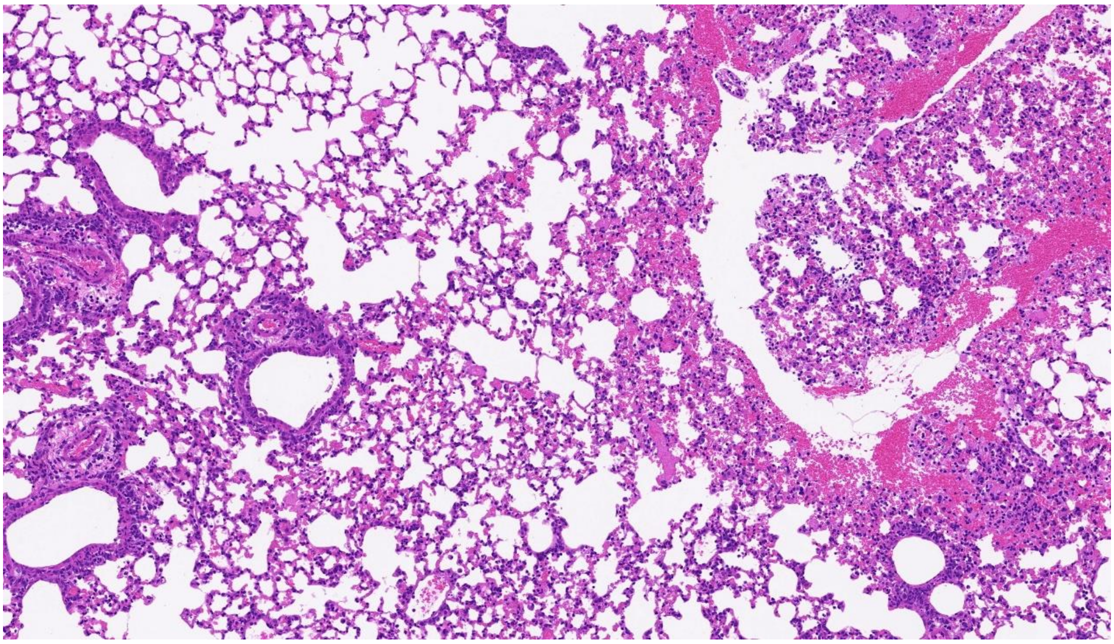

Infected group repeat 3

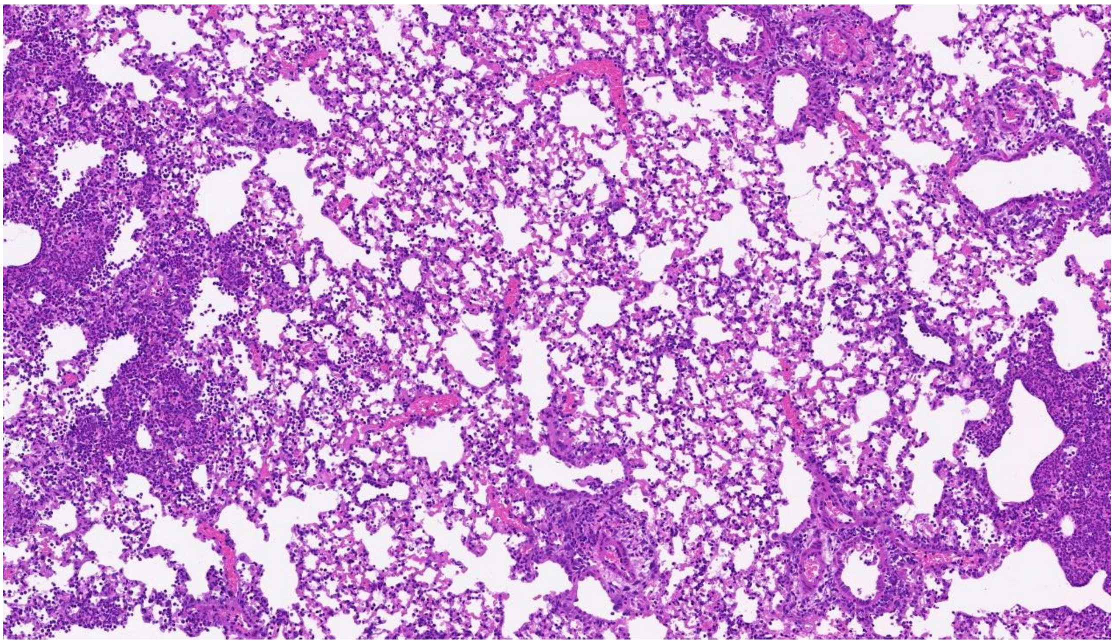

Oseltamivir group repeat 1

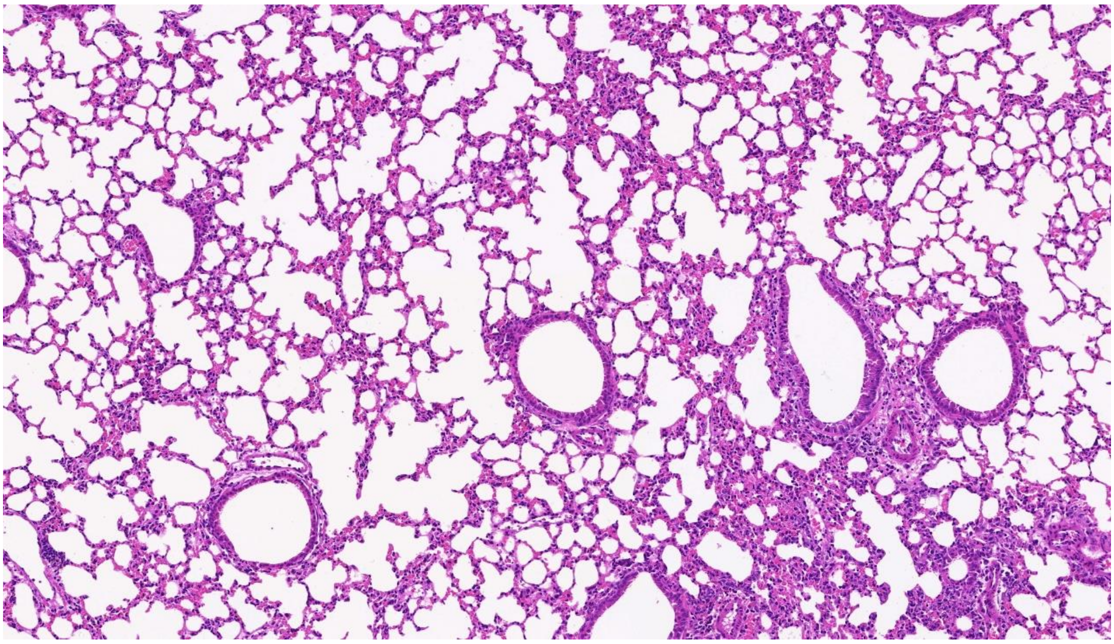

Oseltamivir group repeat 2

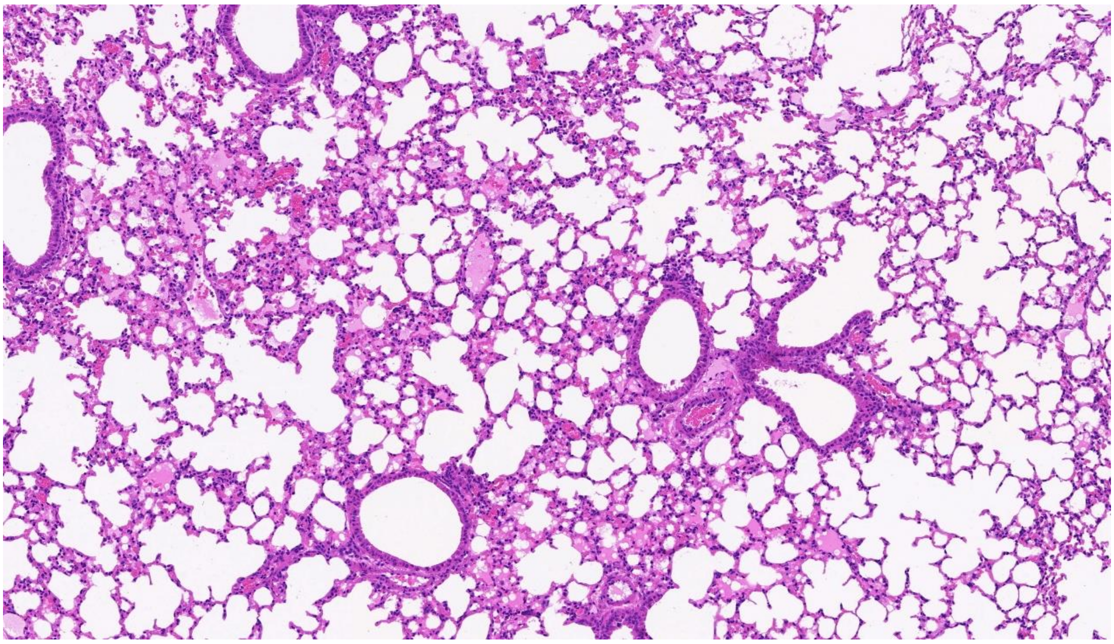

Oseltamivir group repeat 3

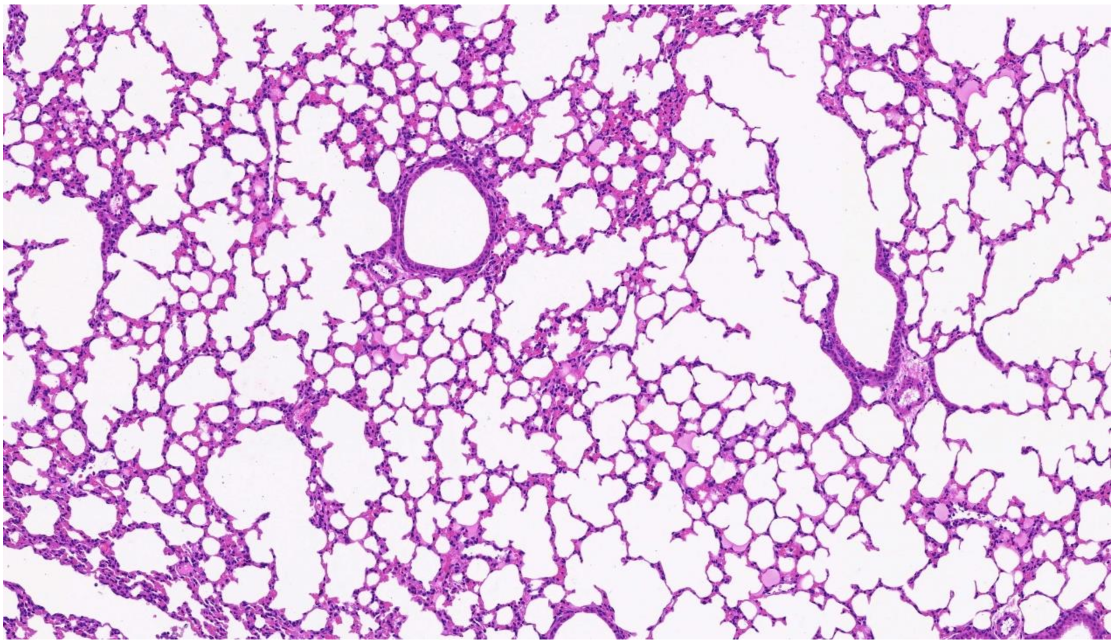

HSSD-L group repeat 1

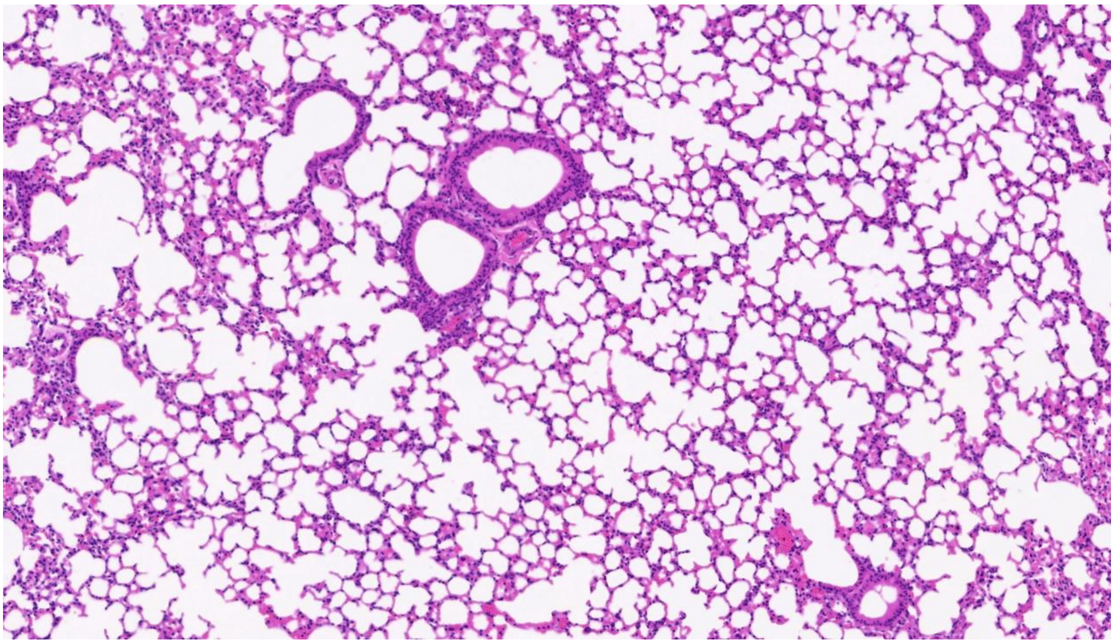

HSSD-L group repeat 2

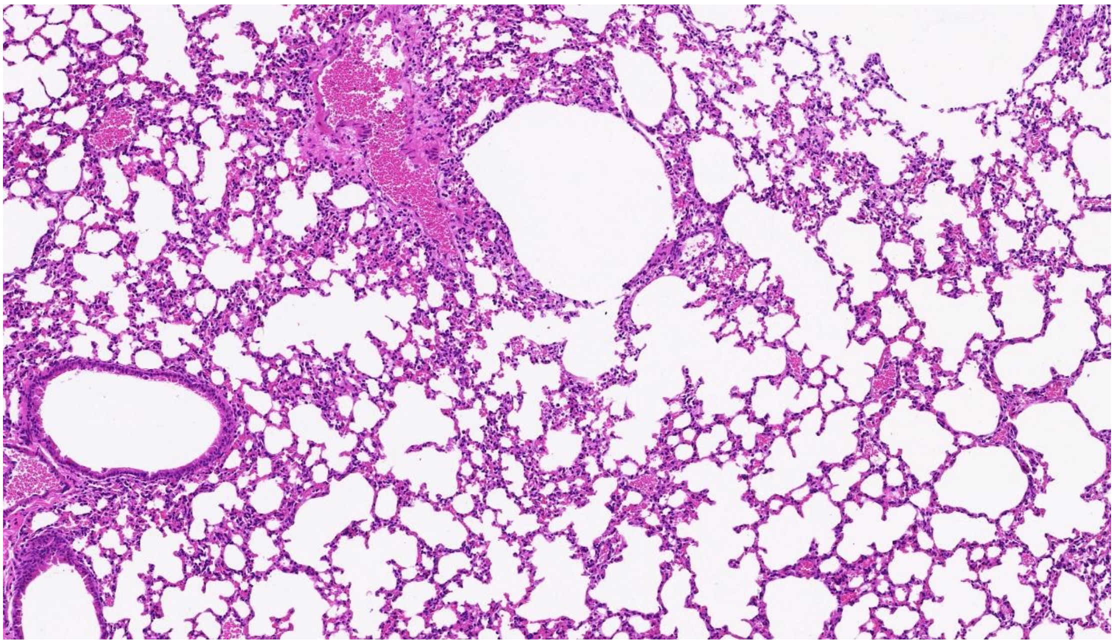

HSSD-L group repeat 3

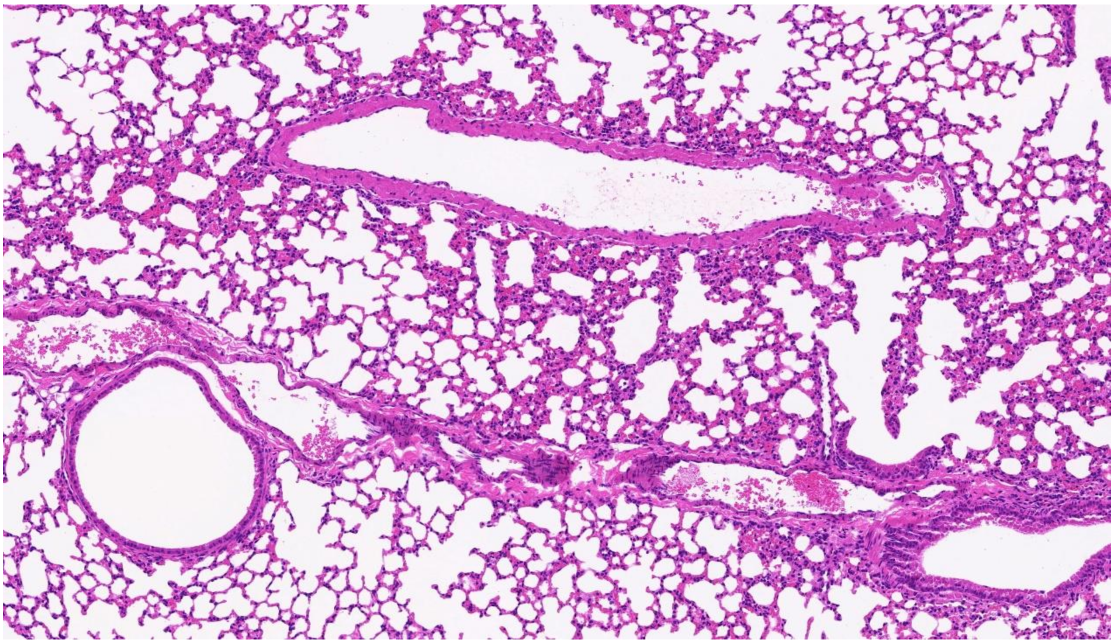

HSSD-M group repeat 1

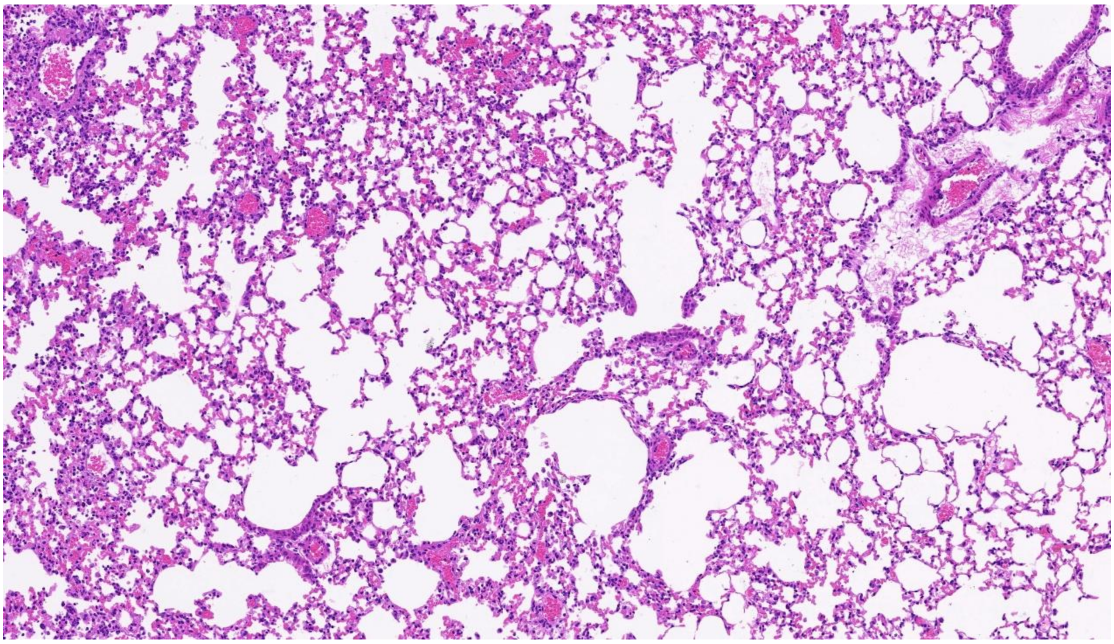

HSSD-M group repeat 2

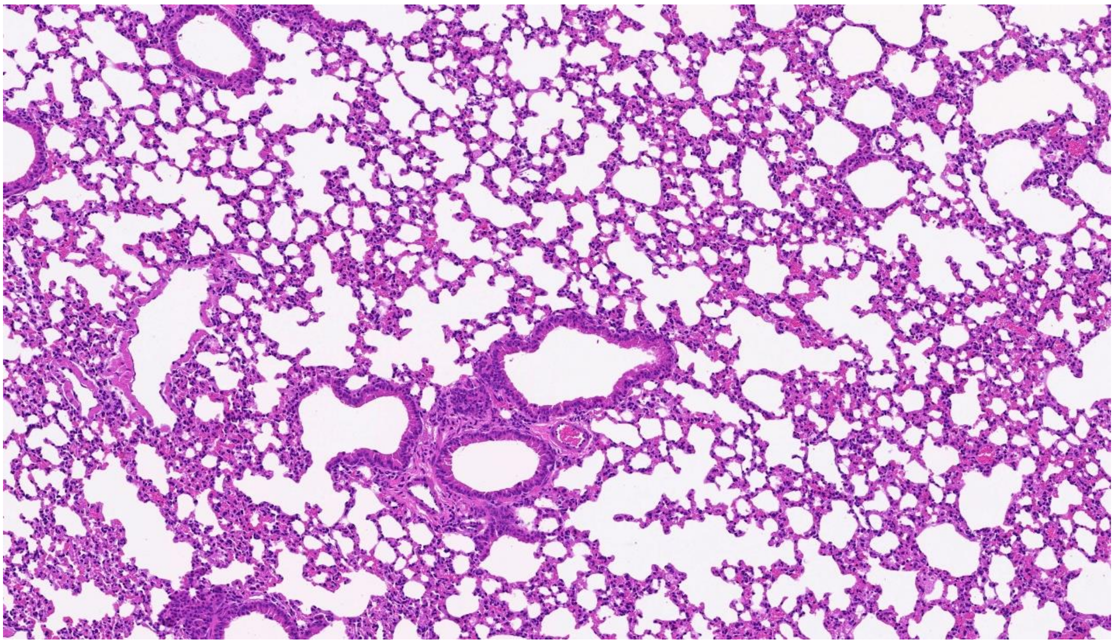

HSSD-M group repeat 3

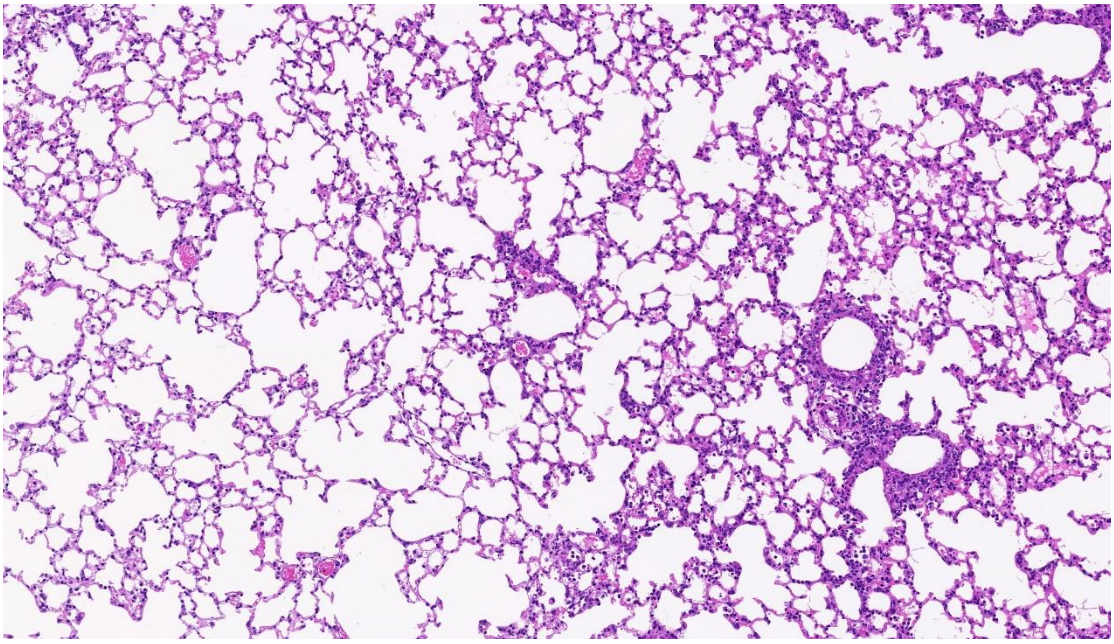

HSSD-H group repeat 1

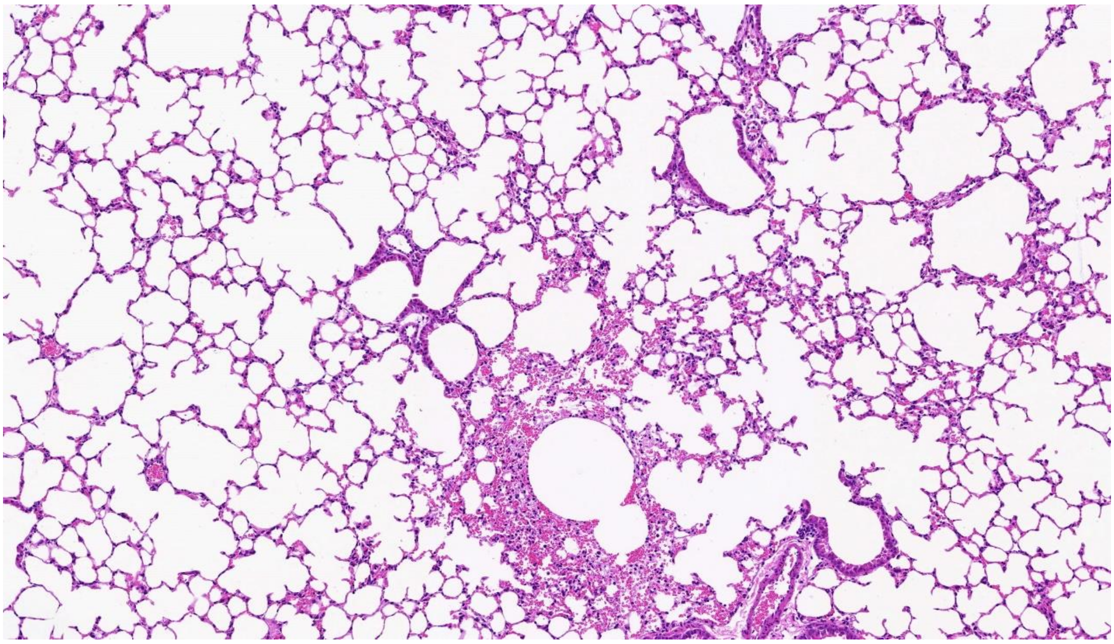

HSSD-H group repeat 2

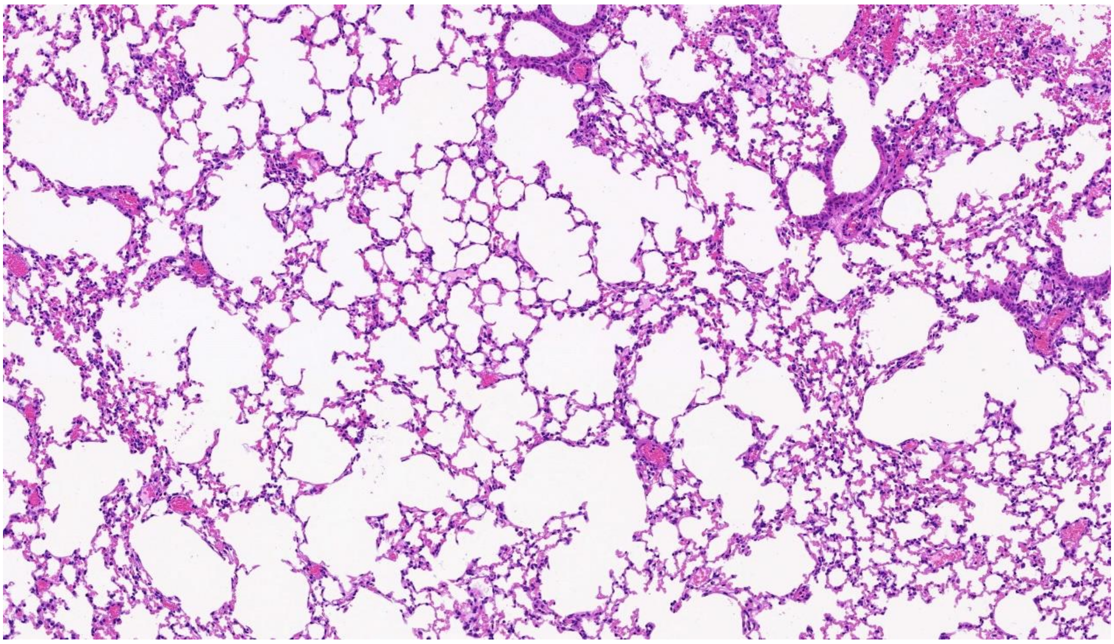

HSSD-H group repeat 3

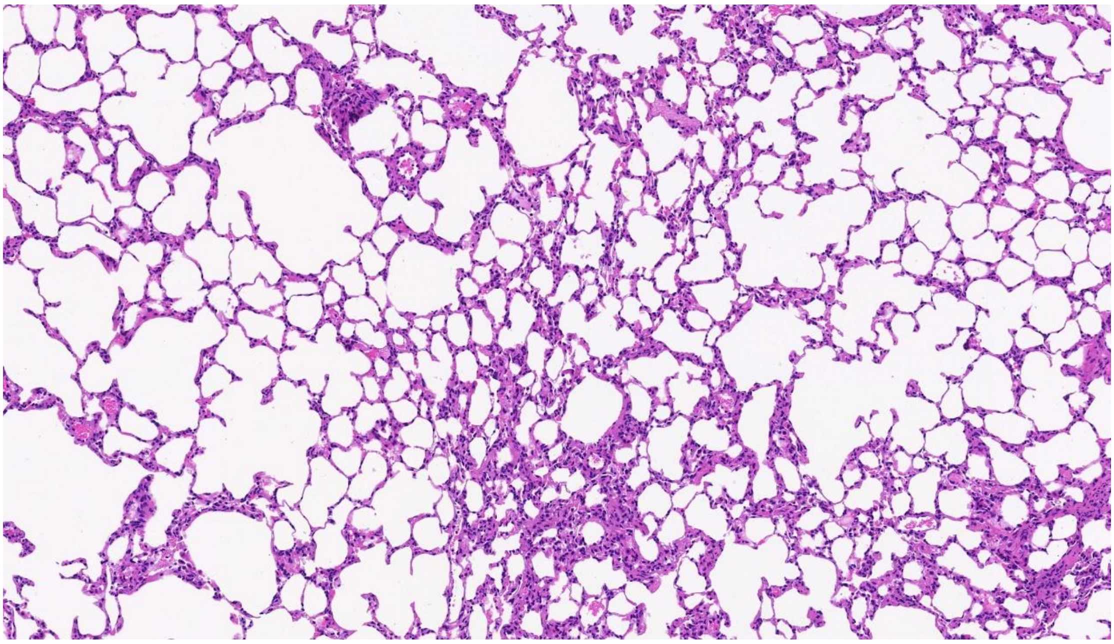

Supplement: Supplementary file 9 [file Data_Sheet_10.PDF]
