# Supplementary material for: Exploring the active ingredients and pharmacological mechanisms of the oral intake formula Huoxiang Suling Shuanghua Decoction on influenza virus type A based on network pharmacology and experimental exploration
Source: Front Microbiol. 2022 Nov 1;13:1040056. doi: 10.3389/fmicb.2022.1040056 (PMC9663660; doi:10.3389/fmicb.2022.1040056)

Supplementary Data Sheet 11: Original images of lung TUNEL of three repeats.

Normal group DAPI repeat 1

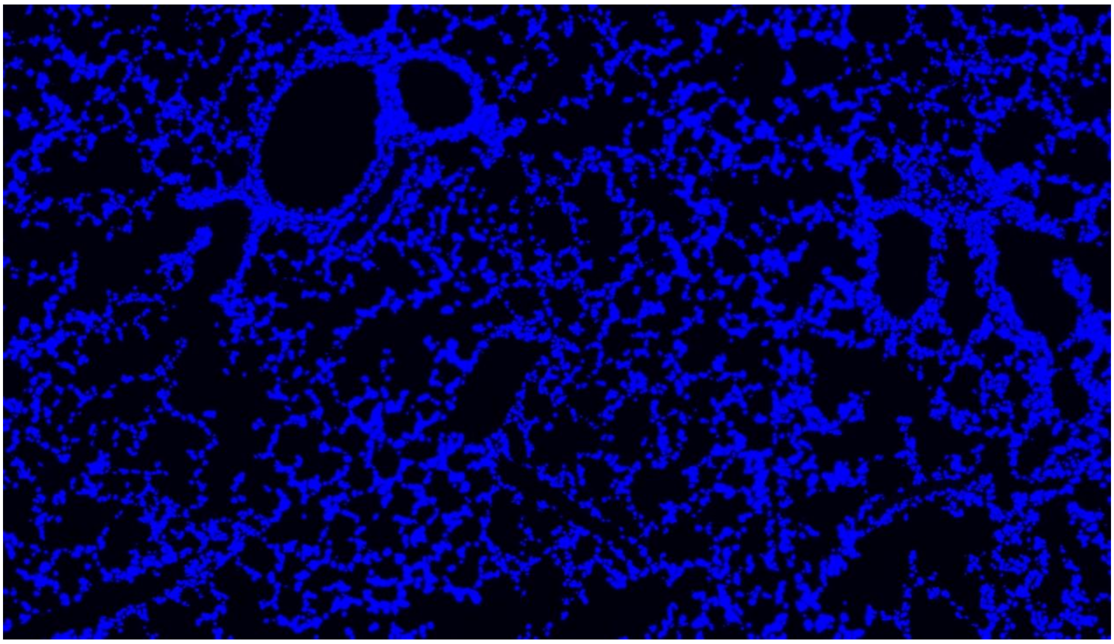

Normal group TUNEL repeat 1

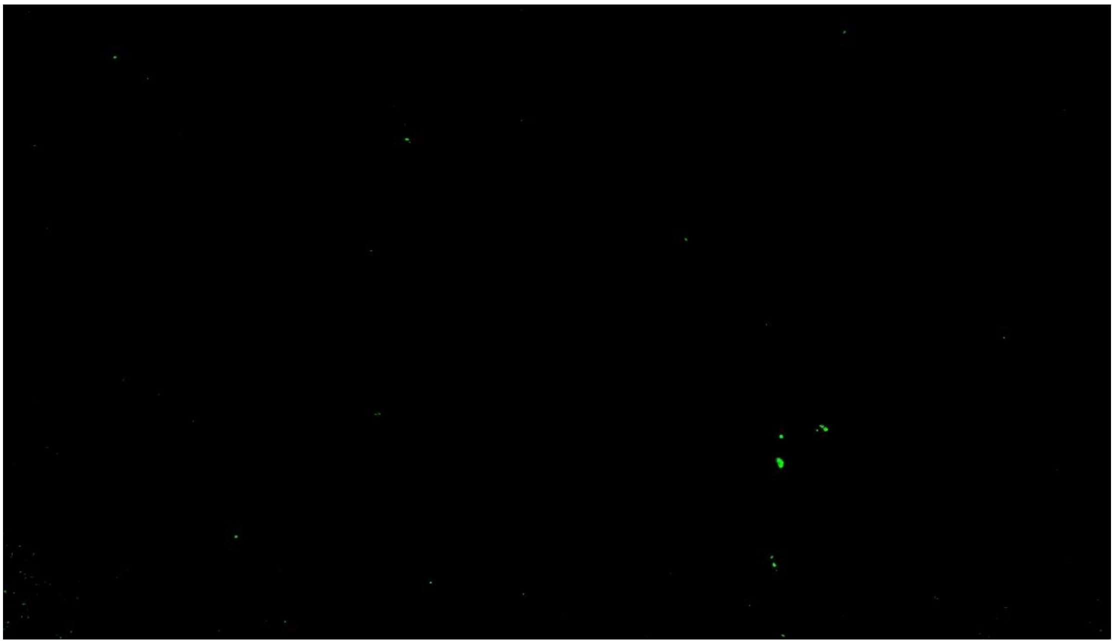

Normal group Merge repeat 1

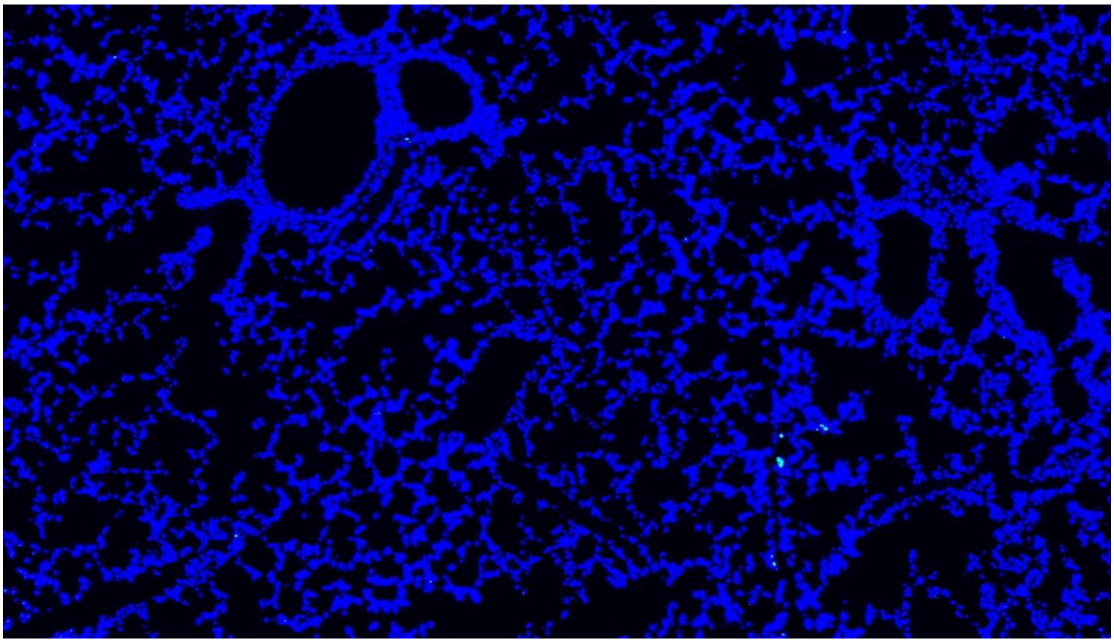

Normal group DAPI repeat 2

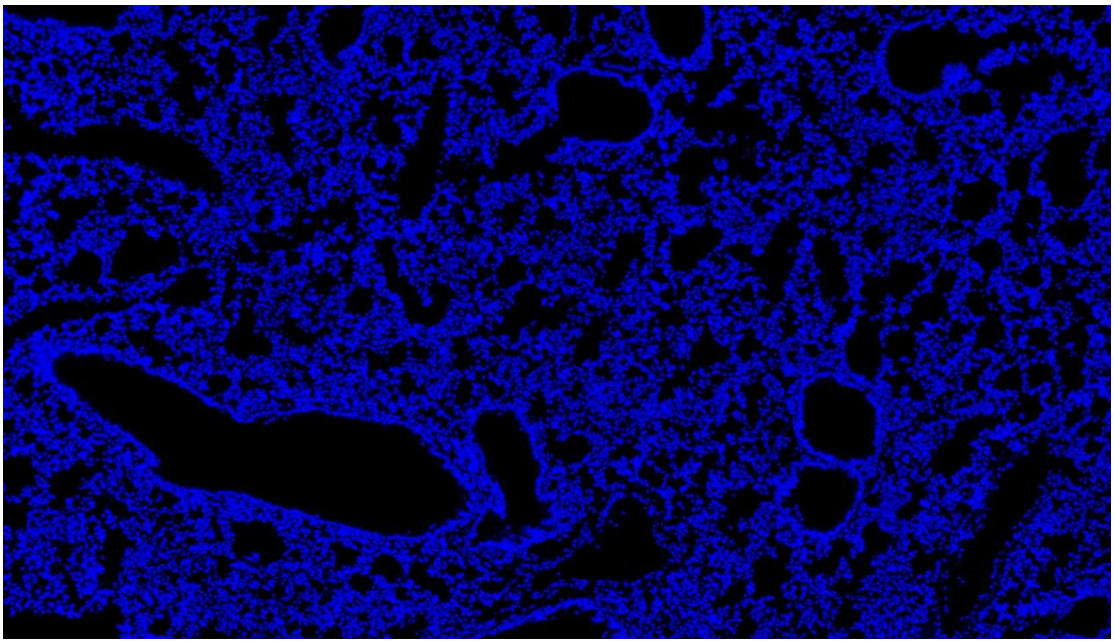

Normal group TUNEL repeat 2

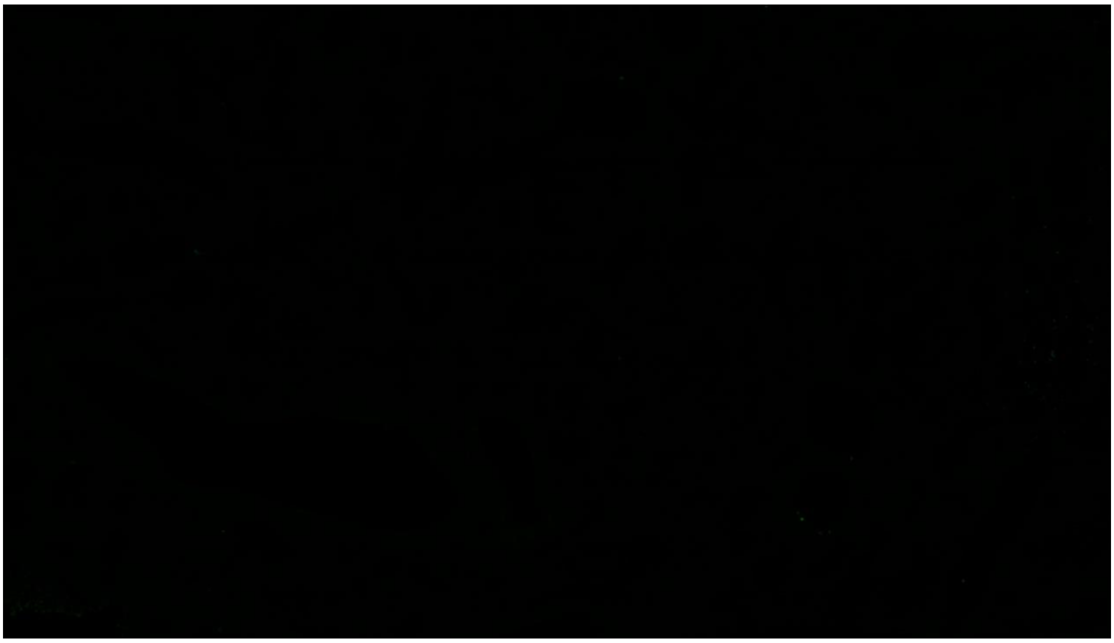

Normal group Merge repeat 2

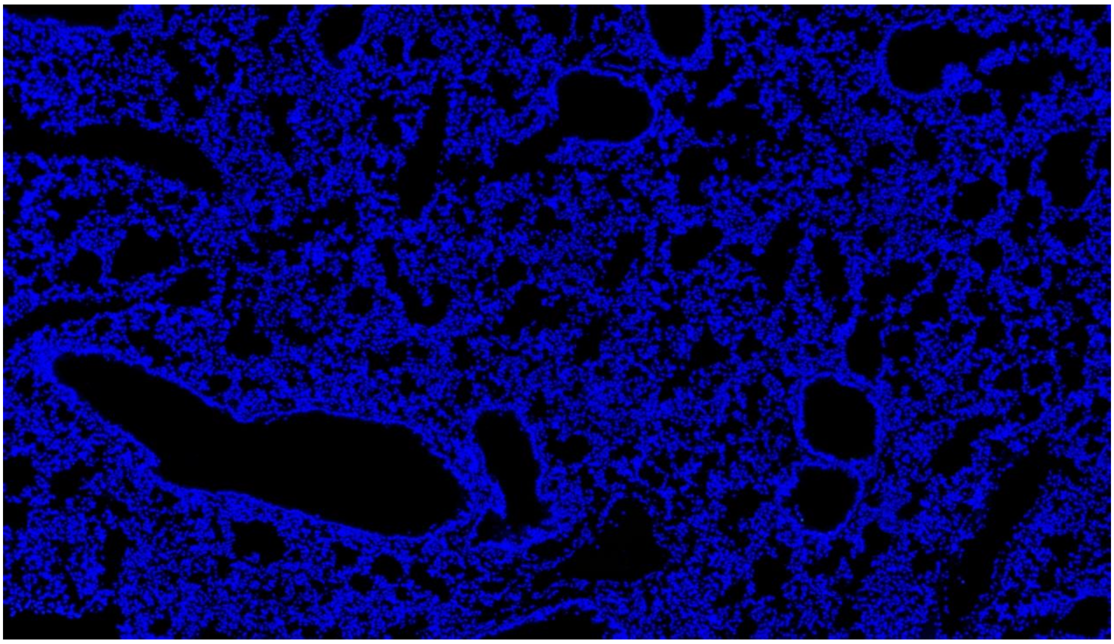

Normal group DAPI repeat 3

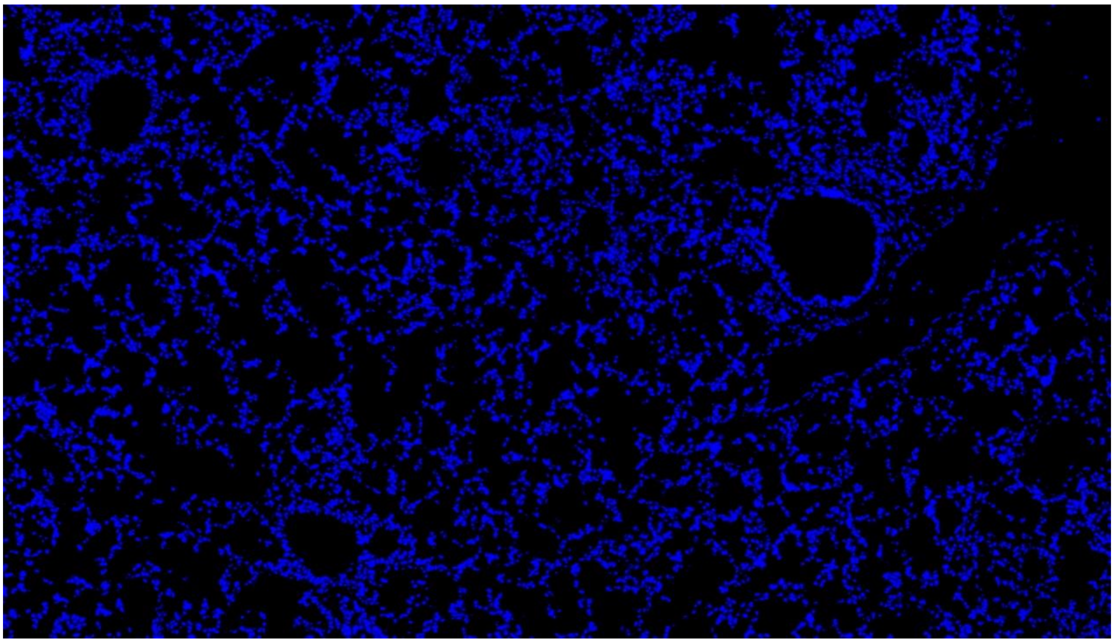

Normal group TUNEL repeat 3

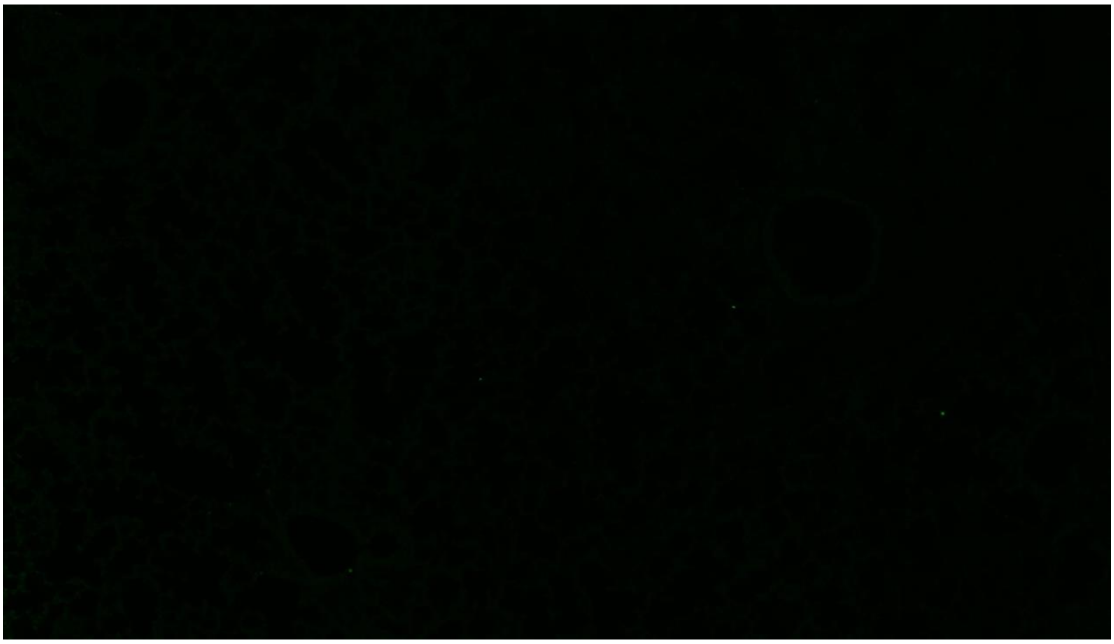

Normal group Merge repeat 3

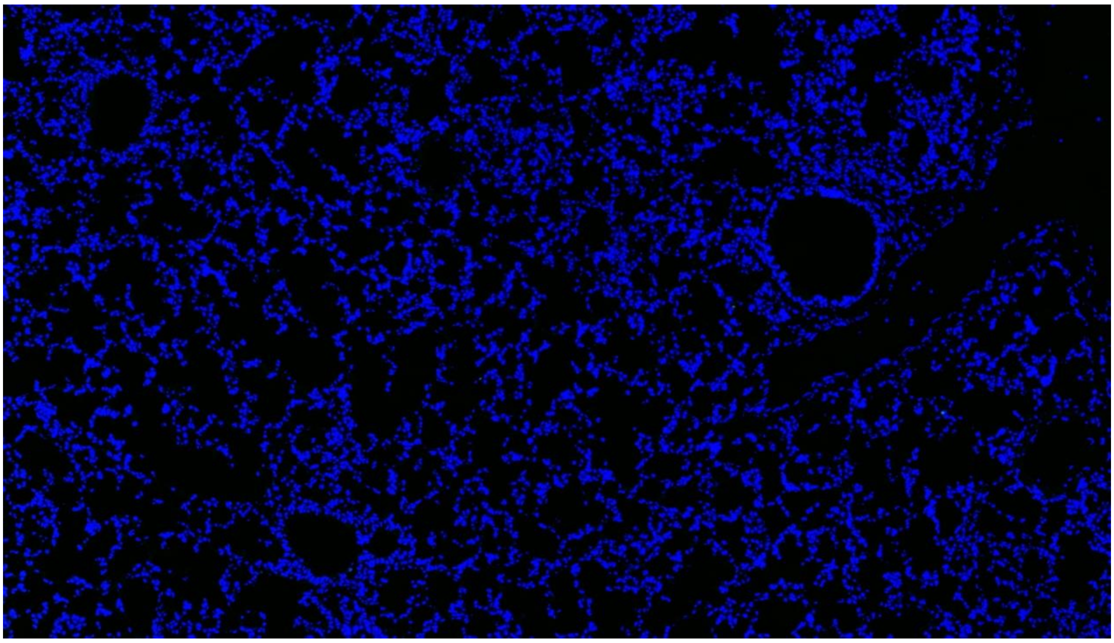

Infected group DAPI repeat 1

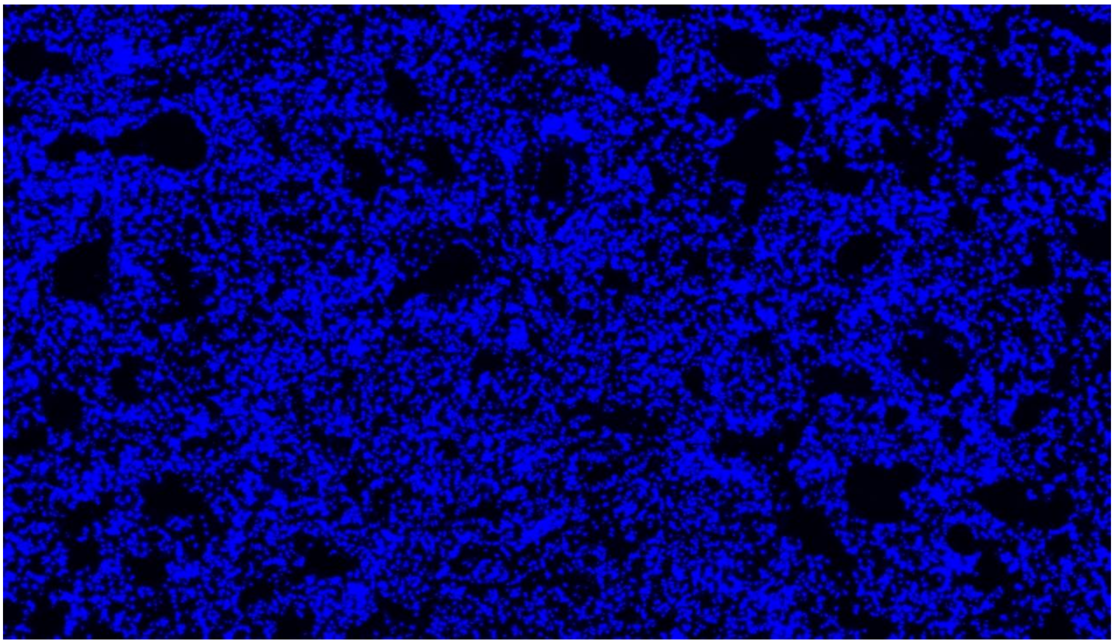

Infected group TUNEL repeat 1

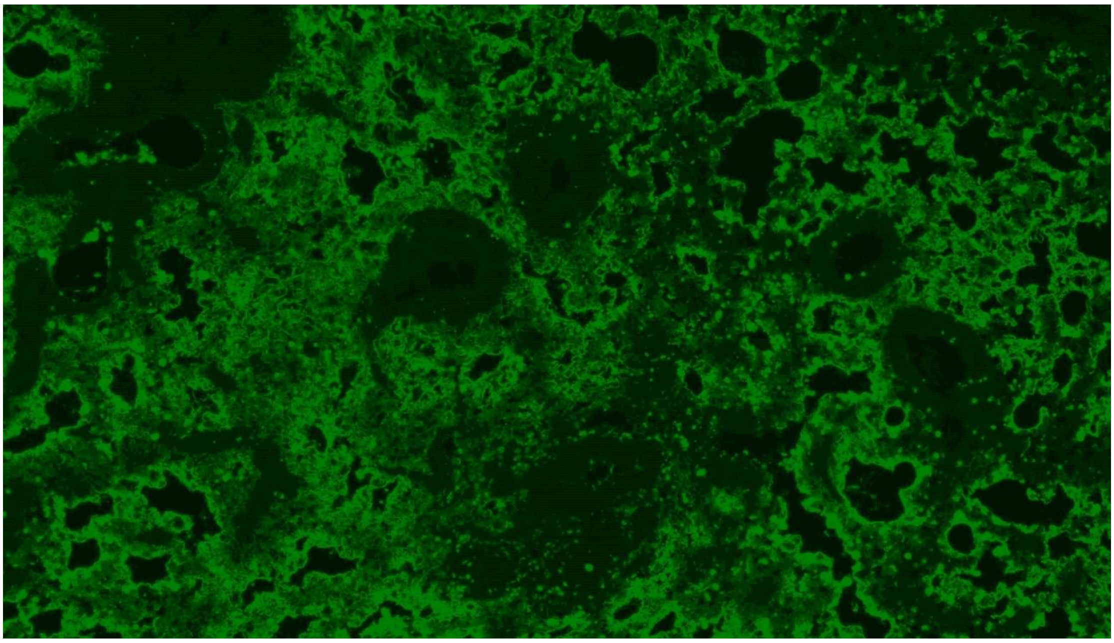

Infected group Merge repeat 1

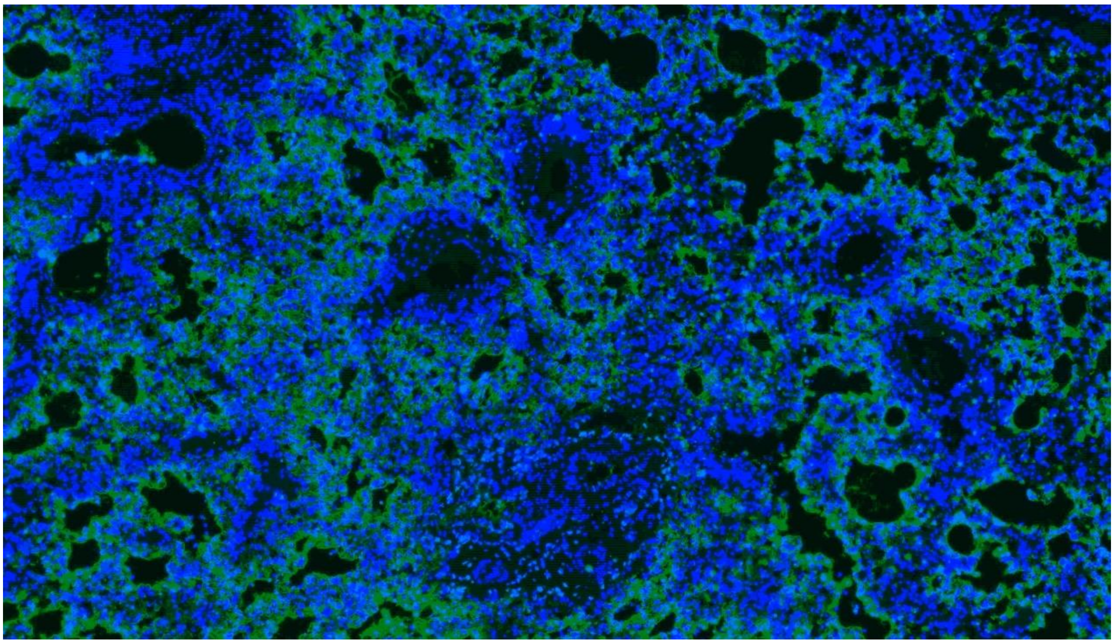

Infected group DAPI repeat 2

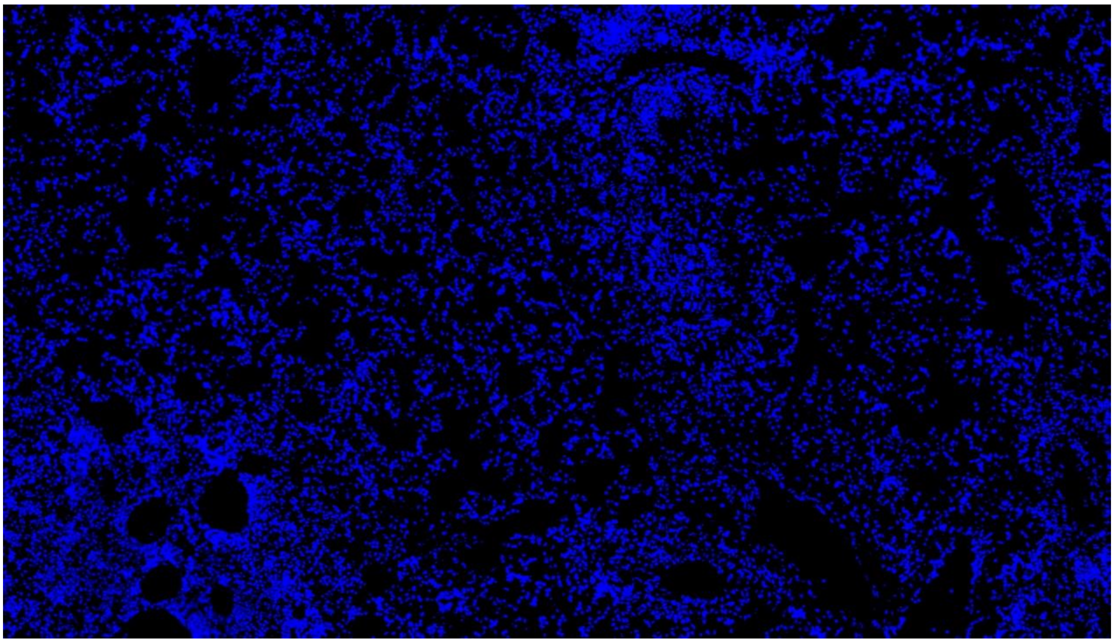

Infected group TUNEL repeat 2

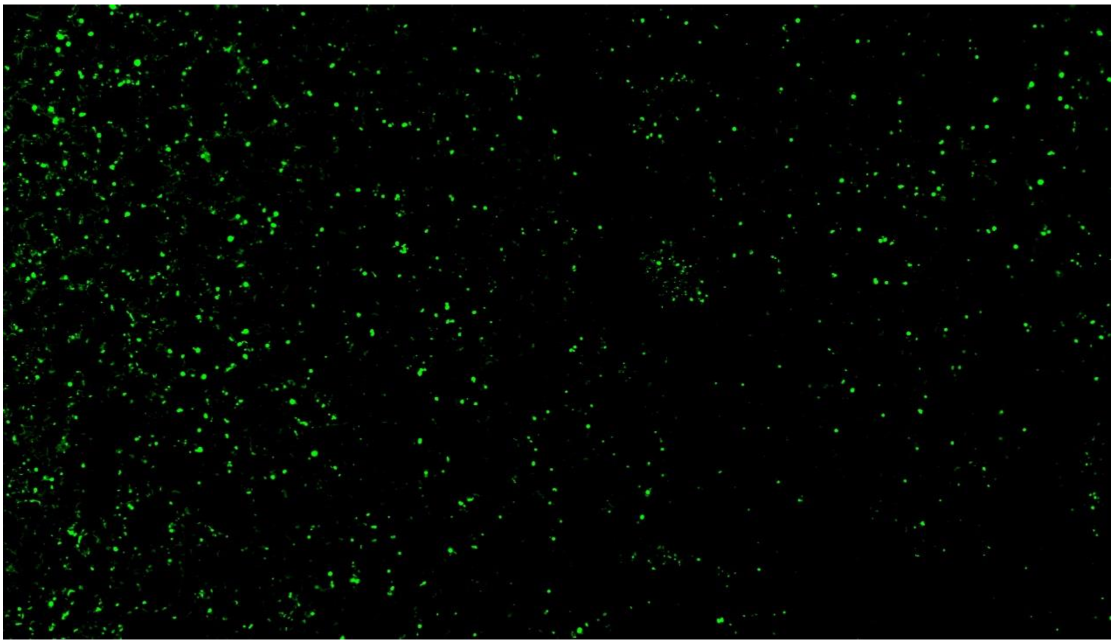

Infected group Merge repeat 2

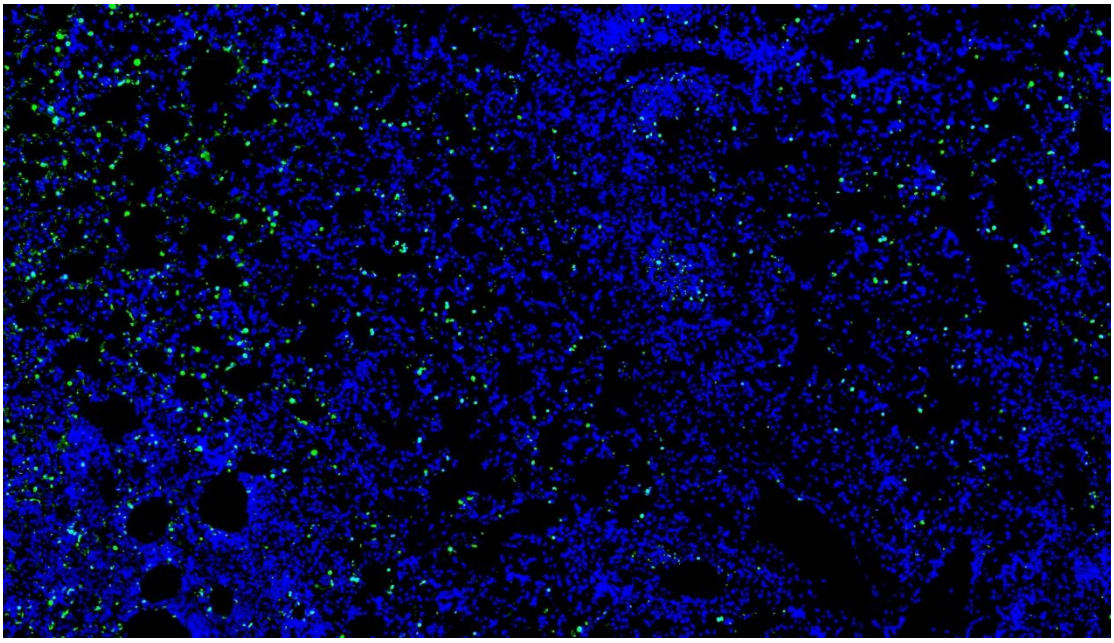

Infected group DAPI repeat 3

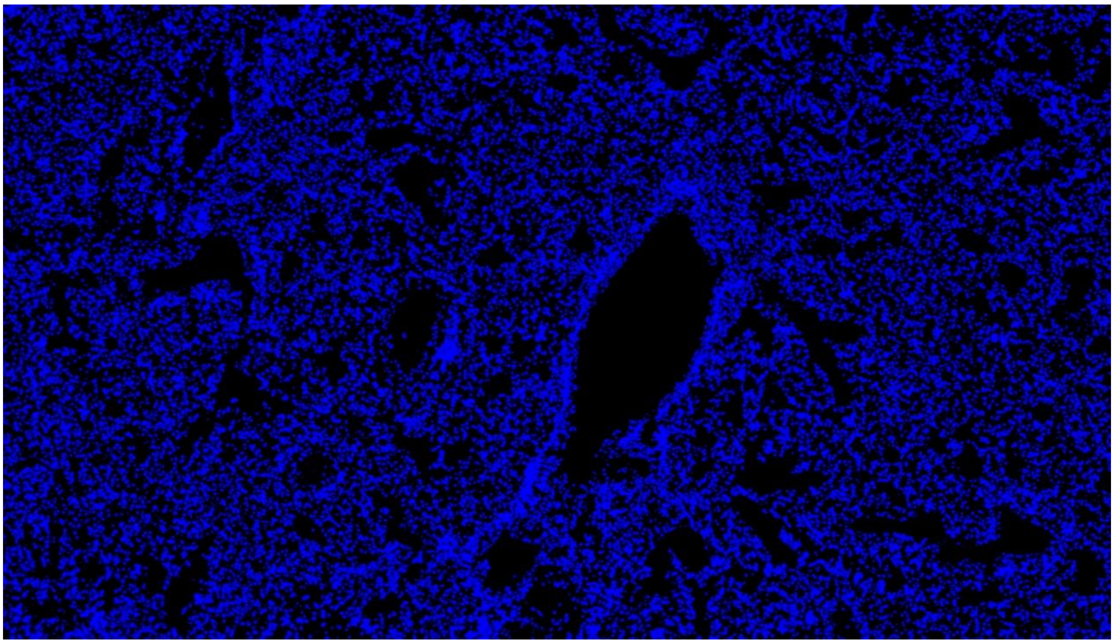

Infected group TUNEL repeat 3

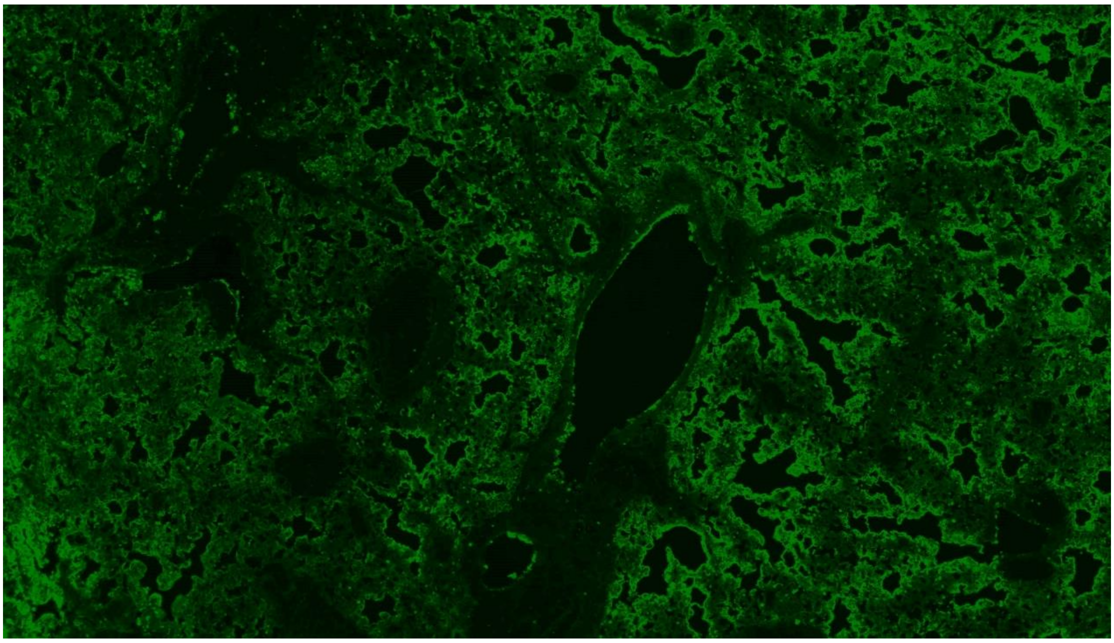

Infected group Merge repeat 3

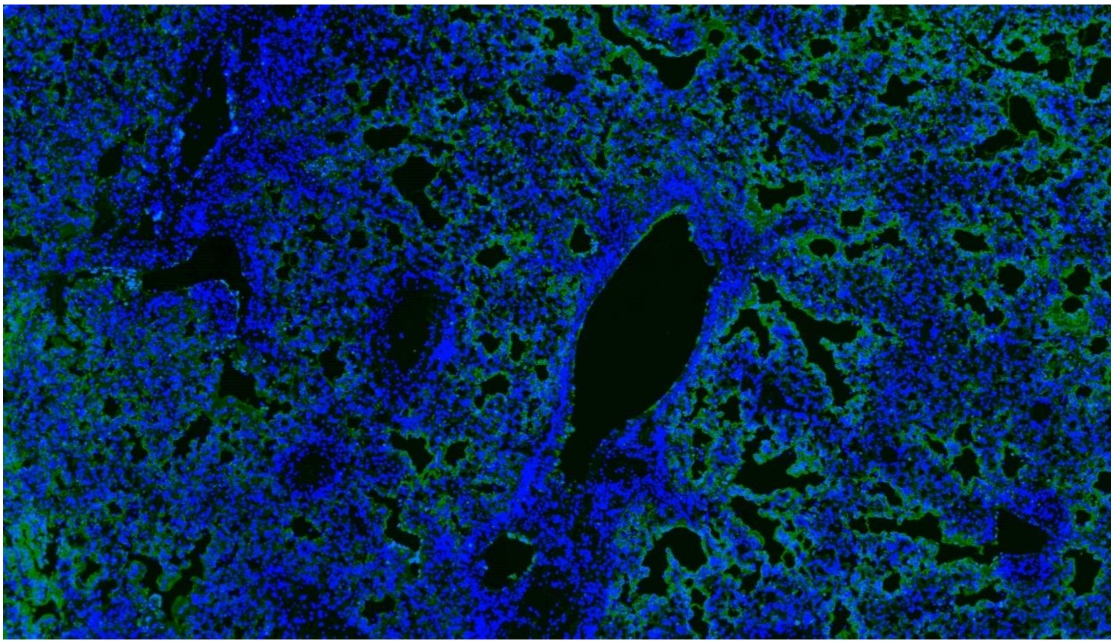

Oseltamivir group DAPI repeat 1

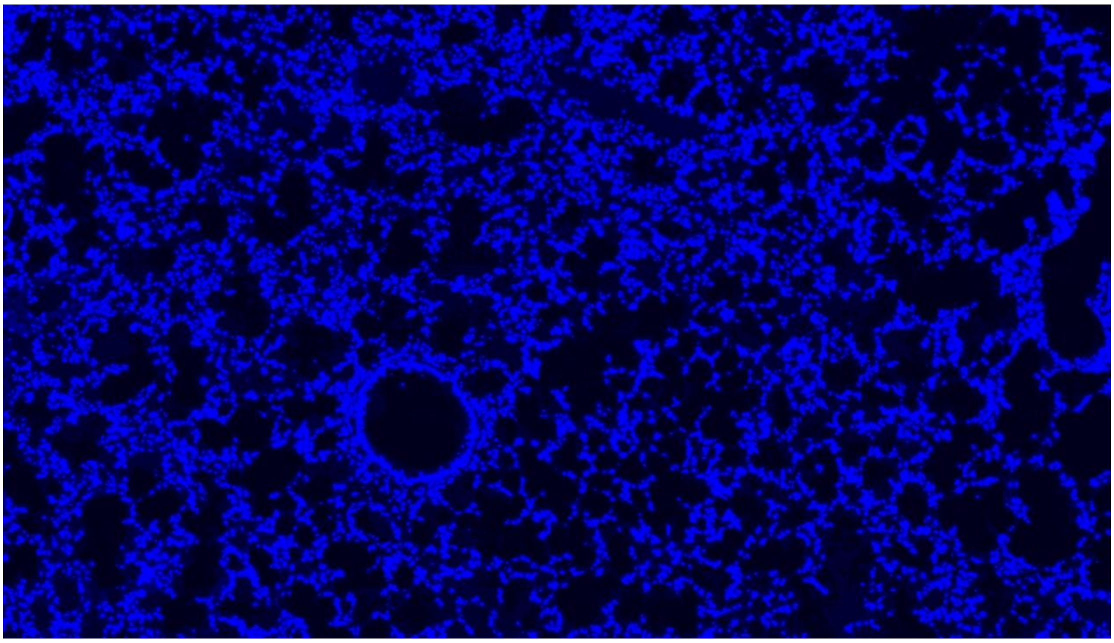

Oseltamivir group TUNEL repeat 1

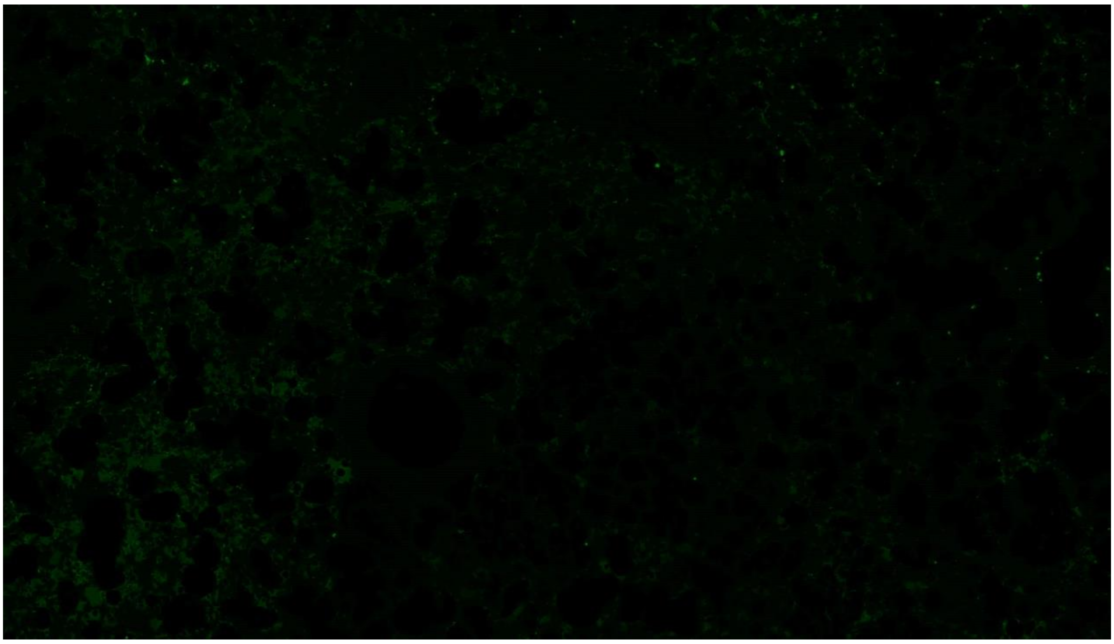

Oseltamivir group Merge repeat 1

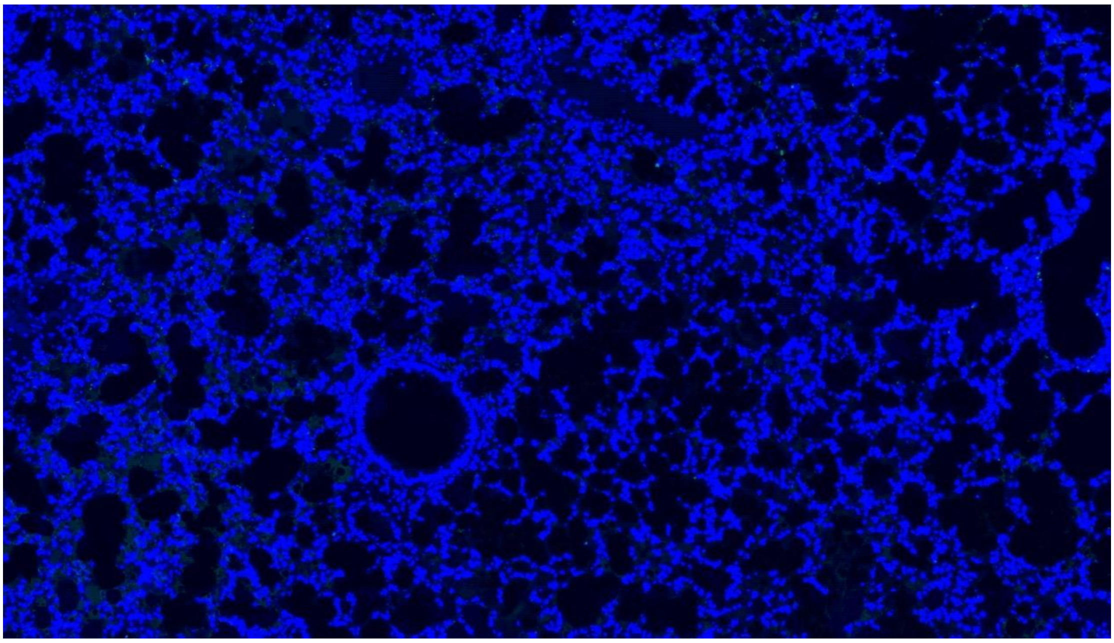

Oseltamivir group DAPI repeat 2

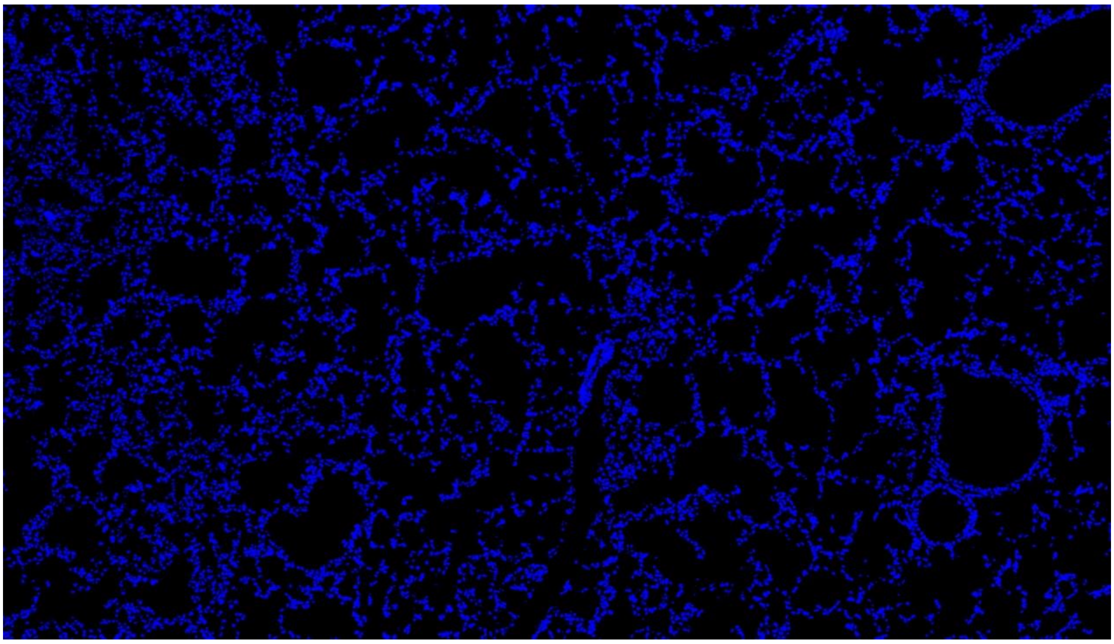

Oseltamivir group TUNEL repeat 2

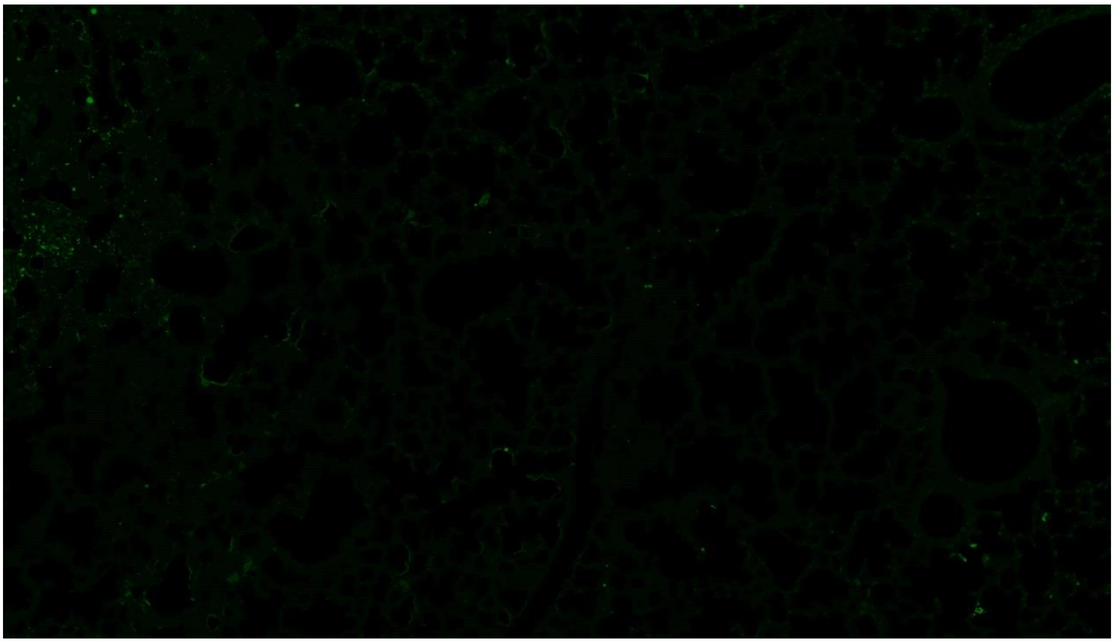

Oseltamivir group Merge repeat 2

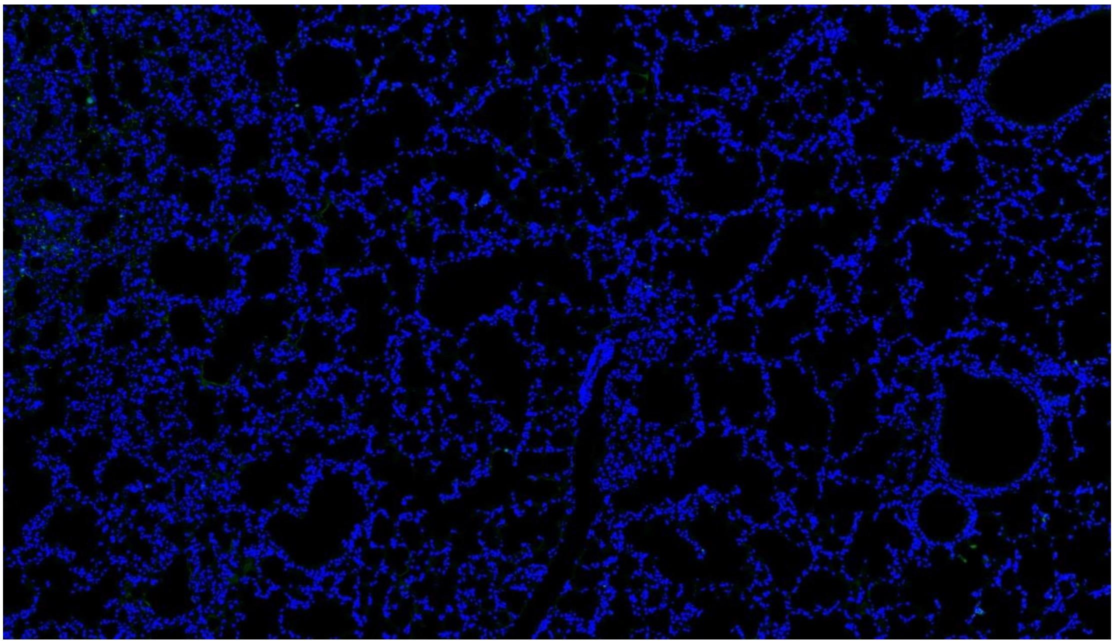

Oseltamivir group DAPI repeat 3

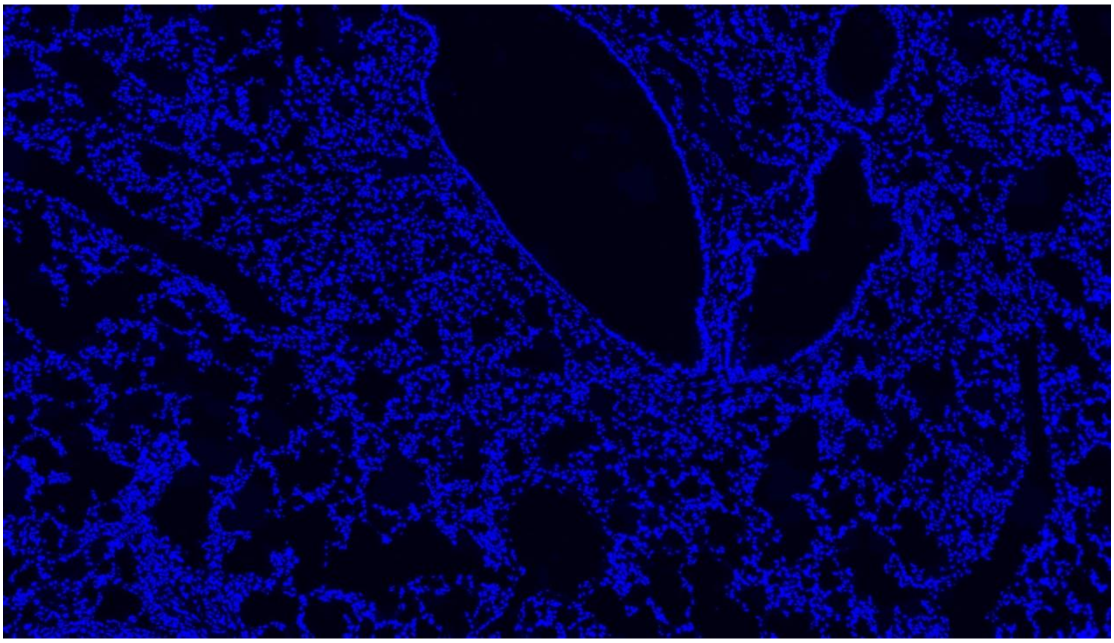

Oseltamivir group TUNEL repeat 3

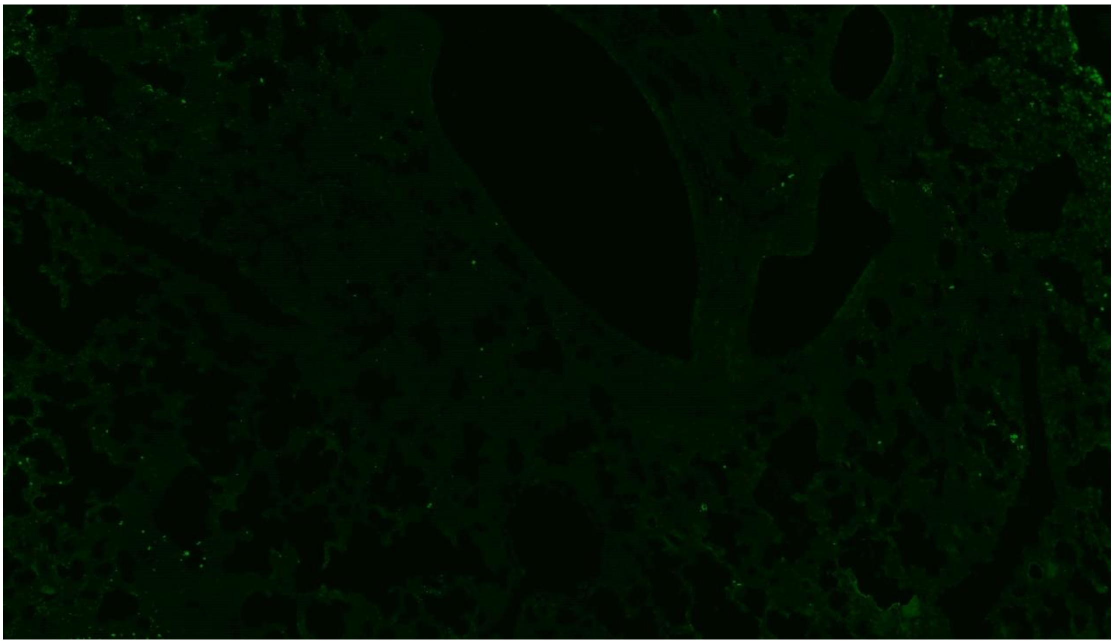

Oseltamivir group Merge repeat 3

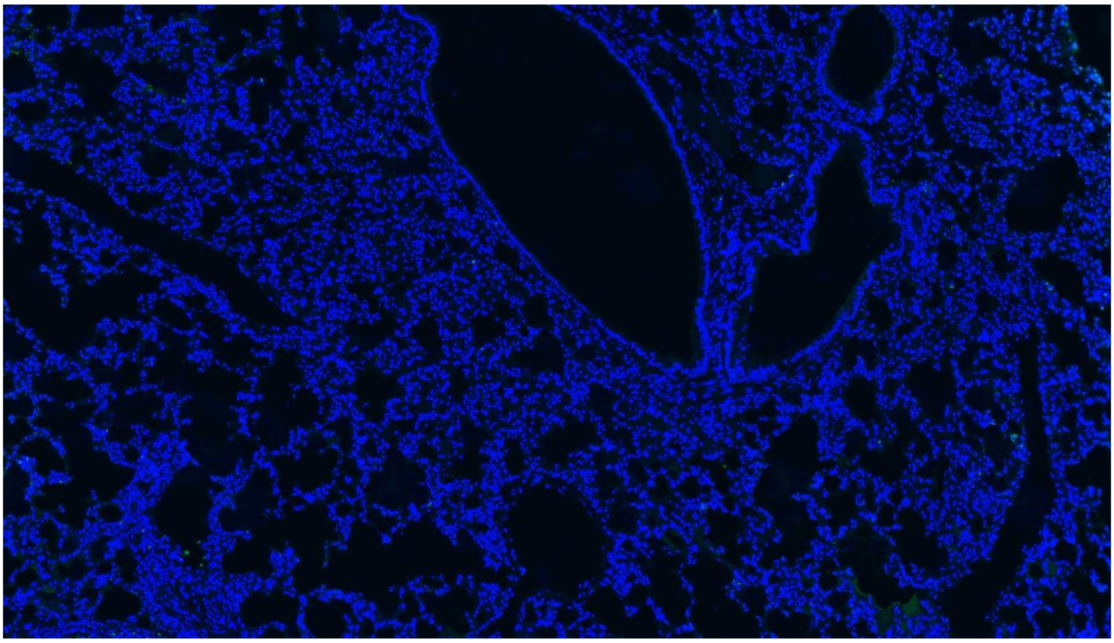

HSSD-L group DAPI repeat 1

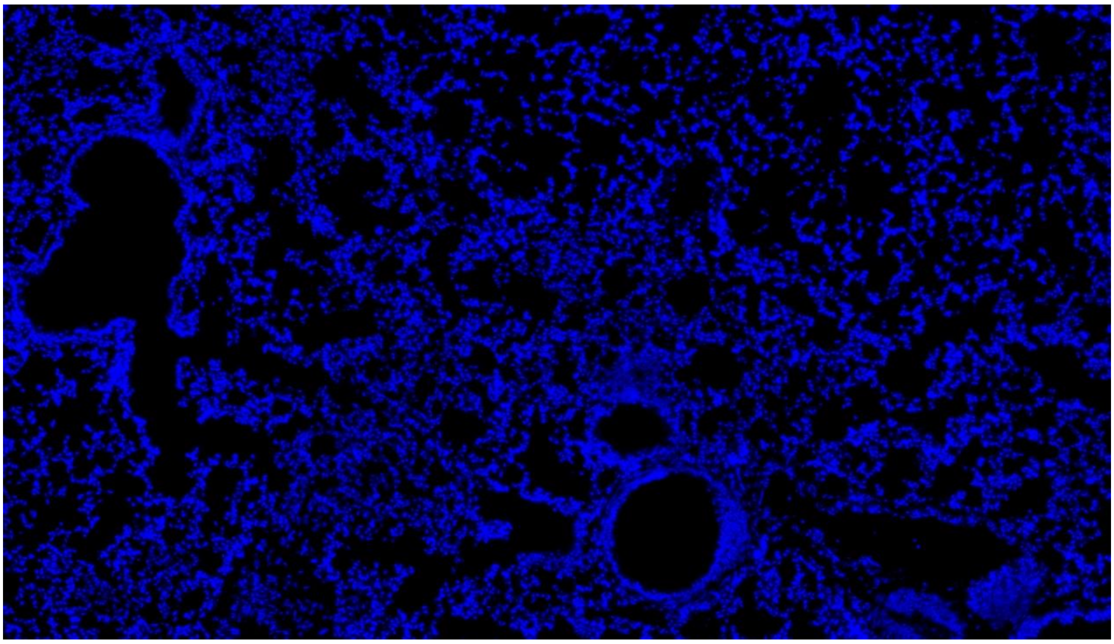

HSSD-L group TUNEL repeat 1

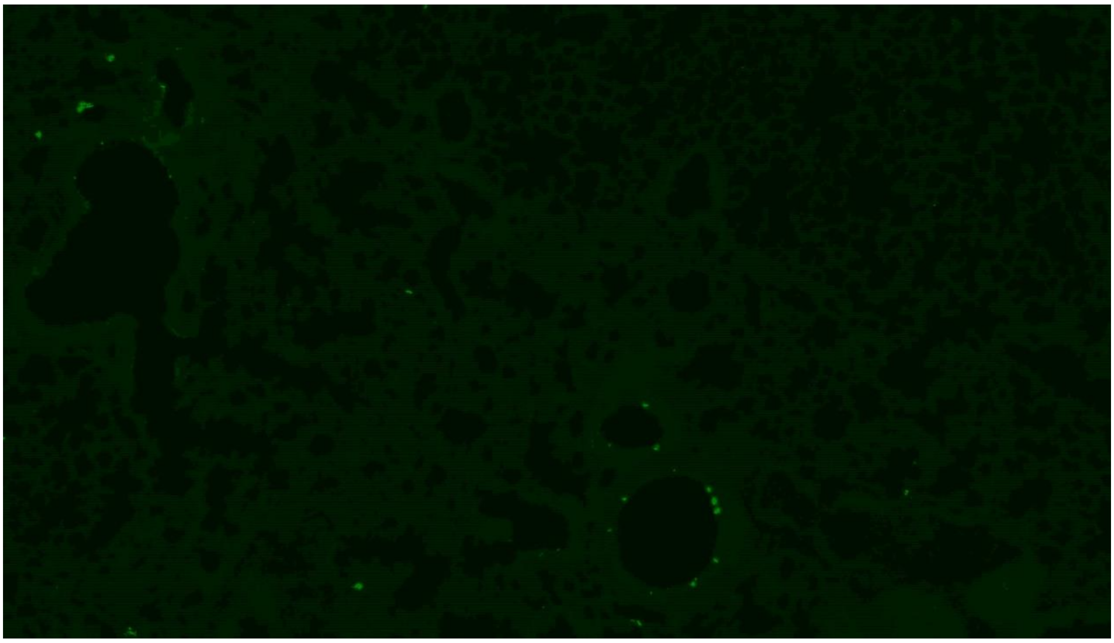

HSSD-L group Merge repeat 1

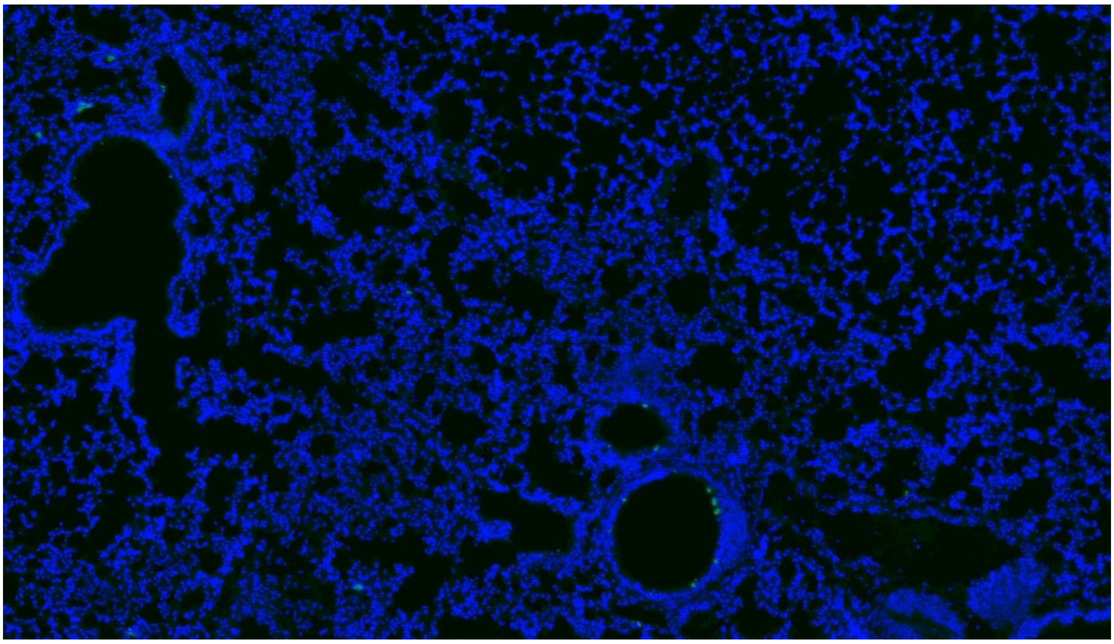

HSSD-L group DAPI repeat 2

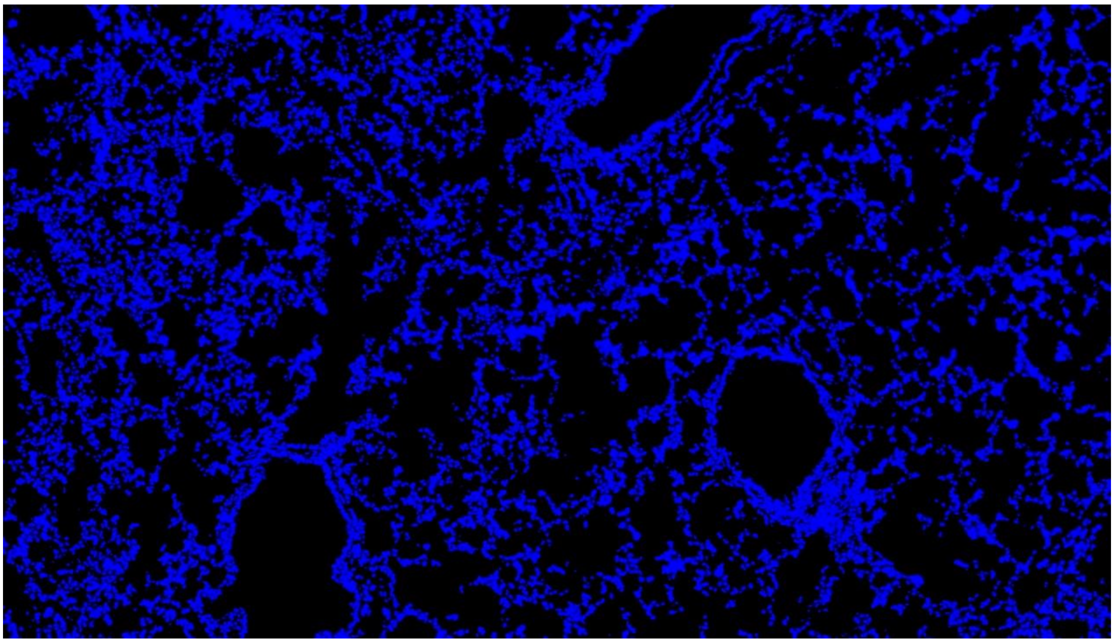

HSSD-L group TUNEL repeat 2

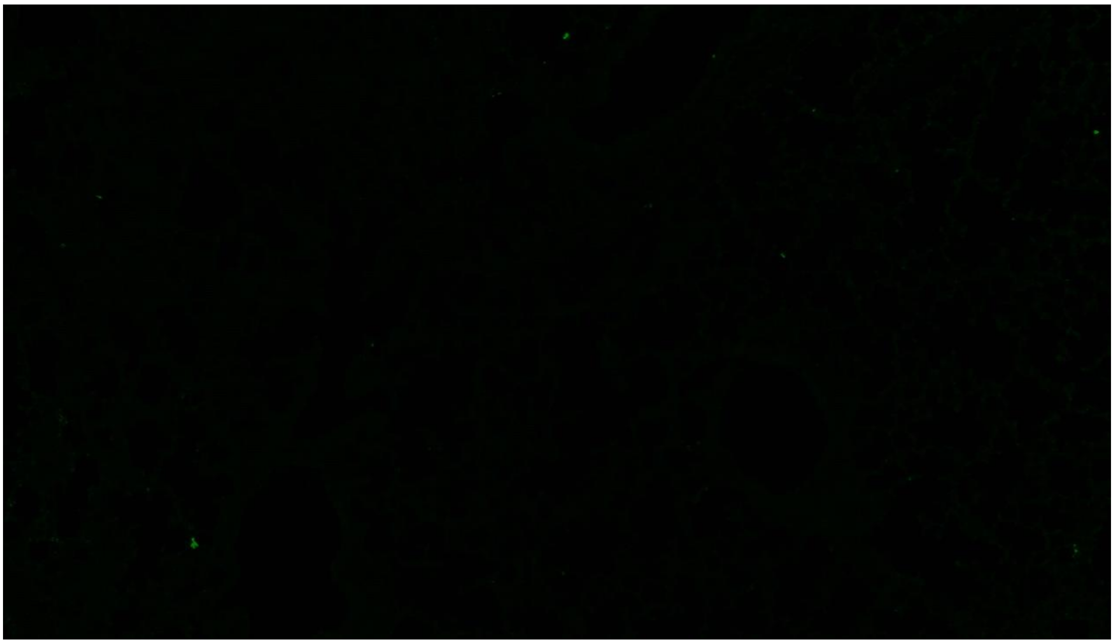

HSSD-L group Merge repeat 2

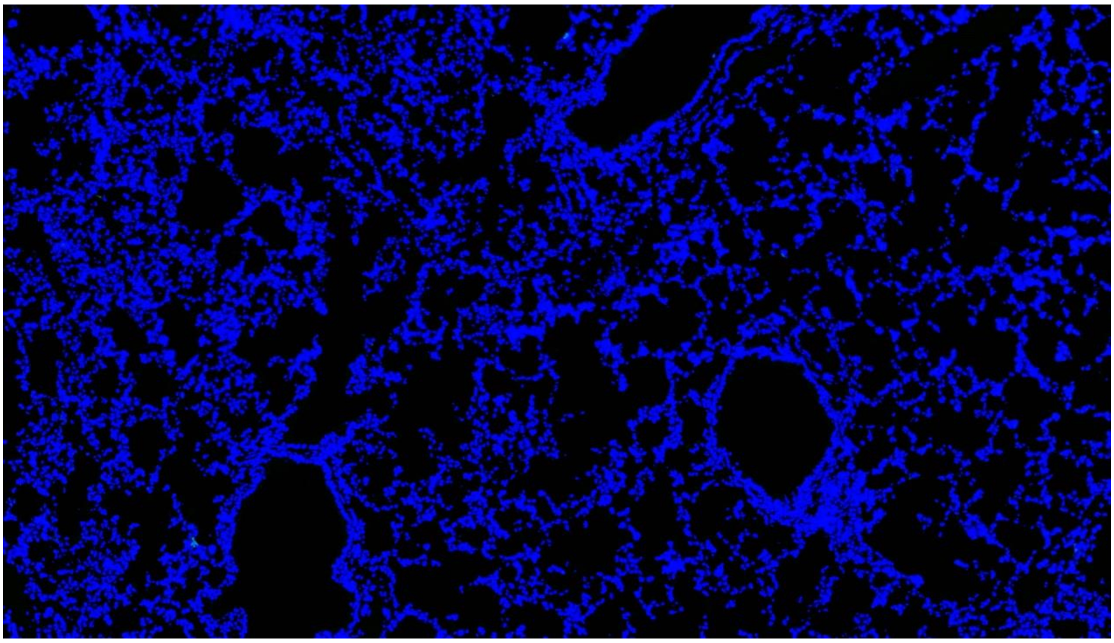

HSSD-L group DAPI repeat 3

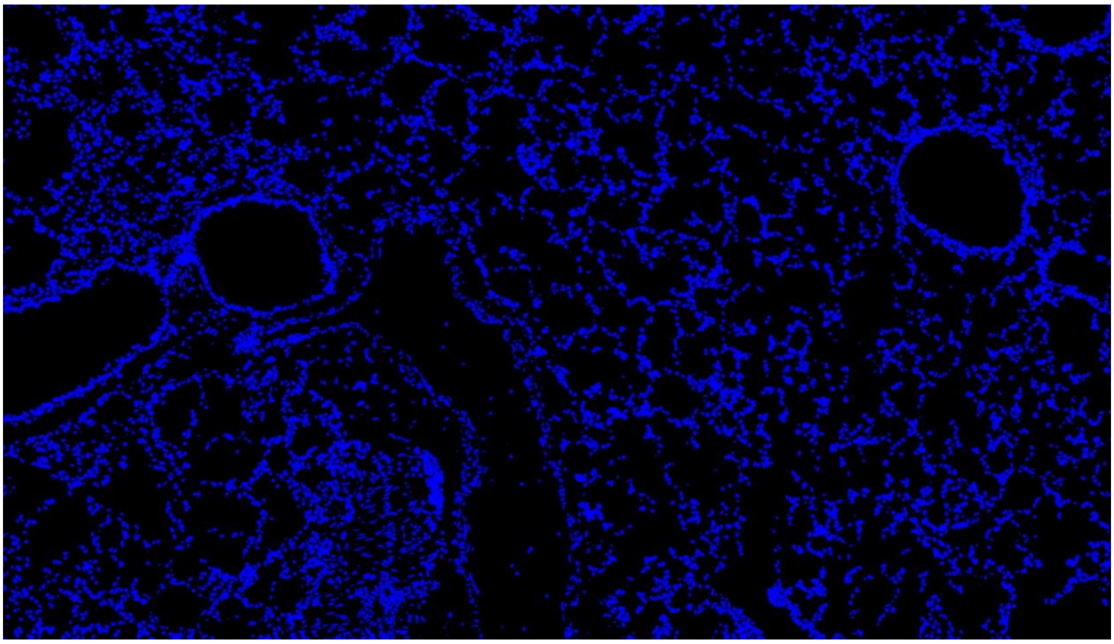

HSSD-L group TUNEL repeat 3

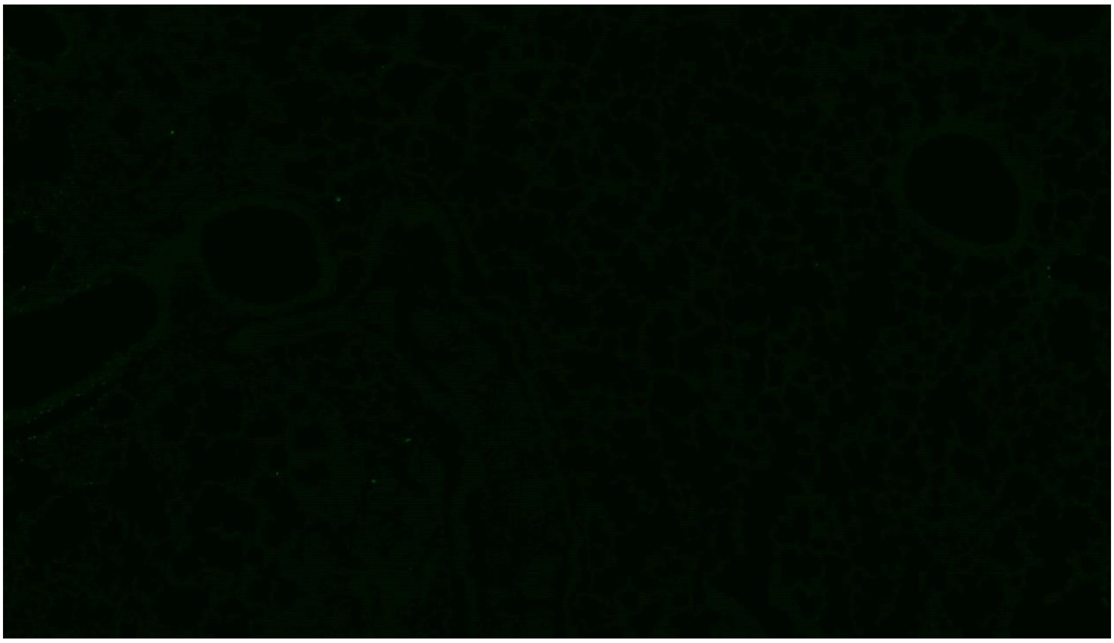

HSSD-L group Merge repeat 3

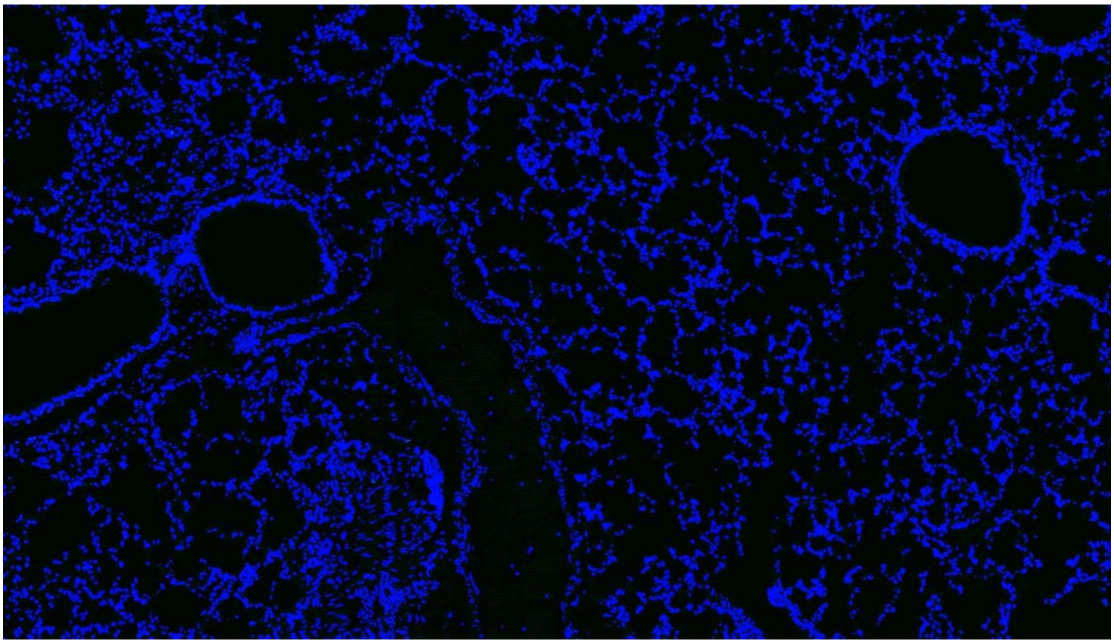

HSSD-M group DAPI repeat 1

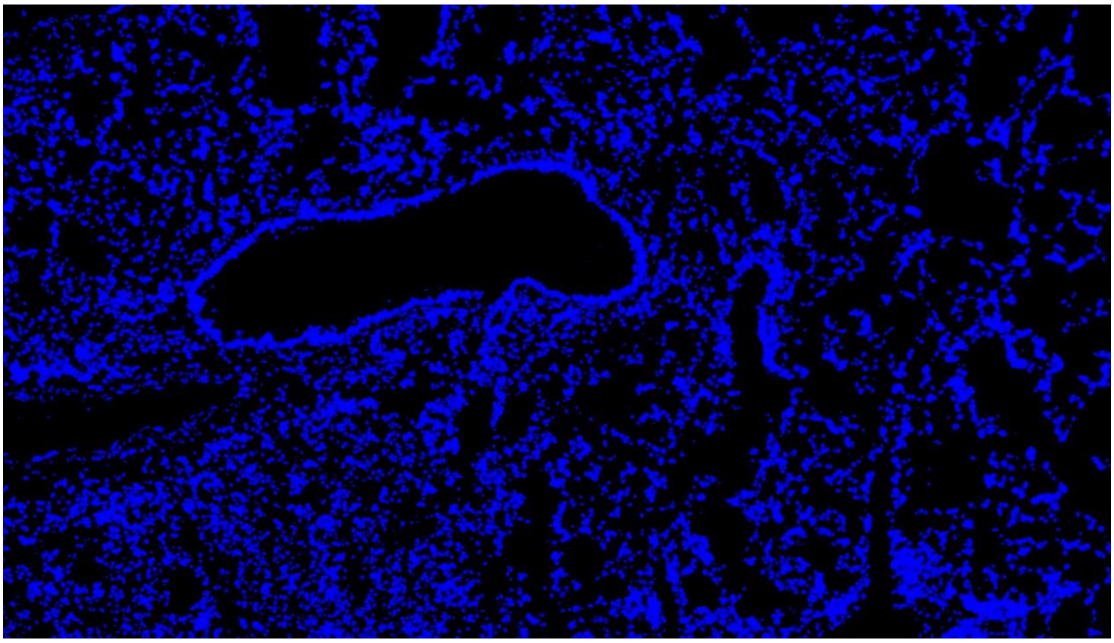

HSSD-M group TUNEL repeat 1

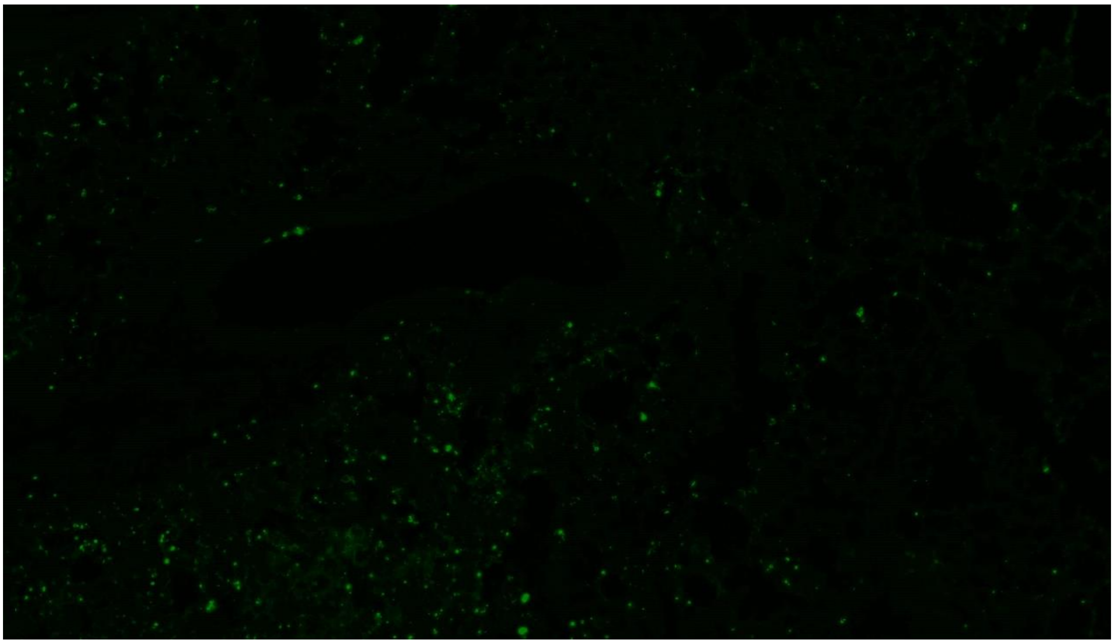

HSSD-M group Merge repeat 1

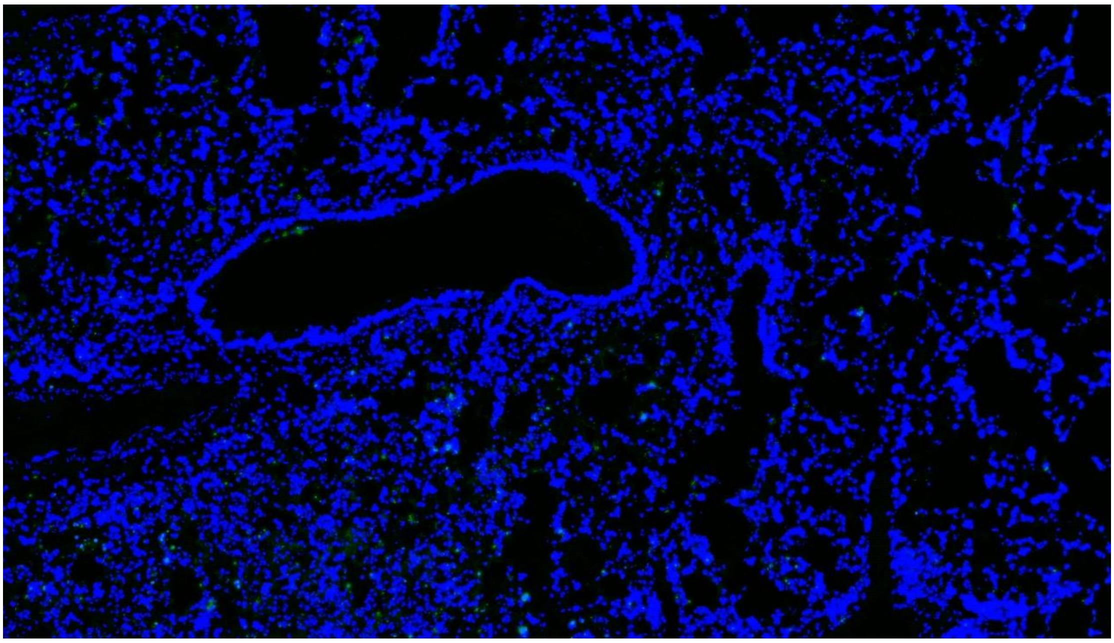

HSSD-M group DAPI repeat 2

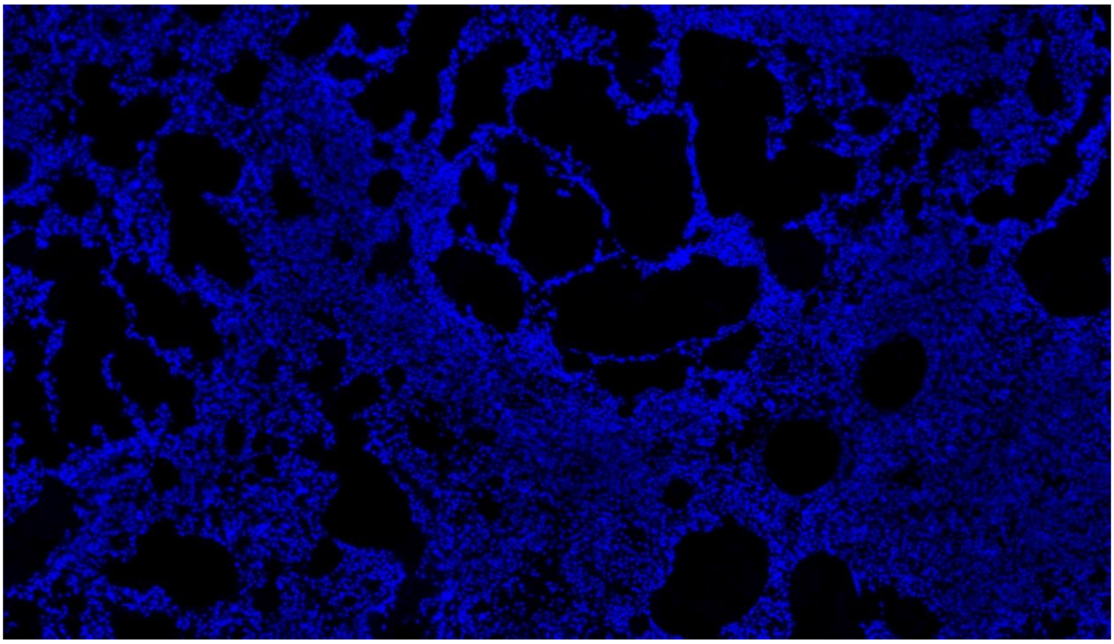

HSSD-M group TUNEL repeat 2

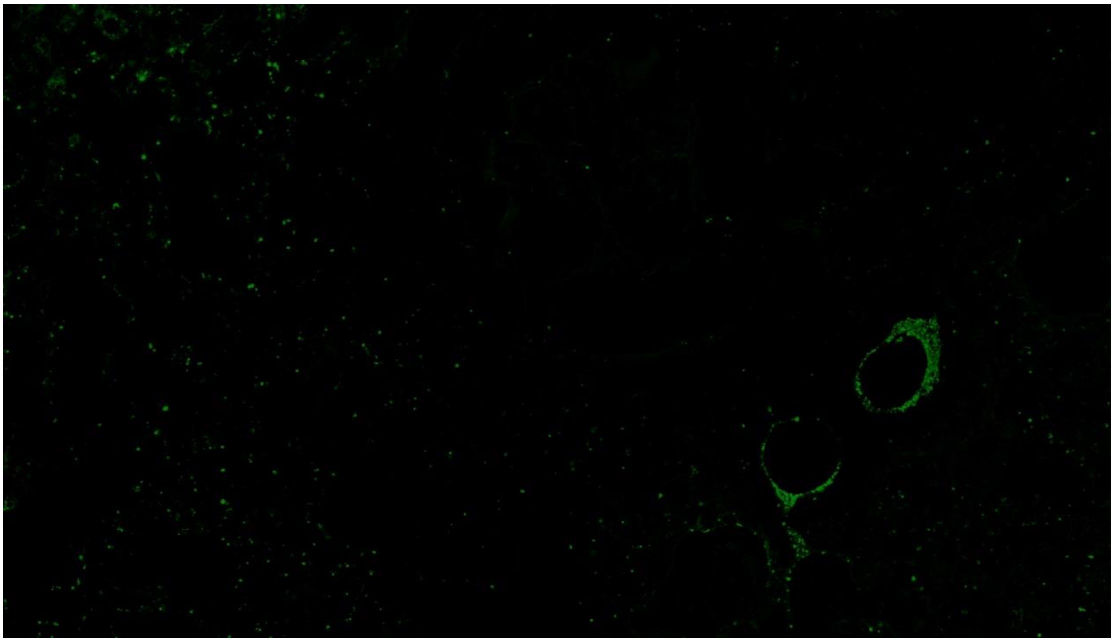

HSSD-M group Merge repeat 2

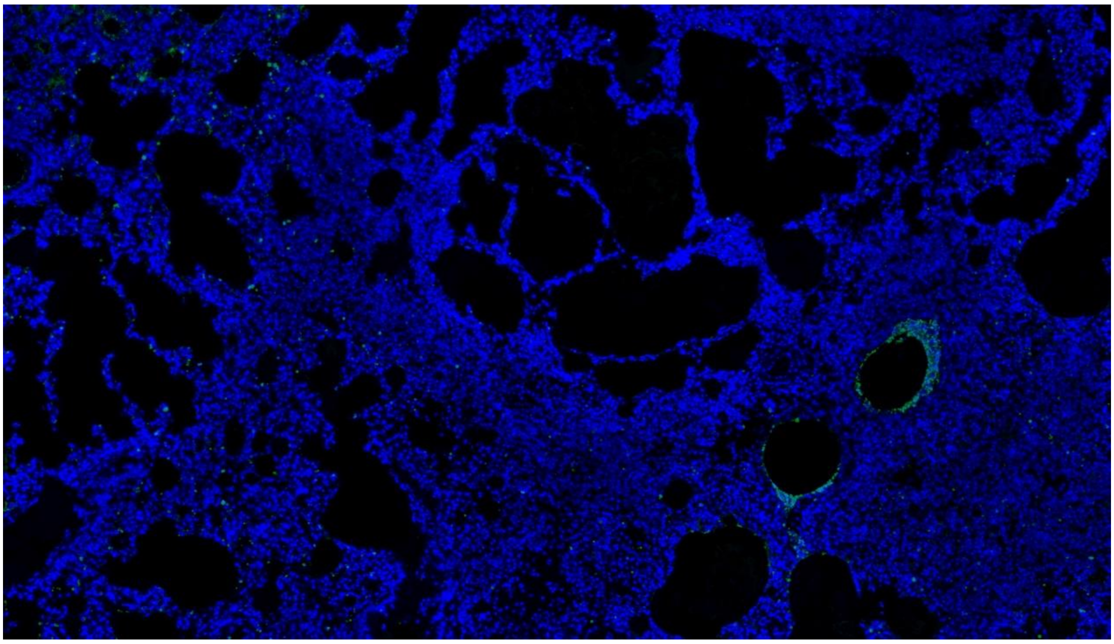

HSSD-M group DAPI repeat 3

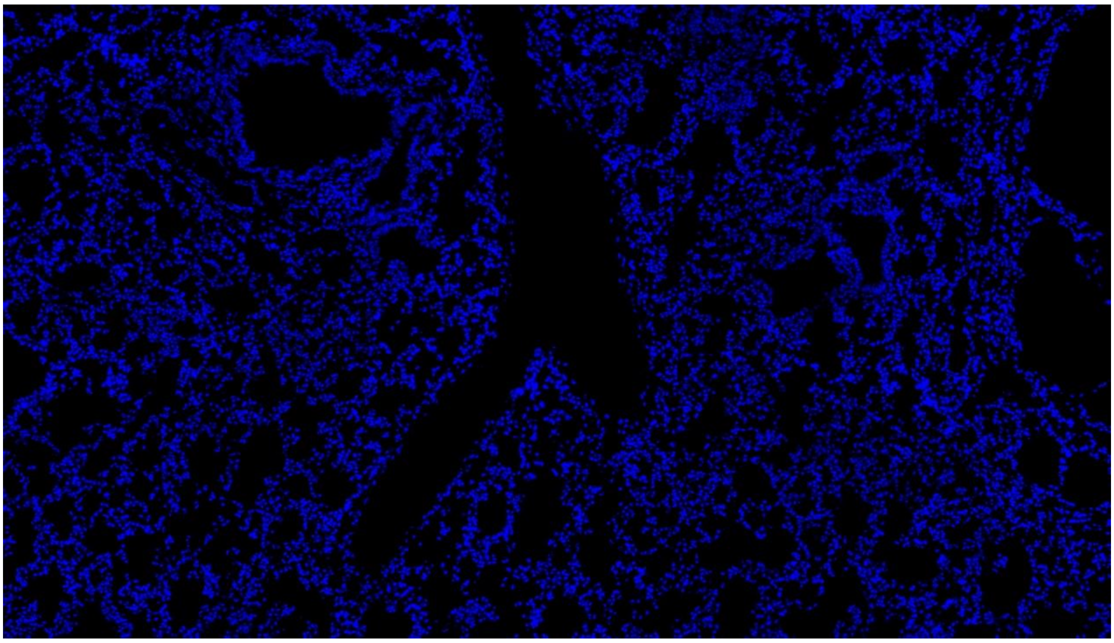

HSSD-M group TUNEL repeat 3

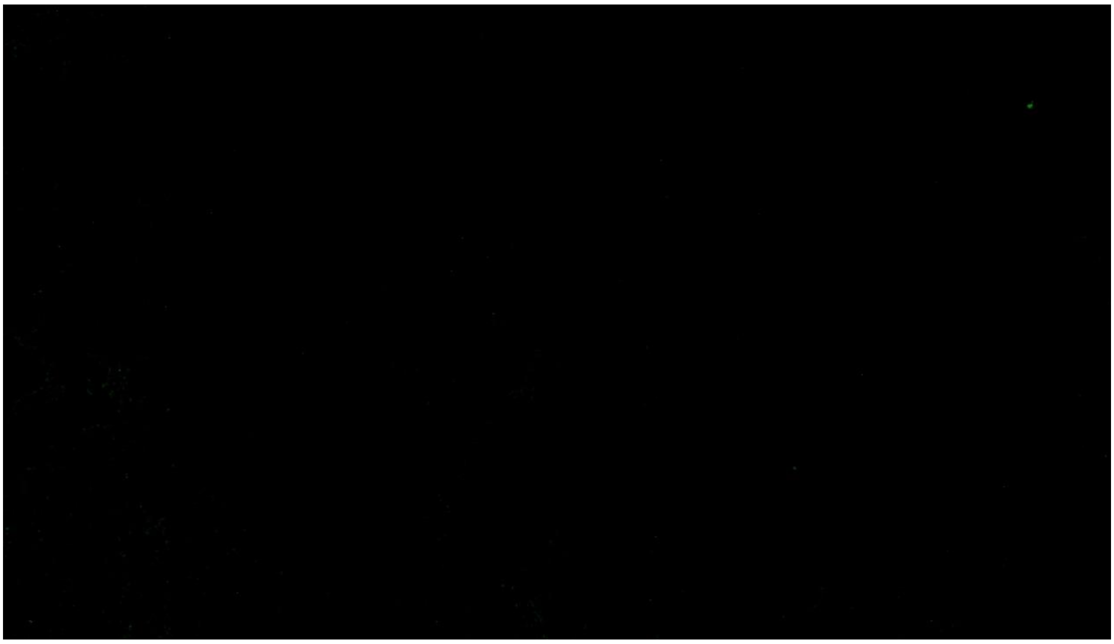

HSSD-M group Merge repeat 3

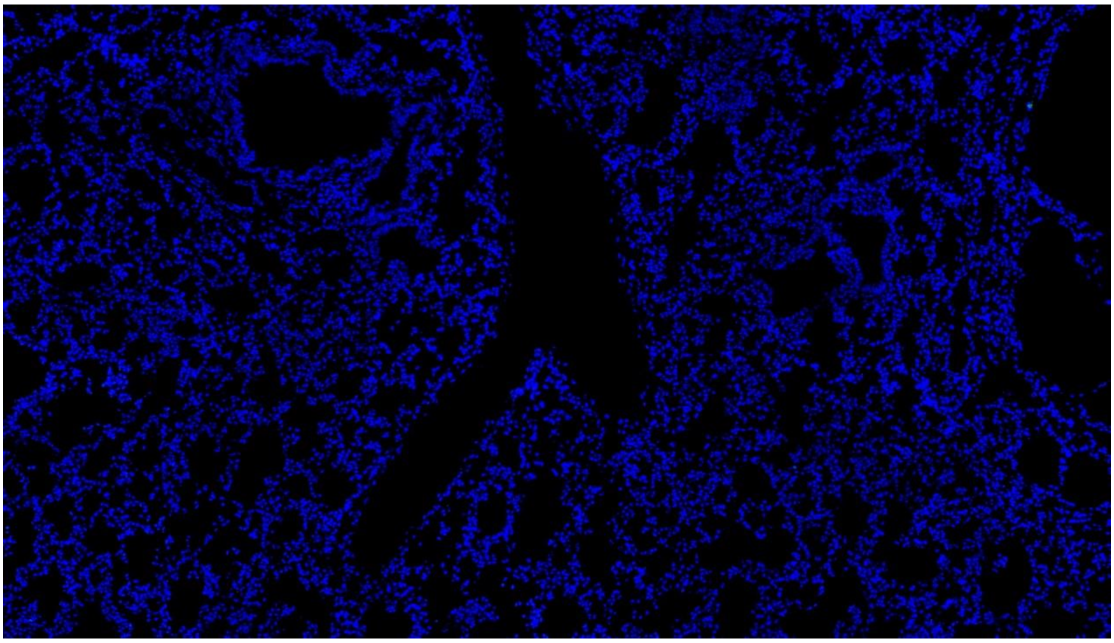

HSSD-H group DAPI repeat 1

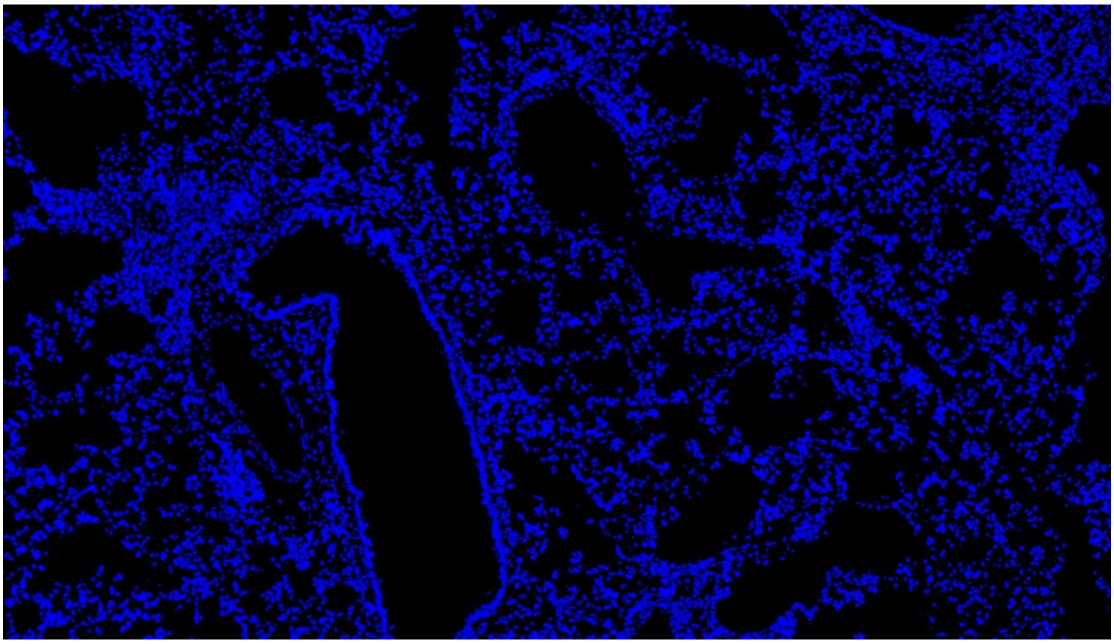

HSSD-H group TUNEL repeat 1

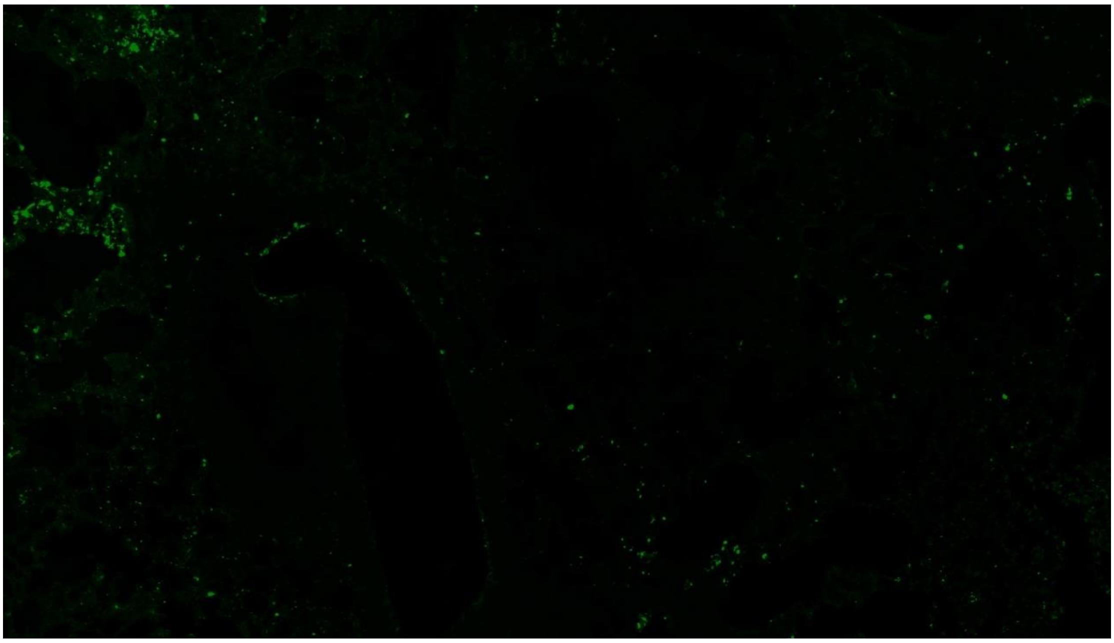

HSSD-H group Merge repeat 1

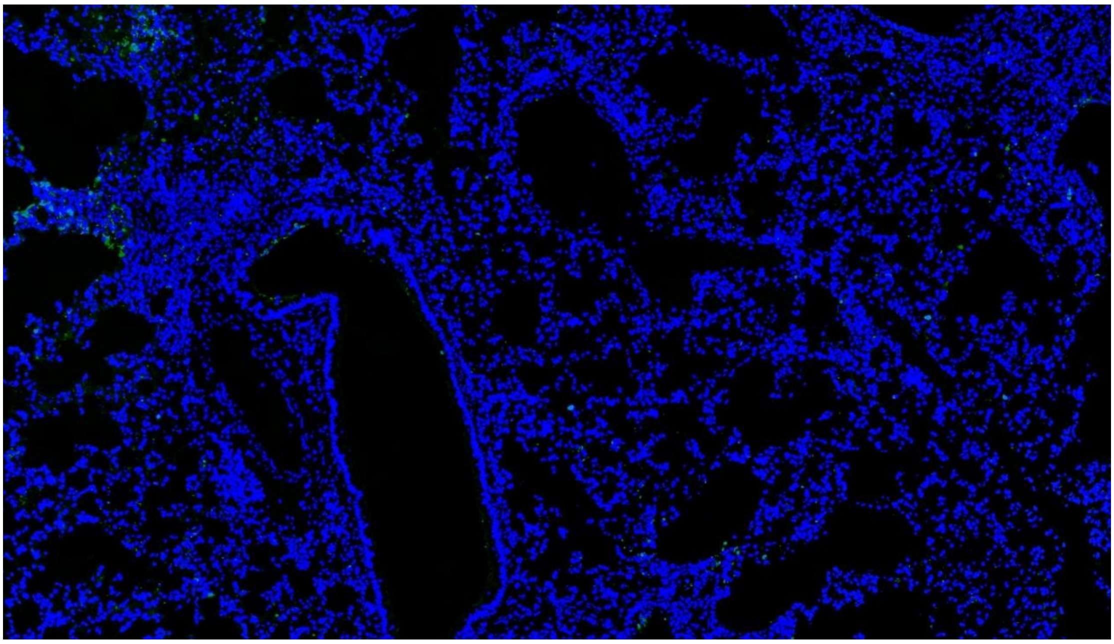

HSSD-H group DAPI repeat 2

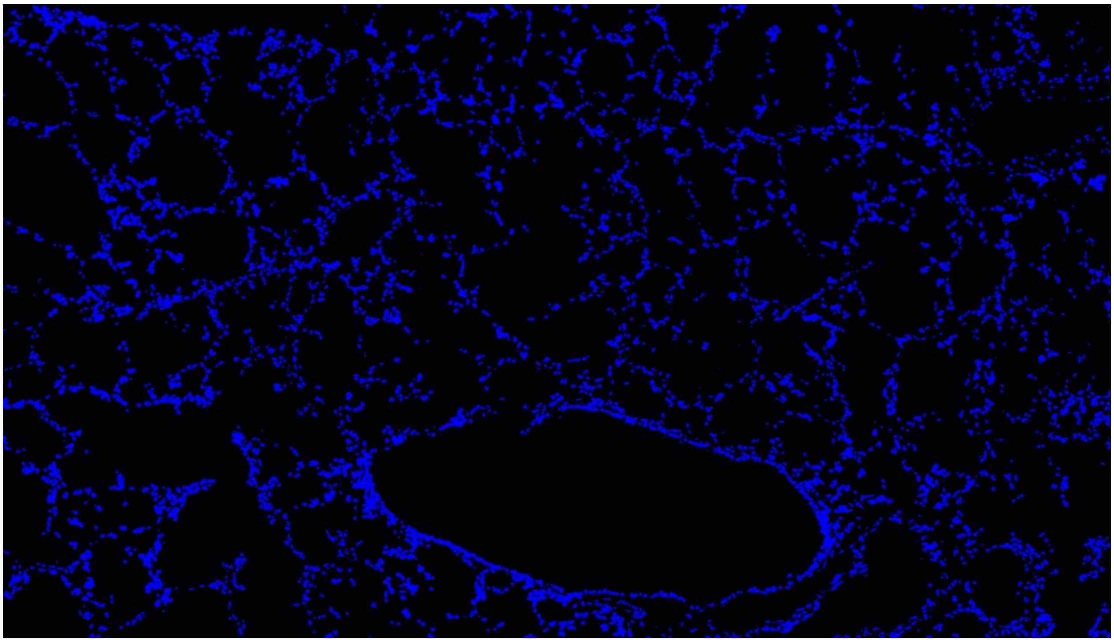

HSSD-H group TUNEL repeat 2

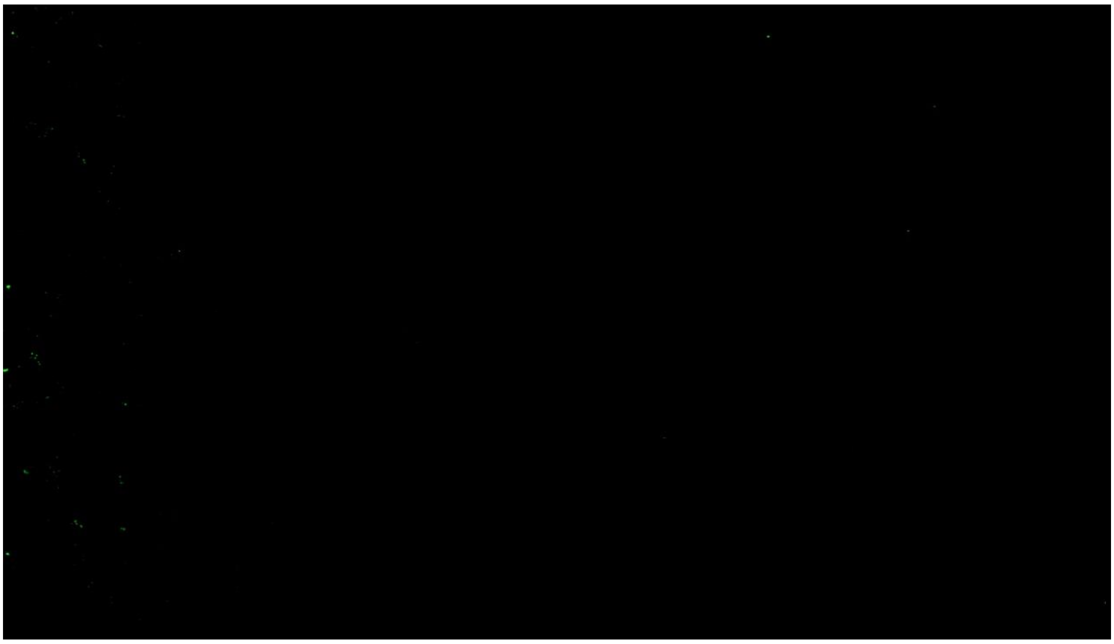

HSSD-H group Merge repeat 2

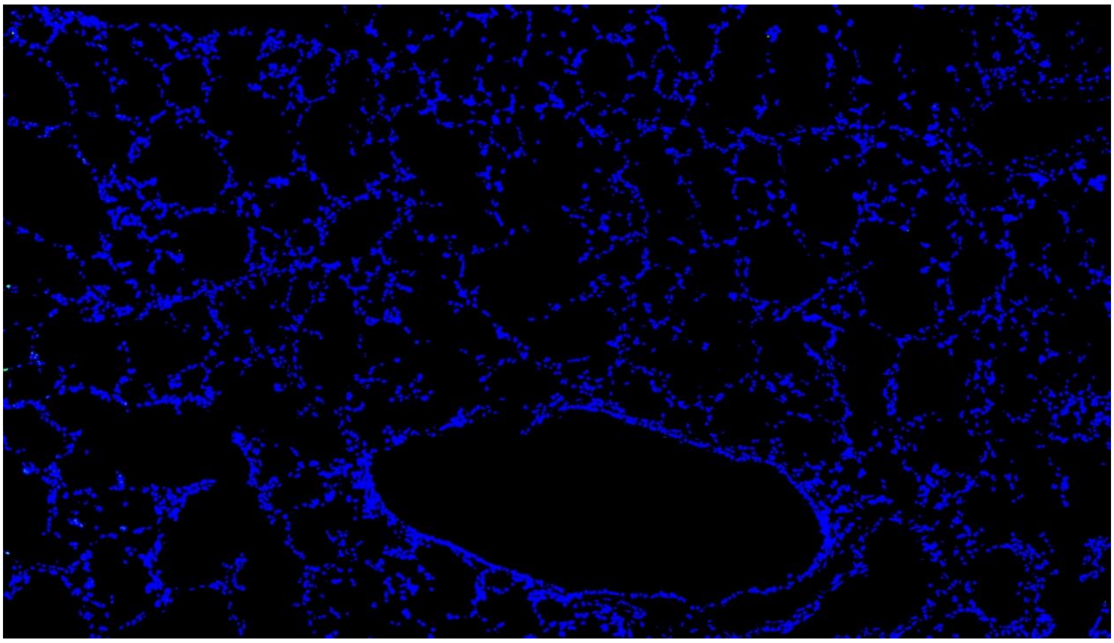

HSSD-H group DAPI repeat 3

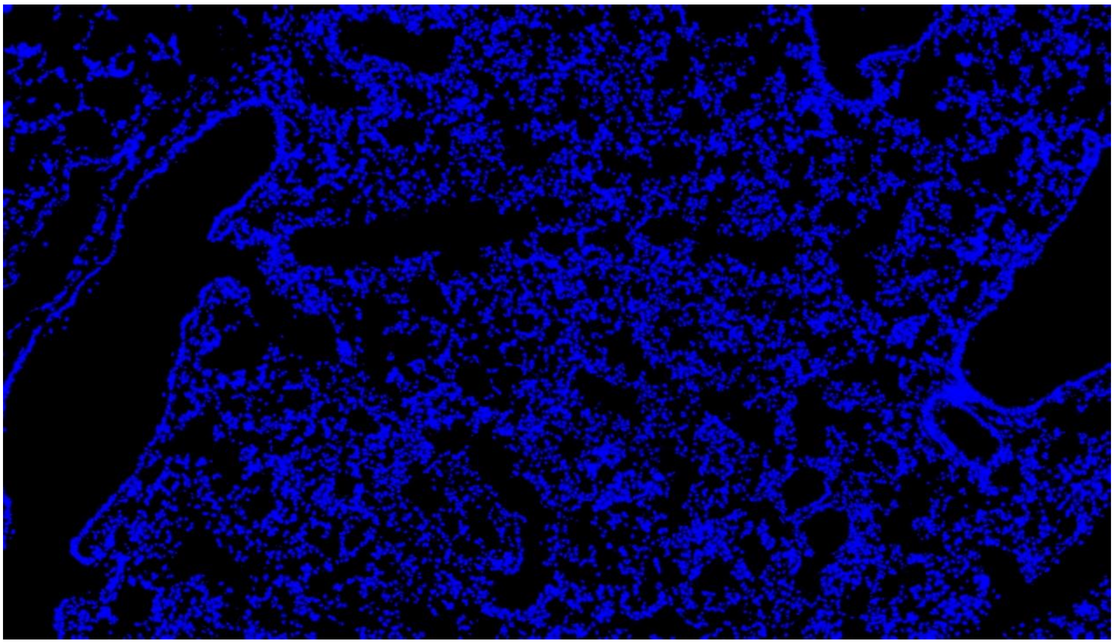

HSSD-H group TUNEL repeat 3

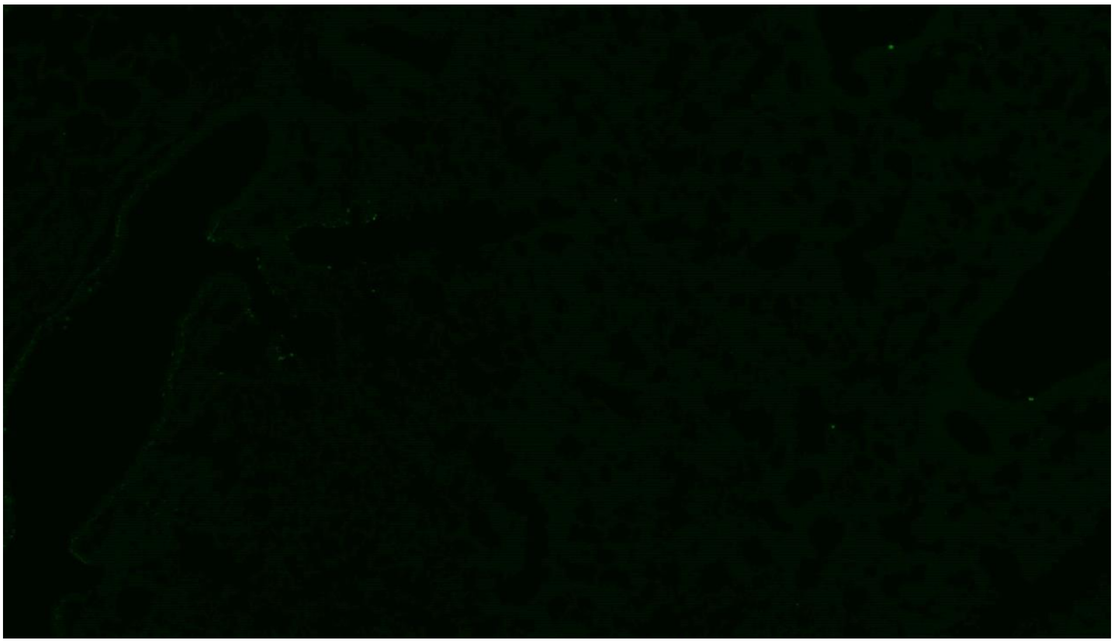

HSSD-H group Merge repeat 3

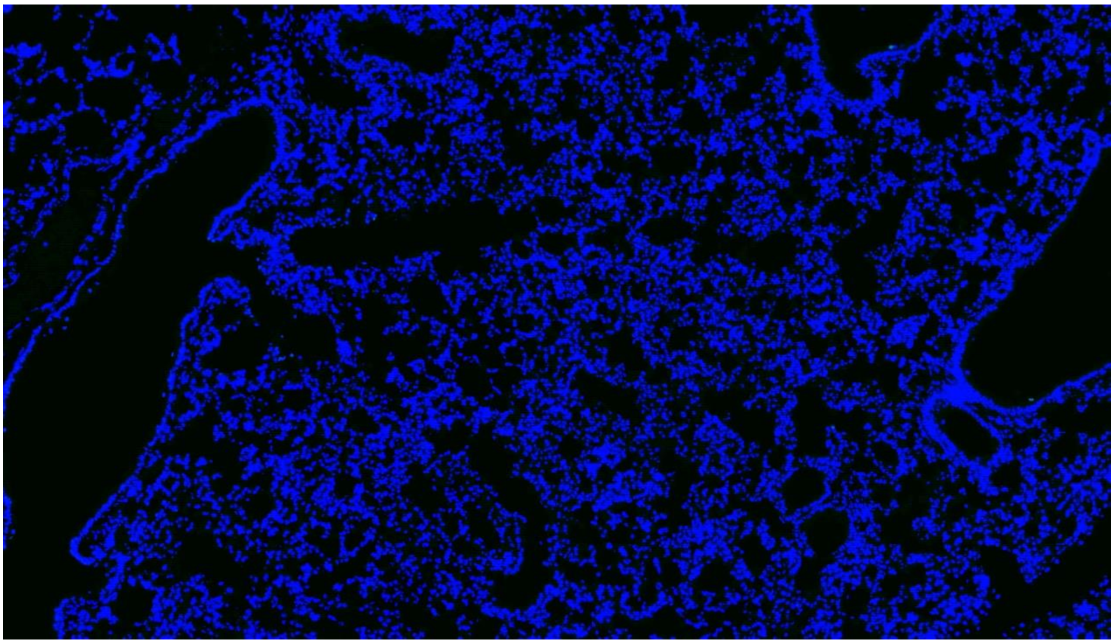

Supplement: Supplementary file 10 [file Data_Sheet_11.PDF]
