# Supplementary material for: Exploring the active ingredients and pharmacological mechanisms of the oral intake formula Huoxiang Suling Shuanghua Decoction on influenza virus type A based on network pharmacology and experimental exploration
Source: Front Microbiol. 2022 Nov 1;13:1040056. doi: 10.3389/fmicb.2022.1040056 (PMC9663660; doi:10.3389/fmicb.2022.1040056)
Supplement: Supplementary file 11 [file Data_Sheet_12.PDF]

Supplementary Data Sheet 12: Effects of HSSD on routine blood parameters in H1N1 infected mice.

The alteration on routine blood parameters from the H1N1 infected mice′ plasma were assessed in our study. Results in the following Figure revealed that GRA#, GRA%, and PLT were increased, while the LYM#, LYM%, WBC, and HGB were decreased in plasma—changes induced by H1N1 infection ($P < 0.001$). Notably, HSSD in different doses reverted the hematologic indices markedly by reducing the GRA#, GRA%, and PLT ($P < 0.001$), and increasing the LYM#, LYM%, WBC, and HGB ($P < 0.001$, or $P < 0.05$).

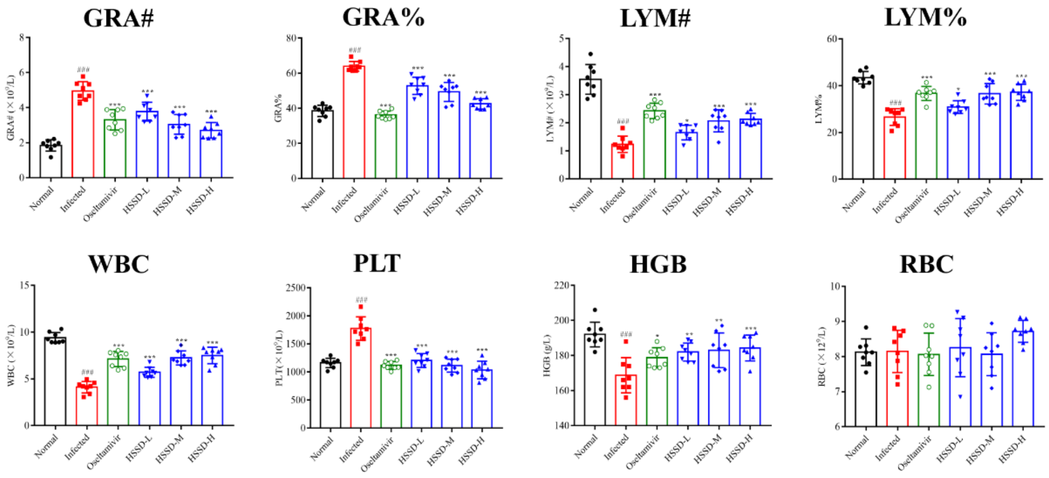

Figure S1

Effects of HSSD on routine blood parameters in H1N1 infected mice. Levels of routine blood parameters including GRA#, GRA%, LYM#, LYM%, WBC, PLT, HGB, and RBC in blood of H1N1 infected mice.
