# Supplementary material for: Exploring the active ingredients and pharmacological mechanisms of the oral intake formula Huoxiang Suling Shuanghua Decoction on influenza virus type A based on network pharmacology and experimental exploration
Source: Front Microbiol. 2022 Nov 1;13:1040056. doi: 10.3389/fmicb.2022.1040056 (PMC9663660; doi:10.3389/fmicb.2022.1040056)
Supplement: Supplementary file 5 [file Data_Sheet_6.PDF]

Supplementary Data Sheet 6: Detail information of PPI and MCODE analysis results.

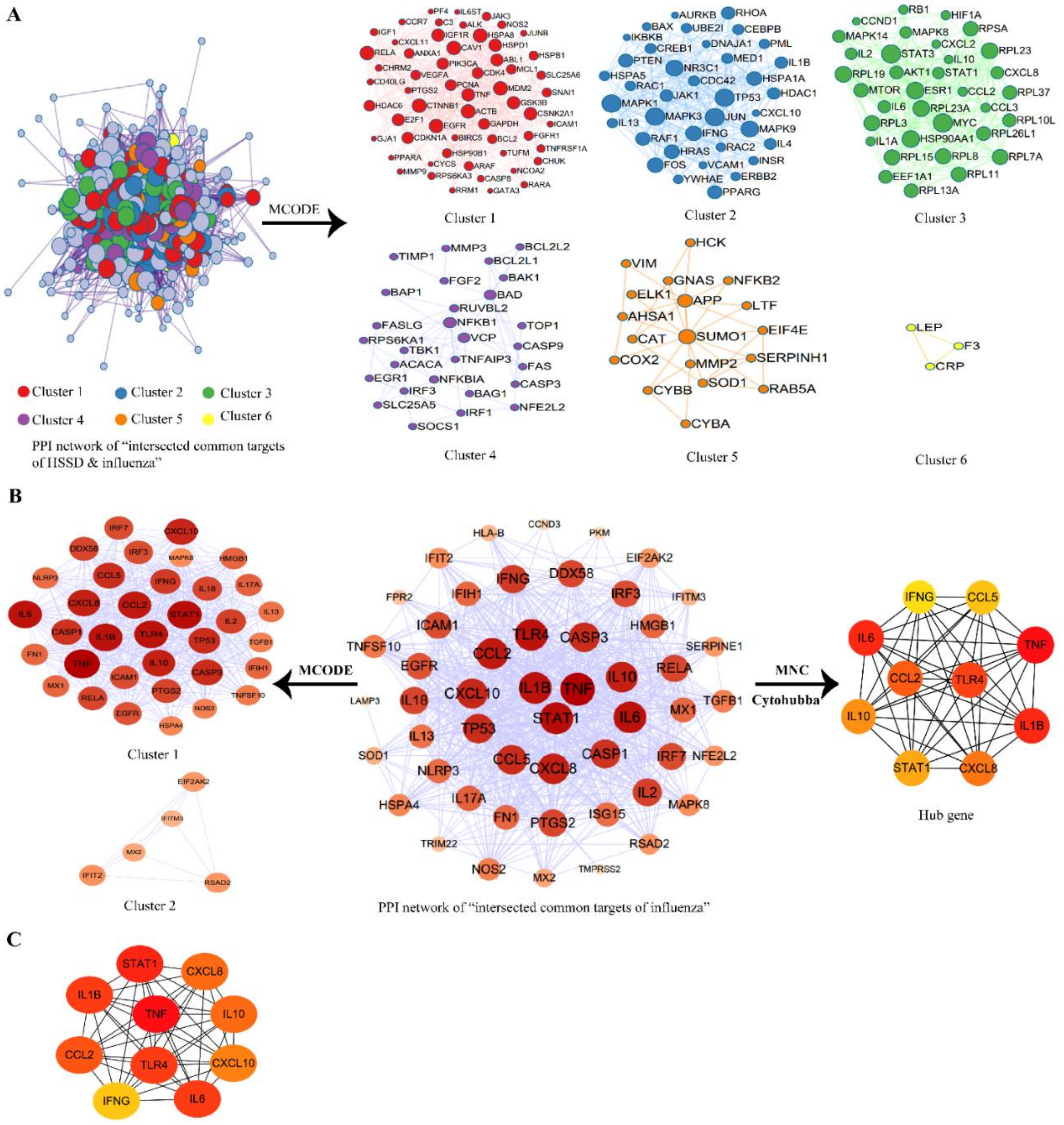

Figure S1

PPI network construction and analysis. **(A)** PPI network of "intersected common targets of HSSD & influenza" and its clusters analysis. **(B)** PPI network of "intersected common targets of influenza" and its clusters analysis, and hub gene screening. **(C)** Critical hub genes getting by intersecting "intersected common targets of HSSD & influenza" hub genes and "intersected common targets of influenza" hub genes.
